# A New Recruitable E3 Ligase UHRF1 Supporting Targeted Protein Degradation: A Minimal Azide as a Recruitment Ligand

**DOI:** 10.1002/advs.77201

**Published:** 2026-08-19

**Authors:** Zehong Lin, Kang Duan, Rui Wan, Tao Jiang, Jinzhe Wei, Miao Sun, Mariia A. Skryl'nikova, Maxim A. Gureev, Zhi‐Min Zhang, Zhang Zhang, Ke Ding, Nan Ma, Tongzheng Liu, Yi Tan, Zhengqiu Li

**Affiliations:** ^1^ State Key Laboratory of Bioactive Molecules and Druggability Assessment Jinan University Guangzhou China; ^2^ Guangdong Basic Research Center of Excellence for Natural Bioactive Molecules and Discovery of Innovative Drugs Jinan University Guangzhou China; ^3^ Department of Chemistry and Technology of Organic Nitrogen Compounds Saint‐Petersburg State Institute of Technology Saint Petersburg Russia; ^4^ Laboratory of bio‐ and chemoinformatics HSE University Saint‐Petersburg Russia; ^5^ MOE Key Laboratory of Tumor Molecular Biology Jinan University Guangzhou China

**Keywords:** azide, cell biology, chemistry, gpx4, in vivo, ligand, protein degradation, targeted therapy, therapeutic strategy, ubiquitin ligase

## Abstract

Targeted protein degradation represents a promising therapeutic strategy, yet its broader application is often limited by the scarcity of usable E3 ligases. Glutathione peroxidase 4 (GPX4) is a key target for inducing ferroptosis, but achieving sustained and potent inhibition remains challenging with conventional enzymatic inhibitors. Herein, we report the first small‐molecule GPX4 degraders that incorporate either electrophilic warheads or a minimal azide group as an E3 recruitment ligand. The azide‐based degrader **DK‐5070** effectively drives potent GPX4 degradation, achieving a DC_50_ of 17.4 nM and a Dmax of 84%, thereby outperforming larger PROTAC‐based degraders. Notably, **DK‑5070** exhibits potent antitumor activity both in vitro (IC_50_ = 47.21 nM) and in vivo (TGI = 41.8%), demonstrating significant efficacy as a GPX4 degrader. Mechanistic studies reveal that degradation is mediated through recruitment of the oncogenic E3 ligase UHRF1, which is frequently overexpressed in tumors, underscoring the potential for tumor‐specific protein degradation. This demonstrated small‐molecule degraders that recruit UHRF1 to facilitate targeted degradation of GPX4. In this system, the azide group functions as a minimal recruitment ligand, thereby expanding the E3 ligase toolbox and offering a promising strategy for targeted cancer therapy.

## Graphical abstract

Targeted protein degradation is often limited by the scarcity of usable E3 ligases. Herein, we report the first small‐molecule GPX4 degraders that incorporate either electrophilic warheads or a minimal azide group as an E3 recruitment ligand. The azide‐based degrader **DK‐5070** effectively drives potent GPX4 degradation; mechanistic studies reveal that degradation is mediated through a new recruitable E3 ligase, UHRF1.

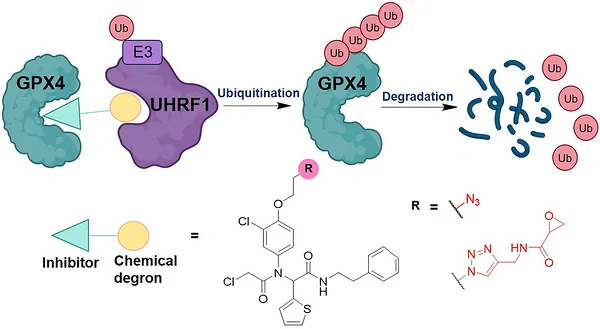

## 1 Introduction

Targeted Protein Degradation (TPD) has emerged as a highly promising therapeutic paradigm in recent years, offering a strategy to overcome key limitations associated with conventional occupancy‐driven small‐molecule drug discovery [1]. TPD operates by inducing proximity between an E3 ubiquitin ligase and a protein of interest (POI), thereby inducing ubiquitination and subsequent proteasomal degradation of the target protein [2, 3, 4]. To date, two principal classes of degraders have been developed. Proteolysis targeting chimeras (PROTACs) are heterobifunctional molecules that utilize a chemical linker to tether an E3 ligase‐recruiting ligand to a POI‐binding ligand, thereby promoting proximity between the E3 ligase and the POI [5, 6, 7, 8, 9]. In contrast, molecular glues are monovalent small molecules that act by reprogramming protein–protein interactions, promoting or stabilizing the association between a POI and an E3 ligase to form a cooperative ternary complex [10, 11, 12, 13, 14]. Despite their distinct mechanisms, both modalities face important challenges. PROTACs often suffer from high molecular weight and poor cellular permeability, whereas molecular glues, although generally more drug‐like, lack well‐defined principles for rational design. Moreover, the field remains heavily reliant on a limited set of E3 ligases, predominantly CRBN and VHL, which constrains the scope and generalizability of current TPD approaches. Efforts to expand the repertoire of E3 ligases have therefore become a central challenge in the field.

An emerging strategy to address this limitation involves the incorporation of electrophilic warheads into solvent‐exposed regions of target‐binding ligands, enabling covalent engagement of E3 ligases. This approach can generate stabilizing covalent interfaces that promote the formation of degradation‐competent complexes, thereby bypassing the need for canonical E3 ligase ligands [15, 16, 17]. Notably, such a strategy has facilitated the recruitment of diverse E3 ligases, including NEDD4, RBBP7, TRIM21, FBXO28, FBXO22, DCAF16 and TRIM28, thereby expanding the accessible E3 ligase landscape for TPD [18, 19, 20, 21, 22, 23, 24, 25, 26]. These findings suggest that covalent ligand design provides a generalizable route for discovering new degrader modalities.

Ferroptosis is an iron‐dependent form of regulated cell death driven by the accumulation of lipid peroxides on cellular membranes, and it is mechanistically distinct from apoptosis [27]. Increasing evidence implicates ferroptosis in cancer and neurodegenerative diseases, and its induction has emerged as a promising strategy to overcome therapeutic resistance [28]. Among ferroptosis regulators, glutathione peroxidase 4 (GPX4) plays a central role by reducing phospholipid hydroperoxides and maintaining membrane integrity. Accordingly, inhibition or depletion of GPX4 effectively induces ferroptosis in cancer cells. However, GPX4 remains a challenging target for conventional drug discovery. Crystallographic analyses reveal a relatively flat protein surface with limited druggable pockets [29, 30], and currently available GPX4 inhibitors, including RSL3, ML162, and ML210, rely on covalent modification of the active selenocysteine residue (Sec46) [31]. Although effective, these inhibitors often suffer from suboptimal drug‐like properties and limited selectivity, constraining their clinical applicability [32]. These limitations have motivated the development of strategies that eliminate GPX4 protein rather than merely inhibiting its enzymatic activity.

A variety of GPX4‐targeting degraders have been reported, including PROTACs recruiting CRBN, VHL, or cIAP1, as well as hydrophobic tagging (HyT)‐ and HSP70‐based degraders (Figure 1A), all of which demonstrated the feasibility of GPX4 degradation [33, 34, 35]. Nevertheless, these approaches remain constrained by high molecular weight, limited cellular permeability, and dependence on specific E3 ligase expression, highlighting the need for alternative degrader design strategies. In this work, we sought to develop compact GPX4 degraders by leveraging a covalent warhead‐based strategy. By introducing diverse electrophilic moieties into the solvent‐exposed region of the GPX4 inhibitor ML162, we generated a series of small‐molecule GPX4 degraders. From this effort, we identified **DK‐6075** and **DK‐5070** as potent GPX4 degraders. Chemoproteomic profiling and functional studies revealed that these compounds induce GPX4 degradation through covalent recruitment of UHRF1. Our findings establish a strategy for GPX4 degradation via previously unexplored E3 ligase engagement and provide a generalizable framework for expanding the E3 ligase repertoire in TPD (Figure 1B).

**FIGURE 1:**
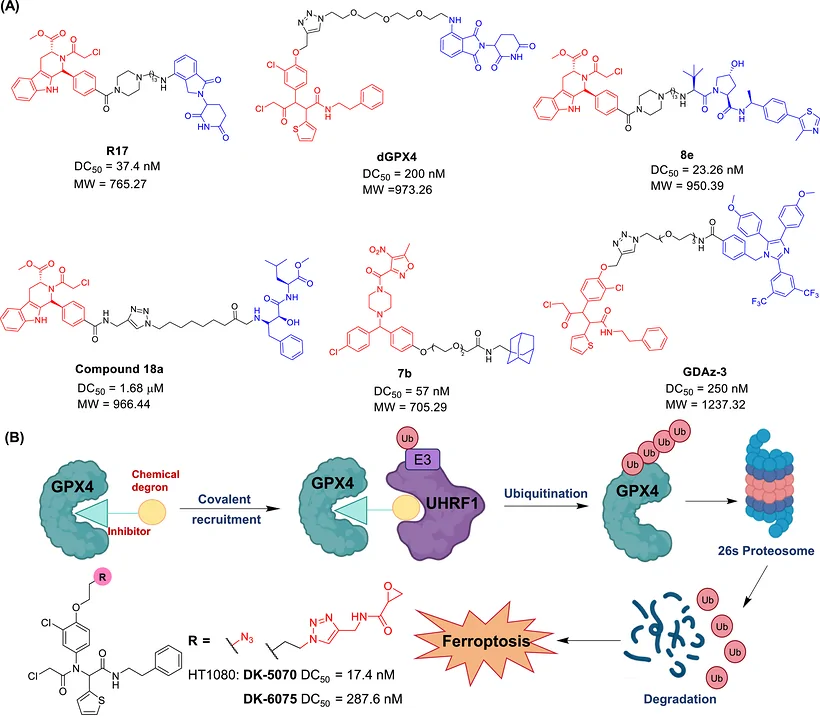
Covalent recruitment of UHRF1 for GPX4 degradation. (A) Reported GPX4 degraders. (B) Covalent recruitment of UHRF1 for GPX4 degradation via dual warhead strategy. **DK‐5070** is an azide‐based degrader, and **DK‐6075** is an epoxide‐based degrader.

## 2 Results and Discussion

### 2.1 Design, Synthesis and Structure‐Activity Relationships of GPX4 Degraders

Despite the limited number of reported ferroptosis inducers and GPX4 inhibitors, the co‐crystal structure of the ML162‐GPX4 complex (PDB: 6HKQ) provides a rationale and theoretical foundation for the design of GPX4‐targeting degraders. Given its potent anti‐GPX4 activity, ML162 was selected as the GPX4‐targeting scaffold. The chloroacetamide moiety of ML162, which covalently engages the active‐site selenocysteine, is essential for GPX4 binding and was therefore retained. In contrast, the solvent‐exposed methyl benzoate region was chosen for diversification to enable installation of electrophilic functionalities for E3 ligase recruitment. To this end, a panel of electrophilic warheads was introduced, including epoxide (**DK‐5084**), acrylamide (**DK‐6076**), and mono‐alkyl fumarate (**DK‐5072**), all of which are known to react with nucleophilic amino acid residues. In addition, sulfonyl fluoride (**DK‐5071**), a warhead widely employed in covalent inhibitor development, was incorporated [36]. Inspired by reports that alkylamines can serve as precursors for small‐molecule degraders [37], an azide group (**DK‐5070**) was incorporated as a potential reactive handle for further evaluation. Furthermore, the recently reported amino‐reactive enol ester warhead [38, 39] was also incorporated into our design. Using click chemistry‐enabled functionalization, we synthesized a panel of 19 compounds (Figure 2A). Detailed synthetic procedures and characterization data are provided in the Supporting Information.

**FIGURE 2:**
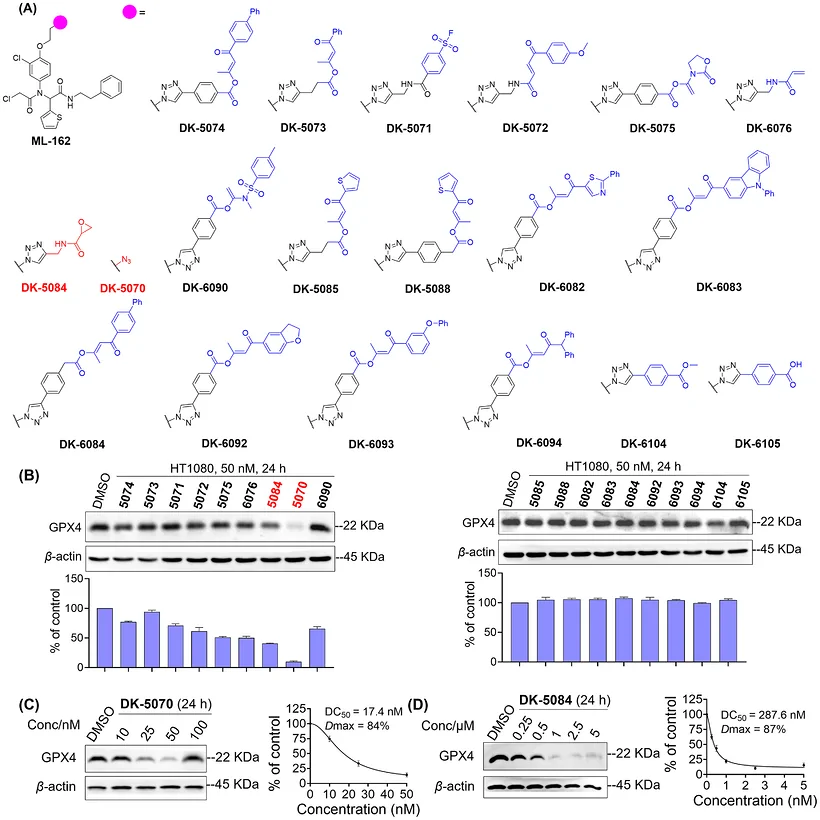
Design, synthesis and structure–activity relationships of GPX4 degraders (A) Structures of ML162 analogs wherein various electrophilic warheads were appended onto the solvent‐exposed end of ML162. (B) Evaluation of ML162 analogs with different electrophilic warheads in HT1080 cells to identify compounds that mediated degradation of GPX4. HT1080 cells were treated with DMSO or compounds (50 nM) for 24 h. GPX4 and loading control Actin levels were assessed by western blotting. (C) The dose‐response of **DK‐5070** on GPX4 degradation in HT1080 cells. GPX4 and loading control Actin levels were assessed by western blotting. (D) The dose‐response of **DK‐5084** on GPX4 degradation in HT1080 cells. GPX4 and loading control Actin levels were assessed by western blotting.

With these compounds in hand, we evaluated their GPX4 degradation activity in HT1080 cells at both high (1 µM) and low (50 nM) concentrations via immunoblotting (Figure 2B and Figure S1). **DK‐5084**, featuring an epoxide moiety as a putative covalent warhead, demonstrated robust GPX4 degradation, particularly at 1 µM, surpassing that of **DK‐5072** and **DK‐6076**, which contain fumarate‐ and acrylamide‐based warheads, respectively [17, 40], which have been previously reported to recruit the E3 ubiquitin ligases RNF126 and FBXO28 to induce potent targeted degradation. Unexpectedly, **DK‐5070**, bearing an azide‐based group, also induced pronounced GPX4 degradation following 24 h treatment at 50 nM, reducing GPX4 protein levels by more than 80%. We therefore conducted concentration‐dependent studies to compare the degradation efficacy of **DK‐5070** and **DK‐5084**. Both compounds induced strong GPX4 degradation in HT1080 cells. **DK‐5070** exhibited a half‐maximal degradation concentration (DC_50_) of 17.4 nM and a maximal degradation (Dmax) of 84% after 24 h treatment (Figure 2C), whereas **DK‐5084** showed a markedly higher DC_50_ of 287.6 nM and a Dmax of 87% (Figure 2D). Notably, a pronounced hook effect was observed for **DK‐5070** at 100 nM, suggesting that the azide group probably does not function as a covalent recruiter.

Previous studies have emphasized the critical role of the linker moiety in proximity‐induced degraders, influencing the stable formation of the ternary complex as well as the physicochemical and pharmacokinetic properties of these molecules. To investigate the impact of linker length on GPX4 degradation activity in **DK‐5070** and **DK‐5084**, we synthesized two series of compounds with varying linkers between the pharmacophore and the electrophilic warhead (Figure 3A). Within the azide‐warhead series, **DK‐5070** remained the most potent degrader overall (Figure 3B). At 30 nM, **DK‐6008** (butylamine linker) displayed GPX4 degradation comparable to **DK‐5070**; however, dose‐response analysis revealed a higher DC_50_ for **DK‐6008** (56.5 nM, Figure S2A) than for **DK‐5070** (17.4 nM), indicating reduced potency. By contrast, substitution of the alkyl linker with a PEG chain consistently impaired activity across the series, demonstrating that **DK‐5070** represents the most potent degrader. In the epoxide‐warhead series, **DK‐6075**, which incorporates a butylamine linker, emerged as the most potent degrader (Figure 3C,D). It displayed both a lower DC_50_ (74.5 nM) and a higher Dmax (90%) than **DK‐5084**. Consistent with the trend observed in the azide‐warhead series, introduction of a PEG linker markedly reduced GPX4 degradation activity, further indicating that alkyl linkers are more favorable for degradation within this structural framework. In addition, we evaluated the antiproliferative activity of these compounds bearing azide‐ and epoxide‐based warheads with varied linkers (Figure S2B,C). To benchmark the degradation efficiency of these compounds, we compared their activity with that of a previously reported CRBN‐recruiting PROTAC (dGPX4). Concentration‐dependent experiments with dGPX4 revealed a DC_50_ of 562.8 nM (Figure S2D). Notably, under comparable conditions, **DK5070** (20 nM) and **DK6075** (200 nM) achieved greater GPX4 degradation than dGPX4 at 200 nM (Figure 3E). In addition, compared with the GPX4 inhibitor ML162, **DK5070** and **DK6075** exhibited improved antiproliferative activity, with IC_50_ values of 47 nM and 298 nM, respectively (Figure 3F), indicating that GPX4 degradation confers superior cellular potency over enzymatic inhibition alone. Moreover, the antiproliferative activity of **DK5070** and **DK6075** was also superior to that of dGPX4 (IC_50_ = 351.3 nM). These results demonstrate that these small‐sized degraders exhibit superior degradation activity and anticancer efficacy. We performed qRT‐PCR to examine GPX4 mRNA levels in HT1080 cells following treatment with **DK5070** or **DK6075** (Figure 3G). The results showed that, at their respective effective degradation concentrations for 24 h, GPX4 mRNA levels were not significantly altered relative to the DMSO control group. This indicates that the observed reduction in GPX4 protein levels is not due to transcriptional downregulation or mRNA instability, but rather from compound‐induced protein degradation.

**FIGURE 3:**
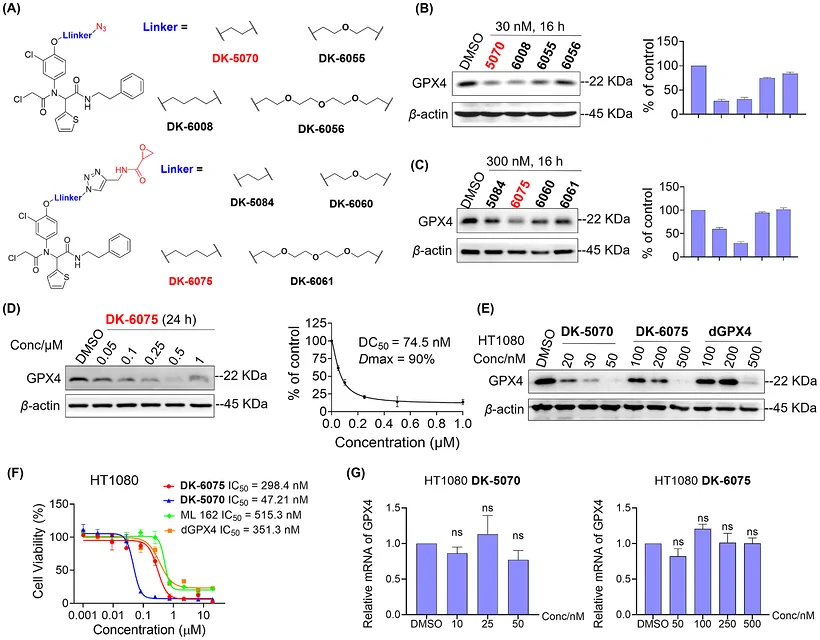
(A) Structures of degrader analogs with different linker lengths. (B) The testing of degrader analogs with different linker lengths in HT1080 cells to identify compounds that reduce GPX4 levels at 30 nM. HT1080 cells were treated with DMSO vehicle or compounds (30 nM). GPX4 and loading control Actin levels were assessed by Western blotting. (C) The testing of degrader analogs with different linker lengths in HT1080 cells to identify compounds that reduce GPX4 levels at 300 nM. HT1080 cells were treated with DMSO vehicle or compounds (300 nM). GPX4 and loading control Actin levels were assessed by Western blotting. (D) The dose‐response of **DK‐6075** on GPX4 degradation in HT1080 cells. GPX4 and loading control Actin levels were assessed by Western blotting. (E) Comparison of the degree of GPX4 degradation after 24‐h treatment with **DK‐5070**, **DK‐6075**, and dGPX4. GPX4 and loading control Actin levels were assessed by western blotting. (F) Cellular inhibition assay of ML162, dGPX4, **DK‐5070,** and **DK‐6075** against HT‐1080 cells was tested by using the CCK8 assay. The maximum probe concentration was 10 µM. (G) HT1080 cells were treated with DMSO, **DK‑5070** or **DK‑6075** for 24 h. GPX4 mRNA levels are expressed as fold change relative to DMSO. No significant changes in GPX4 mRNA were observed, indicating that the compound‑induced reduction in GPX4 protein occurs at the post‑translational level.

### 2.2 DK‐5070 and DK‐6075 Induce Rapid and Sustained Degradation of GPX4 via the Ubiquitin‐Proteasome System

Given their superior GPX4 degradation and antiproliferative activity, **DK‐5070** and **DK‐6075** were selected for subsequent mechanistic studies. To investigate the role of the azide moiety in promoting GPX4 degradation, we removed the azide from **DK‐5070** to generate **DK‐6106** (Figure 4A). For comparison, we also synthesized a triazole‐containing compound, **DK‐6100**, which contains three nitrogen atoms but exhibits distinct chemical properties relative to azide. Similarly, to probe the contribution of the epoxide warhead in **DK‐6075**, we replaced it with an unreactive cyclopropane, yielding **DK‐7057**. Given that the epoxide is expected to react with nucleophilic amino acid residues, particularly cysteines, we also introduced a reversible cysteine‐reactive cyanoacrylamide warhead to generate **DK‐6101** for comparison. Notably, replacement of either the azide moiety or epoxide eliminated the degradation activity of **DK‐5070** and **DK‐6075** (Figure 4A–C), indicating that these functional groups are essential for their activity. Subsequently, to elucidate the mechanism underlying GPX4 degradation, we next investigated how **DK‐5070** and **DK‐6075** promote target protein turnover. In TPD technologies, small‐molecule degraders typically act through either the ubiquitin‐proteasome system (UPS) or the lysosomal pathway. In our study, pretreatment with the proteasome inhibitors MG132 or bortezomib for 2 h effectively blocked **DK‐5070**‐ and **DK‐6075**‐induced GPX4 degradation (Figure 4D,E), indicating that the process is proteasome‐dependent. By contrast, the lysosomal inhibitor bafilomycin A1 failed to rescue GPX4 levels, suggesting that lysosomal degradation is not involved.

**FIGURE 4:**
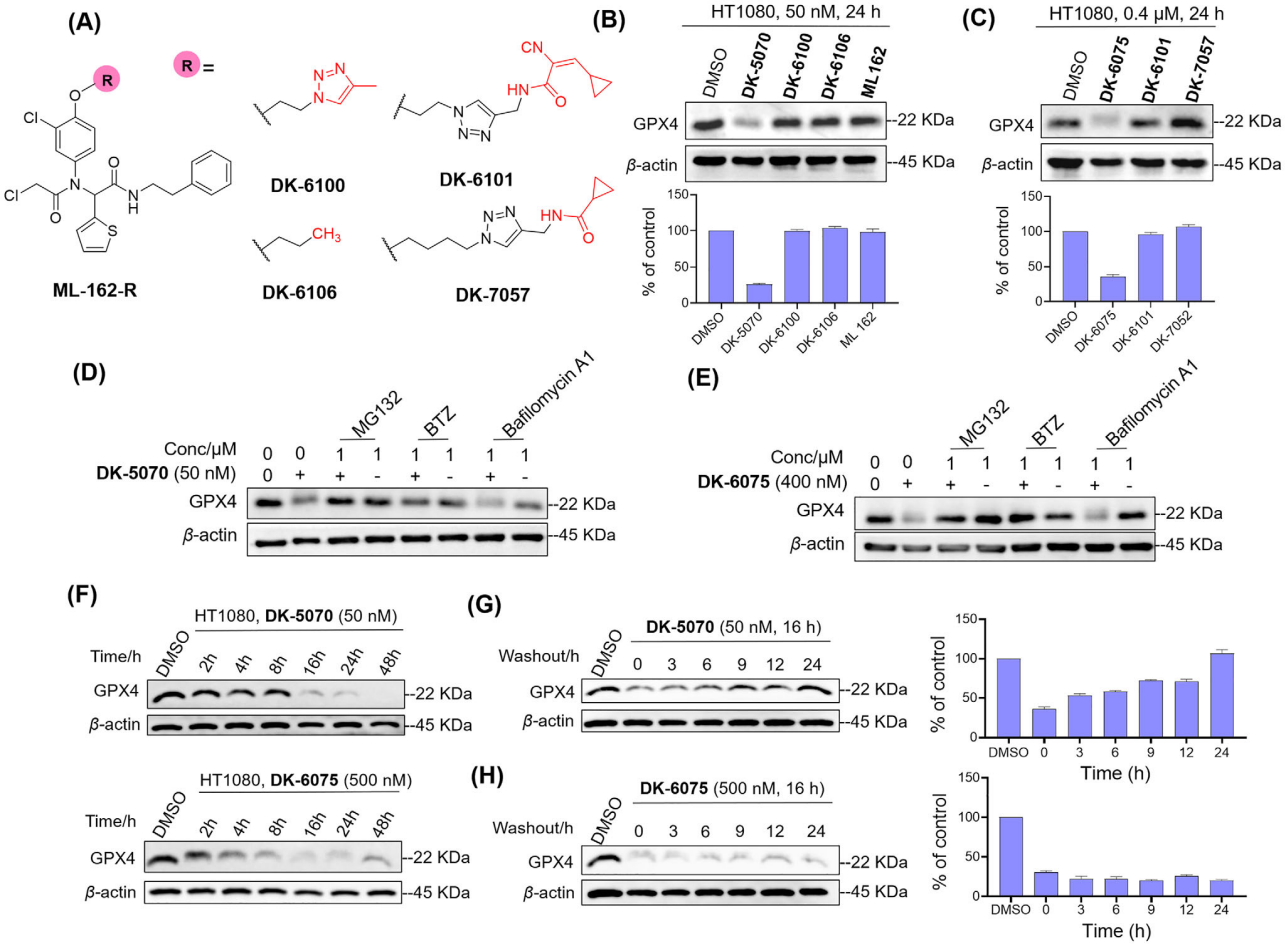
**DK‐5070** and **DK‐6075** induce rapid and durable GPX4 degradation via the ubiquitin‐proteasome system. (A) Structures of analogs generated by replacing the azide moiety or the epoxide group. (B) Testing degraders analogs in HT1080 cells to identify compounds that reduce GPX4 levels at 50 nM. HT1080 cells were treated with DMSO vehicle or compounds (50 nM) for 24 h. GPX4 and loading control Actin levels were assessed by Western blotting. (C) Testing degraders analogs in HT1080 cells to identify compounds that reduce GPX4 levels at 400 nM. HT1080 cells were treated with DMSO vehicle or compounds (400 nM) for 24 h. GPX4 and loading control Actin levels were assessed by Western blotting. (D,E) Pretreatment of HT1080 cells with MG132 or bortezomib (BTZ) or bafilomycin A1. (F) The time‐dependent‐effects of **DK‐5070** and **DK‐6075** on GPX4 degradation in HT1080 cells. GPX4 and loading control Actin levels were assessed by western blotting. Densitometric analysis of the immunoblot data is shown in Figure S6. (G) **DK‐5070** induced GPX4 degradation for up to 12 h after washout of **DK‐5070**. (H) **DK‐6075** induced GPX4 degradation for up to 24 h after washout of **DK‐6075**.

We next performed time‐course studies in HT1080 cells to examine the kinetics of GPX4 degradation. Both **DK‐5070** and **DK‐6075** induced time‐dependent decreases in GPX4 protein levels. **DK‐5070** produced substantial degradation after 16 h at 50 nM (Figure 4F), whereas **DK‐6075** markedly reduced GPX4 within 8 h and reached maximal degradation at 16 h at 500 nM. To assess the persistence of the degradation effects, washout experiments were conducted. Following 16 h treatment of **DK‐5070** or **DK‐6075**, the compounds were removed, and the GPX4 levels were evaluated by western blotting. The results indicated that the **DK‐5070**‐induced GPX4 degradation was largely reversed within 6 h (Figure 4G), whereas **DK‐6075**‐induced degradation persisted for up to 24 h with minimal recovery (Figure 4H), suggesting more sustained target engagement for the epoxide‐containing scaffold through covalent engagement. These results further support that the azide group in **DK‐5070** functions as an E3 ligase recruiter through a non‐covalent mechanism.

### 2.3 DK‐5070 and DK‐6075 induce GPX4 Degradation Through Recruitment of UHRF1

We next sought to identify the E3 ubiquitin ligase responsible for GPX4 degradation induced by **DK‐5070** and **DK‐6075**. First, we employed an activity‑based proteomics approach, a well‑established technique that uses specific probes to identify protein targets in complex systems such as cell lysates, live cells, and tissues. We introduced an alkynyl group onto the core scaffolds of **DK‑5070** and **DK‑6075** to synthesize the probe molecules **DK‑6135** and **DK‑6136** (Figure 5A,B). Subsequently, HT1080 cells were treated with **DK‑6135** or **DK‑6136** for 4 h, and after lysis, the samples were subjected to a click chemistry reaction with biotin‑N_3_ probe to label the probe‑bound proteins, while DMSO‐treated samples were used as controls to eliminate background labeling. After enrichment with streptavidin beads, the resulting samples were digested with trypsin and analyzed by LC‑MS/MS. Protein candidates were then filtered using a threshold of Log_2_(probe/DMSO) > 1.5 and *p* < 0.05 (Figure 5A,B) [23, 24]. Given that both compounds are expected to covalently modify GPX4 via the chloroacetamide warhead, the resulting ternary complexes are likely to exhibit enhanced stability. We therefore also employed co‑immunoprecipitation coupled with mass spectrometry (Co‑IP/MS) to identify the associated E3 ligases. HT1080 cells were pretreated with the proteasome inhibitor MG132, followed by treatment with alkyne‐free probe **DK‑5070** or **DK‑6075** for 8 h, with DMSO as a control. GPX4‑associated protein complexes were immunoprecipitated using a GPX4‑specific antibody, digested with trypsin, and analyzed by LC‑MS/MS. Protein hits were filtered using a threshold of Log_2_(probe/DMSO) > 2 and *p* < 0.05 (Figure 5C,D). In this study, the combination of these two complementary approaches enhanced data accuracy and helped identify the specific E3 ligase responsible for mediating GPX4 degradation. Accordingly, we performed Venn diagram analyses of the two mass spectrometry datasets obtained for **DK‑5070** and **DK‑6075**, respectively (Figure 5E,F). Comparative analysis revealed that only one overlapping candidate protein, UHRF1, was identified in the **DK‑5070** and **DK‑6135**‑treated group, whereas two overlapping proteins, UHRF1 and RBBP7, were identified in the **DK‑6075** and **DK‑6136**‑treated group. Notably, UHRF1 was commonly enriched by both experiments and thus represents the most likely candidate E3 ligase. UHRF1, an epigenetic regulator with five structural domains, is a member of the RING‐finger type E3 ubiquitin ligase family. It has been involved in the regulation of a series of biological functions, such as DNA replication, methylation, and damage repair. Aberrant overexpression of UHRF1 has been observed in many cancers and plays important oncogenic roles. However, very few probes was repored to recruit this essential E3 ligase to drive targeted protein degradation [41, 42, 43].

**FIGURE 5:**
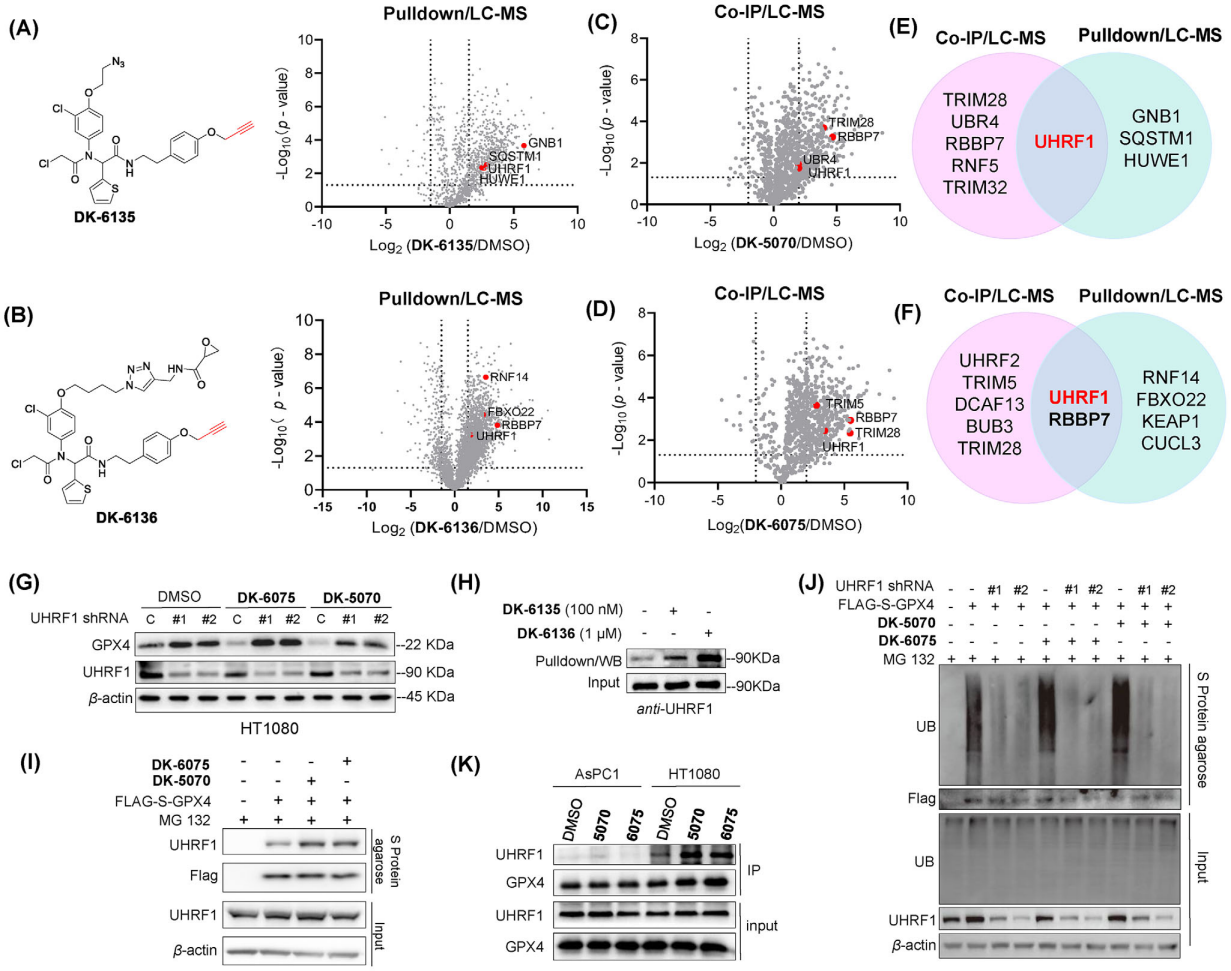
**DK‐6075** and **DK‐5070** covalently recruit UHRF1 to mediate the degradation of GPX4 via the proteasome pathway. (A,B) Structures of probes **DK‐6135** and **DK‐6136**. Protein hits identified by pull‐down/LC−MS/MS with alkyne‐handle‐containing probe **DK‐6135** (0.1 µM)/**DK‐6136** (0.5 µM); a DMSO‐treated sample was used as a control. Detailed data are provided in Tables S1 and S2. (C,D) MG132 (5 µM) was pre‐incubated in cells for 2 h, followed by 8 h with DMSO or **DK‐6075** (500 nM) / **DK‐5070** (50 nM) in HT1080 cells, respectively. Affinity enrichment is performed by immunoprecipitation using a GPX4 antibody. GPX4 was immunoprecipitated, and the interacting proteins of GPX4 protein were examined by LC‐MS/MS. Detailed data are provided in Tables S3 and S4. (E,F) Venn diagram showing the relationship between E3 ligases identified by pull‐down MS and Co‐IP MS. (G) HT1080 cells stably expressing shScramble (shScr) or shUHRF1 (#1 and #2) were treated with DMSO or **DK‐6075** (500 nM) / **DK‐5070** (50 nM) for 24 h. Western blotting was performed with the indicated antibodies. (H) Target validation by pull‐down/WB with **DK‐6135** (100 nM), **DK‐6136** (1 µM); the Western blots used UHRF1 antibodies. (I) Cells were treated with DMSO or **DK‐6075** (500 nM) / **DK‐5070** (50 nM) for 16 h, and then treated with MG132 (10 µM) for 10 h. FLAG‐GPX4 was immunoprecipitated with anti‐FLAG affinity gel, and the ubiquitination of GPX4 protein was examined by Western blotting. (J) Cells were transfected with empty vector and indicated plasmids. Cell lysates were immunoprecipitated with anti‐FLAG affinity gel and immunoblotted as indicated. (K) AsPC1 or HT1080 cells were treated with DMSO, **DK‑5070** (50 nM), or **DK‑6075** (500 nM) for 16 h, followed by immunoprecipitation (IP) with an anti‑GPX4 antibody and immunoblotting (IB) with a UHRF1 antibody. Normal rabbit IgG served as a negative control for IP. Densitometric analysis of the immunoblot data shown in figure 5H, I is shown in Figure S6.

To determine whether these three identified ubiquitination‐related proteins contribute to GPX4 degradation, we first performed stable knockdown experiments in HT1080 cells using short hairpin RNA (shRNA), followed by treatment with **DK‐6075** or **DK‐5070** for 16 h. As shown in Figure 5G, knockdown of UHRF1 effectively reversed **DK‐6075**‐ and **DK‐5070**‐mediated GPX4 degradation, demonstrating that UHRF1 is the critical E3 ligase mediating this process. Pull‐down/western blot analysis confirmed binding of UHRF1 by both probes (Figure 5H), supporting the engagement of UHRF1 with these degrader compounds. Furthermore, co‐immunoprecipitation western blotting (Co‐IP/WB) assays revealed that treatment with **DK‐6075** or **DK‐5070** promotes the interaction between UHRF1 and GPX4, enabling UHRF1 to be co‐immunoprecipitated by a GPX4‐specific antibody (Figure 5I). Consistently, surface plasmon resonance (SPR) analysis revealed that **DK‑5070** dramatically enhanced the GPX4‐UHRF1 interaction, yielding a Kd of 1.02 µM for the ternary complex (Figure S7). Collectively, these data provide direct evidence that **DK‑5070** functions as a molecular glue that efficiently recruits UHRF1 to the GPX4 surface, demonstrating positive cooperativity in ternary complex formation.

We next investigated the effect of **DK‐6075** and **DK‐5070** on UHRF1‐dependent polyubiquitination of GPX4. To this end, UHRF1 knockdown HT1080 cells reconstituted with Flag‐GPX4 were pretreated with MG‐132 to prevent proteasomal degradation, followed by immunoprecipitation of Flag‐GPX4 and immunoblotting for ubiquitin. As shown in Figure 5J, UHRF1 knockdown markedly reduced **DK‐5070**‐ and **DK‐6075**‐induced GPX4 polyubiquitination and attenuated compound‐dependent GPX4 degradation. Notably, UHRF1 knockdown also decreased basal GPX4 polyubiquitination in the absence of either compound (Figure 5J, left lanes), suggesting a role of UHRF1 in maintaining steady‐state GPX4 ubiquitination. Collectively, these results demonstrated that **DK‐5070** and **DK‐6075** effectively recruit UHRF1 and enhance UHRF1‐dependent ubiquitination and proteasomal degradation of GPX4. UHRF1 is an oncogenic E3 ligase that is frequently overexpressed in diverse solid and hematologic tumors. It contributes to tumorigenesis by epigenetically silencing tumor suppressor genes. Its preferential expression in tumor tissues over normal tissues makes UHRF1 an attractive target for molecular glue‐mediated targeted protein degradation, potentially enabling tumor‐selective therapies with reduced off‐target toxicity (Figure S3). These represent the small molecules that function by recruiting this essential oncogenic E3 ligase to facilitate targeted protein degradation.

To assess whether GPX4 degradation activity correlates with endogenous UHRF1 expression levels, we selected the human pancreatic cancer cell line AsPC1, which exhibits high UHRF1 expression, as a comparative model and examined the effects of **DK‑5070** and **DK‑6075**. In contrast to HT1080 cells, neither **DK‑5070** nor **DK‑6075** induced appreciable GPX4 degradation in AsPC1 cells (Figure S4A). To investigate the basis for this differential response, we performed co‑immunoprecipitation (Co‑IP) experiments (Figure 5K). In HT1080 cells, a weak interaction between GPX4 and UHRF1 was observed under basal conditions, and this interaction was markedly enhanced upon treatment with **DK‑5070** or **DK‑6075**. In contrast, in AsPC1 cells, no appreciable basal interaction between GPX4 and UHRF1 was detected, and compound treatment failed to induce their proximity. These results suggest that UHRF1‑mediated GPX4 degradation may rely on the intrinsic spatial proximity or pre‑existing weak interaction between GPX4 and UHRF1 within the cellular context, and that such “proximity relationships” may vary across cell lines, thereby influencing degrader potency.

To further clarify the mode of action of **DK‑5070**, we knocked down GPX4 in HT1080 cells using shRNA and repeated the pull‑down/WB assay (Figure S4B). The results showed that upon GPX4 knockdown, the ability of **DK‑6135** to pull down UHRF1 was markedly diminished, indicating that the binding of **DK‑5070** to UHRF1 is largely dependent on the presence of GPX4. This suggests that **DK‑5070** likely exerts its degradation function by stabilizing or inducing a previously weak interaction between GPX4 and UHRF1, thereby forming a ternary complex. This behavior is more consistent with a molecular glue‑like mechanism, rather than the independent binding mode characteristic of PROTACs.

### 2.4 DK‐6075 Covalently Engages UHRF1 at Cys170 to Mediate GPX4 Degradation

We hypothesize that **DK‐6075** promotes GPX4 degradation by covalently modifying a cysteine residue in UHRF1 (Figure 6A). To identify the site of modification, we performed proteomic mapping using recombinant UHRF1 protein. To avoid potential interference from the chloroacetamide moiety in **DK‐6075** during site identification, we synthesized an analog, **DK‐7062**, in which the chlorine atom was replaced with a methyl group. Following incubation of **DK‐7062** with recombinant UHRF1, tryptic digests were analyzed by LC‐MS/MS, revealing covalent modification at cysteine 170 (C170) (Figure 6B and Table S5). Crystallographic analysis further showed that C170 is solvent‐exposed on the protein surface (Figure 6C), making it accessible for covalent engagement. To validate the functional relevance of this site, we generated a UHRF1 C170A mutant and expressed it in HT1080 cells. Upon treatment with **DK‐6075** or **DK‐5070** for 24 h, the C170A mutation markedly impaired **DK‐6075**‐induced GPX4 degradation (Figure 6D), while having no effect on **DK‐5070**‐mediated degradation (Figure 6E), indicating that C170 is the critical covalent binding site for **DK‐6075**, but is not required for **DK‐5070** activity. Moreover, co‐immunoprecipitation further confirmed that **DK‐6075** promotes the interaction between UHRF1 and GPX4 in a C170‐dependent manner (Figure 6F). Taken together, these results demonstrate that **DK‐6075** recruits UHRF1 through covalent binding of C170, facilitating formation of a GPX4–**DK‐6075**–UHRF1 ternary complex and driving degradation of GPX4 through the ubiquitin‐proteasome system. In contrast, **DK‐5070** recruits UHRF1 through a non‐covalent mechanism independent of C170, highlighting the dual strategies by which UHRF1 can be exploited for targeted protein degradation. The modification site and corresponding binding pocket identified here could guide the future design and development of recruitment ligands targeting UHRF1.

**FIGURE 6:**
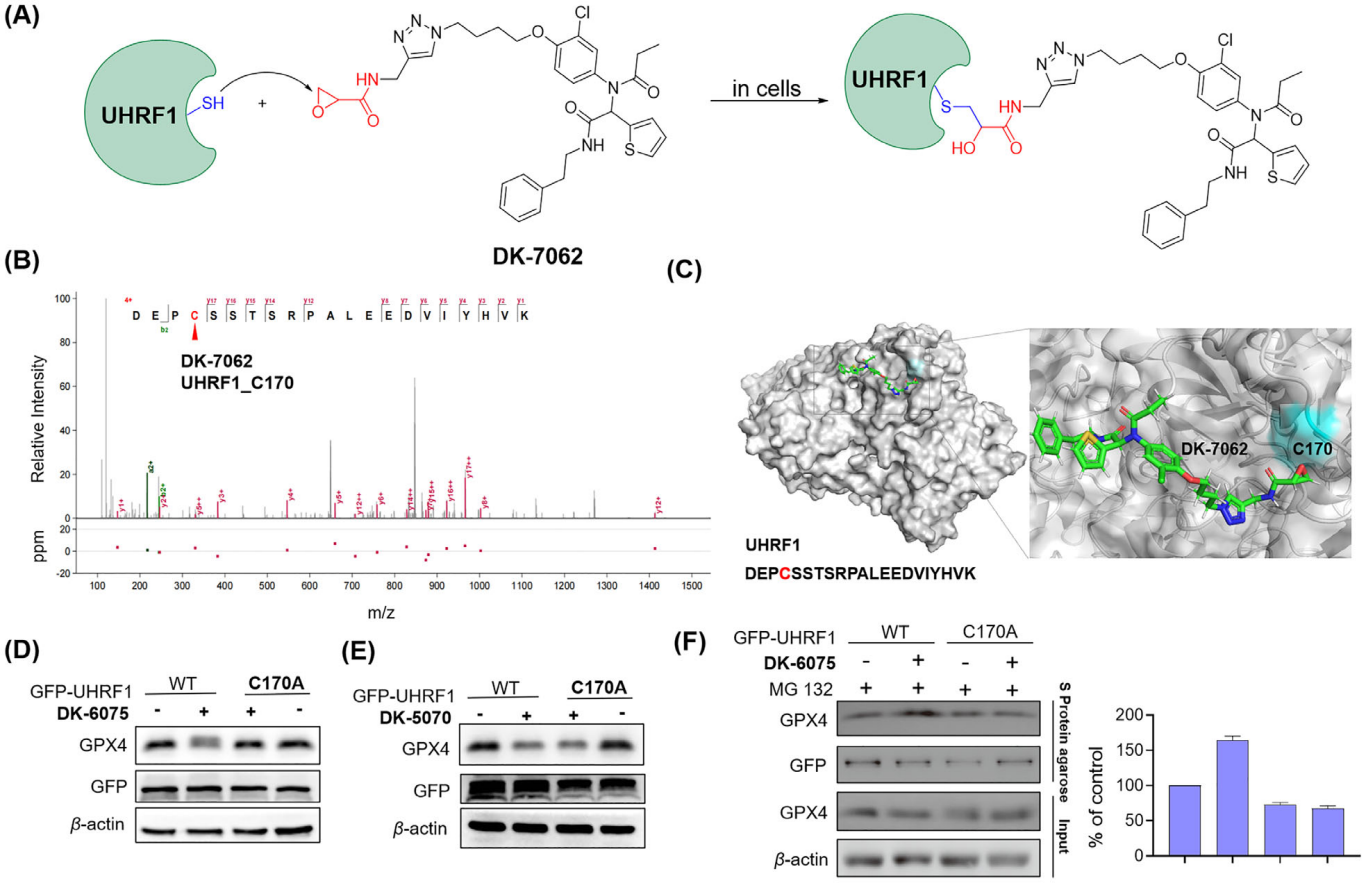
UHRF1 C170 is involved in **DK‐6075**‐mediated degradation of GPX4. (A) Proposed mechanism of **DK‐7062** recruiting UHRF1 by covalent modification of a cysteine residue. (B) LC−MS/MS spectra of DK‐7062‐labeled peptide from UHRF1, highlighted in red. (C) Docking experiments showed that **DK‐7062** can label UHRF1 at C170 on the protein surface. This result was predicted by AlphaFold. (D) HT1080‐GFP‐GPX4 cells stably expressing GFP‐S‐UHRF1 WT or GFP‐S‐UHRF1 C170A were treated with DMSO or **DK‐6075** (0.3 µM) for 24 h, and Western blotting was performed with the indicated antibodies. (E) HT1080‐GFP‐GPX4 cells stably expressing GFP‐S‐UHRF1 WT or GFP‐S‐UHRF1 C170A were treated with DMSO or DK‐5070 (30 nM) for 24 h, and Western blotting was performed with the indicated antibodies. (F) HT1080‐GFP‐GPX4 cells stably expressing GFP‐S‐UHRF1 WT or GFP‐S‐UHRF1 C170A were treated with DMSO or **DK‐6075** (0.3 µM) for 16 h. GFP‐GPX4 was pulled down by S‐protein agaroses, and GFP‐GPX4 was examined by Western blotting. Densitometric analysis of the immunoblot data shown in Figure 6D,E is shown in Figure S6.

### 2.5 Molecular Docking Reveals the Binding Modes of DK‑5070 and the Epoxide‑Containing Degrader to UHRF1

To elucidate the molecular basis underlying the recruitment of UHRF1 by our degraders, particularly the unexpected finding that the minimal azide moiety in **DK‑5070** can mediate effective binding, we performed molecular docking studies using the crystal structure of the tandem Tudor domain of UHRF1 (PDB: 6W92) [44]. Given that our epoxide‑containing degraders (**DK‑5084** and **DK‑6075**) share an identical epoxide warhead and ML162‑derived scaffold, we used **DK‑5084** as a representative for covalent docking (with epoxide ring‑opening), and standard non‑covalent docking was performed for **DK‑5070**, followed by MM‑GBSA binding free energy calculations. As shown in Figure 7A, **DK‑5084** binds to UHRF1 through a covalent linkage formed between its epoxide warhead and Cys172 (corresponding to Cys170 in the uniprot‐reported sequence) via an epoxide ring‑opening reaction. The ligand adopts an orientation that maintains accessibility of the 2‐chloroacetamide moiety for subsequent interaction with GPX4, consistent with its dual‑warhead design. Notably, **DK‑6075** contains the identical epoxide warhead and the same ML162‑based core structure, differing only in the linker length. Given that the covalent reactive group and its spatial presentation are conserved between the two compounds, the binding mode observed for **DK‑5084** is expected to be representative of **DK‑6075**. This prediction is strongly supported by our experimental data (Figure 6D). In striking contrast, **DK‑5070** engages UHRF1 through a non‑covalent binding mode (Figure 7B). The azide group forms a salt bridge with Glu131 (Glu129) and simultaneously participates in π‑stacking interactions with Trp129 (Trp127). The phenylethyl moiety is oriented toward a lipophilic pocket composed of Ala165 and Pro166, providing additional hydrophobic stabilization. This non‑covalent binding mode is further supported by the observation that the C170A mutation had no effect on **DK‑5070**‑mediated GPX4 degradation (Figure 6E), confirming that **DK‑5070** does not rely on covalent modification of Cys170 for its activity. Docking scores and binding free energies are summarized in Table S1. **DK‑5070** exhibited a superior docking score (GScore = −6.92 kcal/mol) compared to **DK‑5084** (−5.96 kcal/mol), primarily driven by stronger lipophilic interactions (Lipo: −2.439 vs. −0.95 kcal/mol). The MM‑GBSA calculated binding free energy further supported the higher predicted affinity of **DK‑5070** (ΔG = −64.32 kcal/mol) over **DK‑5084** (ΔG = −46.05 kcal/mol). These results suggest that the azide group, despite its small size, achieves effective binding through a combination of polar contacts (salt bridge and π‑stacking) and hydrophobic anchoring, providing a structural rationale for the “minimal azide recruiter” concept. Taken together, the molecular docking analyses reveal that the epoxide‑containing degraders (exemplified by **DK‑5084** and extended to **DK‑6075**) and **DK‑5070** employ distinct mechanisms, covalent vs. non‑covalent, to engage UHRF1, both of which are compatible with ternary complex formation with GPX4. These findings provide a structural framework for understanding UHRF1 recruitment and for guiding future optimization of UHRF1‑targeting degraders.

**FIGURE 7:**
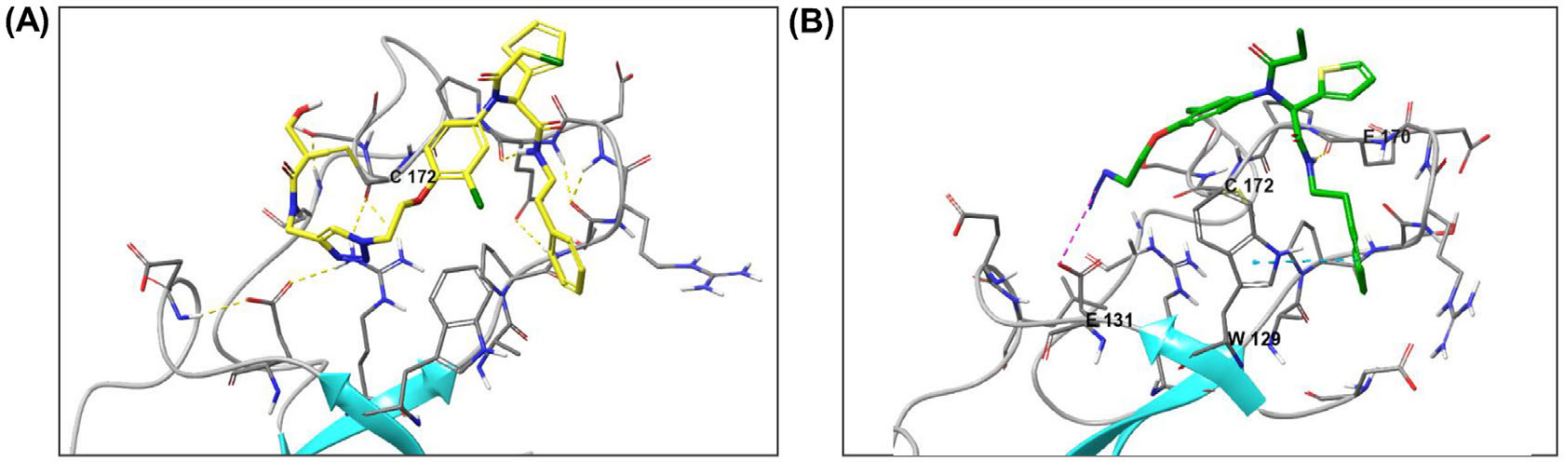
Molecular docking of **DK‑5070** and the representative epoxide‑containing degrader with UHRF1. (A) Covalent docking of **DK‑5084** (shown as a representative of the epoxide‑containing series, including **DK‑6075**) with UHRF1 (PDB: 6W92). (B) Non‑covalent docking of **DK‑5070** with UHRF1. All docking calculations were performed using the Glide algorithm followed by MM‑GBSA binding free energy estimation.

### 2.6 Degradation of GPX4 by DK‐5070 or DK‐6075 Triggers Ferroptosis

To determine whether the cytotoxic potency of **DK‐5070** and **DK‐6075** against HT1080 cells results from ferroptosis, we performed a CCK‐8 assay in the presence of 2 µM Ferrostatin‐1 (Fer‐1), a lipid peroxidation scavenger, using the GPX4 inhibitor ML162 as a control. Both **DK‐5070** and **DK‐6075** displayed antiproliferative activity superior to ML162 in HT1080 cells (IC_50_ = 47.21 and 298.4 nM vs. 515.3 nM), consistent with their respective degradation potencies (Figure 8A). Co‐treatment with Fer‐1 markedly attenuated the cytotoxicity of both **DK‐5070** and **DK‐6075**, indicating that ferroptosis is a major contributor to their cytotoxic effects. Given the essential role of glutathione (GSH) in GPX4 function, we measured intracellular GSH levels. Treatment with **DK‐5070** or **DK‐6075** resulted in a marked reduction in GSH levels (Figure 8B), and both compounds increased malondialdehyde (MDA) levels, a canonical marker of lipid peroxidation (Figure 8C). Furthermore, intracellular Fe^2^^+^ levels, a key driver of ferroptosis, were elevated upon treatment with **DK‐6075** or **DK‐5070**, exceeding the increase induced by ML162 (Figure 8D). Taken together, these results indicate that **DK‐5070** and **DK‐6075** suppress cell proliferation primarily through GPX4 degradation‐induced ferroptosis.

**FIGURE 8:**
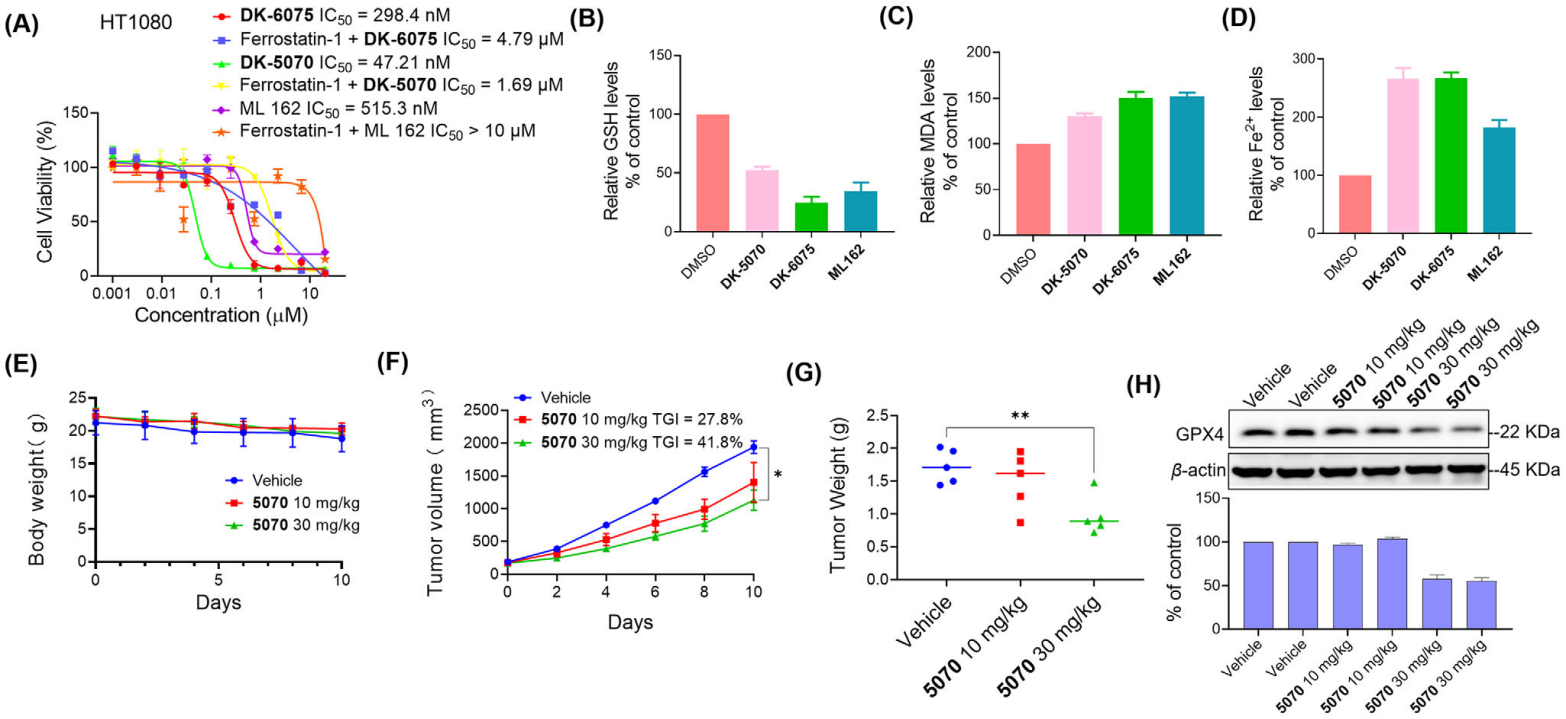
Degradation of GPX4 by **DK‐5070** or **DK‐6075** triggers ferroptosis. (A) Ferroptosis selectivity of **DK‐5070**, **DK‐6075,** and ML162. HT1080 cells were incubated with the tested compounds or in combination with 2 µM Fer‐1 for 72 h. (B–D) Determination of the content of intracellular GSH and MDA and Fe2^+^ after treatment with various concentrations of **DK‐5070** (50 nM, 24 h), **DK‐6075** (500 nM, 24 h), or ML162 (500 nM, 24 h). (E) Changes in the body weights of mice over the treatment period. (F) Tumor growth curves of HT1080 tumor‐bearing mice subjected to diverse treatments. (G) Comparison of tumor weight in mice from different groups. (H) Pharmacodynamic analysis of GPX4 protein in the HT1080 xenograft tumors in nude mice. Tumor tissues were harvested for Western blotting analysis.

### 2.7 Ferroptosis‐Driven Antitumor Efficacy of DK‐5070 in the HT1080 Xenograft Mouse Model

To evaluate the in vivo antitumor efficacy of **DK‐5070**, we subsequently employed HT1080 xenograft mouse models to evaluate the in vivo antitumor efficacy of **DK‐5070**. Tumor‐bearing mice were randomized into three groups (*n* = 5) with minimized intergroup differences in body weight and tumor volume. Treatment groups received daily intraperitoneal injections of **DK‐5070** (10 or 30 mg/kg). After 10 days of treatment, animals were humanely euthanized, and tumors were excised. As shown in Figure 8E,F, **DK‐5070** significantly suppressed tumor growth in a dose‐dependent manner, with tumor growth inhibition (TGI) values of 27.8% (10 mg/kg) and 41.8% (30 mg/kg), while causing no significant loss of body weight at any tested dose. Macroscopic tumor morphology and tumor weights further confirmed the superior antitumor activity of **DK‐5070** in vivo (Figure 8G). These results align with previous observations of **DK‐5070**’s enhanced cytotoxic potency in HT1080 cells. Western blot analysis of tumor tissue lysates confirmed GPX4 degradation under in vivo conditions (Figure 8H). To further validate that **DK‑5070** exerts its in vivo antitumor effect through GPX4 degradation‑induced ferroptosis, we measured ferroptosis‑related markers in tumor tissues from the xenograft model(Figure S5A–C). The results showed that, compared with the vehicle control group, the **DK‑5070** treatment group exhibited a decreasing trend in glutathione (GSH) levels, along with elevated levels of malondialdehyde (MDA) and ferrous iron (Fe^2^^+^) in tumor tissues, suggesting that lipid peroxidation and ferroptosis‑related events did occur to some extent in vivo. Collectively, these findings underscore the therapeutic potential of UHRF1‐recruiting targeted protein degradation and establish **DK‐5070** as a promising candidate for in vivo tumor suppression.

## 3 Conclusion

This study establishes UHRF1 as a new recruitable E3 ligase for targeted protein degradation (TPD) and demonstrates that a minimal azide moiety can serve as an effective small‑molecule recruitment ligand. By incorporating azide‑ and epoxide‑based electrophilic warheads into the ML162 scaffold, we developed the degraders **DK‑5070** and **DK‑6075**, which induce robust and durable GPX4 degradation with potent antitumor activity. Mechanistic and proteomic analyses revealed that UHRF1 recruitment drives GPX4 degradation, with the epoxide warhead of **DK‑6075** covalently engaging UHRF1 at Cys170 and further stabilizing the degradation process. GPX4 depletion subsequently triggers ferroptosis, which in turn contributes to the potent antitumor effects of these degraders. Compared with large‑size PROTAC degraders, these small‑molecule degraders exhibited superior degradation activity and anticancer effects. Although **DK‑5070** achieved a tumor growth inhibition rate (TGI) of 41.8% in the HT1080 xenograft model and GPX4 degradation was detectable in tumor tissues, its antitumor efficacy remained relatively moderate. We speculate that the modest efficacy may be primarily due to suboptimal pharmacokinetic properties of **DK‑5070**, leading to insufficient systemic exposure and tumor distribution. Of note, the azide moiety itself is considered metabolically stable in medicinal chemistry; however, other structural features, such as the ester linkage, may contribute to rapid systemic clearance. Our future optimization efforts will focus on improving metabolic stability through rational structural modifications while preserving the UHRF1‑recruiting activity, as well as exploring improved dosing regimens or formulations to enhance tumor exposure. Collectively, these findings establish minimal‑azide recruitment chemistry as a viable strategy for engaging UHRF1 and broaden the ligandable E3 ligase landscape of TPD.

## 4 Experimental Section

### 4.1 Statistical Analysis

All data are presented as mean ± SD from at least three independent experiments unless otherwise stated. For Western blot, protein band intensities were quantified using ImageJ and normalized to the corresponding loading control (Actin). Statistical comparisons between two groups were performed using unpaired two‐tailed Student's t‐test. A p‐value of less than 0.05 was considered statistically significant (n.s., not significant). All statistical analyses were conducted using GraphPad Prism 9.0 (GraphPad Software, San Diego, CA, USA). For in vivo studies, *n* = 5 mice per group, and data are presented as mean ± SEM.

## Author Contributions

Z.L.: methodology and data curation; K.D.: data curation; R.W.: methodology, data curation, validation, investigation; T.J.: data curation, Writing – review & editing; J.W.: methodology, data curation, validation, investigation; M.S.: methodology, data curation, investigation, validation, visualization; M.A.S.: methodology, data curation; M.A.G.: methodology, data curation; Z‐M.Z.: methodology, data curation; Z.Z.: conceptualization; K.D.: conceptualization, Writing – review & editing; N.M.: conceptualization, writing – review & editing, writing – original draft; T.L.: conceptualization, supervision, formal analysis, Writing – review & editing; Y.T.: supervision, validation, Writing – review & editing; Z.L.: Writing – review & editing, resources, project administration, supervision, writing – original draft, funding acquisition. All authors have read and approved the submitted version of the manuscript.

## Ethical Statement

All animal experiments in this study were conducted in accordance with the ethical standards of the institution and/or national research council. Research involving animals was conducted under the ethical supervision of all participating centers and in accordance with applicable regulations and good practice principles. The research protocol has been reviewed and approved by the Ethics Review Committee of South China University of Technology (Approval No.: AE‐2025101).

## Conflicts of Interest

The authors declare no conflicts of interest.

## Supporting information




**Supporting File 1**: advs77201‐sup‐0001‐SuppMat.pdf.

**Supporting File 2**: advs77201‐sup‐0002‐TableS1–S5.xlsx.

## Data Availability

The data that support the findings of this study are available on request from the corresponding author. The data are not publicly available due to privacy or ethical restrictions.
