# Supplementary material for: A New Recruitable E3 Ligase UHRF1 Supporting Targeted Protein Degradation: A Minimal Azide as a Recruitment Ligand
Source: Adv Sci (Weinh). 2026 Aug 19;13(62):e77201. doi: 10.1002/advs.77201 (PMC13488481; doi:10.1002/advs.77201)

A New Recruitable E3 Ligase UHRF1 Supporting Targeted Protein Degradation: A Minimal Azide as a Recruitment Ligand

Zehong Lin,[1,2,6] Kang Duan,[1,2,6] Rui Wan,[1,2,6] Tao Jiang,[1,2,6] Jinzhe Wei,[1,2] Miao Sun,[1,2] Mariia A. Skryl'nikova,[4] Maxim A. Gureev,[5] Zhi-Min Zhang,[1,2] Zhang Zhang,[1,2] Ke Ding,*[1,2] Nan Ma,*[1,2] Tongzheng Liu,*[1,2] Yi Tan,*[1,2] Zhengqiu Li*[1,2,3]

[1]State Key Laboratory of Bioactive Molecules and Druggability Assessment, Jinan University, 601 Huangpu Avenue West, Guangzhou, 510632 China

[2]Guangdong Basic Research Center of Excellence for Natural Bioactive Molecules and Discovery of Innovative Drugs, Jinan University, 601 Huangpu Avenue West, Guangzhou, 510632 China

[3]MOE Key Laboratory of Tumor Molecular Biology, Jinan University, 601 Huangpu Avenue West, Guangzhou, 510632 China

[4]Department of chemistry and technology of organic nitrogen compounds, Saint-Petersburg State Institute of Technology, 24-26/49 A, Moskovski ave., St. Petersburg, Russia, 190013

[5]Laboratory of bio- and chemoinformatics, HSE University, Soyuza Pechatnikov str., 16, Saint-Petersburg, Russia, 190121

[6]These authors contributed equally.

Email: pharmlzq@jnu.edu.cn (Li, Z.), tanyi@jnu.edu.cn (Tan, Y.), liutongzheng@jnu.edu.cn (Liu, T.), nanma927@126.com (Ma, N.), dingk@sioc.ac.cn (Ding, K.)

Table of contents

1. General Methods 2
2. Reagents 2
3.Cell lines 2
4. Structure of compounds 3
5. Synthetic procedures of probes[1] 4
6. Cell viability assay 15
7. Cell lysis and western blot 16
8. Immunoprecipitations 16
9. Target validation by pulldown/western blot 16
10. Co-IP/LC-MS experiment 17
11. LC-MS/MS analysis 17
12. Database Search 17
13. Modification site analysis of recombinant UHRF1 protein with DK-7062 18
14. Determination of GSH 18
15. Detection of GPX4-DK5070-UHRF1 ternary complex by surface plasmon resonance (SPR) .. 18
16. Determination of malondialdehyde (MDA) 18
17. Determination of iron (Fe^{2+}) intensity 19
18. Animals and animal model 19
19. Molecular docking 19
20. NMR Spectrum 23
20. Supplementary References 49
21. Source data 50

1. General Methods

All reactions were carried out under an atmosphere of argon in oven-dried glassware with a magnetic stirring bar. All reactions were monitored by thin layer chromatography (TLC), which were visualized with ultraviolet light and then developed with basic potassium permanganate solution. Flash column chromatography was carried out using silica gel (HAIYANG, 200-300 mesh). All NMR spectra (^{1}H-NMR, ^{13}C-NMR) were recorded on BrukerAvance ARX- 400. Chemical shifts were reported in parts per million (ppm) referenced with respect to appropriate internal standards or residual solvent peaks ($CDCl_3$ = 7.26 ppm, DMSO-d6 = 2.50 ppm). The following abbreviations were used in reporting spectra, br s (broad singlet), s (singlet), d (doublet), t (triplet), q (quartet), m (multiplet), dd (doublet of doublets). Mass spectra were obtained on Agilent LC-ESI-MS system.

2. Reagents

BCA protein detection kit (Beyotime, P0009). The anti-GPX4 (ab125066, dilution 1:1,000) was purchased from abcam company. The anti-Actin (AA128-1, dilution 1:5,000), anti-GAPDH (AG019-1, dilution 1:3,000) were purchased from Beyotime. The anti-UHRF1(84489-5-RR, dilution 1:1,000), HRP-linked rabbit IgG (7074, dilution 1:5,000) and HRP-linked mouse IgG (7076, dilution 1:5,000) antibodies were purchased from Cell Signaling Technology. The anti-FLAG (F1804, dilution 1:5,000), anti-HA (H3663, dilution 1:5,000) were purchased from Sigma-Aldrich. MG132 (S2619), Bortezomib (S1013), MLN4924 (S7109) were purchased from Selleck Chemicals. The Recombinant human UHRF1 protein (P07941) was purchased from Beijing Solarbio Science. Total Glutathione Assay Kit (Beyotime Biotechnology, S0053), Lipid Peroxidation MDA Assay Kit (Beyotime Biotechnology, S0131S), Cell Ferrous Iron (Fe^{2+}) Fluorometric Assay Kit (E-BC-F101, Elabscience Biotechnology Co., Ltd.) were commercially available.

3.Cell lines

HEK-293T cells was obtained from the American Type Culture Collection (ATCC). HT-1080 cells were obtained from the Cell Bank of the Chinese Academy of Sciences. HEK-293T and HT-1080 cells were cultured in DMEM (Gibco, 11995500), which contains 10% heat-inactivated fetal bovine serum (FBS; Invitrogen), 100 units/mL penicillin, and 100 µg/mL Streptomycin and stored in a 37°C humidified incubator with 5% carbon dioxide.

4. Structure of compounds

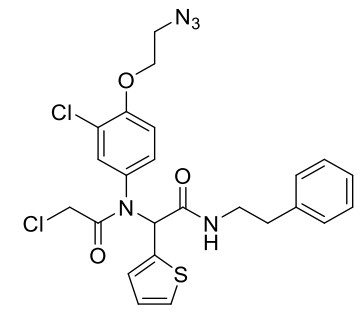

5070

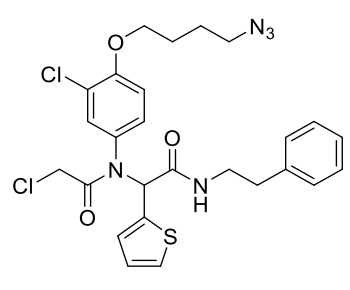

6008

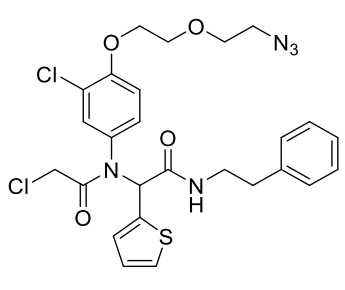

6055

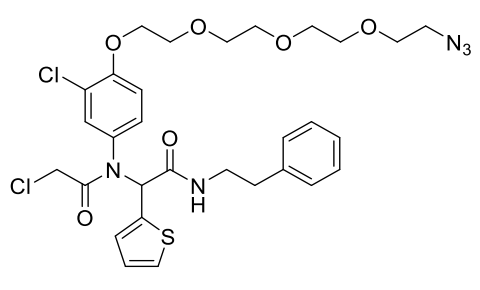

6056

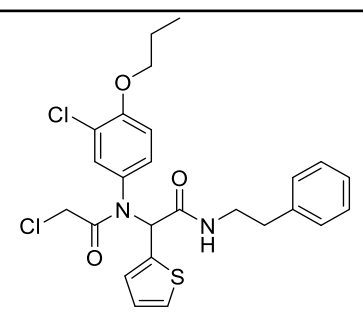

6106

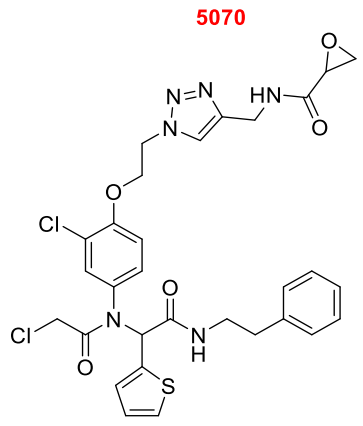

5084

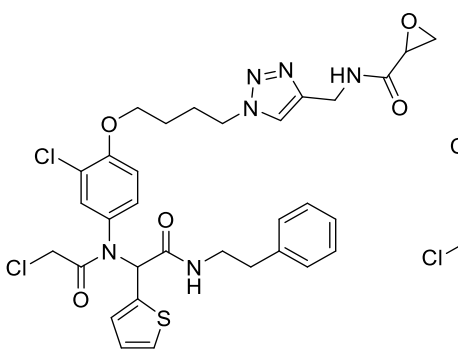

6075

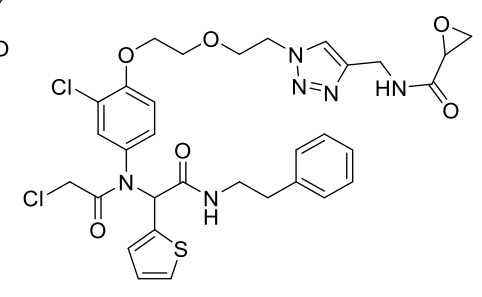

6060

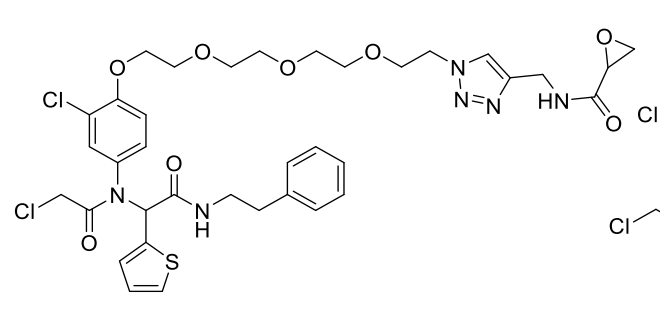

6061

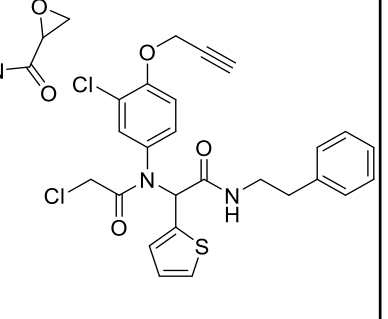

6035

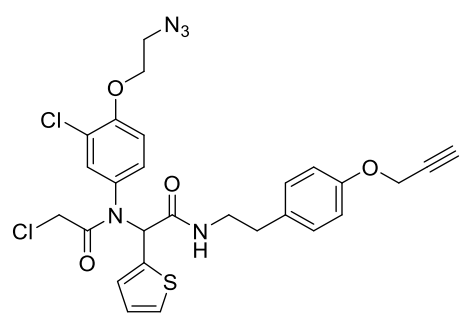

6135

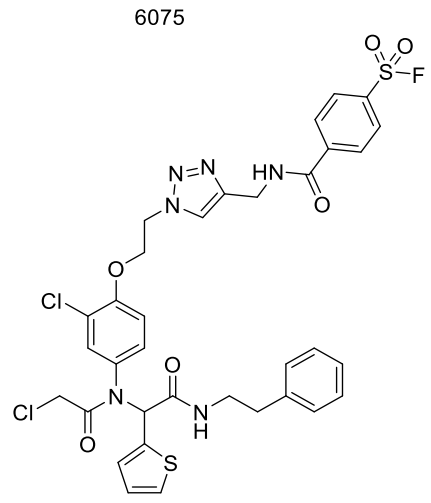

5071

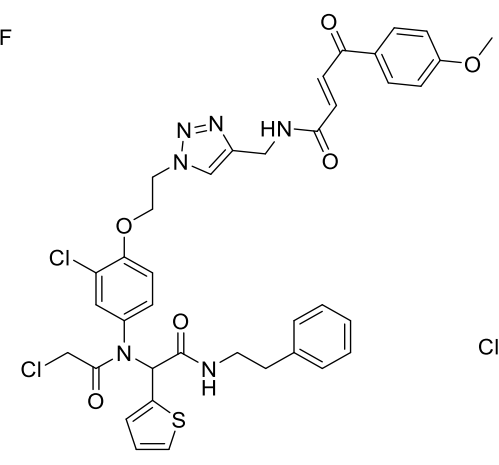

5072

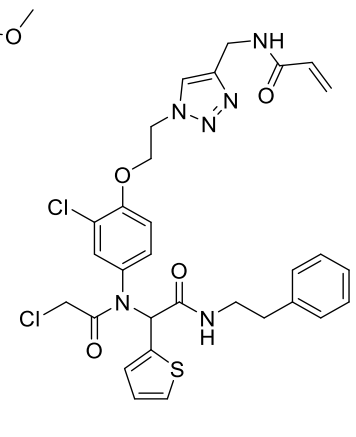

6076

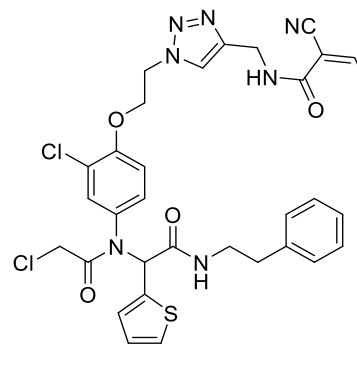

6101

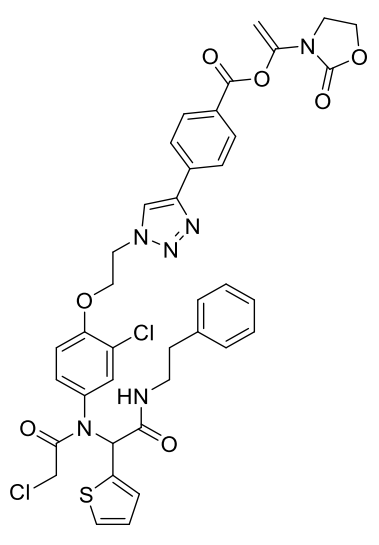

5075

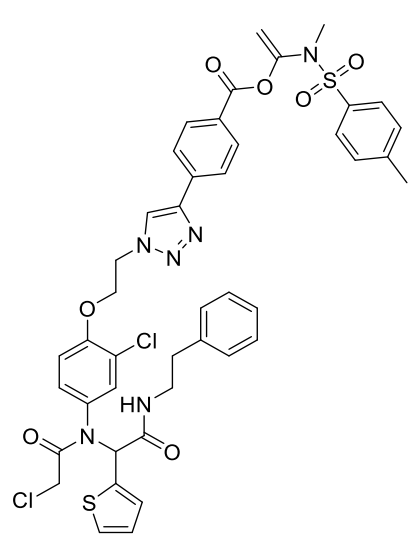

6090

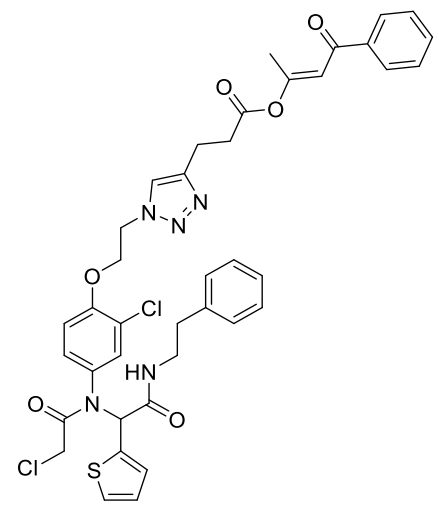

5073

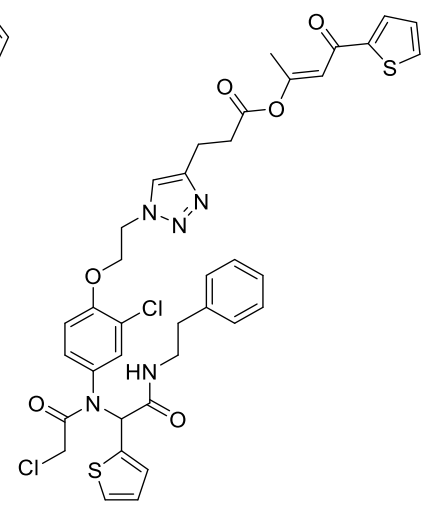

5085

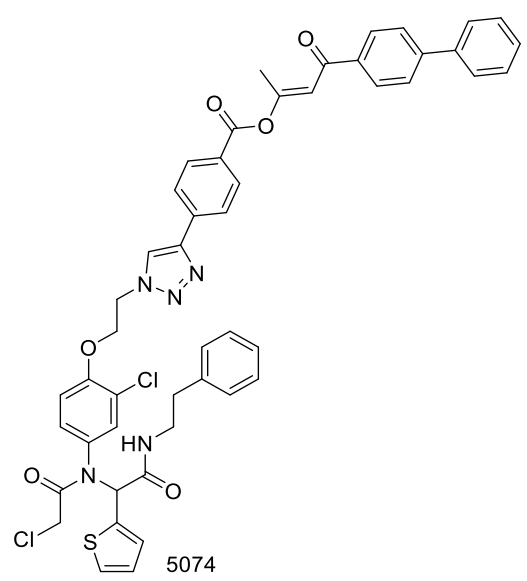

5074

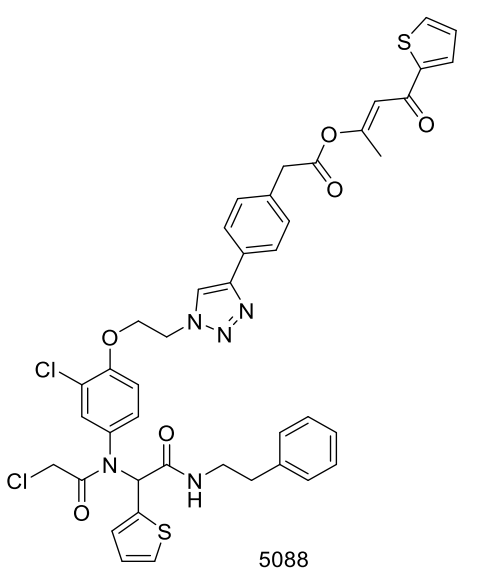

5088

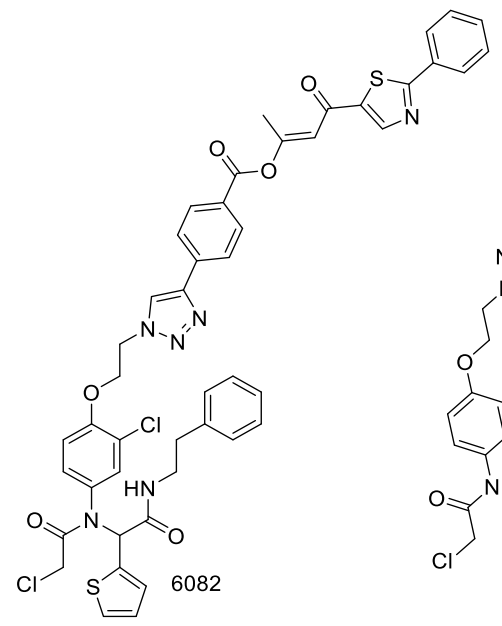

6082

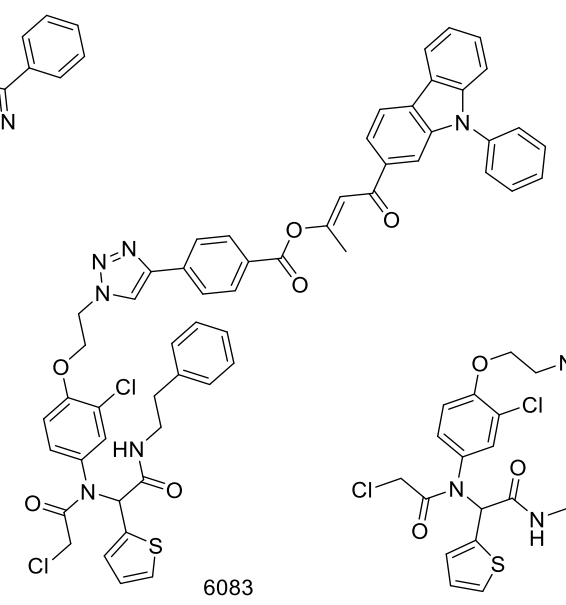

6083

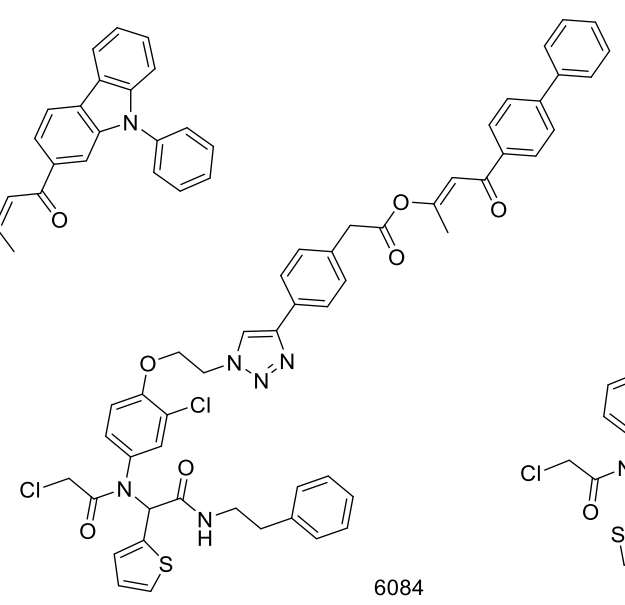

6084

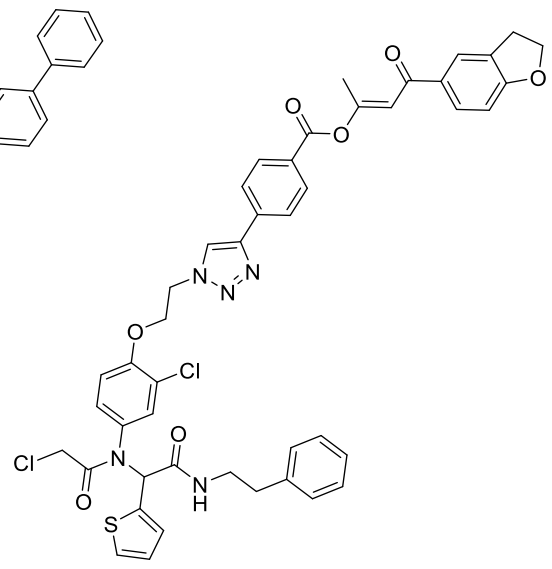

6092

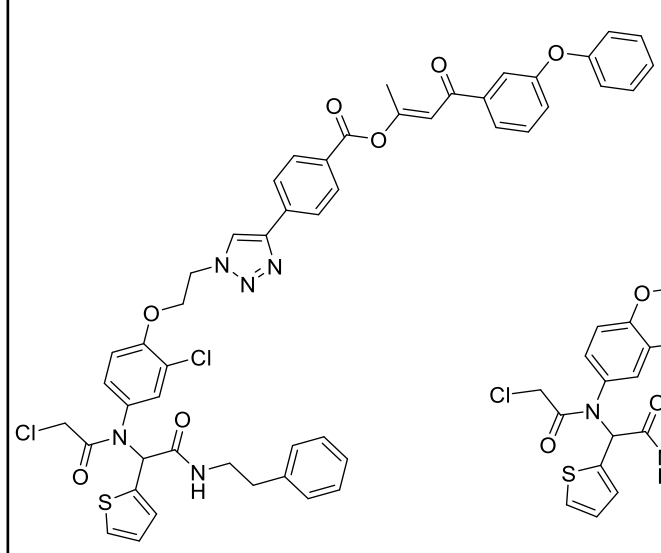

6093

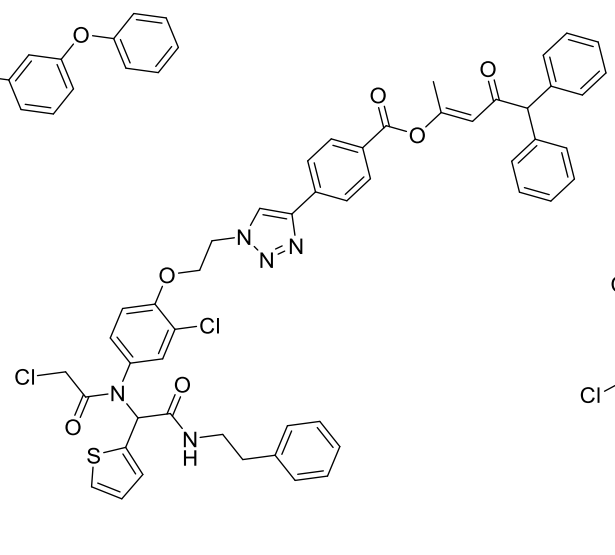

6094

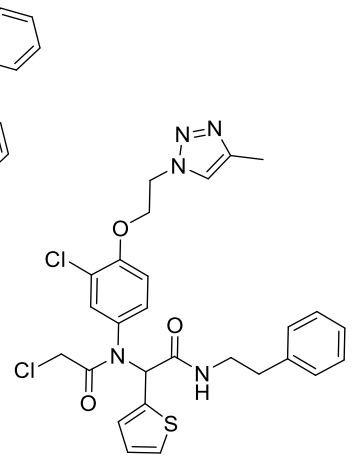

6100

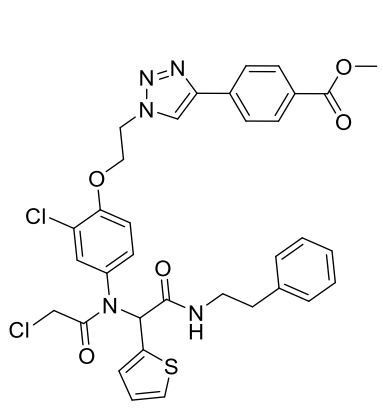

6104

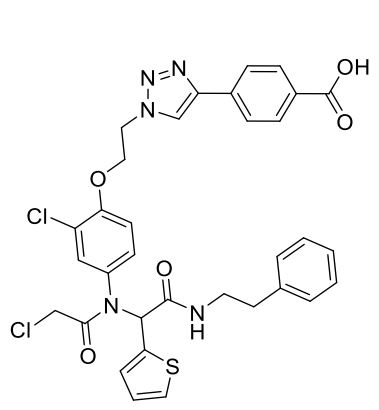

6105

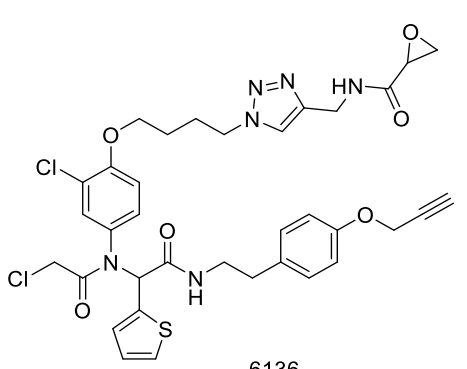

6136

5. Synthetic procedures of probes[1]

General Procedure for Ugi reaction: A solution of S1-3 (0.6 mmol, 127.5 mg) and 2-formylthiophene (0.6 mmol, 67.2 mg) was stirred at rt in 1 mL MeOH for 15 min. 2-chloroacetic acid (0.5 mmol, 47.2 mg) and 2-phenethylisocyanide (0.5 mmol, 65.6 mg) were then added. The reaction was stirred at rt for 24 h and subsequently concentrated *in vacuo*. The crude product was purified by flash chromatography eluting with ethyl acetate/petroleum ether (1:2) to get the desired product.

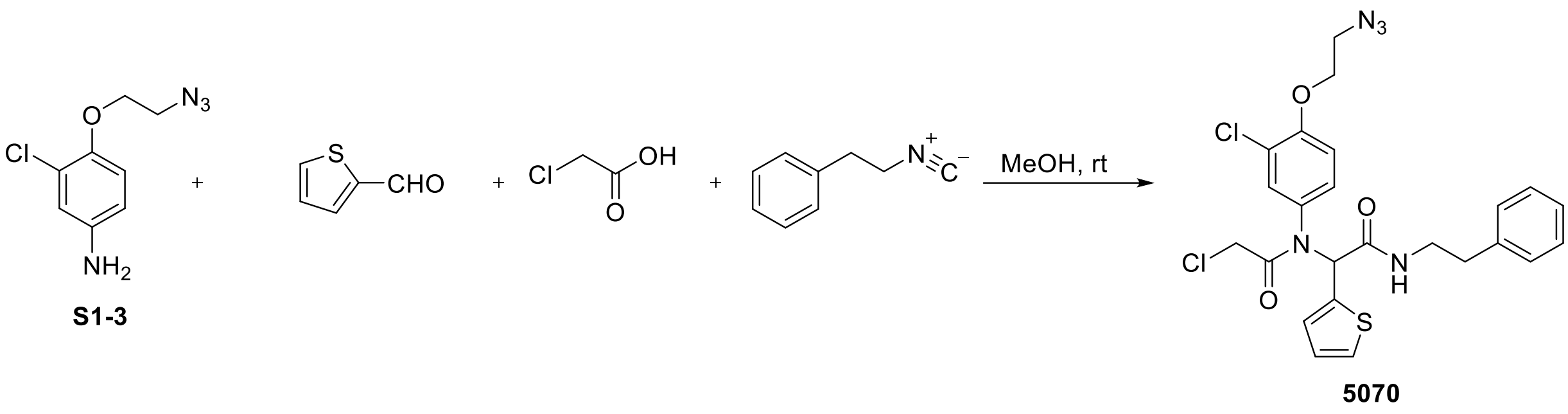

N-(4-(2-azidoethoxy)-3-chlorophenyl)-2-chloro-*N*-(2-oxo-2-(phenethylamino)-1-(thiophen-2-yl)ethyl) acetamide **(5070):** Light yellow oil, 188.4 mg, 70.8% yield. ^{1}H NMR (600 MHz, $CDCl_3$) δ 7.29 – 7.23 (m, 3H), 7.23 – 7.16 (m, 1H), 7.16 – 7.06 (m, 2H), 6.94 – 6.61 (m, 4H), 6.07 (m, 2H), 4.16 (m, 2H), 3.81 (d, J = 3.5 Hz, 2H), 3.65 (t, J = 4.9 Hz, 2H), 3.56 (m, 2H), 2.81 (m, 2H). ^{13}C NMR (151 MHz, $CDCl_3$) δ 167.9, 166.7, 154.4, 138.6, 134.7, 131.7, 130.1, 129.4, 128.8, 128.6, 128.3, 126.6, 126.5, 112.8, 68.1, 50.1, 42.3, 41.1, 35.5. ESI-MS calcd for $C_{24}H_{23}Cl_2N_5NaO_3S$ $[M+Na]^+$ m/z = 554.0796; Found 554.0792.

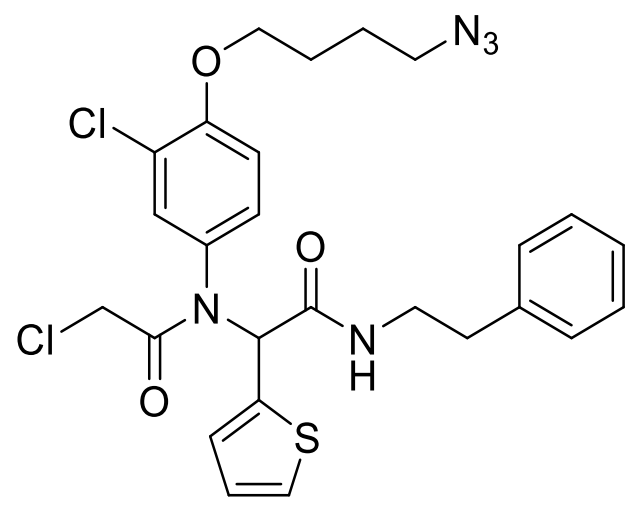

6008

N-(4-(4-azidobutoxy)-3-chlorophenyl)-2-chloro-*N*-(2-oxo-2-(phenethylamino)-1-(thiophen-2-yl)ethyl) acetamide **(6008):** Light yellow oil, 156.9 mg, 56.0% yield. ^{1}H NMR (400 MHz, $CDCl_3$) δ 7.45 – 7.05 (m, 7H), 6.92 – 6.73 (m, 3H), 6.09 (m, 2H), 4.03 (t, J = 6.0 Hz, 2H), 3.81 (s, 2H), 3.56 (m, 2H), 3.40 (t, J = 6.6 Hz, 2H), 2.82 (m, 2H), 1.92 (m, 2H), 1.84 (m, 2H). ^{13}C NMR (151 MHz, $CDCl_3$) δ 168.0, 166.7, 154.9, 138.6, 134.8, 131.5, 130.1, 129.3, 128.9, 128.6, 128.3, 126.6, 126.5, 112.7, 68.5, 51.1, 42.4, 41.1, 35.5, 26.3, 25.7. ESI-MS calcd for $C_{26}H_{28}Cl_2N_5O_3S$ $[M+H]^+$ m/z = 560.1290; Found 560.1284.

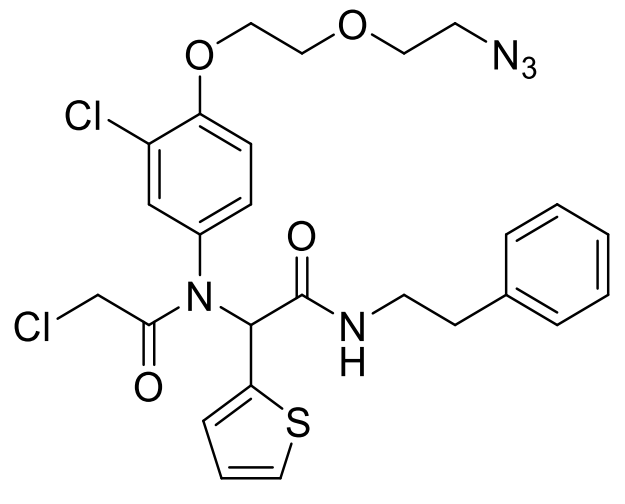

6055

N-(4-(2-(2-azidoethoxy)ethoxy)-3-chlorophenyl)-2-chloro-*N*-(2-oxo-2-(phenethylamino)-1-(thiophen-2-yl)ethyl)acetamide **(6055):** Light yellow oil, 184.5 mg, 64.0% yield. ^{1}H NMR (400 MHz, $CDCl_3$) δ 7.31 – 7.07 (m, 7H), 6.91 – 6.74 (m, 3H), 6.05 (m, 2H), 4.17 (t, *J* = 4.7 Hz, 2H), 3.91 (t, *J* = 4.7 Hz, 2H), 3.83 – 3.70 (m, 4H), 3.55 (m, 2H), 3.41 (t, *J* = 5.0 Hz, 2H), 2.90 – 2.69 (m, 2H). ^{13}C NMR (151 MHz, $CDCl_3$) δ 167.9, 166.7, 154.8, 138.6, 134.7, 131.5, 130.1, 128.8, 128.6, 128.3, 126.6, 126.5, 113.1, 70.6, 69.4, 69.1, 50.8, 42.3, 41.1, 35.5. ESI-MS calcd for $C_{26}H_{27}Cl_2N_5NaO_4S$ $[M+Na]^+$ m/z = 598.1059; Found 598.1052.

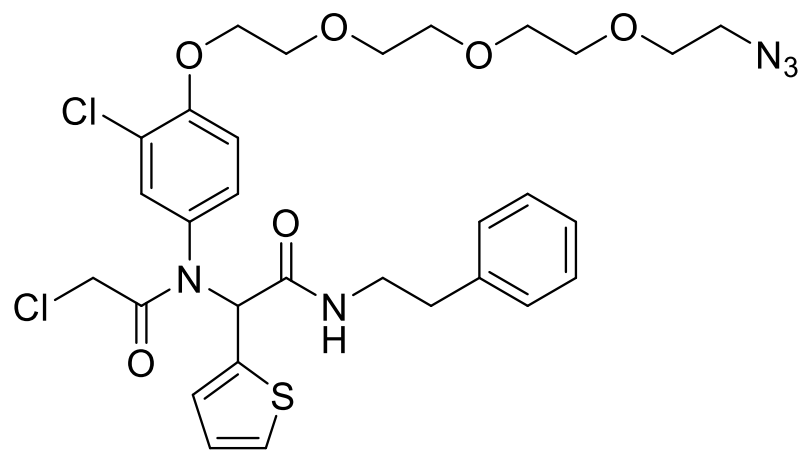

6056

N-(4-(2-(2-(2-(2-azidoethoxy)ethoxy)ethoxy)ethoxy)-3-chlorophenyl)-2-chloro-*N*-(2-oxo-2-(phenethyl amino)-1-(thiophen-2-yl)ethyl)acetamide **(6056):** Light yellow oil, 229.6 mg, 69.1% yield. ^{1}H NMR (400 MHz, $CDCl_3$) δ 7.38 – 7.02 (m, 7H), 6.93 – 6.54 (m, 4H), 6.06 (m, 2H), 4.15 (d, *J* = 5.0 Hz, 2H), 3.91 (t, *J* = 4.3 Hz, 2H), 3.85 – 3.73 (m, 4H), 3.68 (m, 8H), 3.56 (m, 2H), 3.38 (m, 2H), 2.81 (m, 2H). ^{13}C NMR (151 MHz, $CDCl_3$) δ 168.0, 166.7, 154.9, 138.6, 134.7, 131.5, 130.1, 129.2, 128.8, 128.6, 128.3, 126.6, 126.5, 113.0, 71.1, 70.8, 70.7, 70.1, 69.3, 69.0, 50.7, 42.3, 41.1, 35.5. ESI-MS calcd for $C_{30}H_{36}Cl_2N_5O_6S$ $[M+H]^+$ m/z = 664.1763; Found 664.1757.

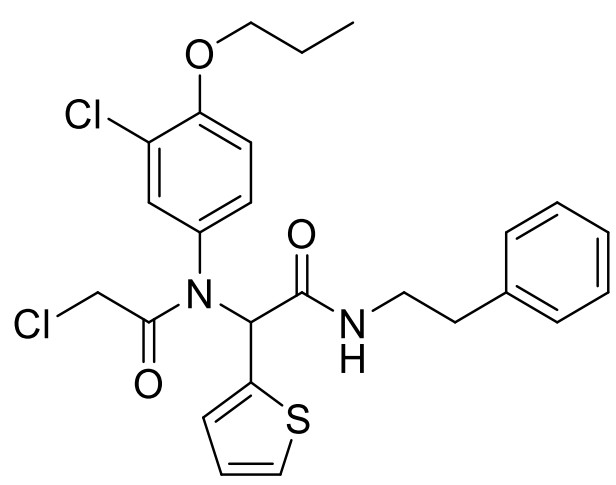

6106

2-chloro-*N*-(3-chloro-4-propoxyphenyl)-*N*-(2-oxo-2-(phenethylamino)-1-(thiophen-2-yl)ethyl) acetamide **(6106):** White solid, 116.2 mg, 46.0% yield. ^{1}H NMR (400 MHz, $CDCl_3$) δ 7.32 – 7.04 (m, 7H), 6.96 – 6.57 (m, 4H), 6.06 (m, 2H), 3.96 (t, *J* = 6.5 Hz, 2H), 3.82 (d, *J* = 2.3 Hz, 2H), 3.68 – 3.44 (m, *J* = 6.8 Hz, 2H), 2.93 – 2.73 (m, 2H), 1.86 (m, 2H), 1.07 (t, *J* = 7.4 Hz, 3H). ^{13}C NMR (151 MHz, $CDCl_3$) δ 168.0, 166.7, 155.1, 138.6, 134.8, 131.3, 130.9, 130.1, 129.2, 128.8, 128.6, 128.3, 126.5, 123.1, 112.7, 70.7, 42.4, 41.1, 35.5, 22.4, 10.5. LC-MS calcd for $C_{25}H_{27}Cl_2N_2O_3S$ $[M+H]^+$ m/z = 505.1; Found 505.1.

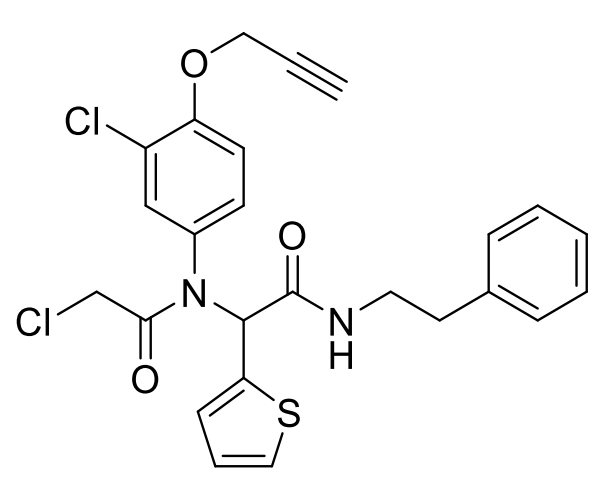

6035

2-chloro-*N*-(3-chloro-4-(prop-2-yn-1-yloxy)phenyl)-*N*-(2-oxo-2-(phenethylamino)-1-(thiophen-2-yl) ethyl) acetamide **(6035)**: White solid, 79.3 mg, 79.1% yield. ^{1}H NMR (400 MHz, $CDCl_3$) δ 7.30 – 7.18 (m, 5H), 7.17 – 7.10 (m, 2H), 7.04 – 6.88 (m, 2H), 6.88 – 6.80 (m, 2H), 6.07 (m, 2H), 4.76 (d, *J* = 2.4 Hz, 2H),

3.82 (d, J = 1.6 Hz, 2H), 3.56 (dp, J = 15.8, 6.7 Hz, 2H), 2.93 – 2.70 (m, 2H), 2.57 (t, J = 2.4 Hz, 1H). LC-MS calcd for $C_{25}H_{23}Cl_2N_2O_3S$ $[M+H]^+$ m/z = 501.1; Found 501.1.

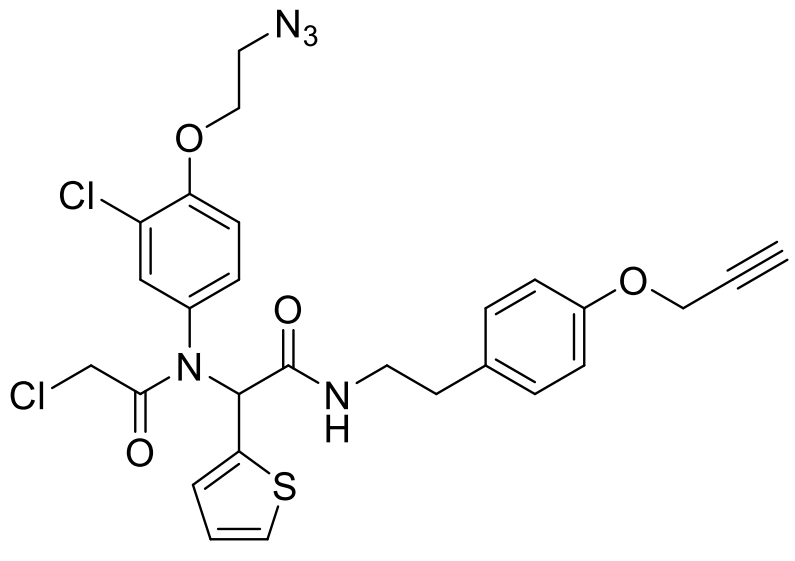

6135

N-(4-(2-azidoethoxy)-3-chlorophenyl)-2-chloro-*N*-(2-oxo-2-((4-(prop-2-yn-1-yloxy)phenethyl) amino)-1- (thiophen-2-yl)ethyl)acetamide **(6135)**: White solid, 79.3 mg, 79.1% yield. ^{1}H NMR (400 MHz, $CDCl_3$) δ 7.25 (d, J = 4.5 Hz, 2H), 7.11 – 7.01 (m, 2H), 6.97 – 6.59 (m, 6H), 6.04 (d, J = 37.6 Hz, 2H), 4.65 (d, J = 2.5 Hz, 2H), 4.16 (t, J = 4.9 Hz, 2H), 3.82 (d, J = 1.0 Hz, 2H), 3.65 (t, J = 4.9 Hz, 2H), 3.53 (m, 2H), 2.86 – 2.65 (m, 2H), 2.49 (t, J = 2.4 Hz, 1H). ^{13}C NMR (151 MHz, $CDCl_3$) δ 167.9, 166.7, 156.3, 154.4, 131.7, 131.6, 130.2, 129.9, 129.3, 128.4, 126.6, 115.0, 112.9, 78.7, 75.5, 68.1, 55.8, 50.1, 42.3, 41.1, 34.6. LC-MS calcd for $C_{27}H_{26}Cl_2N_5O_4S$ $[M+H]^+$ m/z = 586.1; Found 586.1.

General Procedure for click reaction: A solution of sodium ascorbate (0.6 mmol, 118.9 mg) and Copper(II) sulfate (0.2 mmol, 31.9 mg) was stirred at rt in THF/H_2O (10:1, 8 mL) for 15 min. 5070 (0.2 mmol, 106.5 mg) and alkyne (0.2 mmol, 8.0 mg) were then added. The reaction was stirred at rt for 4 h and extracted with ethyl acetate (3 ×5 mL). Organic layers were combined, dried with Na_2SO_4 and evaporated under reduced pressure. The crude product was purified by flash chromatography eluting with MeOH/DCM (1:50) to get the desired product.

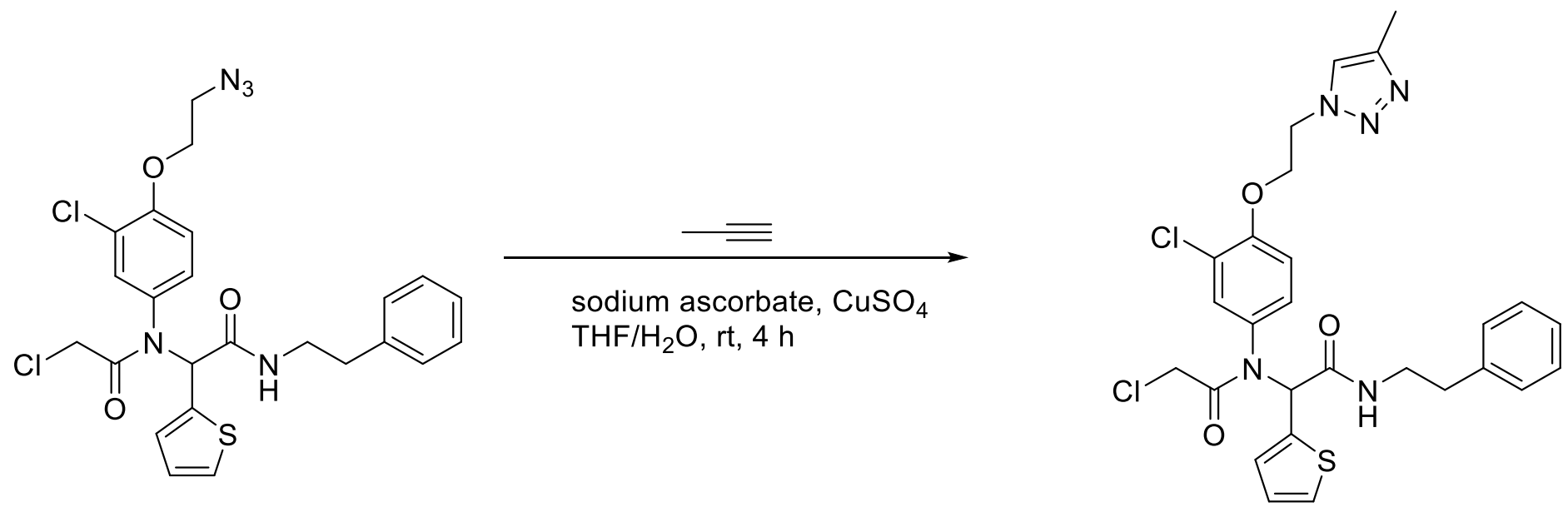

5070 **6100**

2-chloro-*N*-(3-chloro-4-(2-(4-methyl-1*H*-1,2,3-triazol-1-yl)ethoxy)phenyl)-*N*-(2-oxo-2-(phenethylamino) -1-(thiophen-2-yl)ethyl)acetamide **(6100):** White solid, 36.7 mg, 32.1% yield. ^{1}H NMR (400 MHz, $CDCl_3$) δ 7.68 (s, 1H), 7.35 – 7.01 (m, 7H), 6.93 – 6.44 (m, 4H), 6.08 (m, 2H), 4.79 (d, J = 4.9 Hz, 2H), 4.34 (d, J = 5.1 Hz, 2H), 3.78 (s, 2H), 3.56 (m, 2H), 2.91 – 2.69 (m, 2H), 2.35 (s, 3H). ^{13}C NMR (151 MHz, $CDCl_3$) δ 167.9, 166.6, 153.9, 138.6, 134.7, 132.1, 131.8, 130.1, 129.6, 128.8, 128.6, 128.3, 126.6, 126.5, 112.6, 98.4, 67.6, 60.6, 42.3, 41.1, 35.5, 29.7, 10.8. LC-MS calcd for $C_{27}H_{28}Cl_2N_5O_3S$ $[M+H]^+$ m/z = 572.1; Found 572.1.

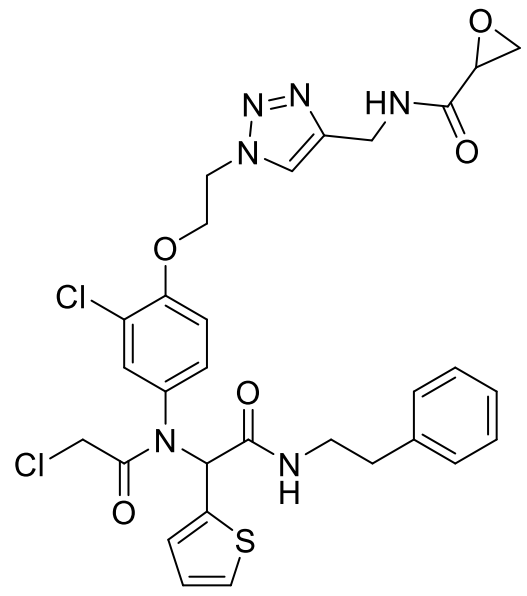

5084

N-((1-(2-(2-chloro-4-(2-chloro-*N*-(2-oxo-2-(phenethylamino)-1-(thiophen-2-yl)ethyl)acetamido) phenoxy)ethyl)-1*H*-1,2,3-triazol-4-yl)methyl)oxirane-2-carboxamide **(5084):** White solid, 109.2 mg, 83.1% yield. ^{1}H NMR (400 MHz, $CDCl_3$) δ 7.85 (s, 1H), 7.26 – 7.07 (m, 6H), 7.00 – 6.44 (m, 5H), 6.06 (m, 2H), 4.80 (t, J = 4.8 Hz, 2H), 4.63 – 4.44 (m, 2H), 4.34 (t, J = 4.9 Hz, 2H), 3.78 (s, 2H), 3.55 (m, 2H), 3.44 (m, 1H), 2.97 (m, 1H), 2.91 – 2.70 (m, 3H). ESI-MS calcd for $C_{30}H_{30}Cl_2N_6NaO_5S$ $[M+Na]^+$ m/z = 679.1273;

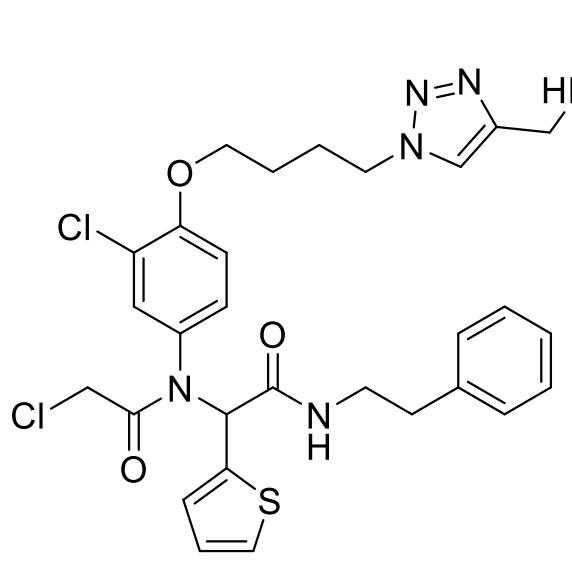

6075

N-((1-(4-(2-chloro-4-(2-chloro-*N*-(2-oxo-2-(phenethylamino)-1-(thiophen-2-yl)ethyl)acetamido) phenoxy)butyl)-1*H*-1,2,3-triazol-4-yl)methyl)oxirane-2-carboxamide **(6075)**: White solid, 99.4 mg, 72.5% yield. ^{1}H NMR (400 MHz, $CDCl_3$) δ 7.60 (d, J = 2.8 Hz, 1H), 7.31 – 7.04 (m, 7H), 7.01 – 6.59 (m, 5H), 6.15 (m, 2H), 4.49 (m, 4H), 4.03 (d, J = 7.4 Hz, 2H), 3.82 (s, 2H), 3.56 (m, 2H), 3.44 (d, J = 4.0 Hz, 1H), 2.97 (q, J = 4.5 Hz, 1H), 2.91 – 2.71 (m, 3H), 2.15 (m, 2H), 1.87 (d, J = 8.3 Hz, 3H). ^{13}C NMR (151 MHz, $CDCl_3$) δ 168.8, 168.1, 168.0, 166.7, 154.7, 144.2, 138.7, 134.8, 131.6, 131.3, 130.1, 129.5, 128.8, 128.6, 128.3, 126.6, 126.5, 122.4, 112.6, 68.3, 60.6, 49.9, 49.5, 47.4, 42.5, 42.4, 41.1, 35.5, 34.3, 27.2, 25.8. ESI-MS calcd for $C_{32}H_{34}Cl_2N_6NaO_5S$ $[M+Na]^+$ m/z = 707.1586; Found 707.1581.

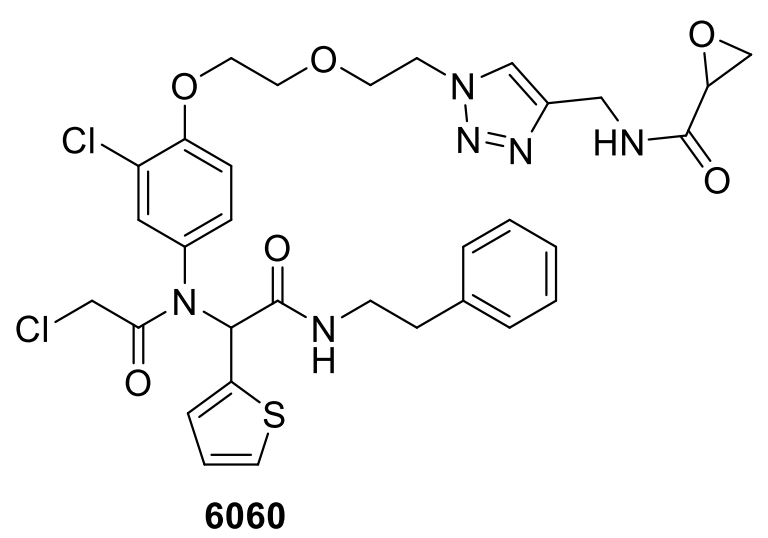

6060

N-((1-(2-(2-(2-chloro-4-(2-chloro-*N*-(2-oxo-2-(phenethylamino)-1-(thiophen-2-yl)ethyl)acetamido) phenoxy)ethoxy)ethyl)-1*H*-1,2,3-triazol-4-yl)methyl)oxirane-2-carboxamide **(6060):** White solid, 89.9 mg, 64.1% yield. ^{1}H NMR (400 MHz, $CDCl_3$) δ 7.71 (d, J = 3.0 Hz, 1H), 7.30 – 7.02 (m, 7H), 6.95 – 6.61 (m, 5H), 6.11 (m, 2H), 4.65 – 4.42 (m, 4H), 4.12 (d, J = 5.0 Hz, 2H), 3.98 (m, 2H), 3.91 – 3.77 (m, 4H), 3.56 (t,

J = 7.4 Hz, 2H), 3.43 (d, J = 4.0 Hz, 1H), 2.96 (m, 1H), 2.92 – 2.71 (m, 3H). ^{13}C NMR (151 MHz, $CDCl_3$) δ 168.7, 168.0, 166.7, 154.6, 143.9, 138.6, 134.8, 131.6, 130.1, 129.4, 128.8, 128.6, 128.3, 126.6, 126.5, 123.3, 112.9, 69.8, 69.3, 68.8, 50.4, 49.5, 47.4, 42.3, 41.1, 35.5, 34.3. ESI-MS calcd for $C_{32}H_{35}Cl_2N_6O_6S$ $[M+H]^+$ m/z = 701.1716; Found 701.1713.

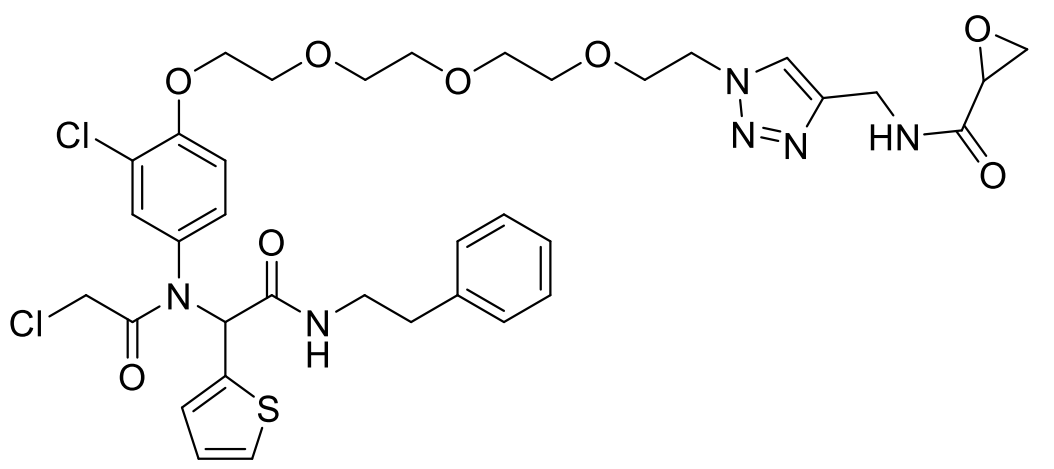

6061

N-((1-(2-(2-(2-(2-(2-chloro-4-(2-chloro-*N*-(2-oxo-2-(phenethylamino)-1-(thiophen-2-yl)ethyl)acetamido) phenoxy)ethoxy)ethoxy)ethoxy)ethyl)-1*H*-1,2,3-triazol-4-yl)methyl)oxirane-2-carboxamide **(6061)**: White solid, 90.4 mg, 57.3% yield. ^{1}H NMR (400 MHz, $CDCl_3$) δ 7.72 (t, J = 2.2 Hz, 1H), 7.33 – 7.07 (m, 7H), 6.98 – 6.46 (m, 5H), 6.09 (m, 2H), 4.51 (m, 4H), 4.15 (s, 2H), 3.94 – 3.71 (m, 8H), 3.71 – 3.48 (m, 7H), 3.43 (m, 1H), 2.96 (m, 1H), 2.88 – 2.69 (m, 3H). ^{13}C NMR (151 MHz, $CDCl_3$) δ 170.4, 168.7, 163.4, 154.9, 151.4, 138.0, 131.4, 130.2, 130.1, 128.8, 128.7, 128.6, 127.6, 126.9, 126.8, 126.7, 126.6, 123.6, 123.4, 120.6, 120.5, 117.1, 114.0, 71.8, 71.1, 71.0, 70.6, 70.5, 69.5, 69.3, 63.0, 55.0, 50.3, 47.5, 41.0, 35.2, 34.7. ESI-MS calcd for $C_{36}H_{43}Cl_2N_6O_8S$ $[M+H]^+$ m/z = 789.2240; Found 789.2235.

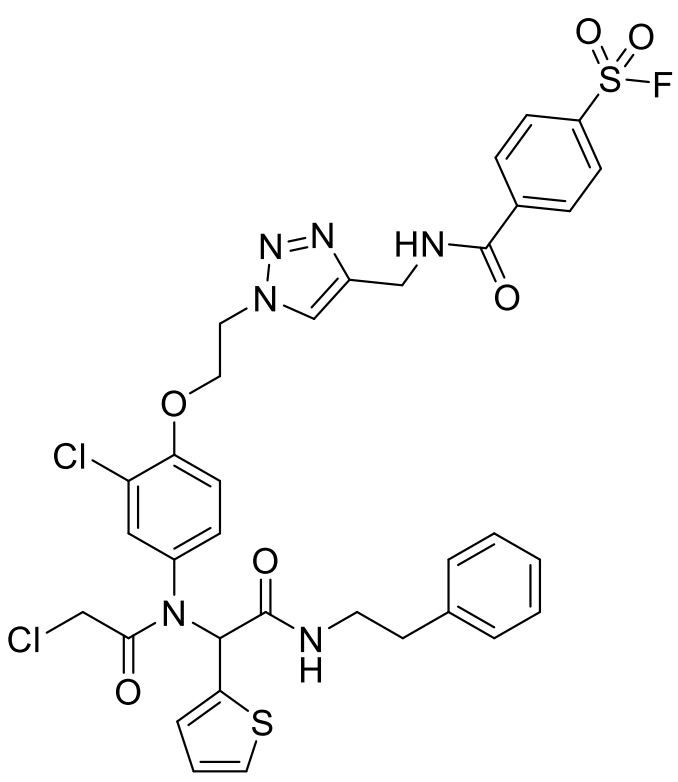

5071

4-(((1-(2-(2-chloro-4-(2-chloro-*N*-(2-oxo-2-(phenethylamino)-1-(thiophen-2-yl)ethyl)acetamido) phenoxy)ethyl)-1*H*-1,2,3-triazol-4-yl)methyl)carbamoyl)benzenesulfonyl fluoride **(5071):** White solid, 104.7mg, 67.7% yield. ^{1}H NMR (400 MHz, $CDCl_3$) δ 8.07 (s, 4H), 7.48 (s, 1H), 7.29 – 7.22 (m, 4H), 7.22 – 7.15 (m, 1H), 7.15 – 7.07 (m, 2H), 6.85 (m, 2H), 6.70 (s, 1H), 6.07 (m, 2H), 5.03 – 4.57 (m, 4H), 4.37 (s, 2H), 3.76 (s, 2H), 3.55 (m, 2H), 2.81 (m, 2H). ESI-MS calcd for $C_{34}H_{32}Cl_2FN_6O_6S_2$ $[M+H]^+$ m/z = 773.1186; Found 773.1176.

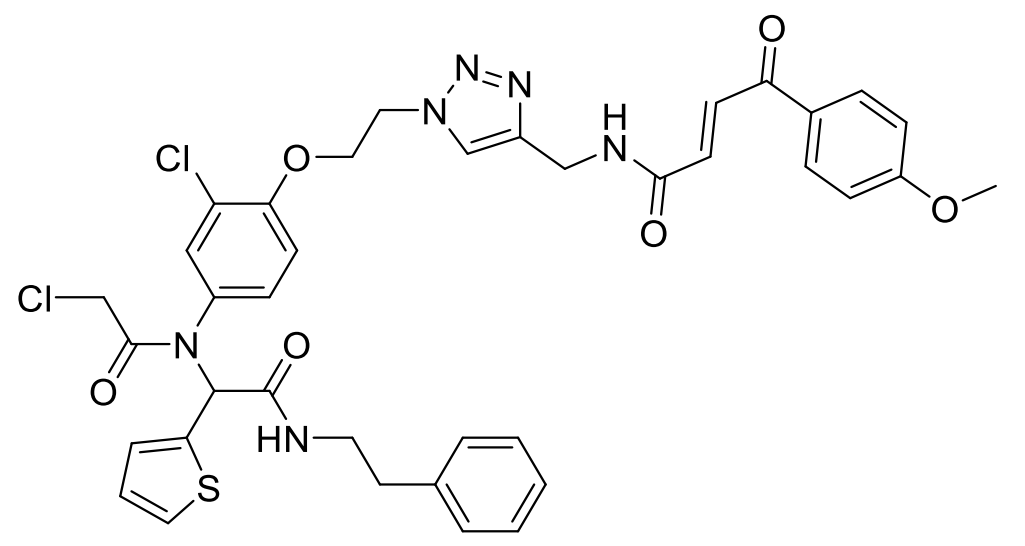

5072

(*E*)-*N*-((1-(2-(2-chloro-4-(2-chloro-*N*-(2-oxo-2-(phenethylamino)-1-(thiophen-2-yl)ethyl)acetamido) phenoxy)ethyl)-1*H*-1,2,3-triazol-4-yl)methyl)-4-(4-methoxyphenyl)-4-oxobut-2-enamide **(5072):** White solid, 128.2mg, 80.4% yield. ^{1}H NMR (400 MHz, $CDCl_3$) δ 8.11 – 7.78 (m, 4H), 7.33 – 6.92 (m, 11H), 6.90 – 6.57 (m, 4H), 6.15 (m, 2H), 4.81 (d, *J* = 6.0 Hz, 2H), 4.69 (d, *J* = 5.7 Hz, 2H), 4.38 (s, 2H), 3.96 – 3.84 (m, 3H), 3.78 (d, *J* = 2.5 Hz, 2H), 3.57 (m, 2H), 2.83 (m, 2H). ESI-MS calcd for $C_{38}H_{36}Cl_2N_6NaO_6S$ $[M+Na]^+$ m/z = 797.1692; Found 797.1690.

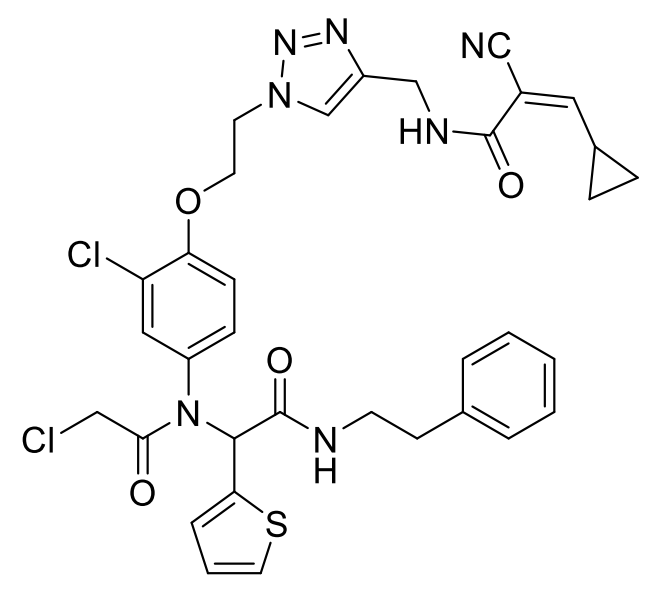

6101

N-((1-(2-(2-chloro-4-(2-chloro-*N*-(2-oxo-2-(phenethylamino)-1-(thiophen-2-yl)ethyl)acetamido) phenoxy)ethyl)-1*H*-1,2,3-triazol-4-yl)methyl)-2-cyano-3-cyclopropylacrylamide **(6101)**: White solid, 66.4 mg, 47.1% yield. ^{1}H NMR (400 MHz, $CDCl_3$) δ 7.90 (s, 1H), 7.34 – 7.11 (m, 7H), 7.06 (d, *J* = 11.4 Hz, 1H), 6.88 (m, 2H), 6.81 – 6.53 (m, 2H), 6.08 (d, *J* = 14.4 Hz, 2H), 4.83 (t, *J* = 4.9 Hz, 2H), 4.66 (d, *J* = 5.7 Hz, 2H), 4.36 (t, *J* = 4.9 Hz, 2H), 3.80 (s, 2H), 3.58 (m, 2H), 2.93 – 2.72 (m, 2H), 2.05 (m, 1H), 1.36 – 1.28 (m, 3H), 0.97 (m, 2H). LC-MS calcd for $C_{34}H_{34}Cl_2N_7O_4S$ $[M+Na]^+$ m/z = 706.2; Found 706.2.

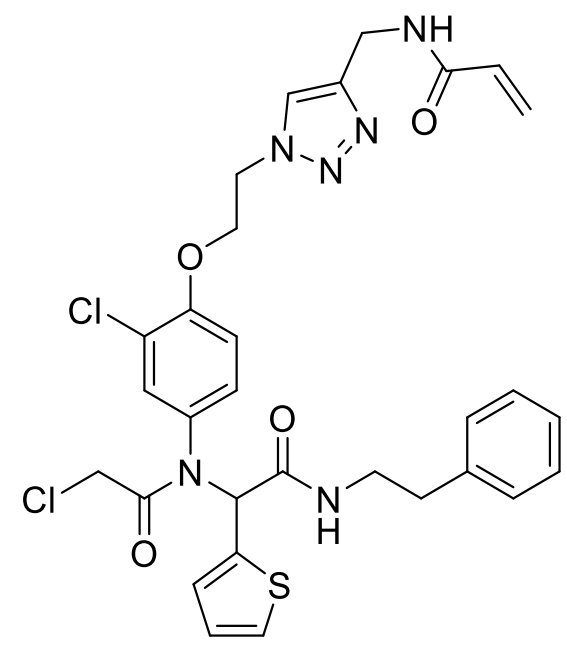

6076

N-((1-(2-(2-chloro-4-(2-chloro-*N*-(2-oxo-2-(phenethylamino)-1-(thiophen-2-yl)ethyl)acetamido)phenoxy) ethyl)-1*H*-1,2,3-triazol-4-yl)methyl)acrylamide **(6076)**: White solid, 105.8 mg, 82.5% yield. ^{1}H NMR (400 MHz, $CDCl_3$) δ 7.88 (s, 1H), 7.30 – 7.03 (m, 7H), 6.86 (m, 2H), 6.69 (s, 1H), 6.52 (d, *J* = 5.9 Hz, 1H), 6.31 (dd, *J* = 17.0, 1.5 Hz, 1H), 6.22 – 6.01 (m, 3H), 5.66 (dd, *J* = 10.3, 1.5 Hz, 1H), 4.79 (t, *J* = 4.9 Hz, 2H), 4.60 (d, *J* = 5.7 Hz, 2H), 4.34 (d, *J* = 5.2 Hz, 2H), 3.78 (s, 2H), 3.55 (m, 2H), 2.91 – 2.66 (m, 2H). ^{13}C NMR (151 MHz, $CDCl_3$) δ 167.9, 166.6, 165.6, 153.8, 144.8, 138.6, 134.6, 132.2, 131.8, 130.5, 130.1, 128.8, 128.6,

128.4, 127.0, 126.6, 126.5, 124.1, 112.8, 67.4, 60.5, 49.5, 42.3, 41.1, 35.5, 35.0. ESI-MS calcd for $C_{30}H_{31}Cl_2N_6O_4S$ $[M+H]^+$ m/z = 641.1505; Found 641.1502.

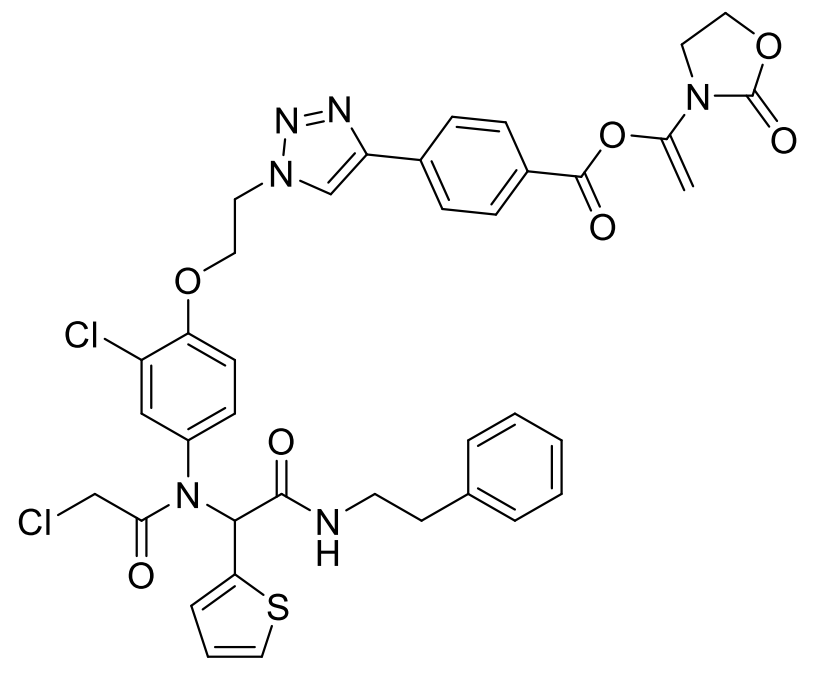

5075

1-(2-oxooxazolidin-3-yl)vinyl 4-(1-(2-(2-chloro-4-(2-chloro-*N*-(2-oxo-2-(phenethylamino)-1-(thiophen-2-yl)ethyl)acetamido)phenoxy)ethyl)-1*H*-1,2,3-triazol-4-yl)benzoate **(5075):** White solid, 119.4 mg, 75.6% yield. ^{1}H NMR (400 MHz, $CDCl_3$) δ 8.31 (s, 1H), 8.19 (d, *J* = 8.0 Hz, 2H), 7.97 (d, *J* = 8.1 Hz, 2H), 7.34 – 7.05 (m, 7H), 6.96 – 6.57 (m, 4H), 6.07 (m, 2H), 4.93 (m, 3H), 4.73 (d, *J* = 3.1 Hz, 1H), 4.41 (t, *J* = 7.9 Hz, 4H), 3.92 (m, 2H), 3.78 (s, 2H), 3.57 (m, 2H), 2.94 – 2.71 (m, 2H). ESI-MS calcd for $C_{38}H_{34}Cl_2N_6NaO_7S$ $[M+Na]^+$ m/z = 811.1484; Found 811.1475.

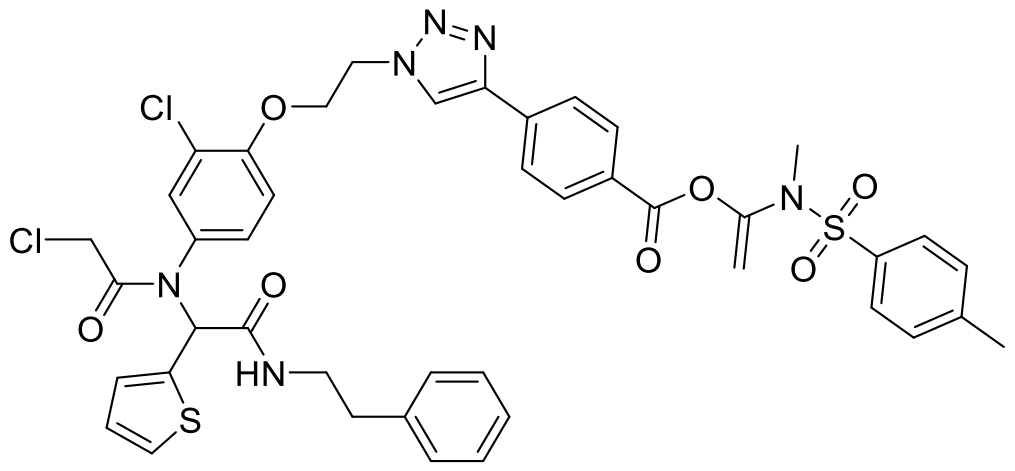

6090

1-((*N*,4-dimethylphenyl)sulfonamido)vinyl 4-(1-(2-(2-chloro-4-(2-chloro-*N*-(2-oxo-2-(phenethylamino)-1- (thiophen-2-yl)ethyl)acetamido)phenoxy)ethyl)-1*H*-1,2,3-triazol-4-yl)benzoate **(6090)**: White solid, 110.6 mg, 62.3% yield. ^{1}H NMR (600 MHz, $CDCl_3$) δ 8.28 (s, 1H), 7.93 (d, *J* = 8.4 Hz, 2H), 7.88 (d, *J* = 8.1 Hz, 2H), 7.75 (d, *J* = 8.2 Hz, 2H), 7.33 – 7.20 (m, 5H), 7.20 – 7.15 (m, 1H), 7.15 – 7.07 (m, 2H), 6.84 (m, 4H), 6.06 (m, 2H), 5.04 (d, *J* = 2.6 Hz, 1H), 4.96 – 4.75 (m, 3H), 4.40 (s, 2H), 3.77 (d, *J* = 1.7 Hz, 2H), 3.55 (m, 2H), 3.11 (s, 3H), 2.80 (m, 2H), 2.39 (s, 3H). ^{13}C NMR (151 MHz, $CDCl_3$) δ 167.9, 166.6, 163.7, 161.3, 153.7, 146.9, 146.7, 144.1, 138.6, 135.7, 134.6, 134.2, 132.3, 131.9, 130.9, 130.1, 129.7, 129.6, 128.8, 128.6, 128.4, 128.0, 126.6, 126.5, 125.5, 122.7, 112.6, 101.7, 67.4, 60.6, 49.6, 42.2, 41.1, 37.2, 35.5, 30.9, 21.6. ESI-MS calcd for $C_{43}H_{41}Cl_2N_6O_7S_2$ $[M+H]^+$ m/z =887.1855; Found887.1860.

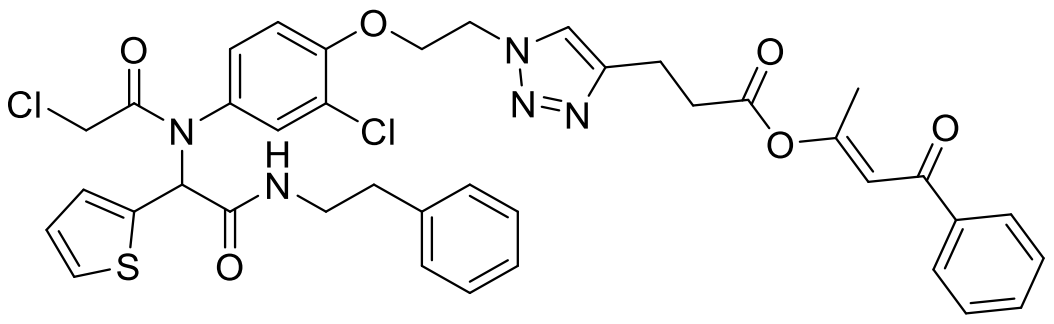

5073

(*E*)-4-oxo-4-phenylbut-2-en-2-yl 3-(1-(2-(2-chloro-4-(2-chloro-*N*-(2-oxo-2-(phenethylamino)-1-(thiophen-2-yl)ethyl)acetamido)phenoxy)ethyl)-1*H*-1,2,3-triazol-4-yl)propanoate **(5073):** White solid, 115.7 mg, 74.6% yield. ^{1}H NMR (400 MHz, $CDCl_3$) δ 7.93 (m, 2H), 7.78 (s, 1H), 7.62 – 7.52 (m, 1H), 7.47 (t, *J* = 7.7 Hz, 3H), 7.33 – 7.24 (m, 4H), 7.25 – 7.17 (m, 1H), 7.17 – 7.10 (m, 2H), 6.92 – 6.56 (m, 5H), 6.08 (m,

2H), 4.82 (t, *J* = 4.8 Hz, 2H), 4.35 (m, 2H), 3.80 (s, 2H), 3.57 (m, 2H), 3.14 (d, *J* = 7.1 Hz, 2H), 2.96 (t, *J* = 6.8 Hz, 2H), 2.91 – 2.73 (m, 2H), 2.38 (s, 3H). ESI-MS calcd for $C_{39}H_{38}Cl_2N_5O_6S$ $[M+H]^+$ m/z = 774.1920; Found 774.1923.

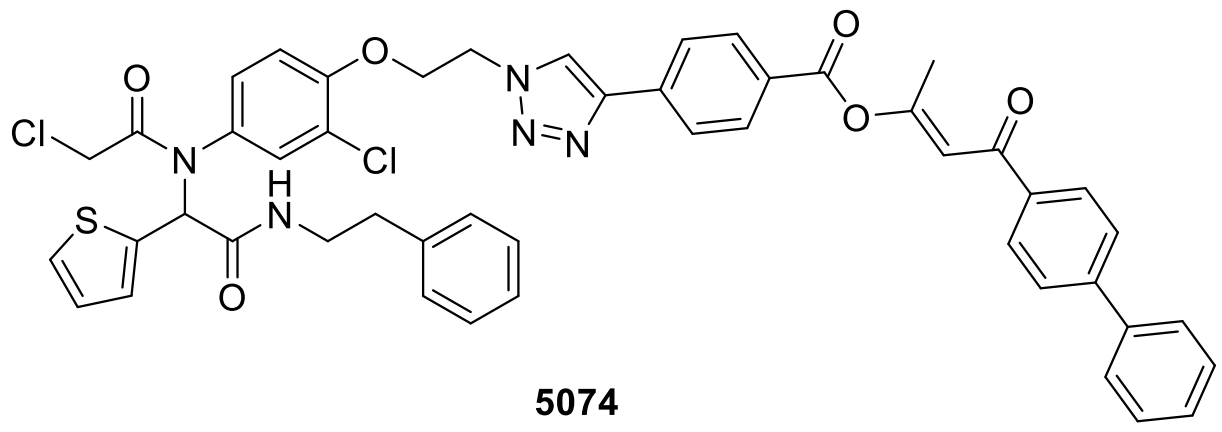

5074

(*E*)-4-([1,1'-biphenyl]-4-yl)-4-oxobut-2-en-2-yl-4-(1-(2-(2-chloro-4-(2-chloro-*N*-(2-oxo-2-(phenethyl amino)-1-(thiophen-2-yl)ethyl)acetamido)phenoxy)ethyl)-1*H*-1,2,3-triazol-4-yl)benzoate **(5074):** White solid, 92.5 mg, 51.5% yield. ^{1}H NMR (400 MHz, $CDCl_3$) δ 8.30 (s, 1H), 8.20 (d, *J* = 8.3 Hz, 2H), 8.09 – 7.91 (m, 4H), 7.74 – 7.66 (m, 2H), 7.66 – 7.59 (m, 2H), 7.47 (dd, *J* = 8.4, 6.6 Hz, 2H), 7.40 (dd, *J* = 8.4, 6.2 Hz, 1H), 7.25 – 7.21 (m, 2H), 7.21 – 7.15 (m, 1H), 7.15 – 7.05 (m, 2H), 7.00 (s, 1H), 6.96 – 6.57 (m, 4H), 6.03 (m, 2H), 4.92 (t, *J* = 4.8 Hz, 2H), 4.40 (s, 2H), 3.76 (s, 2H), 3.55 (m, 2H), 2.81 (m, 2H), 2.57 (s, 3H). ESI-MS calcd for: $C_{49}H_{42}Cl_2N_5O_6S$ $[M+H]^+$ m/z = 898.2233; Found 898.2226.

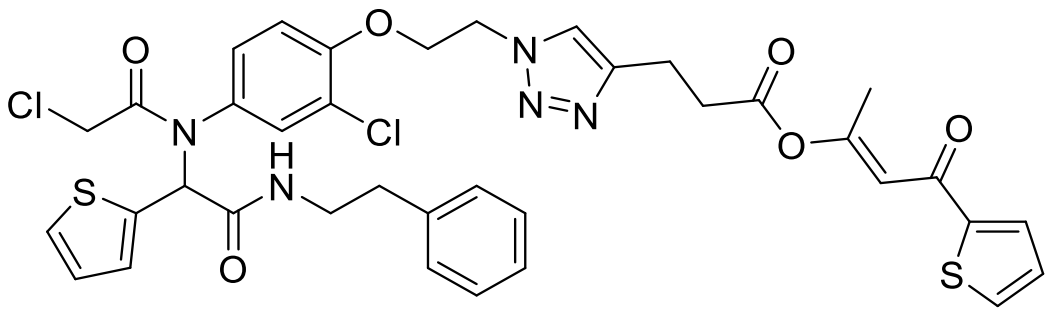

5085

(*E*)-4-oxo-4-(thiophen-2-yl)but-2-en-2-yl-3-(1-(2-(2-chloro-4-(2-chloro-*N*-(2-oxo-2-(phenethyl amino)-1- (thiophen-2-yl)ethyl)acetamido)phenoxy)ethyl)-1*H*-1,2,3-triazol-4-yl)propanoate **(5085):** White solid, 119.3 mg, 76.4% yield. ^{1}H NMR (400 MHz, $CDCl_3$) δ 7.67 (m, 2H), 7.33 – 7.08 (m, 9H), 6.88 (m, 3H), 6.72 (s, 2H), 6.06 (m, 2H), 4.88 (s, 2H), 4.39 (s, 2H), 3.80 (s, 2H), 3.58 (p, *J* = 6.4 Hz, 2H), 3.09 (m, 4H), 2.82 (m, 2H), 2.42 (s, 3H). ESI-MS calcd for $C_{37}H_{36}Cl_2N_5O_6S_2$ $[M+H]^+$ m/z = 780.1484; Found 780.1484.

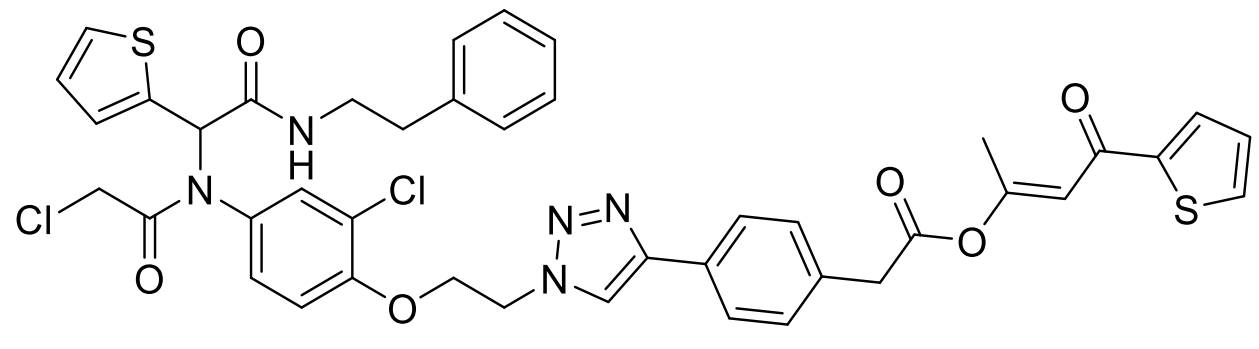

5088

(*E*)-4-oxo-4-(thiophen-2-yl)but-2-en-2-yl-2-(4-(1-(2-(2-chloro-4-(2-chloro-*N*-(2-oxo-2-(phenethyl amino)-1-(thiophen-2-yl)ethyl)acetamido)phenoxy)ethyl)-1*H*-1,2,3-triazol-4-yl)phenyl) acetate **(5088):** White solid, 128.1 mg, 76.1% yield. ^{1}H NMR (400 MHz, $CDCl_3$) δ 7.91 (s, 2H), 7.66 (dd, *J* = 11.6, 4.4 Hz, 2H), 7.45 (s, 3H), 7.28 – 7.06 (m, 7H), 6.86 (d, *J* = 6.1 Hz, 3H), 6.71 (s, 2H), 6.09 (s, 1H), 6.00 (s, 1H), 4.93 (s, 2H), 4.42 (s, 2H), 3.81 (m, 4H), 3.57 (m, 2H), 2.83 (m, 2H), 2.43 (s, 3H). ESI-MS calcd for $C_{42}H_{38}Cl_2N_5O_6S_2$ $[M+H]^+$ m/z = 842.1641; Found 842.1639.

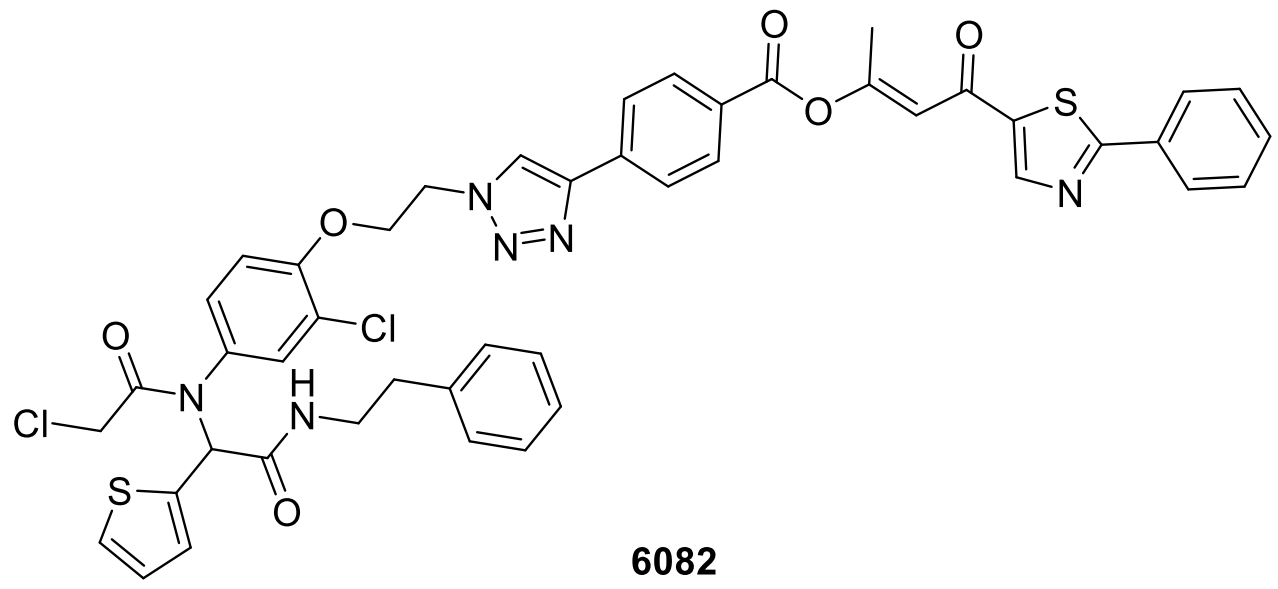

6082

(*E*)-4-oxo-4-(2-phenylthiazol-5-yl)but-2-en-2-yl 4-(1-(2-(2-chloro-4-(2-chloro-*N*-(2-oxo-2-(phenethyl amino)-1-(thiophen-2-yl)ethyl)acetamido)phenoxy)ethyl)-1*H*-1,2,3-triazol-4-yl)benzoate **(6082)**: Light yellow solid, 105.5 mg, 57.0% yield. ^{1}H NMR (600 MHz, $CDCl_3$) δ 8.33 (d, *J* = 21.2 Hz, 2H), 8.20 (d, *J* = 8.3 Hz, 2H), 8.11 – 7.92 (m, 3H), 7.58 – 7.34 (m, 3H), 7.27 – 7.05 (m, 6H), 6.85 (m, 5H), 6.04 (m, 2H), 4.92 (t, *J* = 4.9 Hz, 2H), 4.41 (s, 2H), 3.77 (d, *J* = 1.9 Hz, 2H), 3.55 (m, 2H), 2.81 (m, 2H), 2.61 (s, 3H). ^{13}C NMR (151 MHz, $CDCl_3$) δ 181.7, 174.5, 167.9, 166.6, 165.5, 163.5, 153.7, 146.9, 146.7, 141.7, 138.6, 135.9, 134.6, 133.0, 132.4, 131.4, 131.0, 130.1, 129.7, 129.2, 128.8, 128.6, 128.4, 128.3, 127.1, 126.6, 126.5, 125.7, 122.8, 113.2, 67.4, 60.6, 49.7, 42.2, 41.1, 35.5, 19.5. ESI-MS calcd for $C_{46}H_{38}Cl_2N_6NaO_6S_2$ $[M+H]^+$ m/z = 927.1569; Found 927.1573.

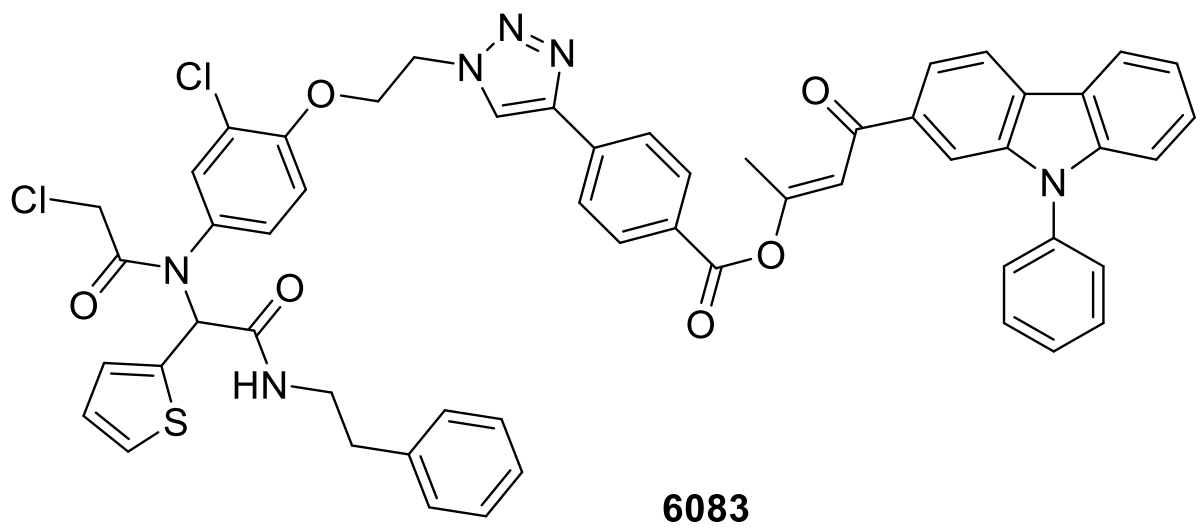

6083

(*E*)-4-oxo-4-(9-phenyl-9*H*-carbazol-2-yl)but-2-en-2-yl 4-(1-(2-(2-chloro-4-(2-chloro-*N*-(2-oxo-2-(phenethylamino)-1-(thiophen-2-yl)ethyl)acetamido)phenoxy)ethyl)-1*H*-1,2,3-triazol-4-yl) benzoate **(6083)**: White solid, 101.7 mg, 51.5% yield. ^{1}H NMR (400 MHz, $CDCl_3$) δ 8.81 (s, 1H), 8.40 – 8.15 (m, 4H), 8.15 – 7.92 (m, 3H), 7.63 (d, *J* = 7.9 Hz, 2H), 7.60 – 7.48 (m, 3H), 7.48 – 7.30 (m, 4H), 7.30 – 7.05 (m, 8H), 6.98 – 6.51 (m, 4H), 6.04 (m, 2H), 5.03 – 4.81 (m, 2H), 4.41 (s, 2H), 3.77 (t, *J* = 2.3 Hz, 2H), 3.56 (s, 2H), 2.80 (m, 2H), 2.59 (t, *J* = 2.2 Hz, 3H). ^{13}C NMR (151 MHz, $CDCl_3$) δ 189.8, 167.9, 166.6, 163.9, 163.0, 153.7, 146.8, 143.6, 141.8, 138.6, 136.9, 135.7, 134.6, 132.3, 131.9, 131.1, 130.9, 130.1, 129.7, 128.8, 128.6, 128.4, 128.1, 127.1, 126.7, 126.5, 125.7, 123.5, 123.2, 122.8, 121.8, 120.9, 120.7, 114.5, 112.6, 110.3, 109.6, 67.4, 60.6, 49.7, 42.2, 41.1, 35.5, 19.1. ESI-MS calcd for $C_{55}H_{45}Cl_2N_6O_6S$ $[M+H]^+$ m/z = 987.2498; Found 987.2497.

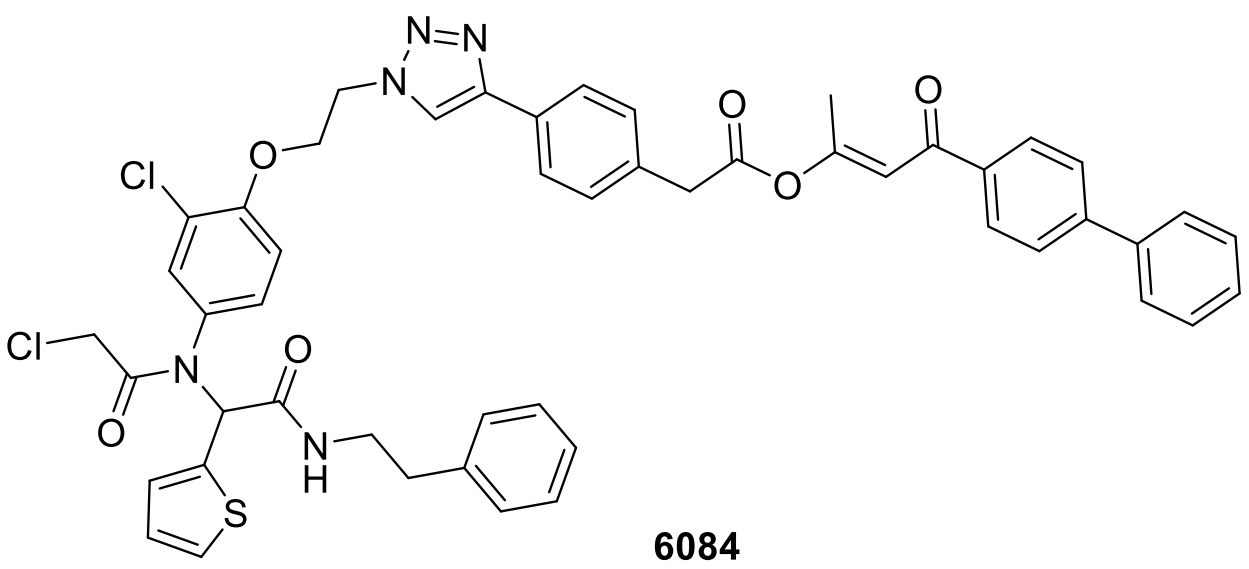

6084

(*E*)-4-([1,1'-biphenyl]-4-yl)-4-oxobut-2-en-2-yl-2-(4-(1-(2-(2-chloro-4-(2-chloro-*N*-(2-oxo-2-(phenethyl

amino)-1-(thiophen-2-yl)ethyl)acetamido)phenoxy)ethyl)-1*H*-1,2,3-triazol-4-yl)phenyl) acetate **(6084)**: White solid, 102.7 mg, 56.3% yield. ^{1}H NMR (400 MHz, $CDCl_3$) δ 8.17 (s, 1H), 8.04 – 7.90 (m, 2H), 7.85 (d, *J* = 8.1 Hz, 2H), 7.72 – 7.56 (m, 4H), 7.54 – 7.33 (m, 5H), 7.33 – 7.06 (m, 7H), 6.83 (d, *J* = 5.3 Hz, 5H), 6.02 (m, 2H), 4.88 (t, *J* = 4.8 Hz, 2H), 4.38 (d, *J* = 5.2 Hz, 2H), 3.86 – 3.72 (m, 4H), 3.55 (m, 2H), 2.90 – 2.67 (m, 2H), 2.39 (s, 3H). ESI-MS calcd for $C_{50}H_{44}Cl_2N_5O_6S$ $[M+H]^+$ m/z = 912.2389; Found 912.2393.

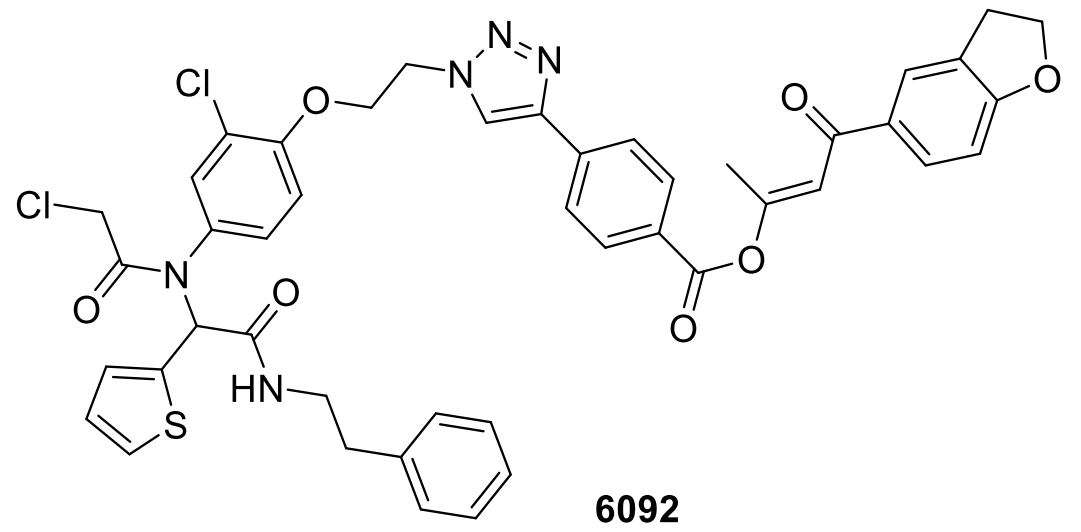

6092

(*E*)-4-(2,3-dihydrobenzofuran-5-yl)-4-oxobut-2-en-2-yl 4-(1-(2-(2-chloro-4-(2-chloro-*N*-(2-oxo-2-(phenethylamino)-1-(thiophen-2-yl)ethyl)acetamido)phenoxy)ethyl)-1*H*-1,2,3-triazol-4-yl) benzoate **(6092)**: White solid, 96.8 mg, 56.0% yield. ^{1}H NMR (600 MHz, $CDCl_3$) δ 8.30 (s, 1H), 8.22 – 8.11 (m, 2H), 7.98 (d, *J* = 8.2 Hz, 2H), 7.87 (d, *J* = 1.7 Hz, 1H), 7.81 (dd, *J* = 8.5, 1.9 Hz, 1H), 7.29 – 7.15 (m, 4H), 7.15 – 7.05 (m, 2H), 6.93 – 6.54 (m, 5H), 6.06 (m, 2H), 4.92 (t, *J* = 4.8 Hz, 2H), 4.66 (t, *J* = 8.8 Hz, 2H), 4.40 (d, *J* = 5.4 Hz, 2H), 3.82 – 3.65 (m, 2H), 3.55 (m, 2H), 3.26 (t, *J* = 8.7 Hz, 2H), 2.80 (m, 2H), 2.50 (d, *J* = 0.9 Hz, 3H). ^{13}C NMR (151 MHz, $CDCl_3$) δ 188.9, 167.9, 166.6, 164.4, 163.8, 162.8, 153.7, 146.8, 138.6, 135.7, 134.6, 132.3, 132.0, 130.9, 130.3, 130.1, 128.8, 128.6, 128.4, 127.8, 126.6, 126.5, 125.6, 122.8, 114.2, 109.1, 72.2, 67.4, 60.6, 49.7, 42.2, 41.1, 35.5, 31.6, 29.0, 22.7, 18.9, 14.1. ESI-MS calcd for $C_{45}H_{40}Cl_2N_5O_7S$ $[M+H]^+$ m/z = 864.2025; Found 864.2018.

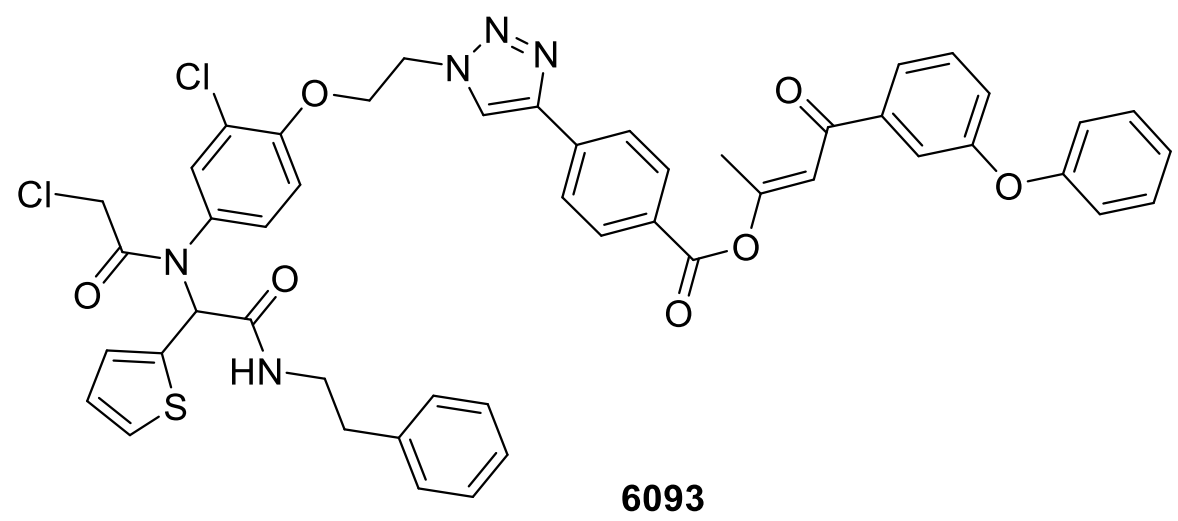

6093

(*E*)-4-oxo-4-(3-phenoxyphenyl)but-2-en-2-yl 4-(1-(2-(2-chloro-4-(2-chloro-*N*-(2-oxo-2-(phenethylamino) -1-(thiophen-2-yl)ethyl)acetamido)phenoxy)ethyl)-1*H*-1,2,3-triazol-4-yl)benzoate **(6093)**: White solid, 122.5 mg, 67.0% yield. ^{1}H NMR (600 MHz, $CDCl_3$) δ 8.30 (s, 1H), 8.18 (d, *J* = 8.2 Hz, 2H), 8.02 – 7.89 (m, 4H), 7.39 (t, *J* = 7.8 Hz, 2H), 7.24 (t, *J* = 6.6 Hz, 2H), 7.19 (m, 2H), 7.12 (d, *J* = 7.4 Hz, 2H), 7.07 (d, *J* = 8.0 Hz, 2H), 7.04 – 6.97 (m, 2H), 6.91 (s, 1H), 6.85 (dd, *J* = 9.3, 4.8 Hz, 2H), 6.72 (s, 3H), 6.06 (s, 1H), 5.99 (s, 1H), 4.92 (t, *J* = 4.8 Hz, 2H), 4.40 (s, 2H), 3.76 (d, *J* = 2.0 Hz, 2H), 3.59 – 3.51 (m, 2H), 2.80 (m, 2H), 2.53 (s, 3H). ^{13}C NMR (151 MHz, $CDCl_3$) δ 188.9, 167.9, 166.6, 163.8, 163.6, 161.9, 155.5, 153.7, 146.8, 138.5, 135.7, 133.3, 132.4, 130.9, 130.5, 130.1, 128.8, 128.6, 128.4, 126.6, 126.5, 125.7, 124.6, 122.8, 120.2, 117.4, 113.8, 67.4, 60.6, 49.7, 42.2, 41.1, 35.5, 22.7, 19.1, 14.1. ESI-MS calcd for $C_{49}H_{42}Cl_2N_5O_7S$ $[M+H]^+$ m/z = 914.2182; Found 914.2180.

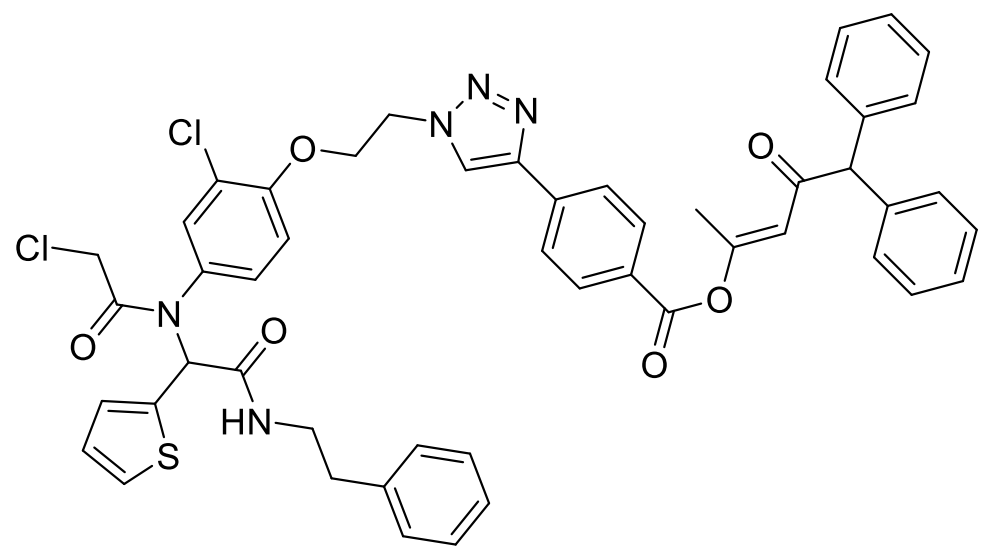

6094

(*E*)-4-oxo-5,5-diphenylpent-2-en-2-yl 4-(1-(2-(2-chloro-4-(2-chloro-*N*-(2-oxo-2-(phenethylamino)-1-(thiophen-2-yl)ethyl)acetamido)phenoxy)ethyl)-1*H*-1,2,3-triazol-4-yl)benzoate **(6094)**: White solid, 96.7 mg, 52.9% yield. ^{1}H NMR (600 MHz, $CDCl_3$) δ 8.27 (s, 1H), 8.13 – 8.03 (m, 2H), 7.93 (d, *J* = 8.1 Hz, 2H), 7.34 (t, *J* = 7.6 Hz, 4H), 7.30 – 7.15 (m, 9H), 7.15 – 7.06 (m, 2H), 6.94 – 6.45 (m, 5H), 6.27 (s, 1H), 6.07 (s, 1H), 6.01 (s, 1H), 5.15 (s, 1H), 4.90 (t, *J* = 4.8 Hz, 2H), 4.38 (s, 2H), 3.76 (d, *J* = 1.9 Hz, 2H), 3.55 (m, 2H), 2.80 (m, 2H), 2.50 (s, 3H). ^{13}C NMR (151 MHz, $CDCl_3$) δ 197.4, 167.9, 166.6, 164.4, 163.5, 161.3, 153.7, 146.7, 138.6, 138.2, 135.7, 134.6, 132.3, 130.8, 130.1, 129.3, 128.8, 128.7, 128.6, 128.4, 127.3, 126.6, 126.5, 125.6, 122.7, 116.3, 112.6, 67.4, 65.7, 49.6, 42.2, 41.1, 35.5, 19.0. ESI-MS calcd for $C_{50}H_{44}Cl_2N_5O_6S$ [M+H]$^+$ m/z = 912.2389; Found 912.2383.

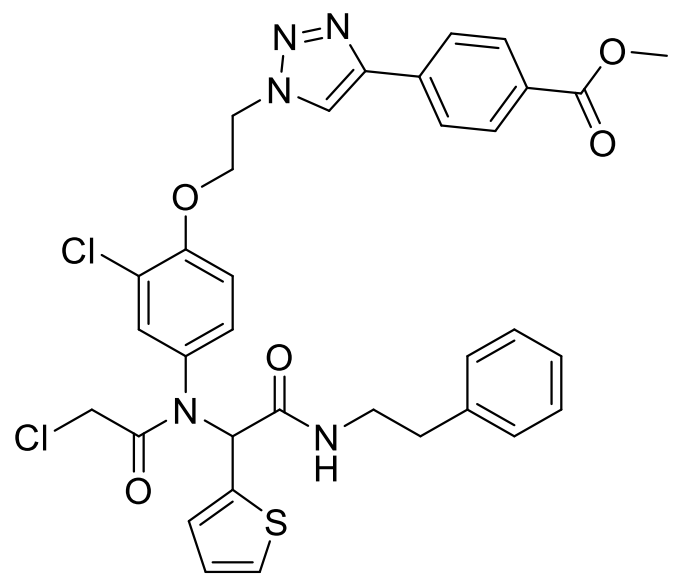

6104

Methyl 4-(1-(2-(2-chloro-4-(2-chloro-*N*-(2-oxo-2-(phenethylamino)-1-(thiophen-2-yl)ethyl) acetamido) phenoxy)ethyl)-1*H*-1,2,3-triazol-4-yl)benzoate **(6104):** White solid, 80.1mg, 57.8% yield. ^{1}H NMR (400 MHz, $CDCl_3$) δ 8.26 (d, *J* = 2.6 Hz, 1H), 8.17 – 8.02 (m, 2H), 7.98 – 7.83 (m, 2H), 7.34 – 7.00 (m, 7H), 6.96 – 6.54 (m, 4H), 6.09 (m, 2H), 4.90 (m, 2H), 4.39 (d, *J* = 5.6 Hz, 2H), 3.93 (t, *J* = 2.6 Hz, 3H), 3.82 – 3.69 (m, 3H), 3.54 (m, 2H), 2.79 (m, 2H). ^{13}C NMR (151 MHz, $CDCl_3$) δ 167.9, 166.7, 166.5, 153.7, 147.0, 138.6, 134.8, 134.7, 132.2, 130.3, 130.1, 129.6, 128.8, 128.6, 128.3, 126.6, 126.5, 125.4, 122.5, 112.6, 67.4, 60.5, 52.2, 49.6, 42.3, 41.1, 35.5. ESI-MS calcd for $C_{34}H_{32}Cl_2N_5O_5S$ [M+H]$^+$ m/z = 692.1501.2389; Found 692.1506.

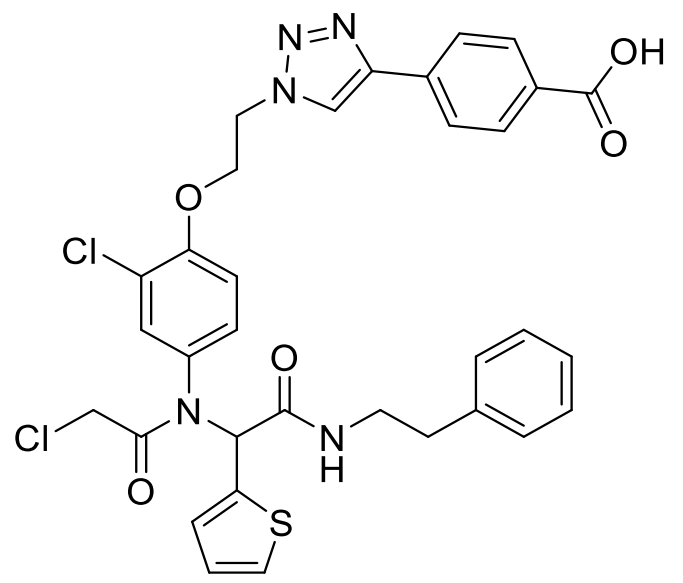

6105

4-(1-(2-(2-chloro-4-(2-chloro-*N*-(2-oxo-2-(phenethylamino)-1-(thiophen-2-yl)ethyl)acetamido)phenoxy) ethyl)-1*H*-1,2,3-triazol-4-yl)benzoic acid **(6105):** White solid, 42.3 mg, 31.2% yield. ^{1}H NMR (400 MHz, $CDCl_3$) δ 8.28 (s, 1H), 8.17 (d, *J* = 8.1 Hz, 2H), 7.95 (d, *J* = 8.1 Hz, 2H), 7.31 – 7.03 (m, 7H), 6.98 – 6.55 (m, 4H), 6.06 (m, 2H), 4.91 (t, *J* = 4.8 Hz, 2H), 4.40 (t, *J* = 4.9 Hz, 2H), 3.77 (s, 2H), 3.54 (m, 2H), 2.89 – 2.70 (m, 2H). ^{13}C NMR (151 MHz, $CDCl_3$) δ 170.3, 167.9, 166.7, 153.7, 147.0, 138.6, 135.5, 134.6, 132.3, 130.9, 130.2, 129.7, 128.9, 128.8, 128.6, 128.4, 126.6, 126.5, 125.6, 122.7, 67.4, 60.6, 49.7, 42.2, 41.1, 35.5. ESI-MS calcd for $C_{33}H_{30}Cl_2N_5O_5S$ $[M+H]^+$ m/z = 678.1345; Found 678.1346.

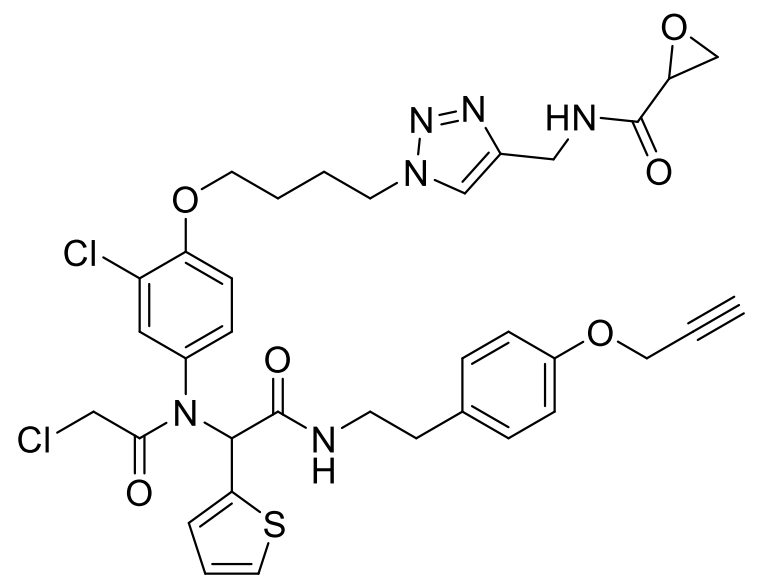

6136

N-((1-(2-(2-chloro-4-(2-chloro-*N*-(2-oxo-2-((4-(prop-2-yn-1-yloxy)phenethyl)amino)-1-(thiophen-2-yl)ethyl)acetamido)phenoxy)ethyl)-1H-1,2,3-triazol-4-yl)methyl)oxirane-2-carboxamide **(6136)**:
^{1}H NMR (400 MHz, $CDCl_3$) δ 7.58 (s, 1H), 7.25 (s, 1H), 7.18 – 6.98 (m, 3H), 6.96 – 6.56 (m, 7H), 6.05 (d, *J* = 28.1 Hz, 2H), 4.65 (d, *J* = 2.4 Hz, 2H), 4.59 – 4.41 (m, 4H), 4.03 (d, *J* = 6.1 Hz, 2H), 3.82 (s, 2H), 3.53 (p, *J* = 6.5 Hz, 2H), 3.44 (m, 1H), 2.97 (t, *J* = 5.1 Hz, 1H), 2.87 – 2.65 (m, 3H), 2.49 (t, *J* = 2.5 Hz, 1H), 2.16 (m, 2H), 1.86 (m, 2H). LC-MS calcd for $C_{35}H_{37}Cl_2N_6O_6S$ [M+H]+ m/z = 739.2; Found 739.2

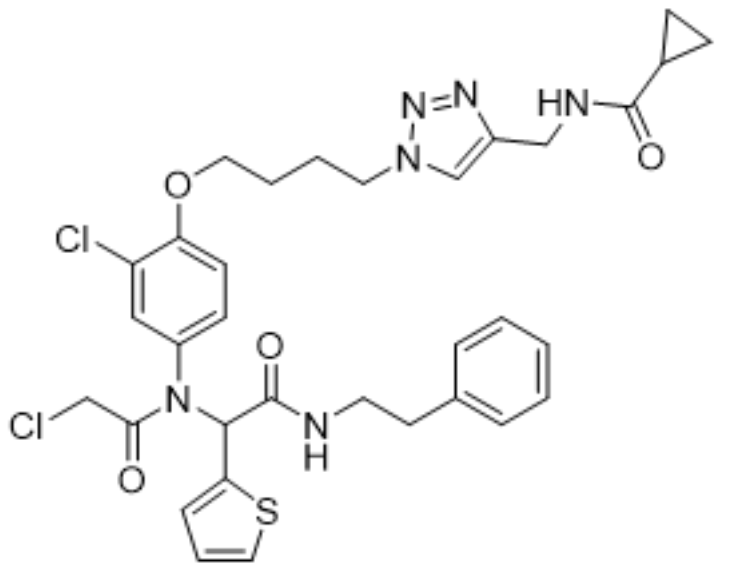

7057

N-((1-(4-(2-chloro-4-(2-chloro-N-(2-oxo-2-(phenethylamino)-1-(thiophen-2-yl)ethyl)acetamido)phenoxy) butyl)-1*H*-1,2,3-triazol-4-yl)methyl)cyclopropanecarboxamide (**7057**): White solid, 101.2 mg, 74% yield. ^{1}H NMR (400 MHz, $CDCl_3$) δ 7.58 (s, 1H), 7.31 – 7.07 (m, 7H), 6.87 (d, *J* = 4.7 Hz, 4H), 6.54 (d, *J* = 6.5 Hz, 1H), 6.09 (s, 2H), 4.59 – 4.36 (m, 4H), 4.02 (t, *J* = 5.6 Hz, 2H), 3.81 (d, *J* = 1.7 Hz, 2H), 3.56 (m, 2H), 2.88 – 2.71 (m, 2H), 2.15 (m, 2H), 1.91 – 1.77 (m, 3H), 1.39 (m, 1H), 1.00 – 0.92 (m, 2H), 0.74 (m, 2H).

6. Cell viability assay

Cell viability was determined by using a CCK-8 assay. The procedures were similar to previously published protocols[2,3], 8000 cells per well were seeded in a 96-well plate and incubated for 24 h in a humidified incubator for adherence. The compounds (0 μM to 10 μM) were dissolved in DMSO and added to each well, while maintaining a final DMSO concentration of 0.1%. After incubation for 72 h, 10 μL of

Cell Counting Kit-8 (CCK-8, DOJINDO, #CK04) was added to each well and incubated for 2 h. After that, the absorbance was measured at 450 nm and 650 nm on a plate reader (Synergy HI, BioTek Instruments, Inc. Vermont, US). Cell viability rate was determined as VR = $(A–A_0)/(As–A_0) \times 100$ %, where A is the absorbance of the experimental group, As is the absorbance of the control group (DMSO was used as the control) and A_0 is the absorbance of the blank group (no cells). IC_{50} values were calculated using GraphPad Prism.

7. Cell lysis and western blot

For western blot experiments, cells were washed twice with cold phosphate buffered saline (PBS), collected with 1 × trypsin or a cell scraper, and centrifuged to generate a protein lysate. Cell pellets were then washed with PBS, lysed with RIPA (Thermo ScientificTM, #89900) containing 2.5% protease and phosphatase inhibitors (Beyotime, P1046). The protein concentration was determined by using the BCA protein detection kit (Beyotime, P0009) and a Synergy H1 hybrid multi-mode reader (BioTek). Samples were dissolved by SDS-polyacrylamide gels and transferred to polymer membranes. The membrane was then blocked with 5% bovine serum albumin (BSA) in TBST (0.1% Tween in Tris buffer) for 1 h at room temperature. After blocking, the membrane was incubated with the corresponding primary antibody at 4℃ overnight. After incubation, the membrane was washed with TBST (4×10 min) and then incubated with the appropriate secondary antibody. The blots were washed again with TBST, and finally imaged with Amersham Imager 600 (GE Healthcare) after development with the Super Signal Sidurah Kit (Thermo Scientific).

All data are presented as mean ± SD from at least three independent experiments unless otherwise stated. For Western blot, protein band intensities were quantified using ImageJ and normalized to the corresponding loading control (Actin). Statistical comparisons between two groups were performed using unpaired two-tailed Student's t-test. P value of less than 0.05 was considered statistically significant (n.s., not significant). All statistical analyses were conducted using GraphPad Prism 9.0 (GraphPad Software, San Diego, CA, USA). For in vivo studies, n = 5 mice per group, and data are presented as mean ± SEM.

8. Immunoprecipitations

Cells were suspended and lysed in NP-40 lysis buffer supplemented with complete protease inhibitor cocktail. The cell suspension was incubated on ice for 1 hour. Subsequently, the mixture was centrifuged at 3,000g for 10 min at 4 ℃ and the resulting supernatant was extracted for use in immunoprecipitation. For immunoprecipitation, FLAG or HA affinity gel (25 µL slurry per sample) was added to the protein lysates and rotated at 4 ℃ for 2 h. The affinity gel was then washed four times with immunoprecipitation washing buffer consisting of 0.2% NP-40, 25 mM Tris-HCl pH 7.4 and 150 mM NaCl. Subsequently, the affinity gel was mixed with Loading buffer and heated at 95℃ for 10 min. The resulting supernatant, containing the eluted proteins, was collected and used for subsequent Western blot analysis.

9. Target validation by pulldown/western blot

HT-1080 cells were used in this study. Cells were pretreated with either **DK-6135** (100 nM)/**DK-6136** (1 µM) or vehicle (DMSO) for 4 h. Upon cell lysis, the protein concentration was determined by BCA protein assay and then diluted to 1 mg/mL with PBS. A freshly premixed click chemistry cocktail was added (100 µM biotin-N_3 from 10 mM stock solution in DMSO, 100 µM TBTA from 10 mM freshly prepared stock

solution in DMSO, 2 mM sodium ascorbate from 200 mM freshly prepared stock solution in deionized water, and 1 mM $CuSO_4$ from 100 mM freshly prepared stock solution in deionized water). The reaction was further incubated for 2 h with gentle mixing prior to precipitation by addition of prechilled methanol (-20℃). Precipitated proteins were subsequently collected by centrifugation (14000 rpm × 10 min at 4℃) and dissolved in PBS containing 1% SDS. Upon incubation with streptavidin beads for 4 h at room temperature, the beads were washed with PBS containing 1% SDS (3 × 1 mL) and PBS (3 × 1 mL). 50 µL (2 ×) SDS loading buffer was added and was heated for 30 min at 95℃. The samples were separated by SDS-PAGE (8% gel) and the target protein was validated by standard Western blot procedure [4].

10. Co-IP/LC-MS experiment

HT-1080 cells were used in this study. Cells were incubated with MG132 (5 µM) for 2 h, and then incubated with **DK-6075** (0.5 µM) / **DK-5070** (50 nM) or DMSO for 8h. Cells were suspended and lysed in NP-40 lysis buffer supplemented with complete protease inhibitor cocktail. The cell suspension was incubated on ice for 1 h. Subsequently, the mixture was centrifuged at 3,000g for 10 min at 4℃. The protein concentration was determined by BCA protein assay. For immunoprecipitation, GPX4 antibody was added to the protein lysates and rotated at 4 ℃ for 4 h. 50 µL of protein A/G Agarose (Beyotime, P2012) was then added to each group of samples for enrichment. The Agarose was then washed four times with precooled PBS. Subsequently, the sample was resuspended in 500 µL ammonium bicarbonate (50 mM) and incubated with 25 µL of 200 mM DTT for 30 min at 37℃, followed by 25 µL of 400 mM IAA for 1 h at room temperature in the dark. Dilute the sample with 500 µL ammonium bicarbonate and digest overnight at 37℃ with 5 µg trypsin. The peptides were desalted by C18 column and then analyzed by LC-MS/MS [5].

11. LC-MS/MS analysis

LC-MS/MS analysis was performed on EASY-nLC 1200 HPLC system coupled to an QExactive Plus (Thermo Fisher Scientific). About 1 µg of each sample was injected into a trap column (75 µm × 2 cm, C18, 3 µm, 100 Å, 164535) and an Acclaim PepMapC18 RSLC 75 µm ID × 25 cm separation column in an EASY-Spray setting, S41S42 three injections were loaded for each sample continuously. Peptides were separated with a 70-min gradient (buffer A: 0.1% formic acid in deionized water; buffer B: 0.1% formic acid in 80% acetonitrile with a flow rate of 0.3 µL/min: 51 min of 4-28 % B, 5 min of 28-38% B, 4 min of 38-90% B, 5 min of 90-4% B, 5 min of 4% B). Separated peptides were then directly analyzed on the QE Plus in a data-dependent manner. Peptides were ionized by using spray voltage of 2.4 kV and the ion transfer tube temperature was 320℃, with automatic switching between MS and MS/MS scans using exclusion duration 25 s. Mass spectra were acquired at a resolution of 60,000 with a target value of 3 × 106 ions or a maximum integration time of 20 ms. The scan range was limited from 350 to 1500 m/z. Peptide fragmentation was performed via higher-energy collision dissociation (HCD) with the energy set to 30%. High-resolution MS2 spectra were acquired with an exclusion duration of 25 s in the orbitrap with a maximum injection time of 40 ms at 17500 resolution (isolation window 1.6 m/z), an AGC target value of 1× 105 and normalized collision energy of 30%. The fixed first m/z was 100, and the isolation window was 1.6 m/z.

12. Database Search

The protein identification raw data were processed by using MaxQuant and processed as per default workflow. The resulting mass spectrum data were analyzed quantitatively using Perseus software [6]. The

identification of modification site raw data was processed by using pFind software and processed as per default workflow. MS tolerance is 4.5 ppm, and MS/MS tolerance is 20 ppm. Reversed database searches were used to evaluate false discovery rate (FDR) of peptide and protein identifications. Two missed cleavage sites of trypsin were allowed., compound modification was set as variable modifications. The FDR of both peptide identification and protein identification were set to be 1%. The options of "second peptides", "match between runs" and "dependent peptides" were used. Label-free quantification was used to quantify the difference between different samples. All raw mass spectrometry data have been stored in a public database PRIDE with the data identifier PXD080539.

13. Modification site analysis of recombinant UHRF1 protein with DK-7062

Recombinant UHRF1 protein was diluted to 1 mg/mL with PBS and was incubated with the probe **DK-7062** (final concentration is 5 μM) at 37 °C for 5 h. The resulting mixture was transferred to the pre activated ultrafiltration membrane spin column, followed by washing with PBS (3 times), 1 × TEAB (2 times), and finally reconstituted with TEAB. The reaction solution was resuspended with 1 M DTT solution (final concentration is 10 mM), and incubated at 37 °C for 30 min. For alkylation, 1 M IAA aqueous solution (final concentration is 20 mM) was added followed by incubation for 30 min at rt. in dark. After alkylation, DTT aqueous solution (final concentration is 10 mM) was added. The samples were washed with PBS and TEAB buffer 6 times, respectively, and reconstituted with TEAB. For the digestion, 1 μL trypsin (1 μg/μL) was added in a ratio of 1:50 (amount of protein). The reaction was incubated at room temperature overnight. After that, the filtrates were collected by centrifugation and washed twice with ultrapure water. The washing buffer were combined and then desalted by C18 column. After evaporation, the samples were analyzed by LC-MS/MS.MS/MS[7].

14. Determination of GSH

HT1080 cells were seeded into 10 cm-diameter culture dishes at a density of 2 × 10^6 cells overnight and then exposed to **DK-5070** (50 nM), **DK-6075** (500 nM), or ML162 (500 nM), respectively. After 24 hours of incubation, the culture medium was discarded, and the cells were digested with trypsin. The cells were collected and washed with PBS, and three volumes of protein removal reagent S were added. Two freeze-thaw cycles were performed using liquid nitrogen and a 37 °C water bath, followed by a 5-minute ice bath. The samples were centrifuged at 10,000 × g for 10 minutes at 4 °C. The supernatant was collected and measured according to the instructions of the Total Glutathione Assay Kit (Beyotime Biotechnology, S0053) using a kinetic assay method[8].

15. Detection of GPX4-DK5070-UHRF1 ternary complex by surface plasmon resonance (SPR)

DK-5070 molecules were diluted in PBS to a maximum concentration of 25 μM, with 3-fold dilutions resulting in 10 different concentrations. The recombinant UHRF1 protein solution was diluted 2-fold to a maximum concentration of 10 μM, with 10 different concentrations. The mixed solution of DK-5070 and UHRF1 with a maximum concentration of 10 μM, with 25 μM DK-5070 in PBS as the solvent. Detection was then performed using a Biacore S200 analyzer.

16. Determination of malondialdehyde (MDA)

HT1080 cells were seeded into 10 cm diameter culture dishes at a density of 2 × 10^6 cells overnight and

then exposed to **DK-5070** (50 nM), **DK-6075** (500 nM), or ML162 (500 nM), respectively. The cells were cultured for another 24 hours, and then the culture medium was discarded. The cells were washed with PBS and lysed using IP lysis buffer (Beyotime, P0013F). After lysis, the samples were centrifuged at 12,000 × g for 15 minutes at 4 °C. The supernatant was collected, and the MDA content was determined according to the instructions of the Lipid Peroxidation MDA Assay Kit (Beyotime Biotechnology, S0131S) [8].

17. Determination of iron (Fe^{2+}) intensity

HT1080 cells were seeded into 10 cm culture dishes at a density of 2 × 10^6 cells overnight, and then treated with **DK-5070** (50 nM), **DK-6075** (500 nM), or ML162 (500 nM), respectively. After 24 hours of drug treatment, the cells were digested and collected. The cells were detected according to the instructions of the Cell Ferrous Iron (Fe^{2+}) Fluorometric Assay Kit (E-BC-F101, Elabscience Biotechnology Co., Ltd.). In brief, the cells were washed twice with Reagent 1 working solution from the kit, and then Reagent 2 working solution was added (1 mL per 10^6 cells). The cells were incubated at 37 °C in the dark for 30–60 minutes. The samples were centrifuged at 300 × g for 5 minutes, and the supernatant was discarded. The cells were washed 2–3 times with Reagent 1 working solution. After resuspending the cells in 0.2–0.5 mL of Reagent 1 working solution, the samples were detected using a fluorescence microplate reader (excitation wavelength: 542 nm, emission wavelength: 575 nm).

18. Animals and animal model

After in vitro expansion of HT-1060 cells in 30 culture dishes, the cells were harvested into 50 mL centrifuge tubes and centrifuged at 800 rpm. The supernatant was discarded, and the cells were pooled into one 15 mL sterile centrifuge tube. The cells were washed once with phosphate-buffered saline (PBS), centrifuged again at 800 rpm, and resuspended in an appropriate volume of normal saline. After cell counting, the cell suspension was adjusted to 4 × 10^7 cells/mL.

Each mouse was inoculated subcutaneously with 0.2 mL of the tumor cell suspension in the right anterior axilla. Daily observations of the inoculation site were performed after inoculation. When the tumor volume reached 100–200 mm^3 (approximately 14 days after inoculation), the animals were randomly divided into 2 groups of 6 mice each, to ensure uniformity in tumor volume and body weight among groups. The mean tumor volume of each group was controlled to deviate no more than ±10% from the overall mean tumor volume of all experimental animals. Subsequently, the two groups of animals were administered intraperitoneally for 10 consecutive days: the test compound **DK-5070** (10 mg/kg and 30 mg/kg, once daily) and vehicle (5% DMSO + 25% castor oil solution ($V_{castor\ oil}$: $V_{ethanol}$ = 7:3) + 70% normal saline, 0.1 mL/10 g, once daily), respectively. Body weight and tumor size were measured every other day. Tumor volume (V) was calculated using the formula: $V = L \times W^2/2$, where L = tumor length and W = tumor width. On day 10, all mice were sacrificed and dissected. Tumor tissues were excised, weighed, and recorded. Finally, tumor tissues and other tissues were rapidly frozen in liquid nitrogen or fixed in formalin solution for subsequent experimental analysis. Tumor growth inhibition (TGI) was calculated as: $TGI = (1 - V_{treated} / V_{vehicle}) \times 100\%$, where $V_{treated}$ and $V_{vehicle\ represent}$ the mean tumor volumes of the treated group and the vehicle group, respectively.

19. Molecular docking

Models used: for calculations is used PDB model 6W92, containing structure of tandem tudor domain of

the epigenetic factor UHRF1 [9]. This model was prepared for calculations using Protein PrepWizard (Schrodinger) [10]. This procedure was employed to correct structural deficiencies in the protein model, such as missing amino acid side chains, incorrect bond orders, improper protonation states, and gaps in the protein sequence (missing segments). All manipulations performed in OPLS4 forcefield. Molecular docking was performed within a region centered around Cys172 (residue 170 in the reported uniprot sequence). The GridBox was defined based on the centroid coordinates of residues located within a radius of 4 Å from Cys172 (170). No positional constraints were imposed during the docking process. For compound DK5084, covalent docking was conducted utilizing an epoxide ring-opening reaction mechanism. In contrast, classical non-covalent docking was performed for compound DK5070. The binding free energy for all compounds was calculated using the MM-GBSA method. All calculations performed with Glide algorithm [11, 12] and Prime MM-GBSA [13].

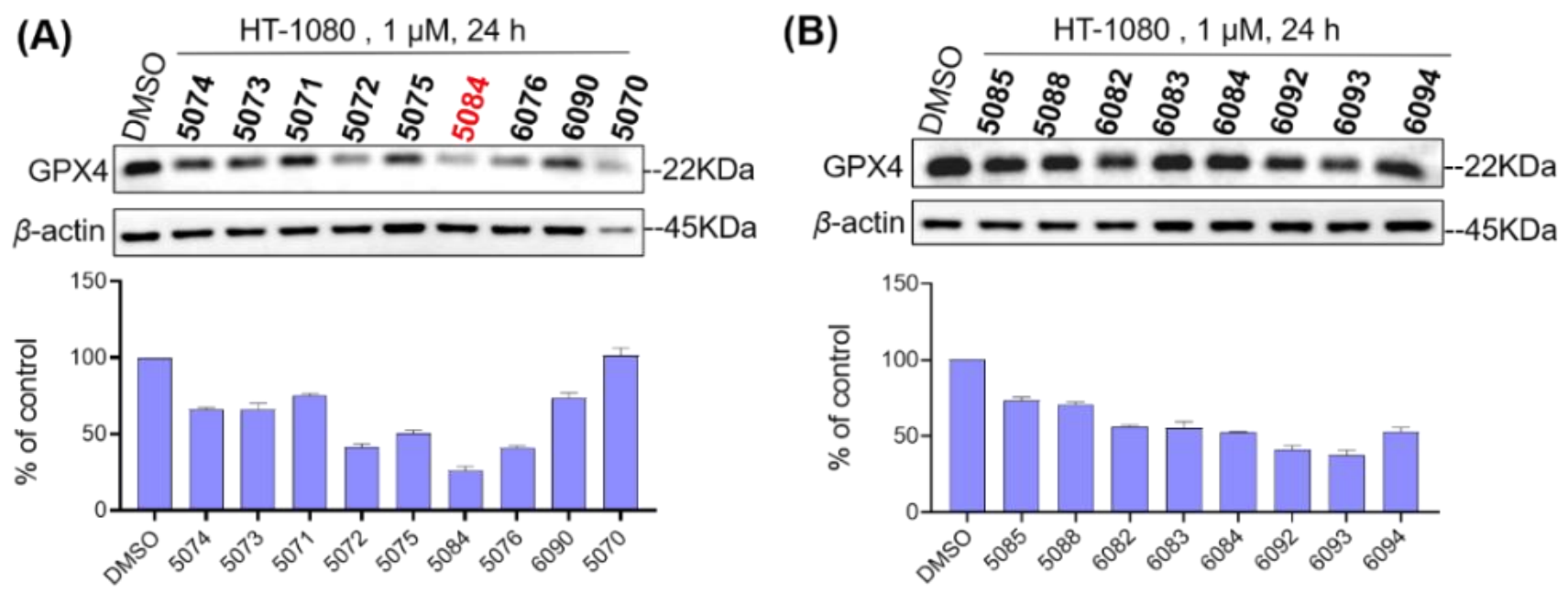

Figure S1. (A) (B) Evaluation of ML-162 analogs with different electrophilic warheads in HT-1080 cells to identify compounds that mediated degradation of GPX4. HT-1080 cells were treated with DMSO or compounds (1µM) for 24 h.

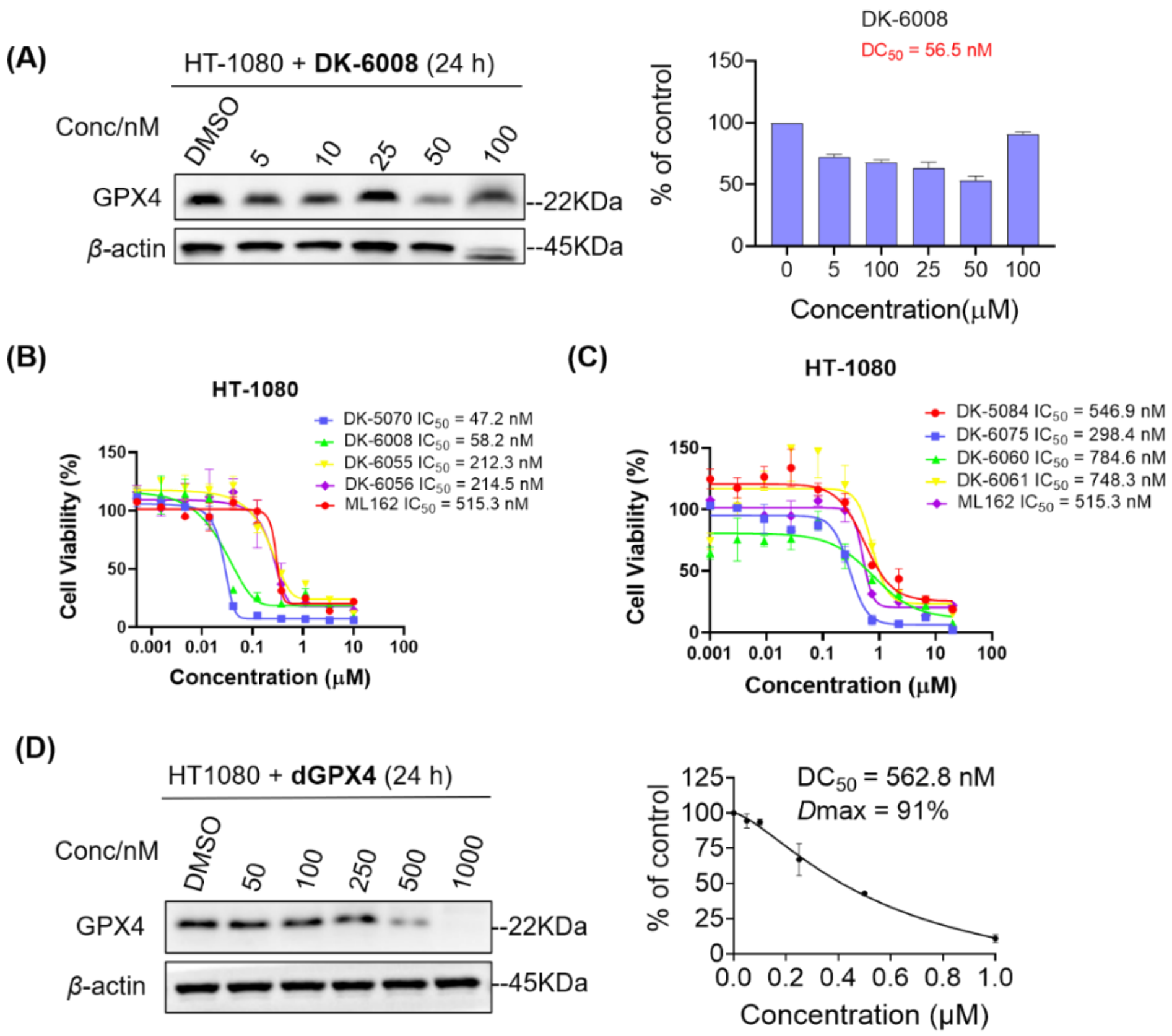

Figure S2. **(A)** The dose-response of DK-6008 on GPX4 degradation in HT-1080 cells. GPX4 and loading control Actin levels were assessed by western blotting. **(B) (C)** Proliferation assay of Compounds on HT-1080 cell were incubated with Compounds or ML162 for 72 h. Data were obtained from two biologically independent experiments. **(D)** The dose-response of dGPX4 on GPX4 degradation in HT-1080 cells. GPX4 and loading control Actin levels were assessed by western blotting.

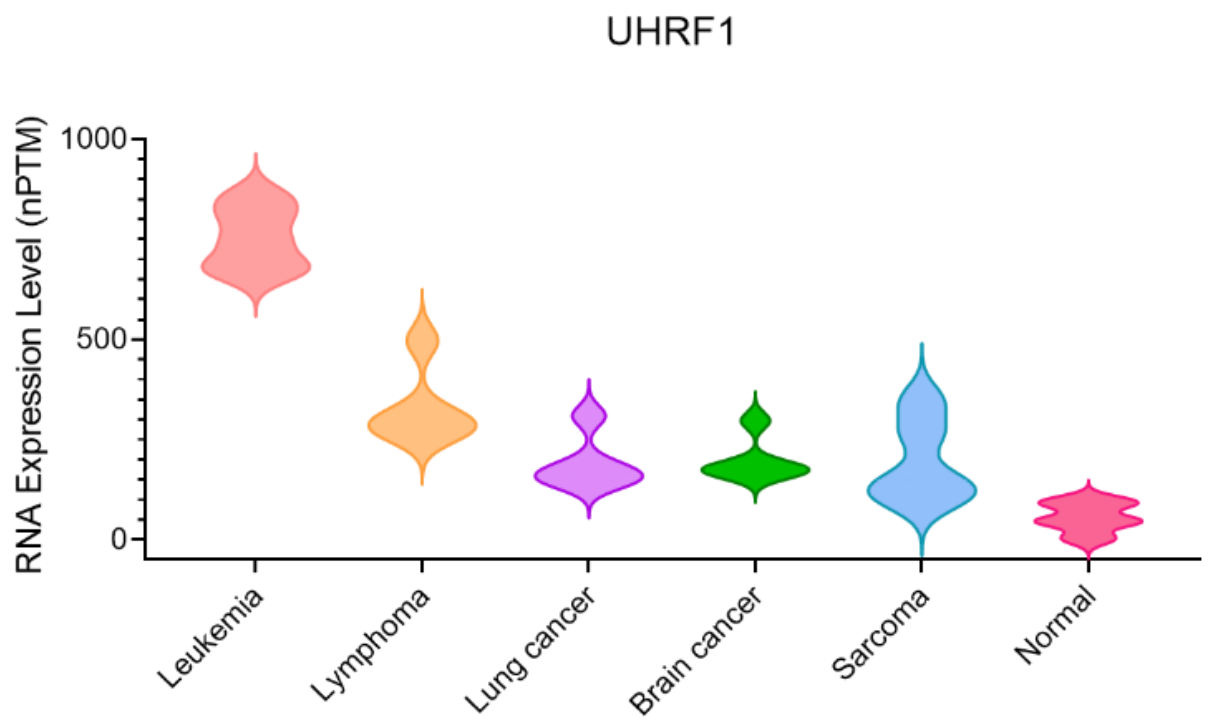

Figure S3. Violin plot demonstrates differential UHRF1transcript expression in cancer versus normal tissues. Data from The Human Protein Atlas.

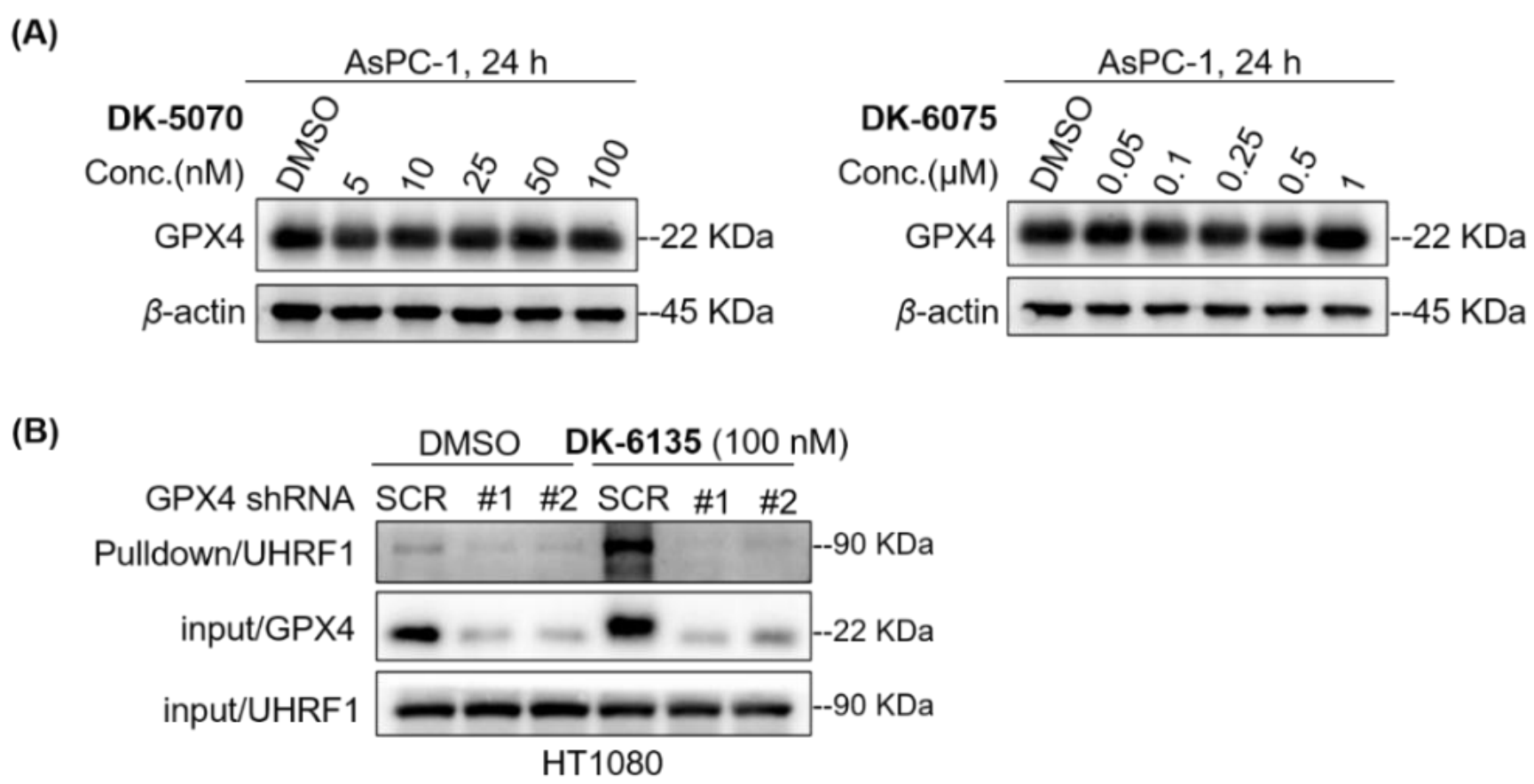

Figure S4. **(A)** The dose-response of **DK-5070** and **DK-6075** on GPX4 degradation in AsPC-1 cells. GPX4 and loading control Actin levels were assessed by western blotting. **(B)** HT1080 cell stably expressing shScramble (shScr) or shGPX4 (#1 and #2) were targeted validation by pull-down/WB with **DK-6135** (100 nM) and indicated antibodies.

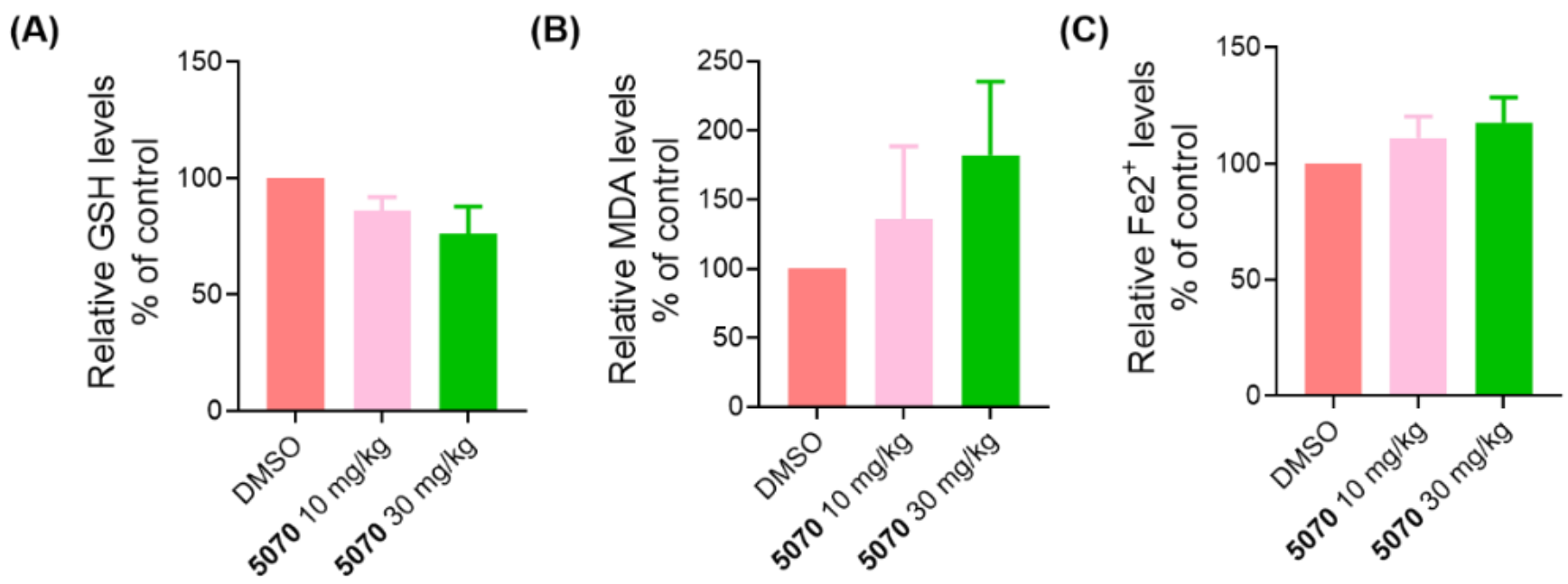

Figure S5. **(A) (B) (C)** Levels of glutathione (GSH), malondialdehyde (MDA), and ferrous iron (Fe^{2+}) were measured in tumor lysates.

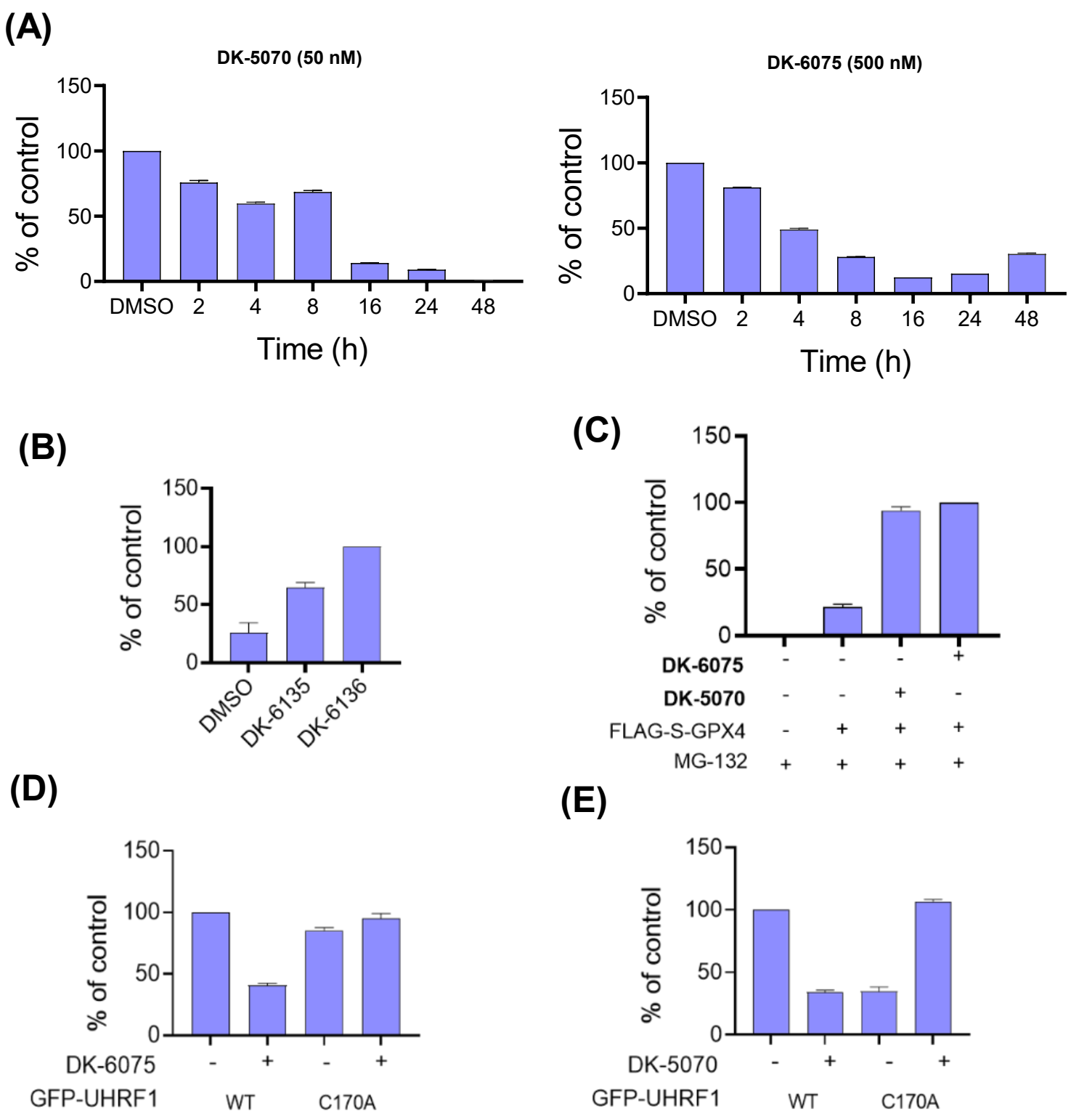

Figure S6. (**A)** Densitometric analysis of the immunoblot data shown in Figure 4F. **(B)** Densitometric analysis of the immunoblot data shown in Figure 5H. (**C)** Densitometric analysis of the immunoblot data shown in Figure 5I. (**D) (E)** Densitometric analysis of the immunoblot data shown in Figure 6D/E.

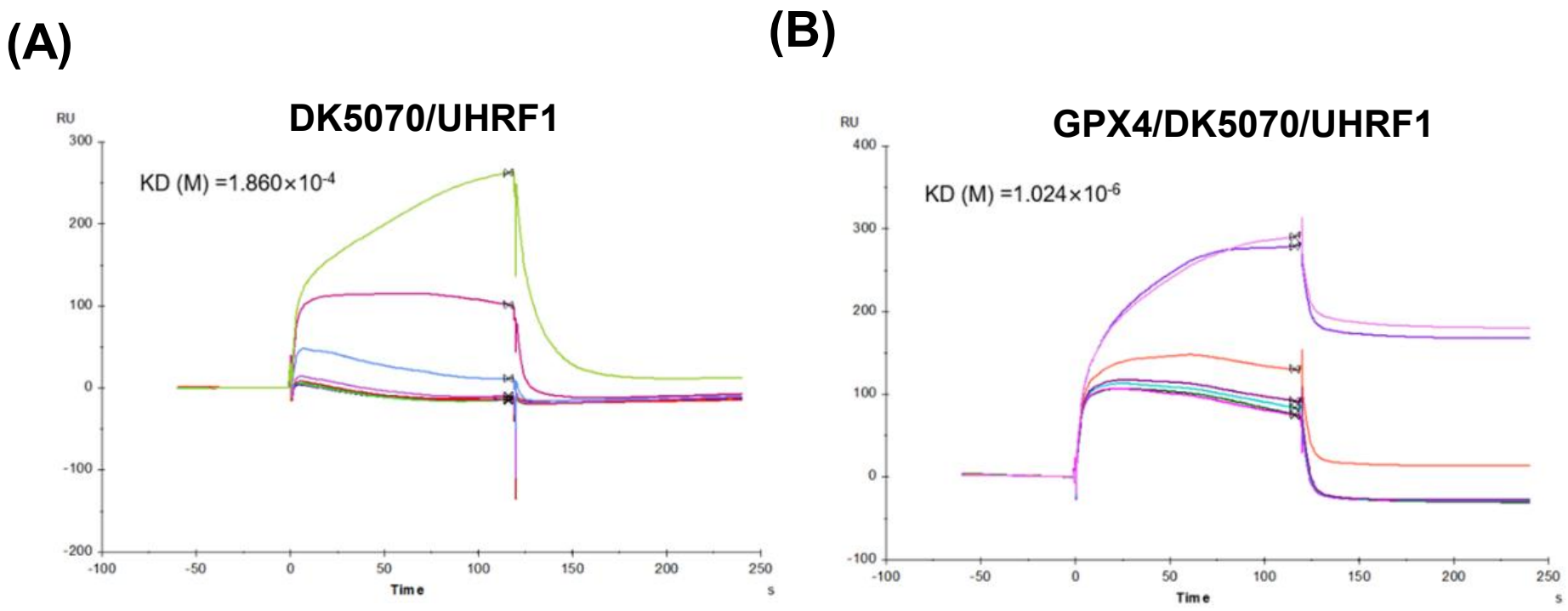

Figure S7. (A) Surface plasmon resonance (SPR) experiments showed that **DK-5070** binds to recombinant UHRF1 protein in a concentration-dependent manner. (B) **DK-5070** enhances the interaction between GPX4 and UHRF1.

Table S1. Comparison of Docking Scores and Binding Free Energies

Compound	GScore (kcal/mol)	Lipo (kcal/mol)	HBond (kcal/mol)	MMGBSA ΔG (kcal/mol)
**DK-5070**	-6.92	-2.439	-0.32	-64.32
**DK-5084**	-5.96	-0.95	-0.32	-46.05

20. NMR Spectra

^{1}H of 5070

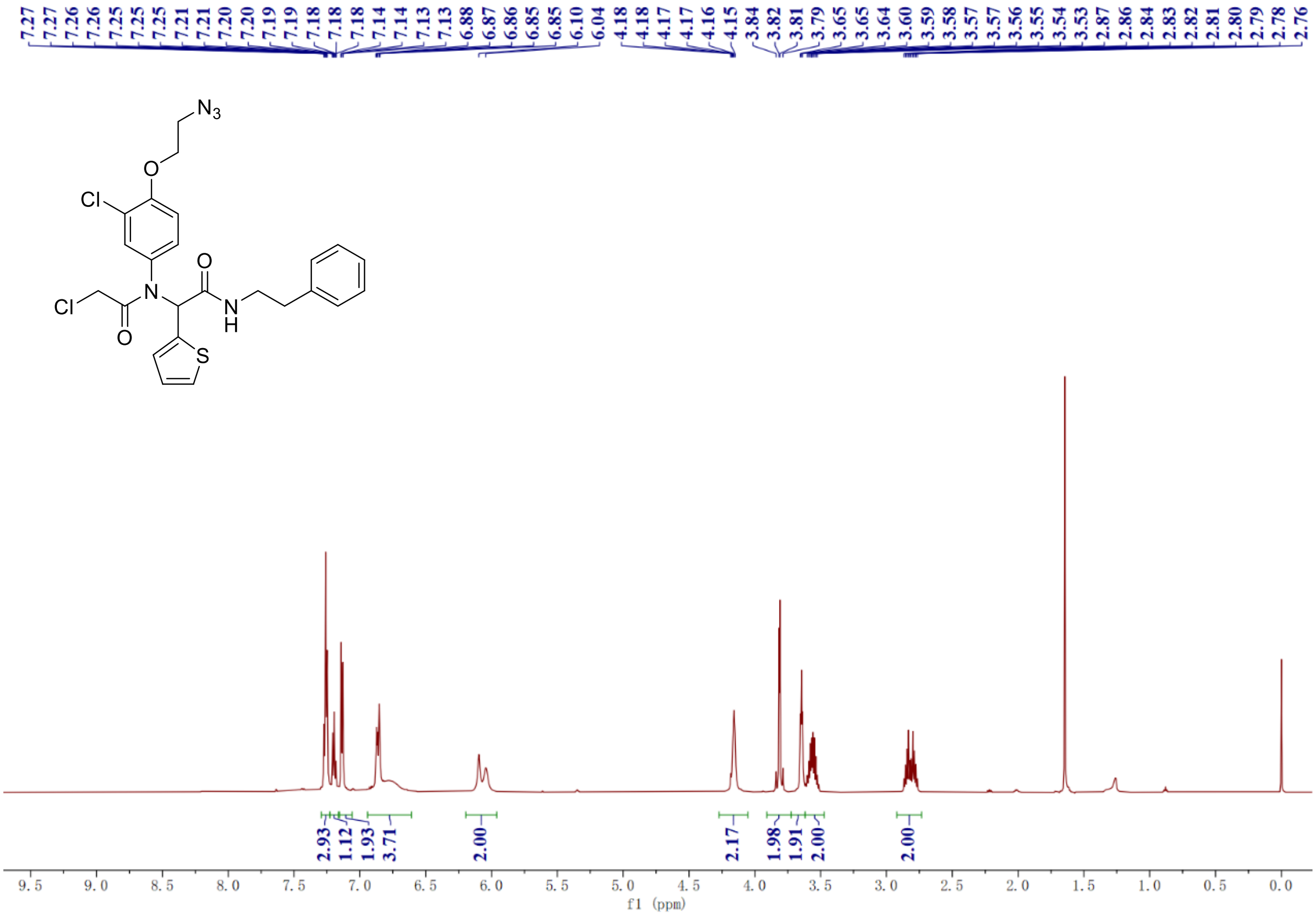

^{13}C of 5070

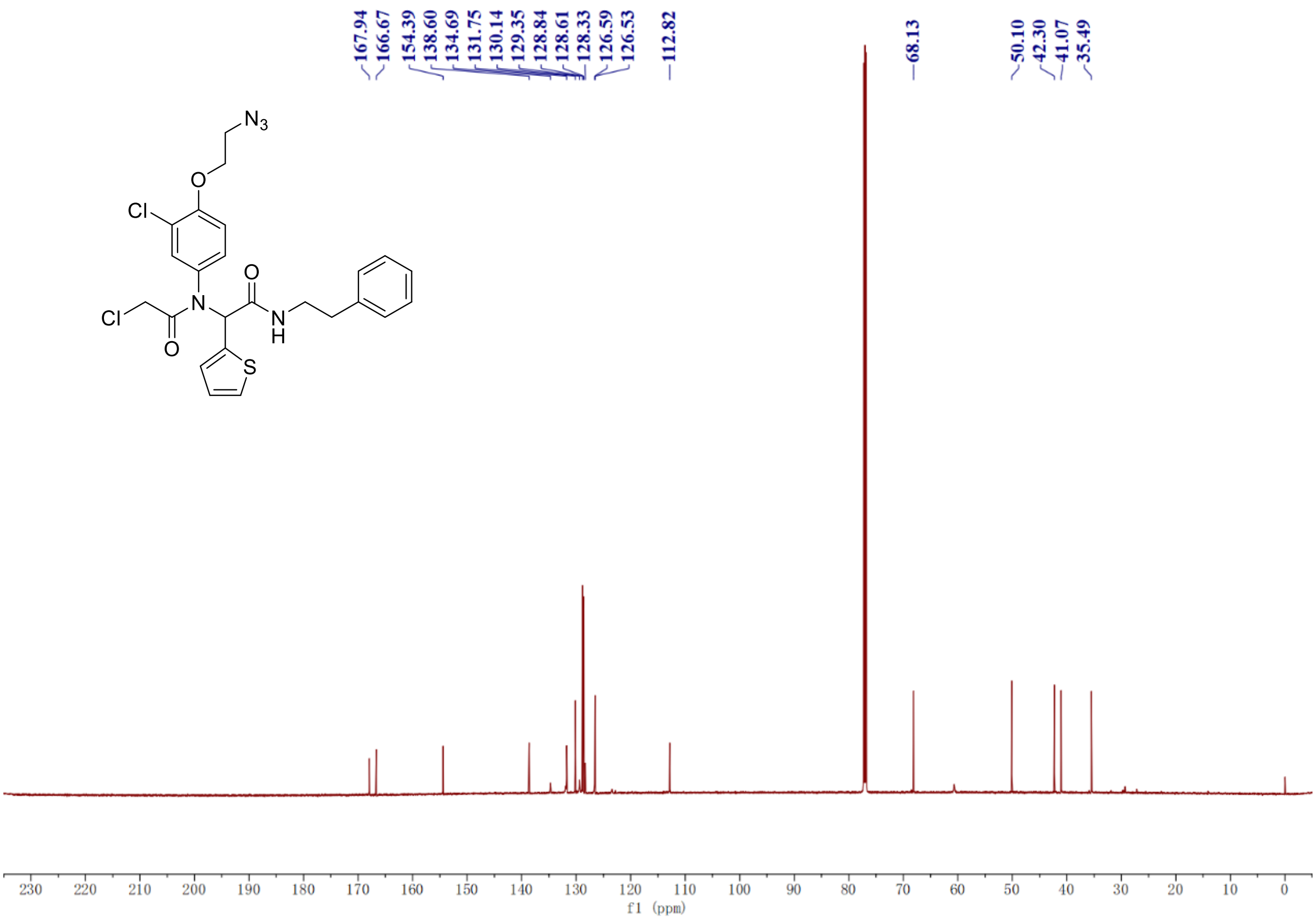

^{1}H of 6008

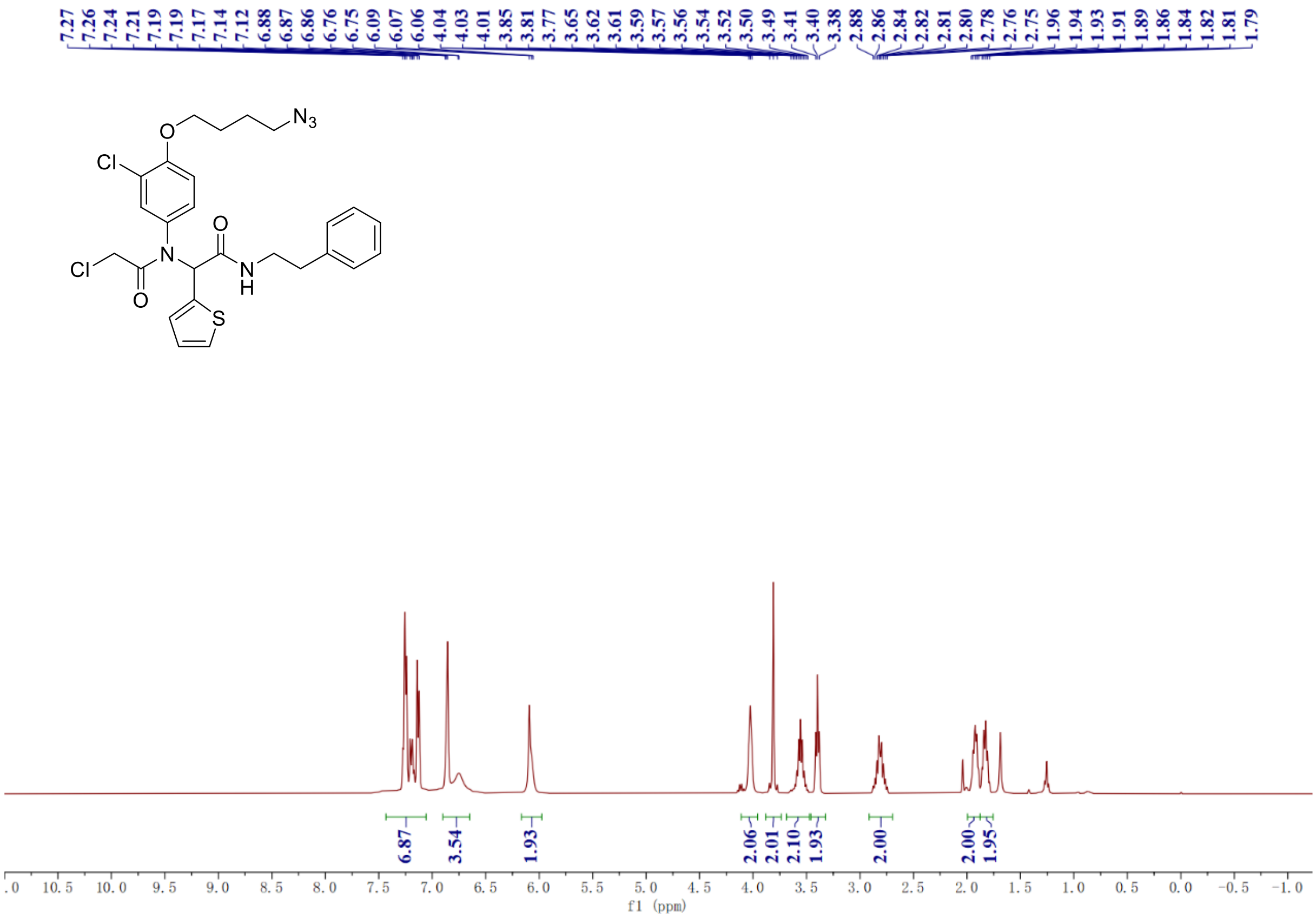

^{13}C of 6008

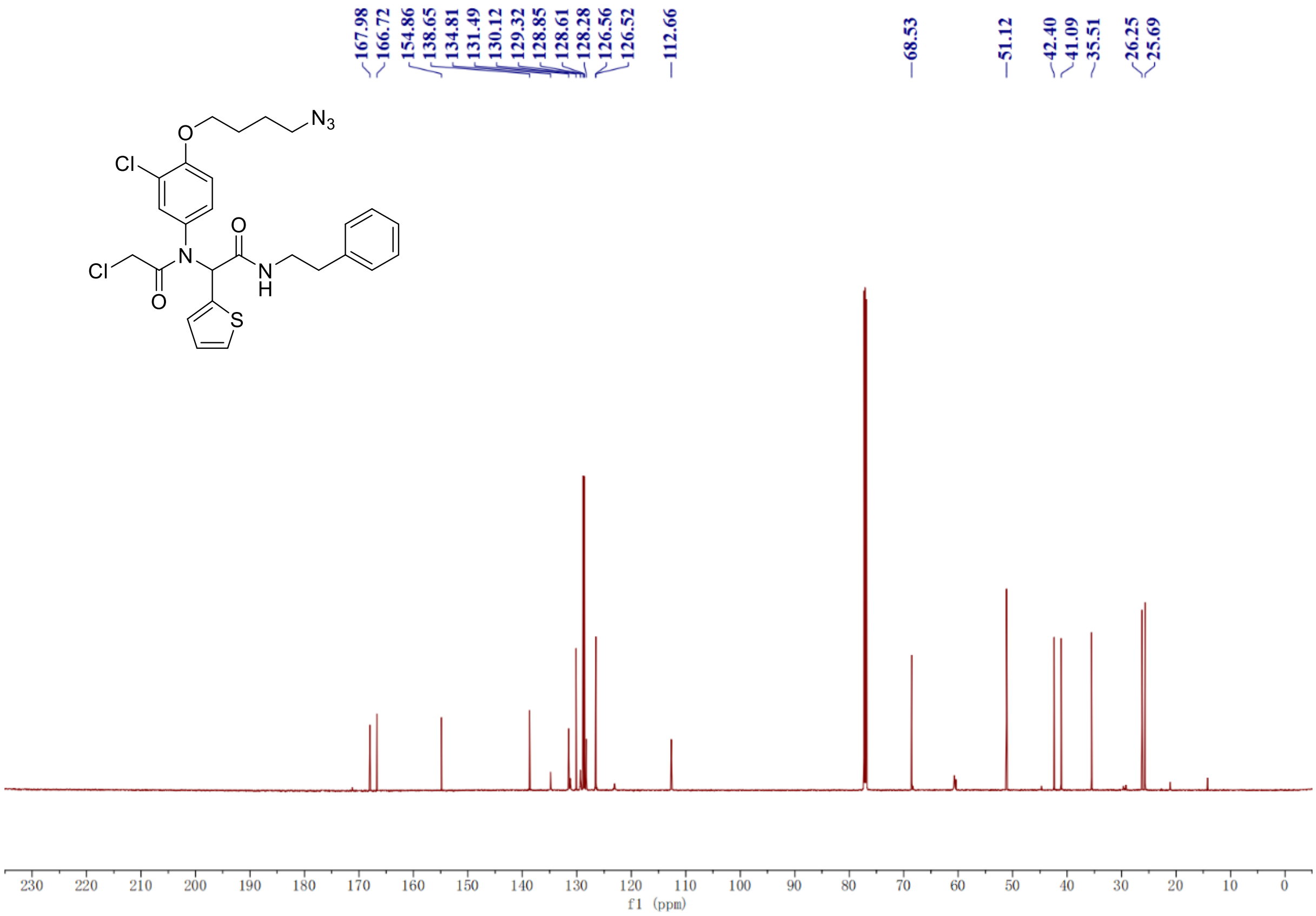

[1]H of 6055

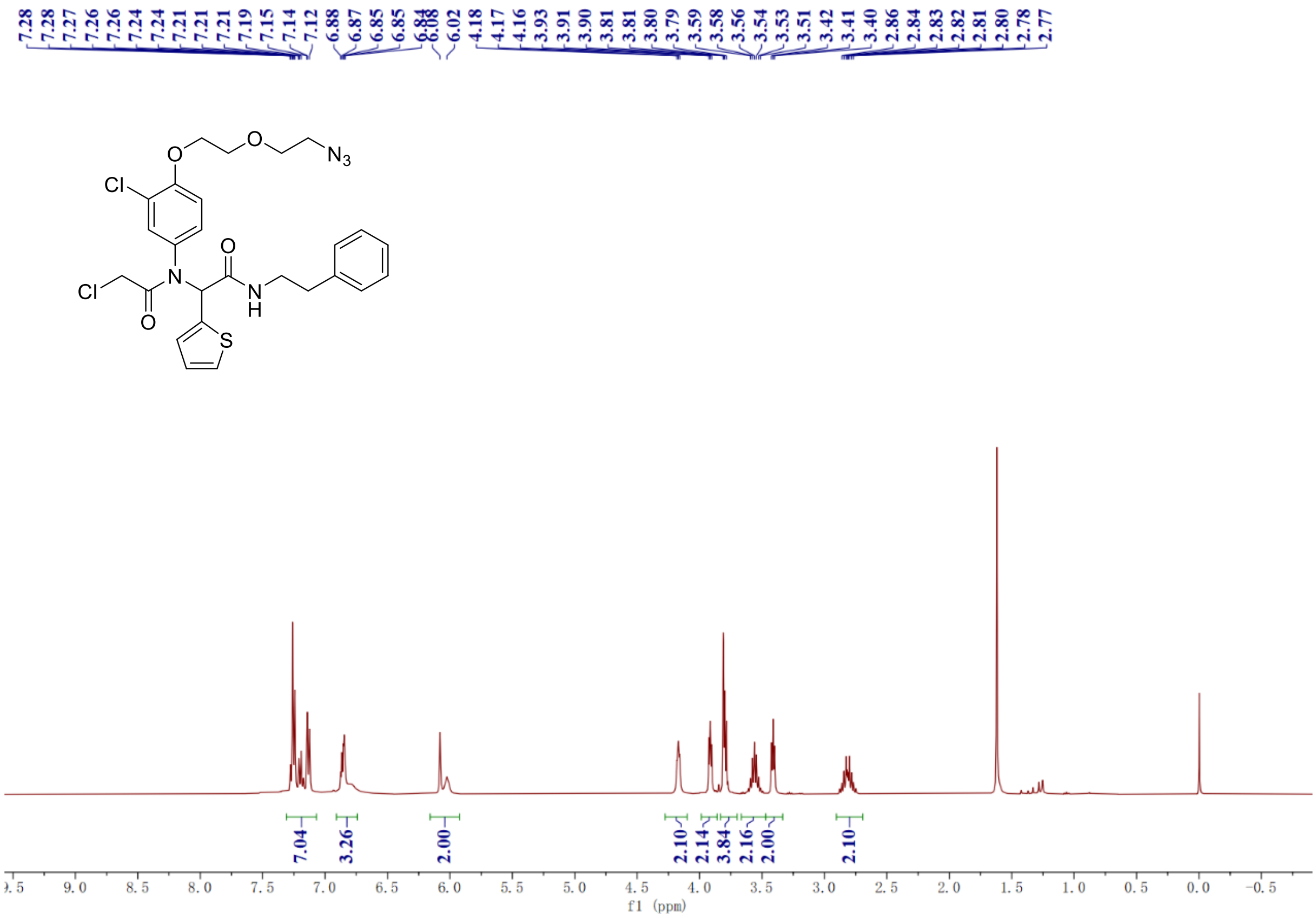

[13]C of 6055

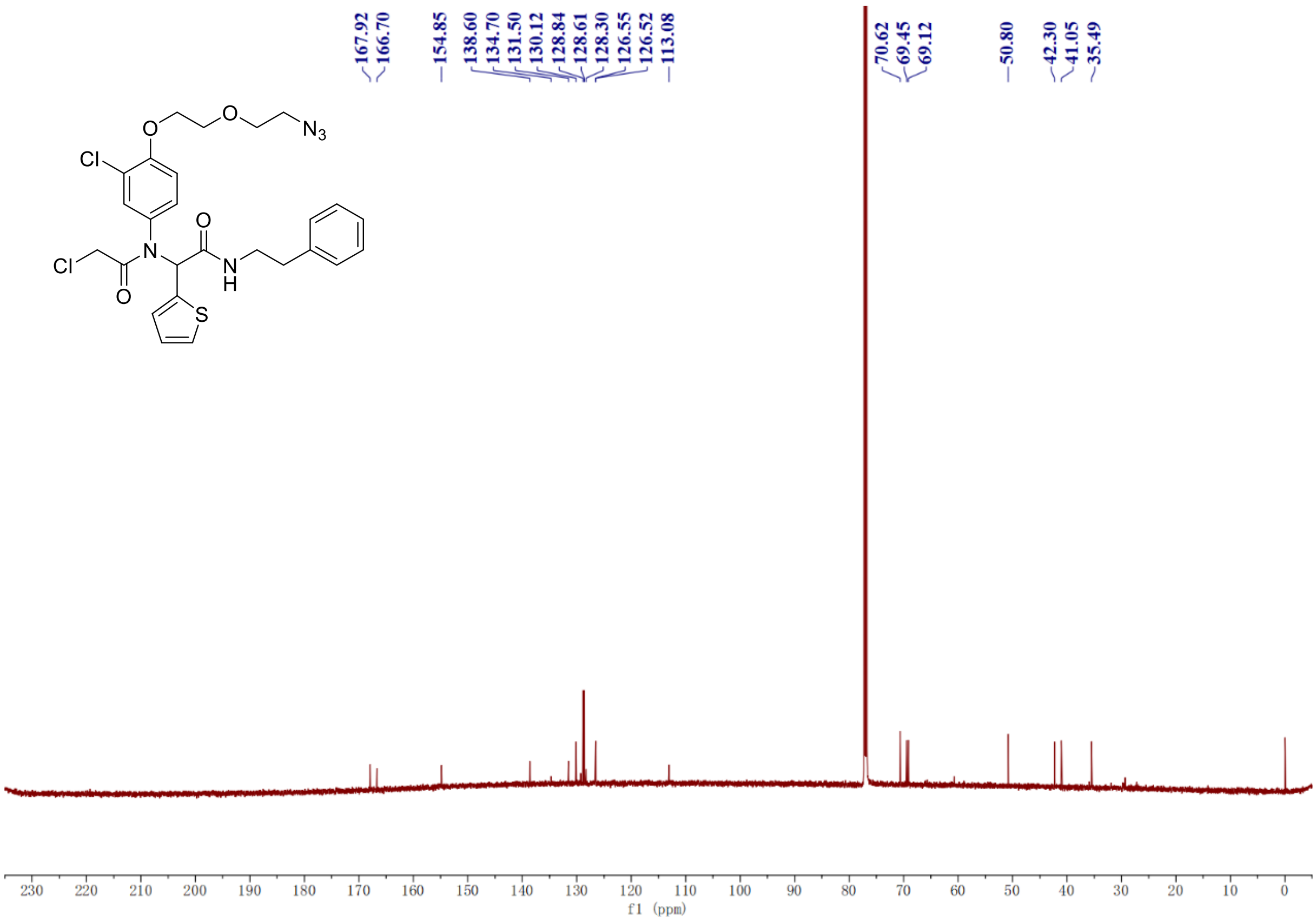

^{1}H of 6056

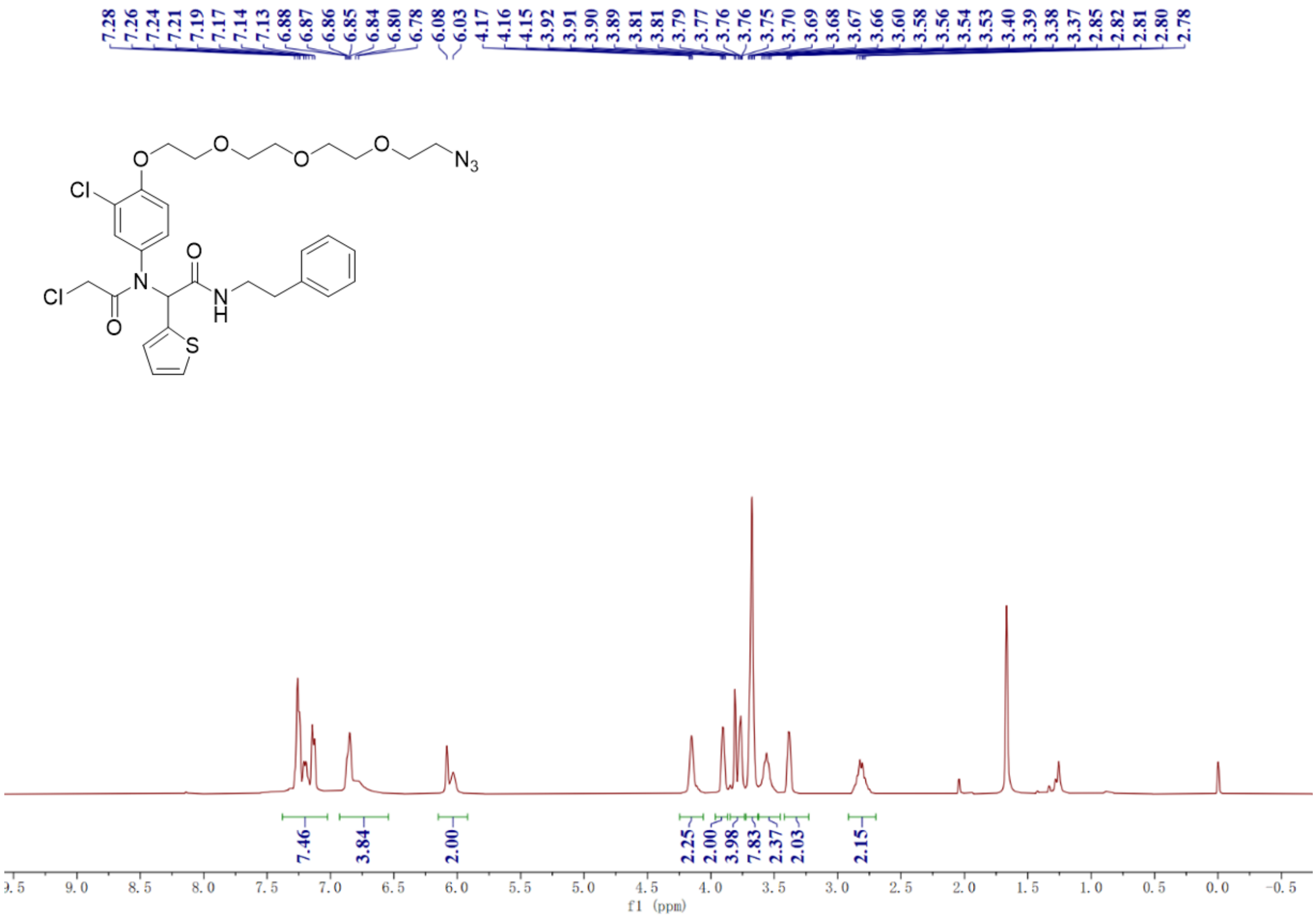

^{13}C of 6056

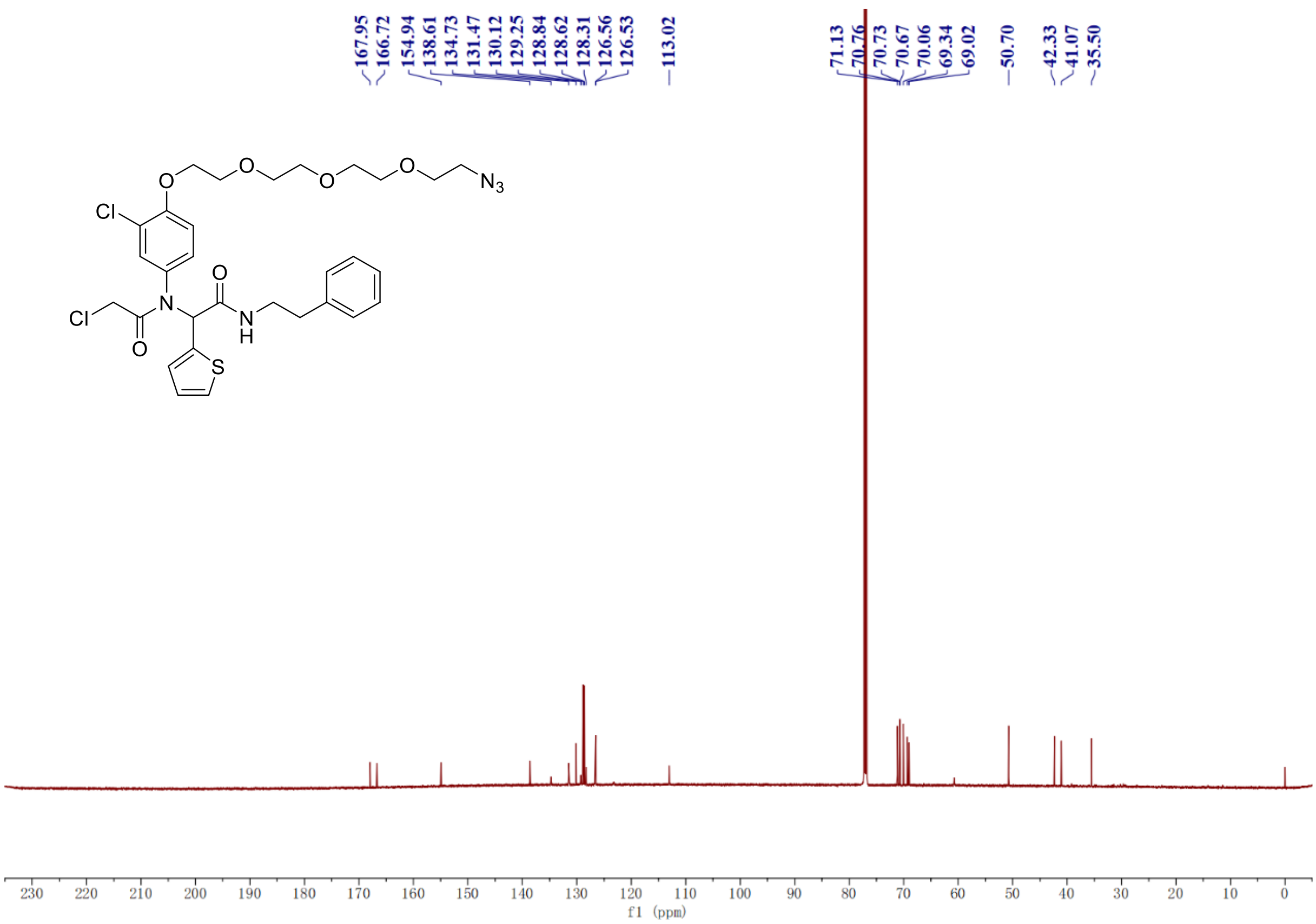

[1]H of 6106

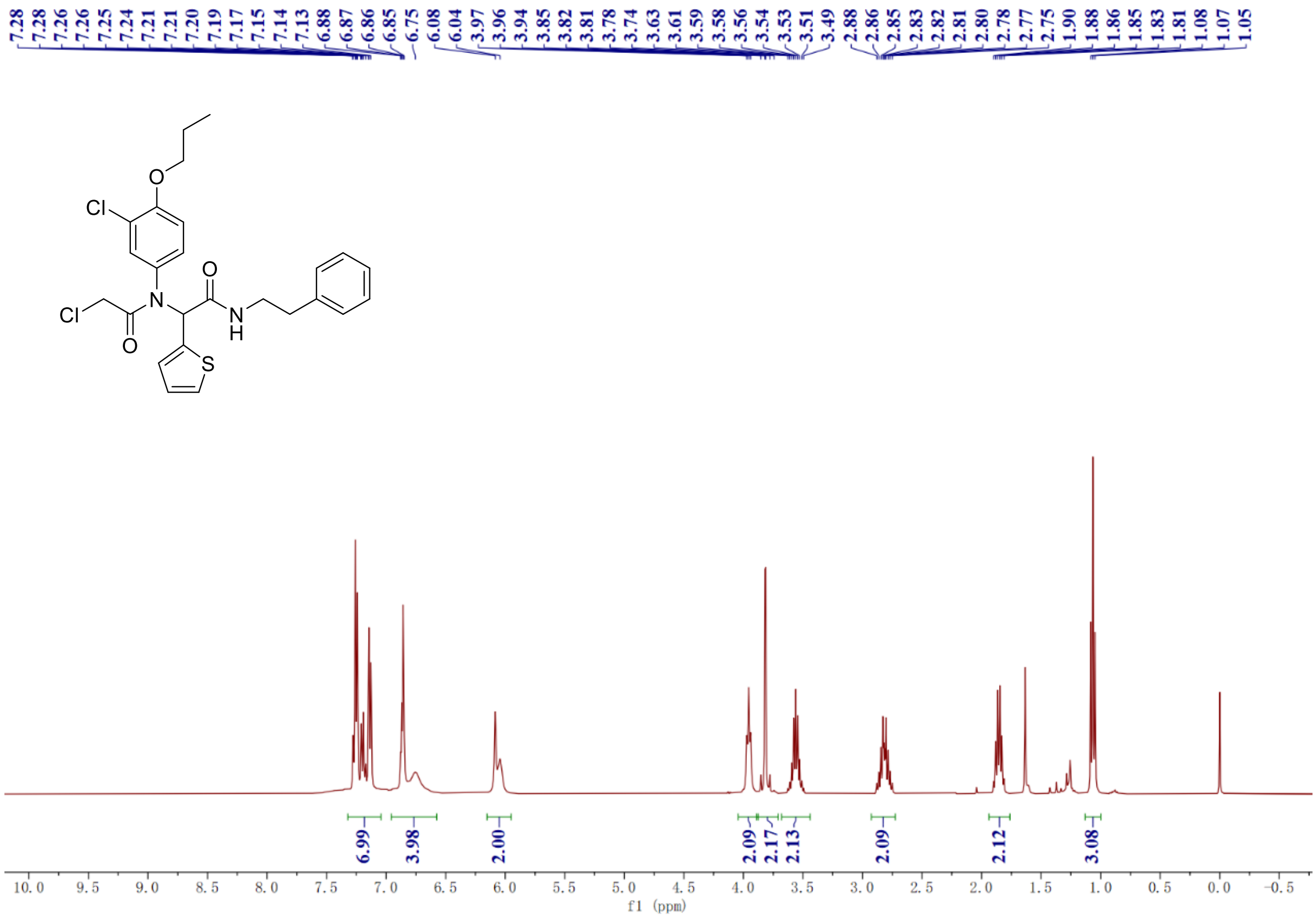

[13]C of 6106

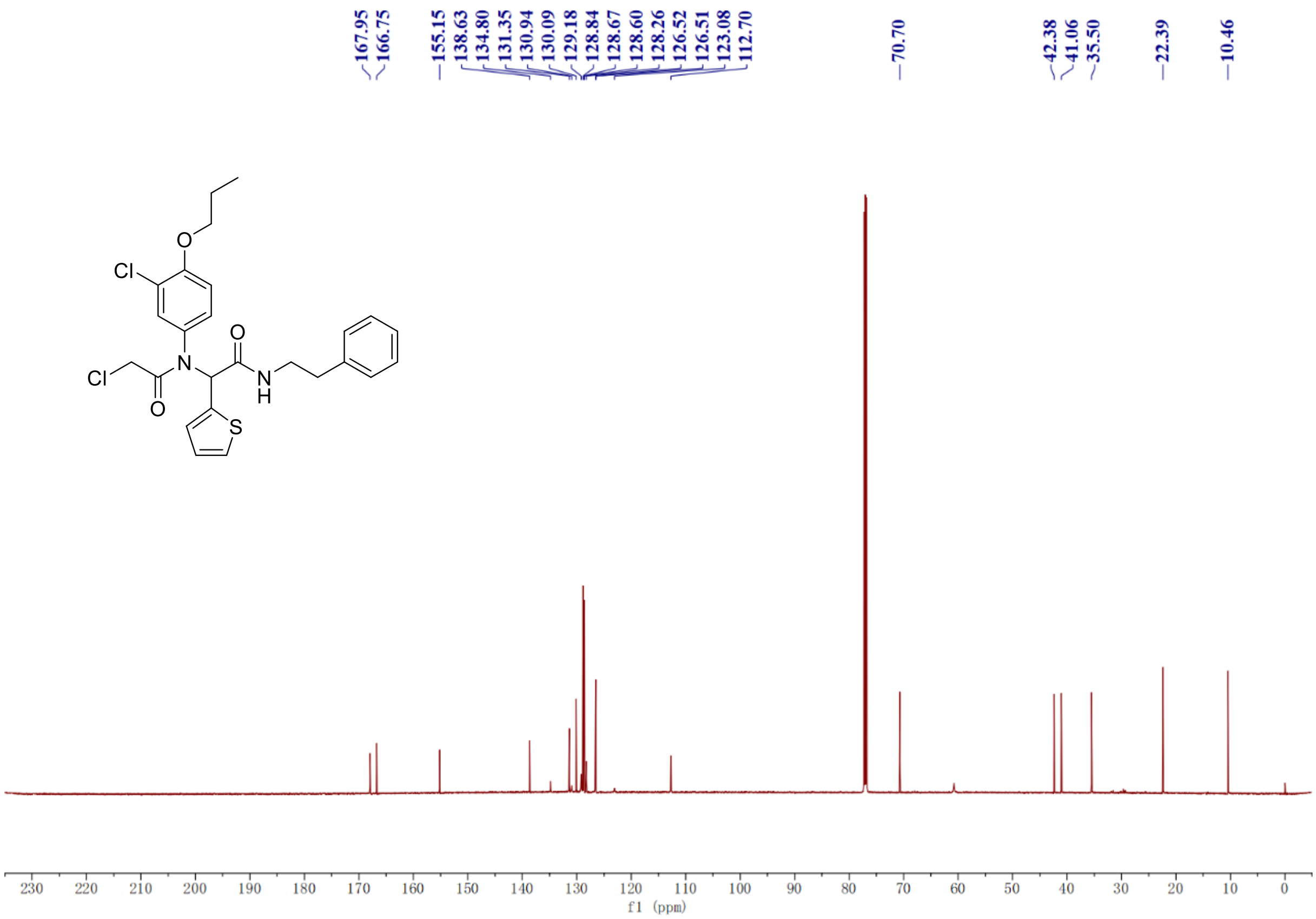

^{1}H of 6035

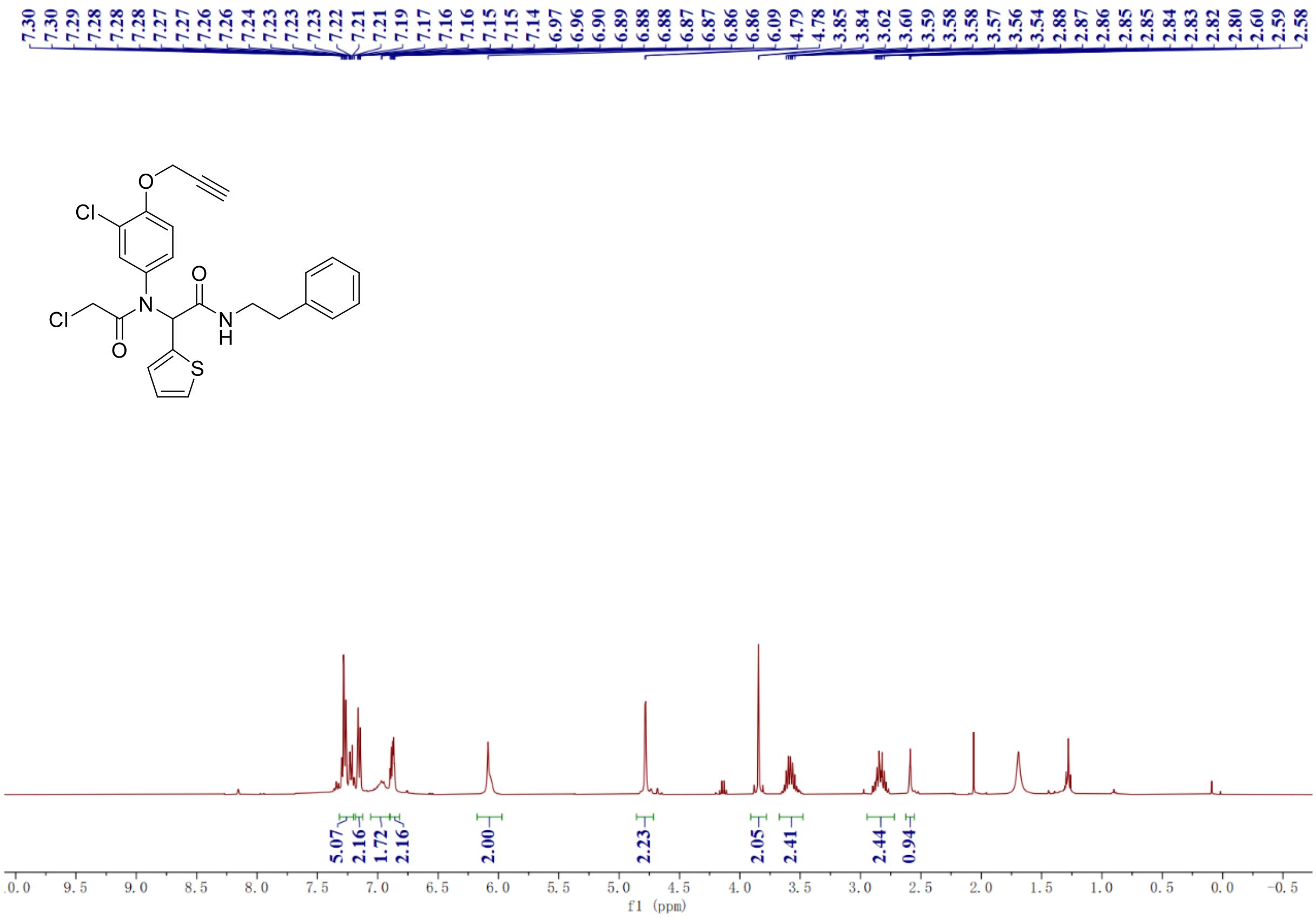

^{1}H of 6135

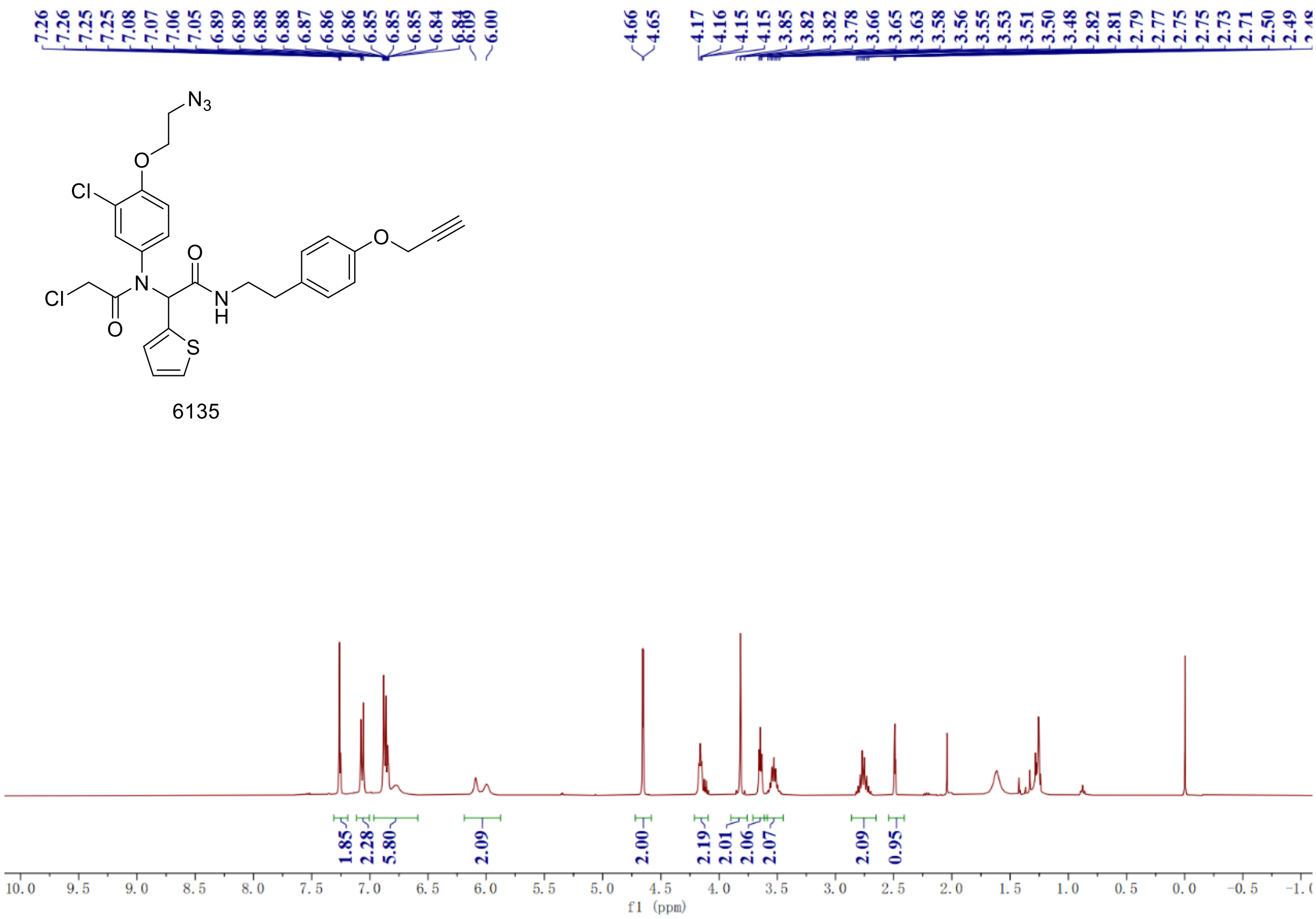

6135

^{13}C of 6135

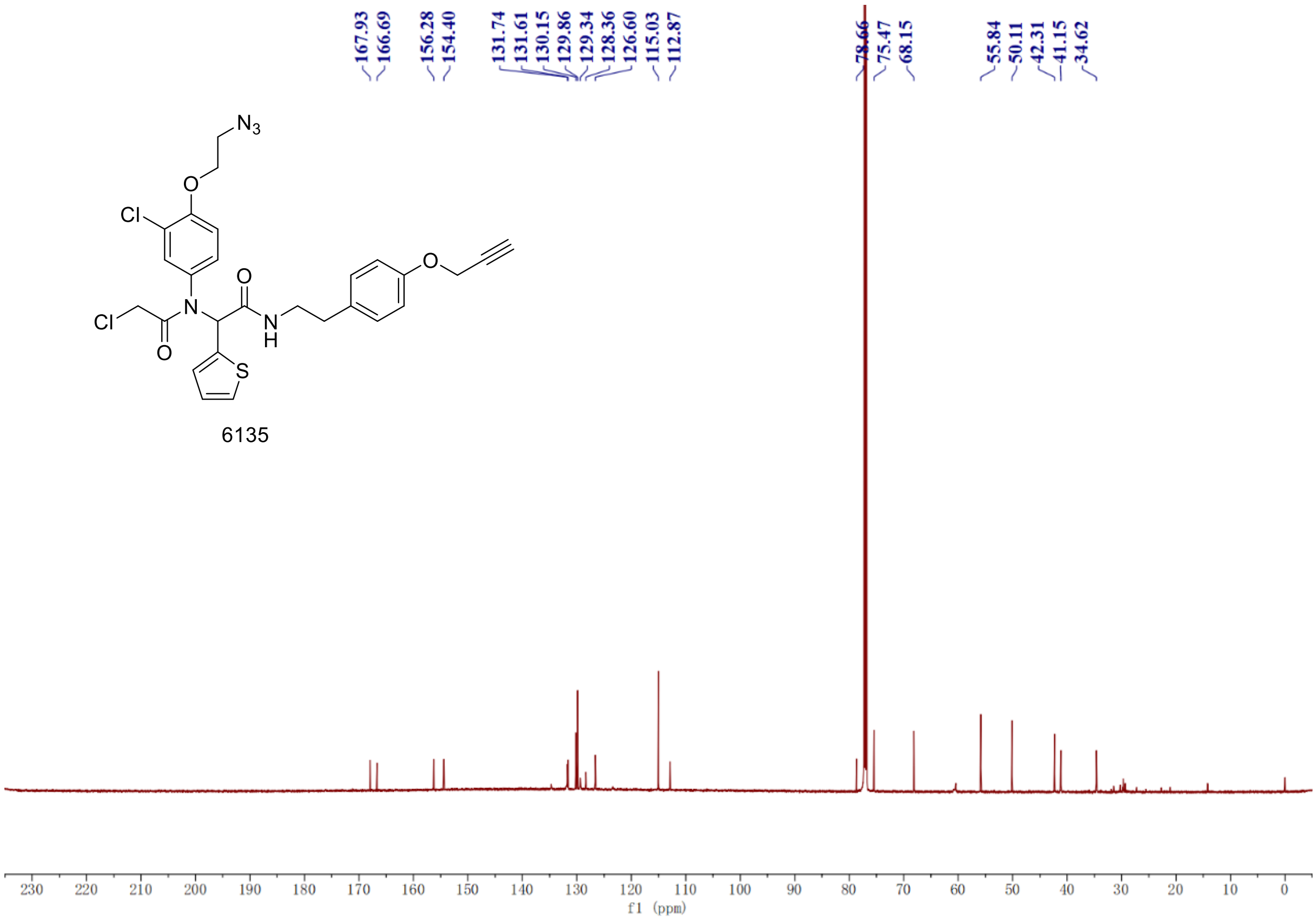

^{1}H of 5084

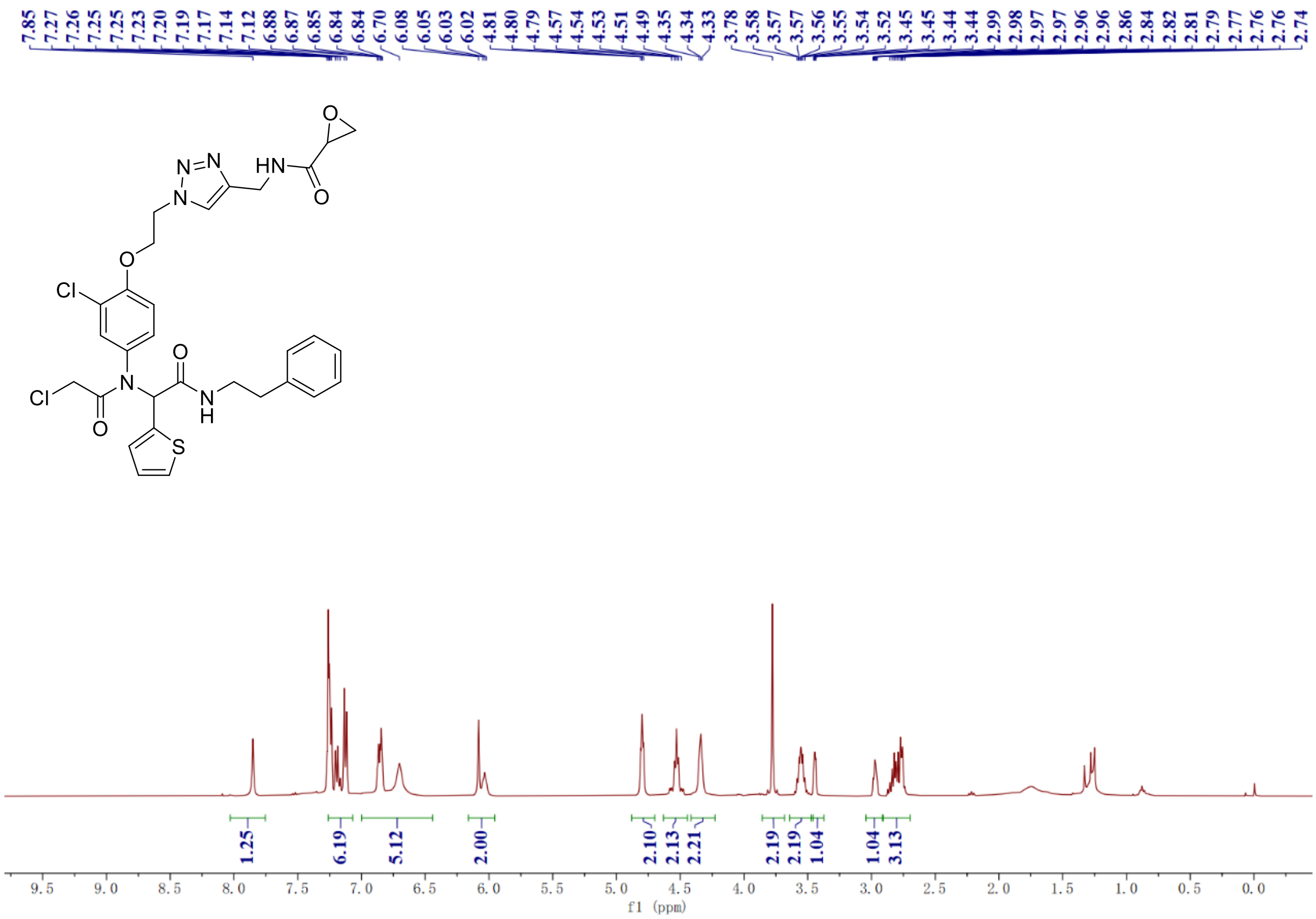

[1]H of 6075

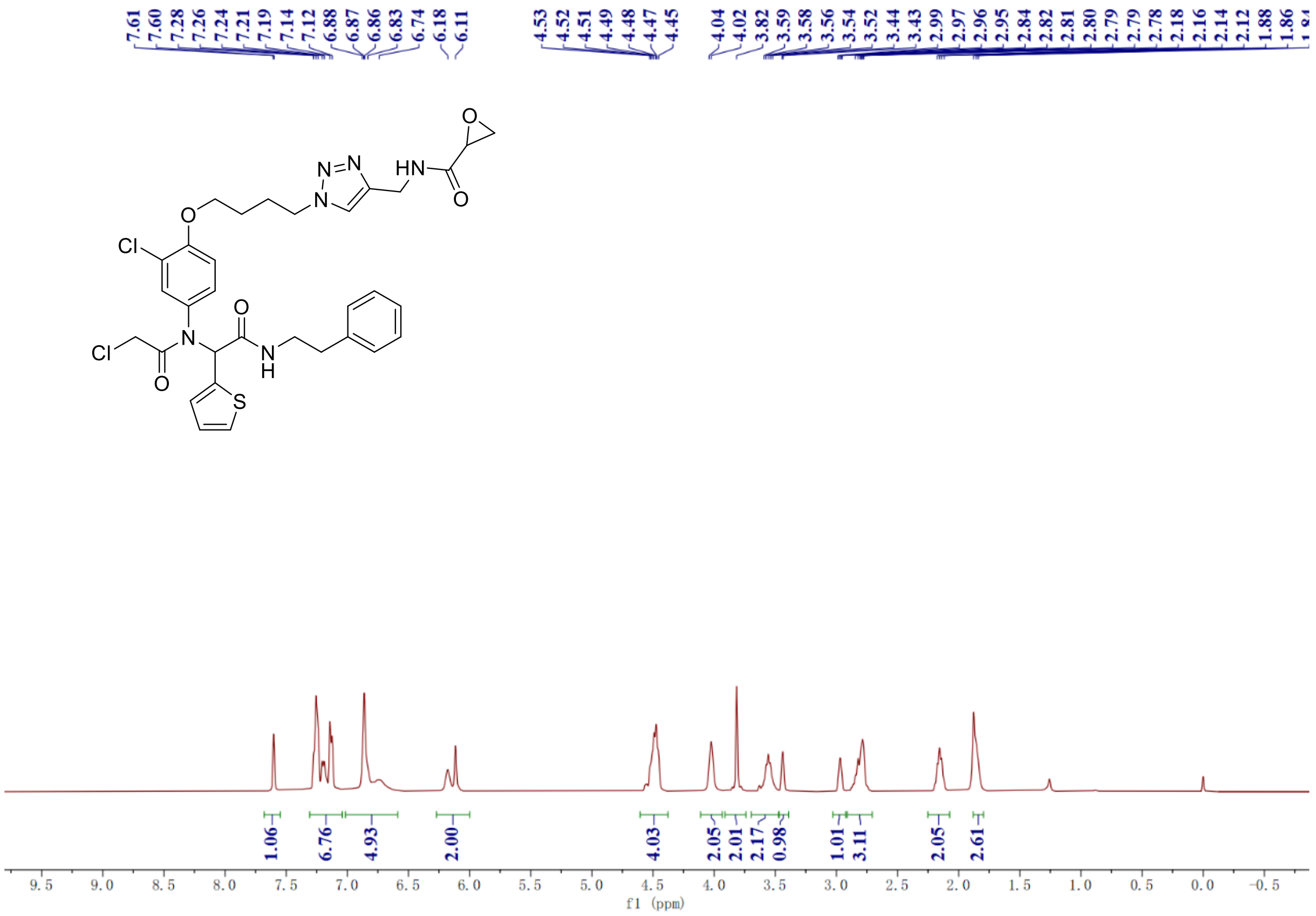

[13]C of 6075

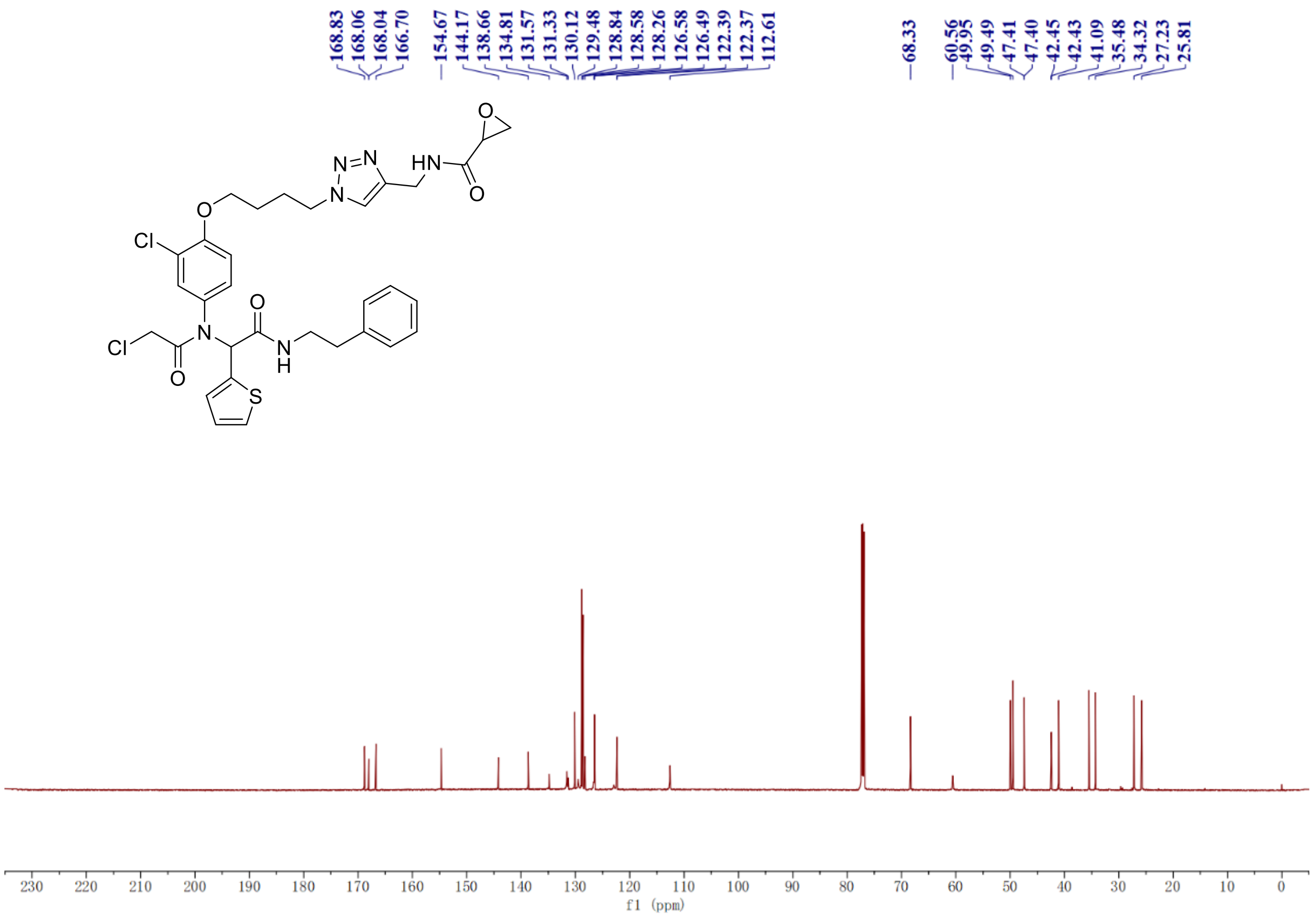

^{1}H of 6060

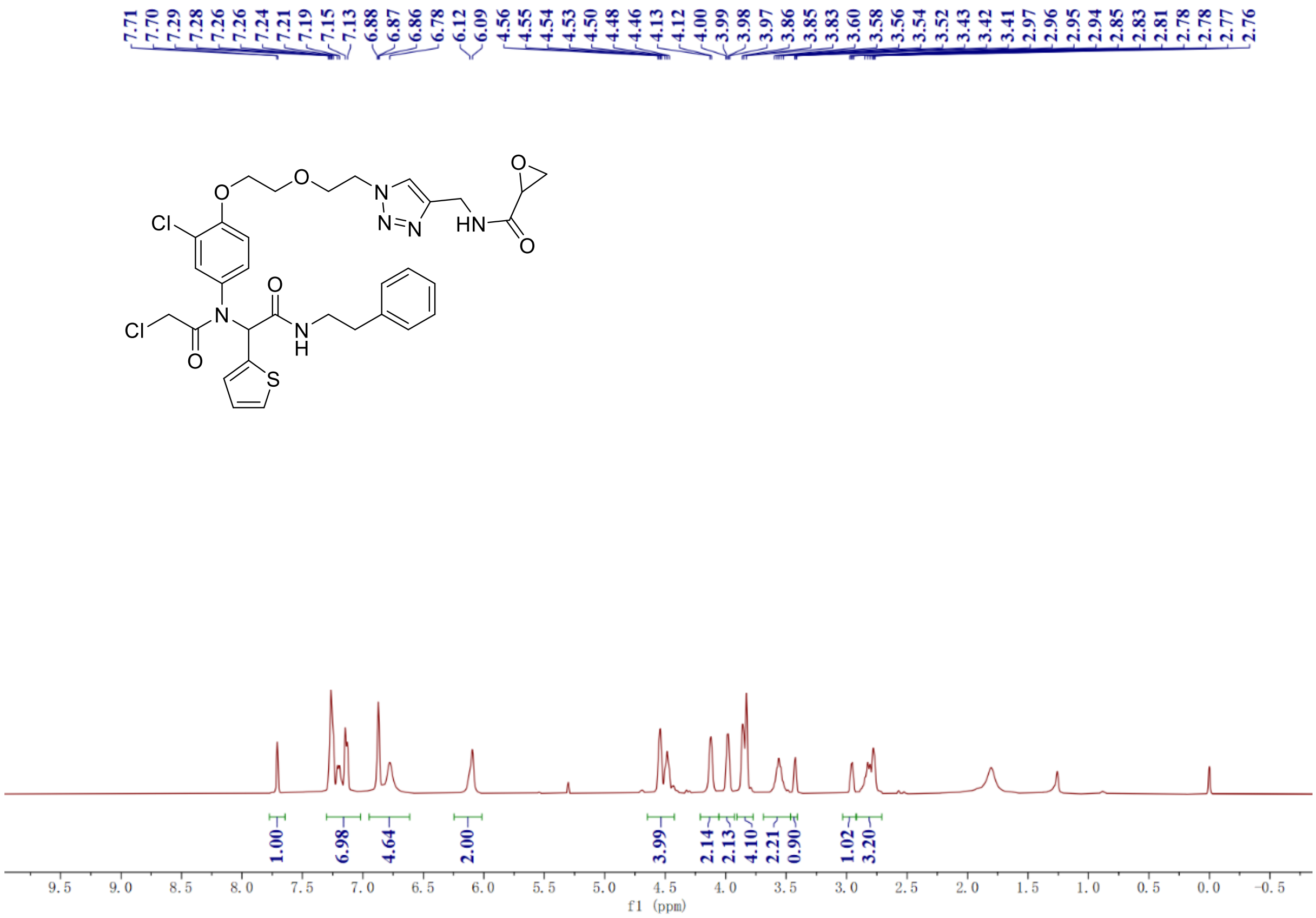

^{13}C of 6060

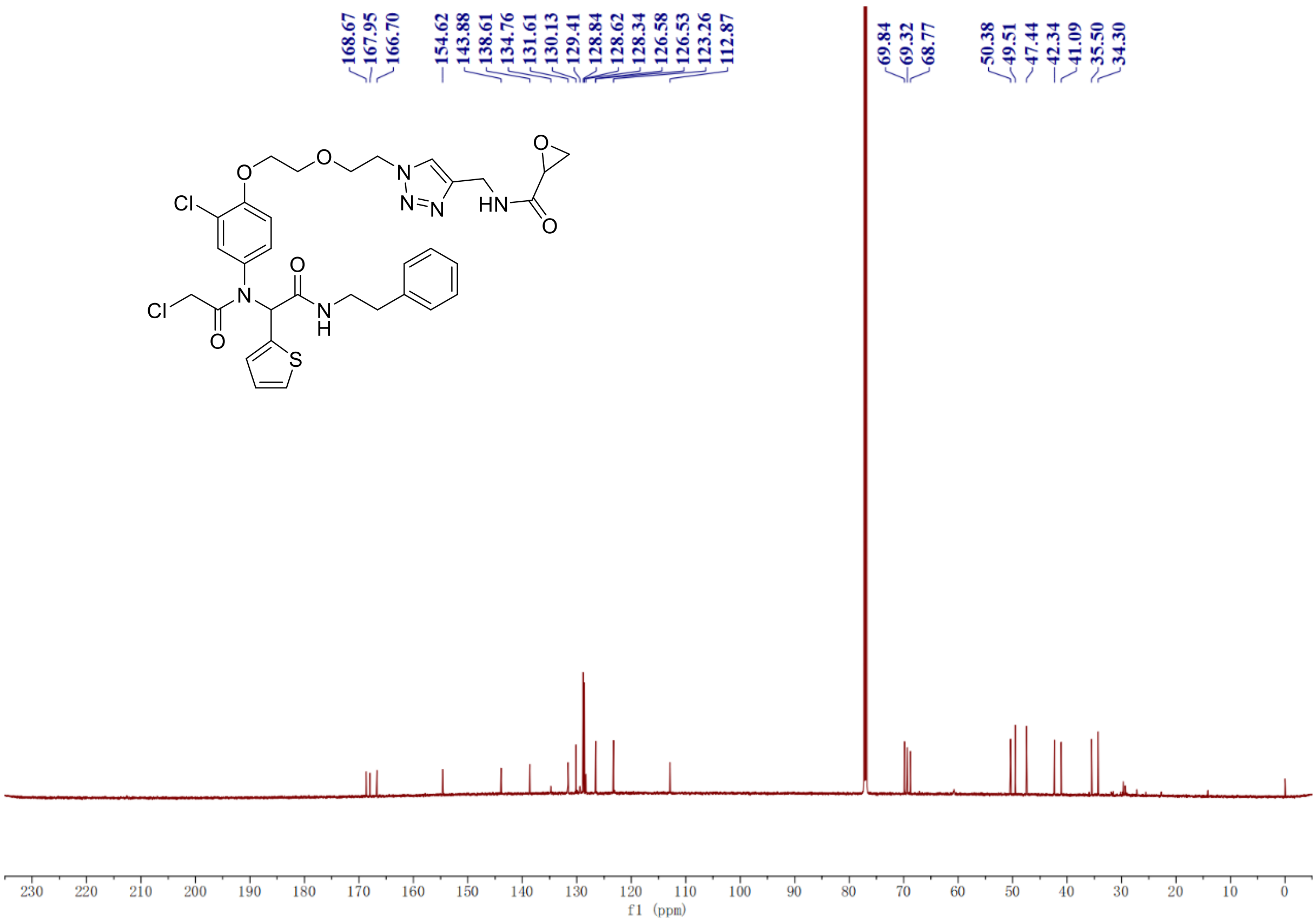

^{1}H of 6061

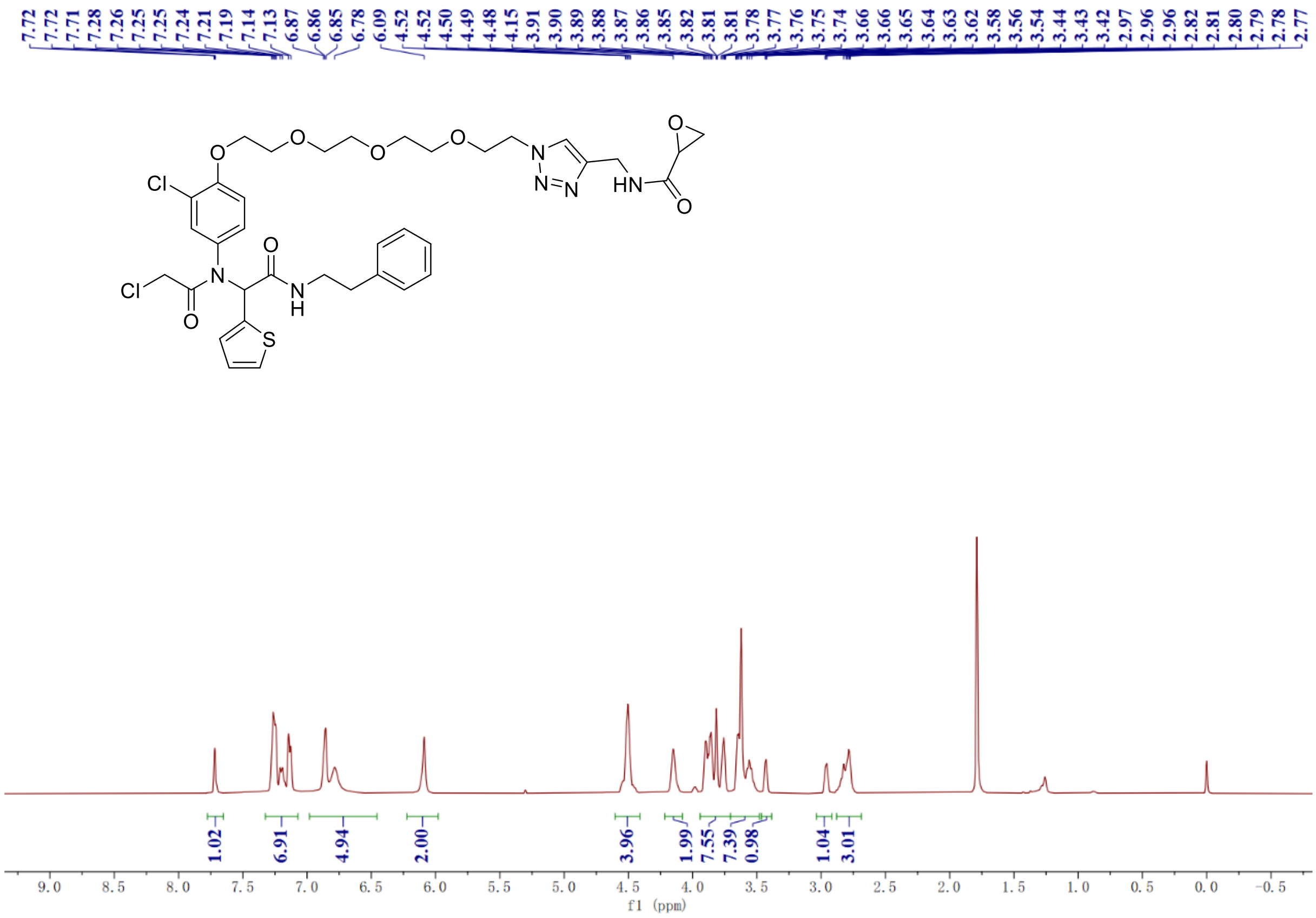

^{13}C of 6061

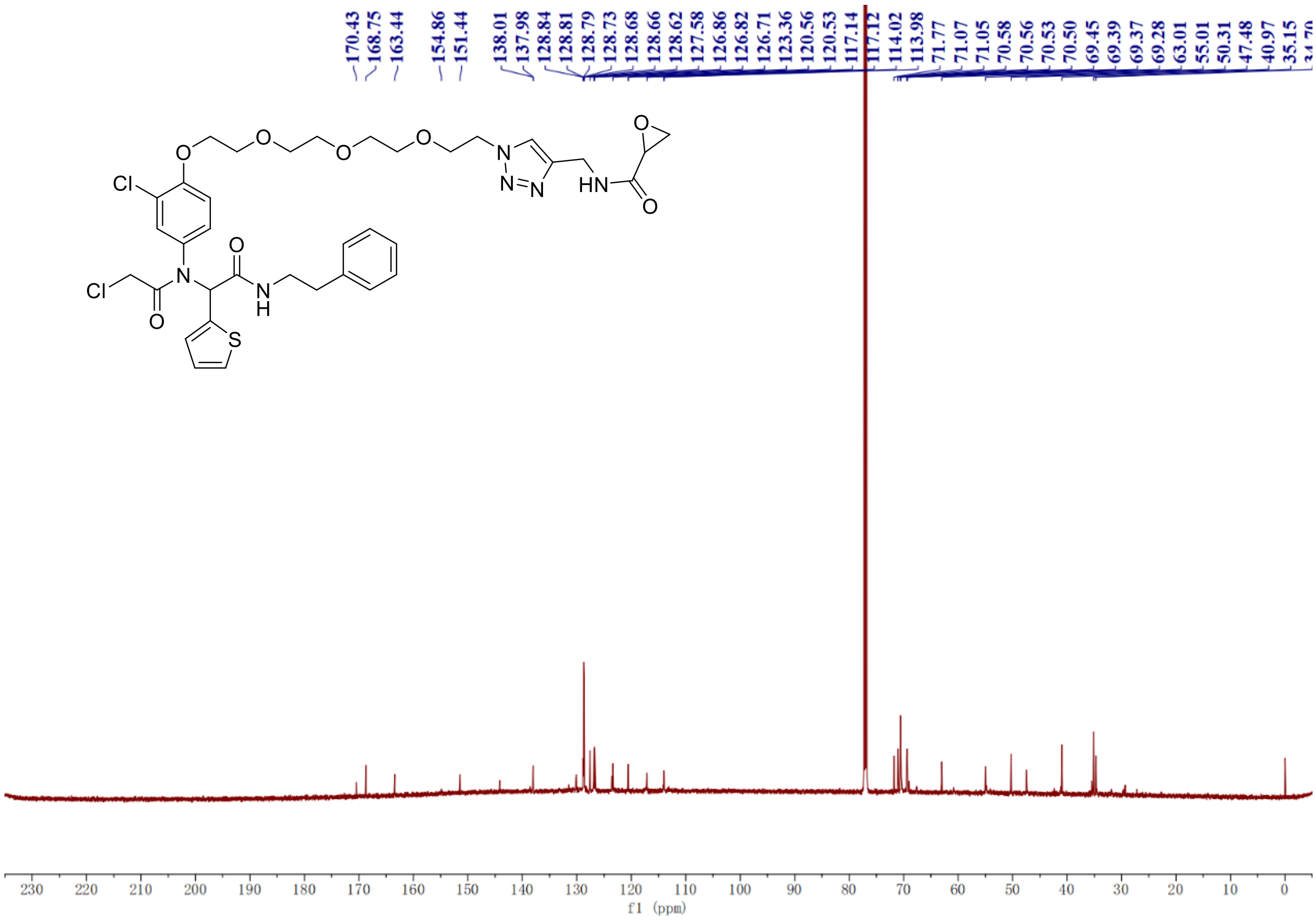

[1]H of 5071

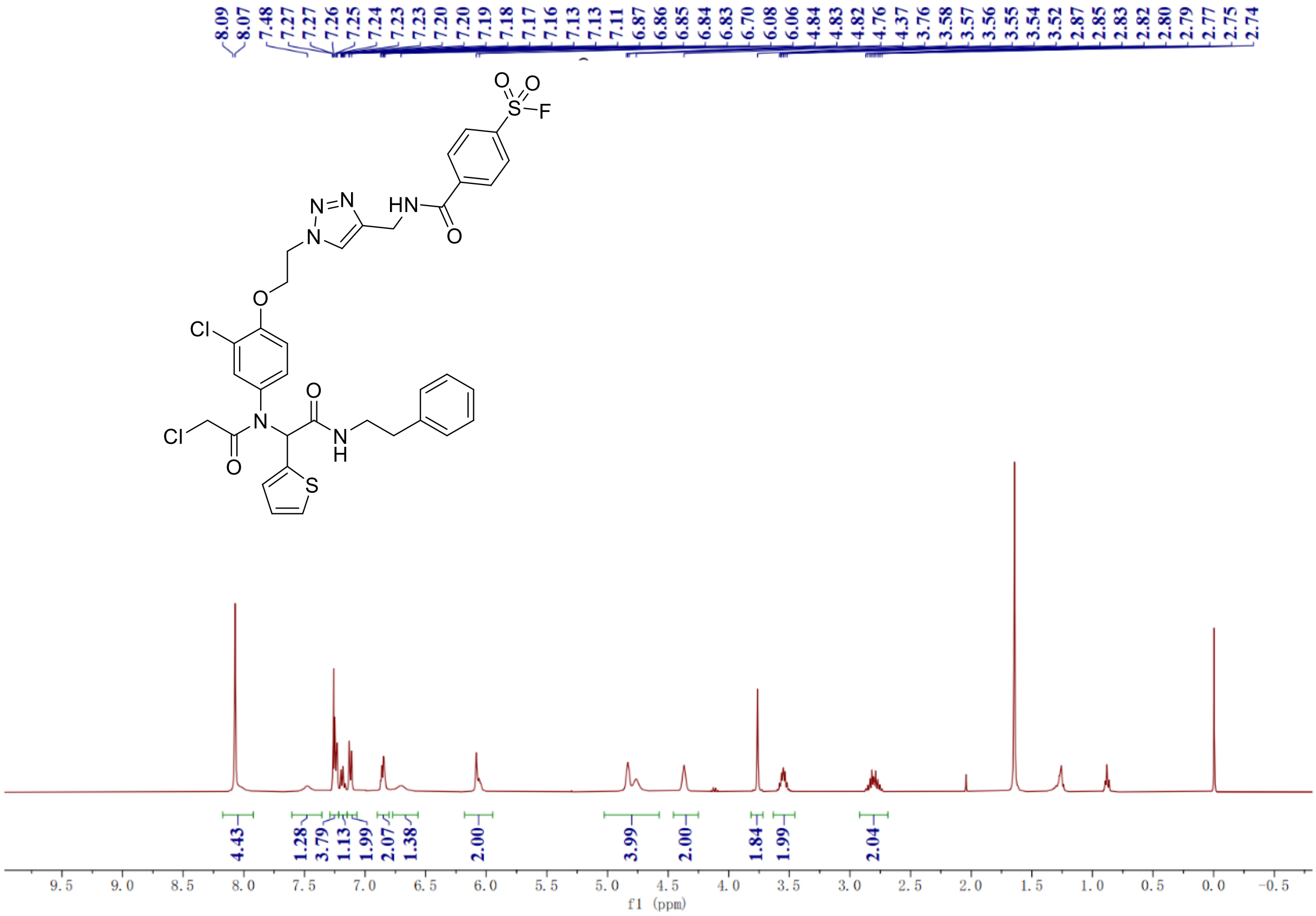

[1]H of 5072

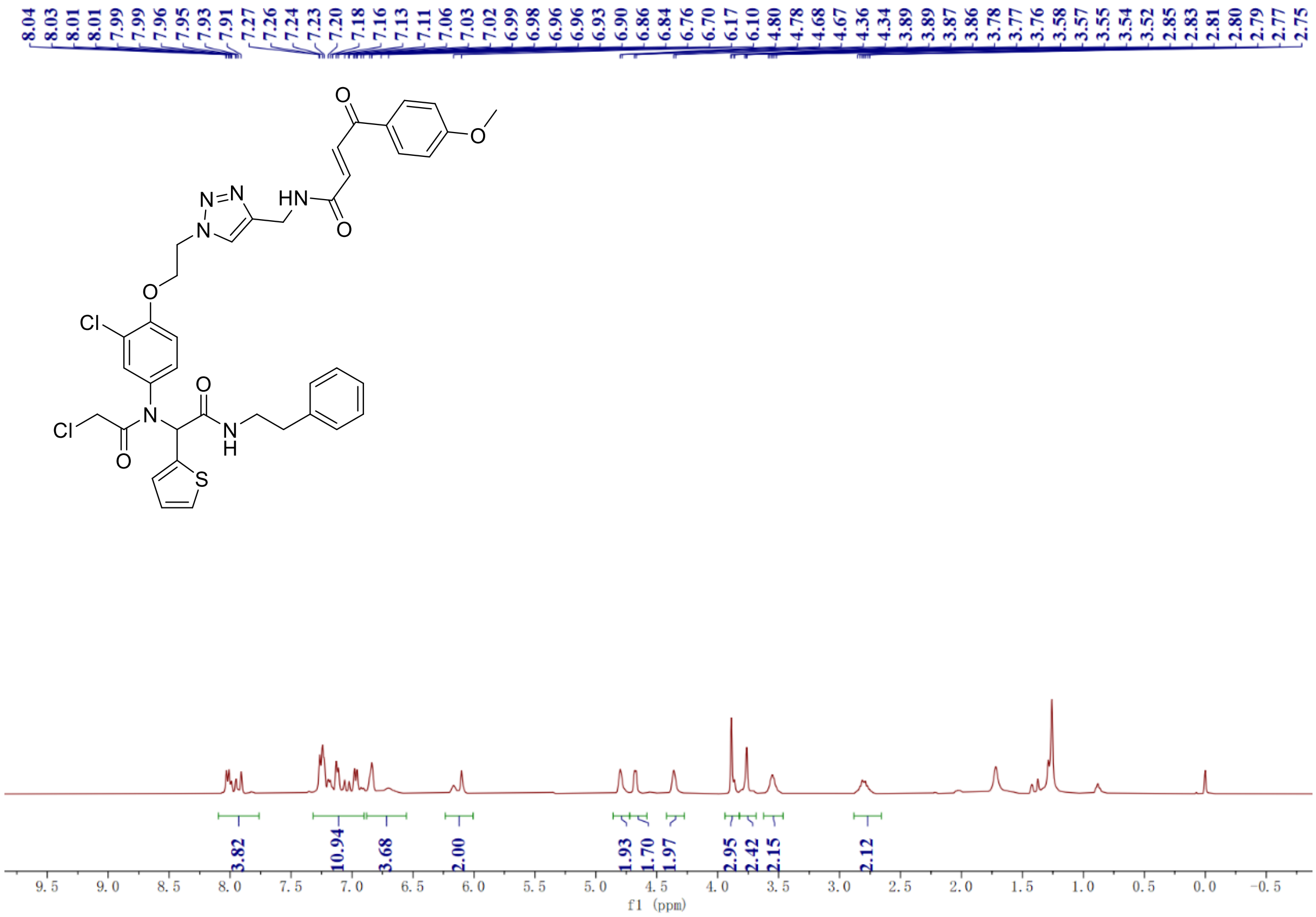

[1]H of 6076

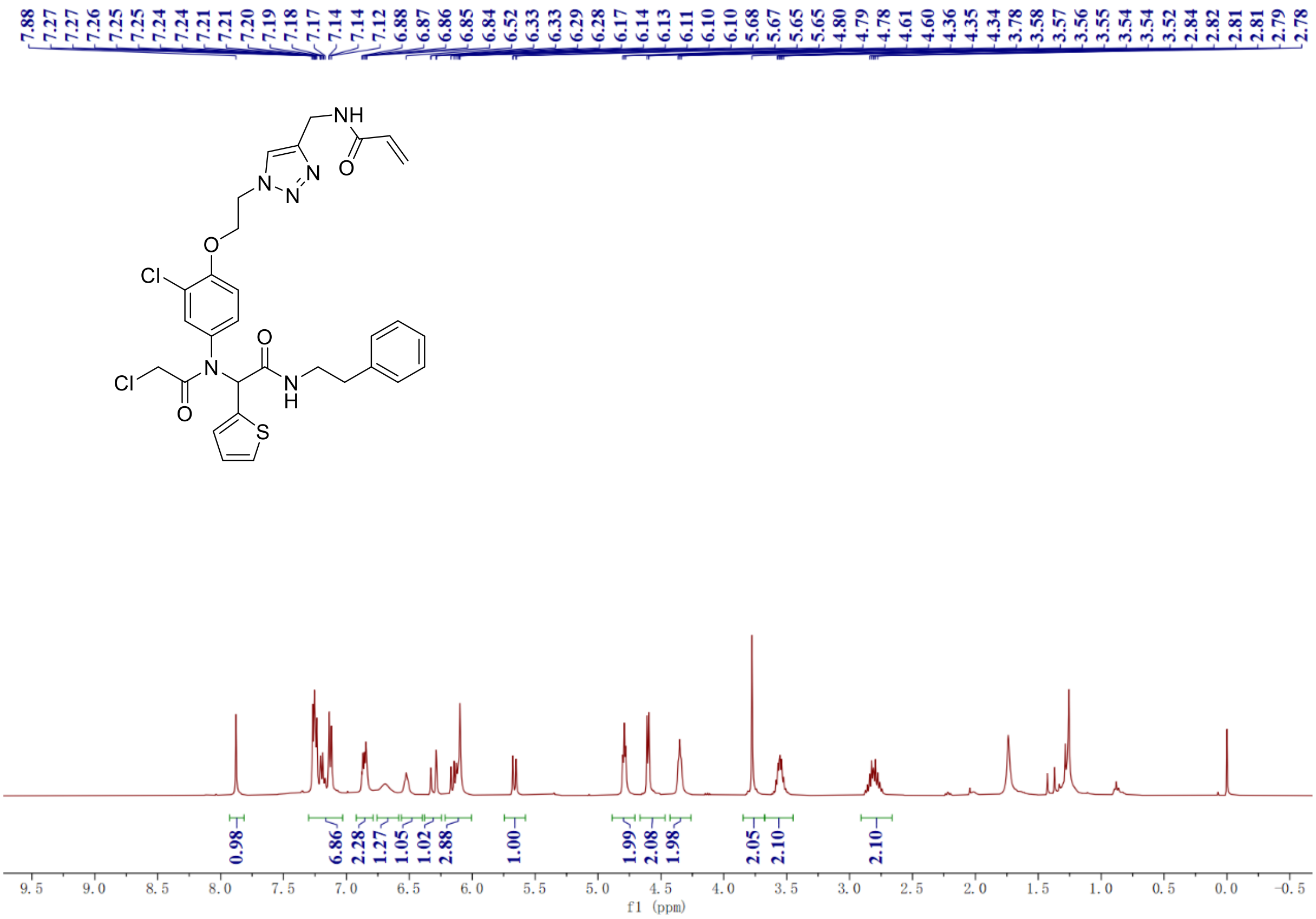

[13]C of 6076

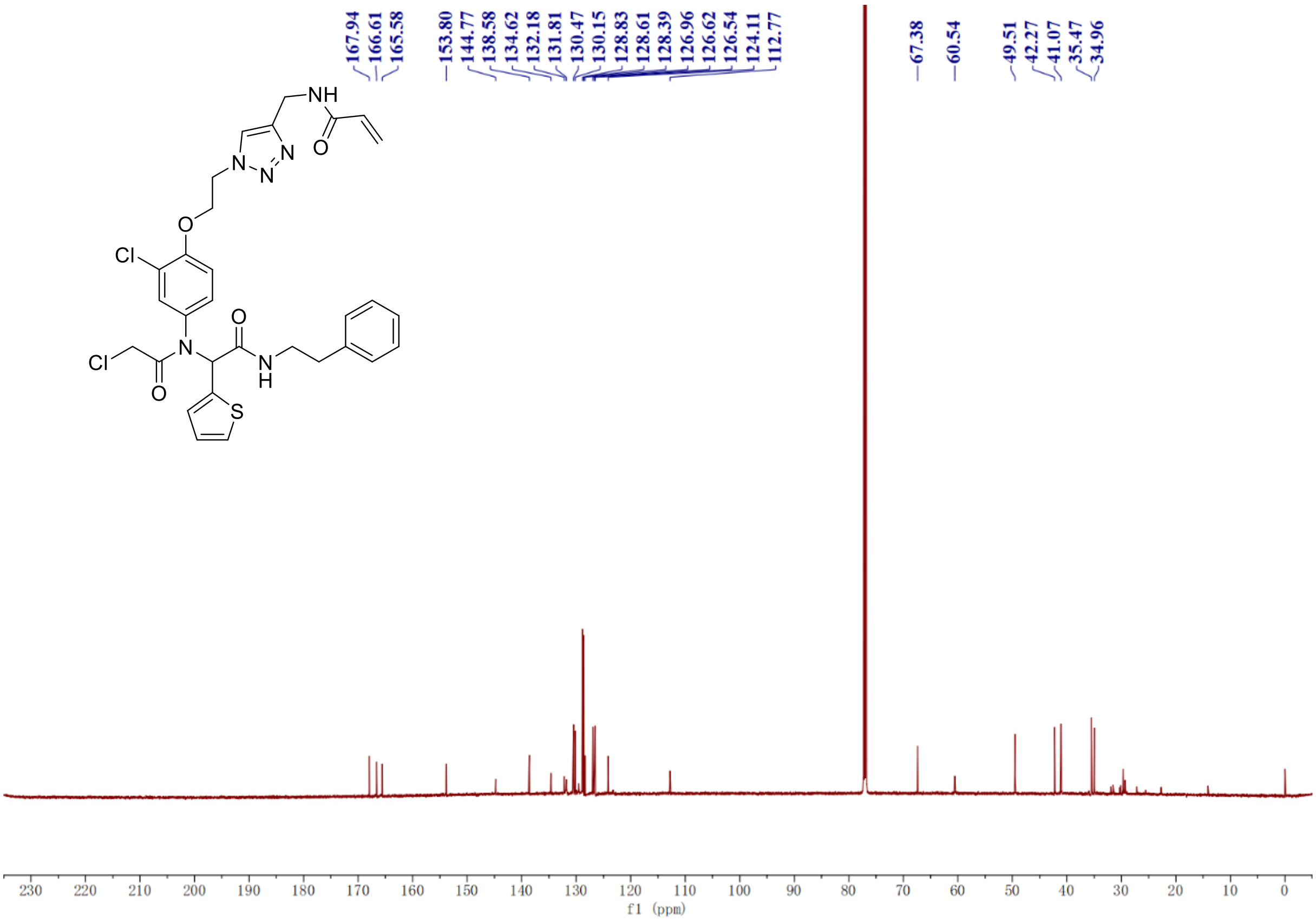

^{1}H of 6101

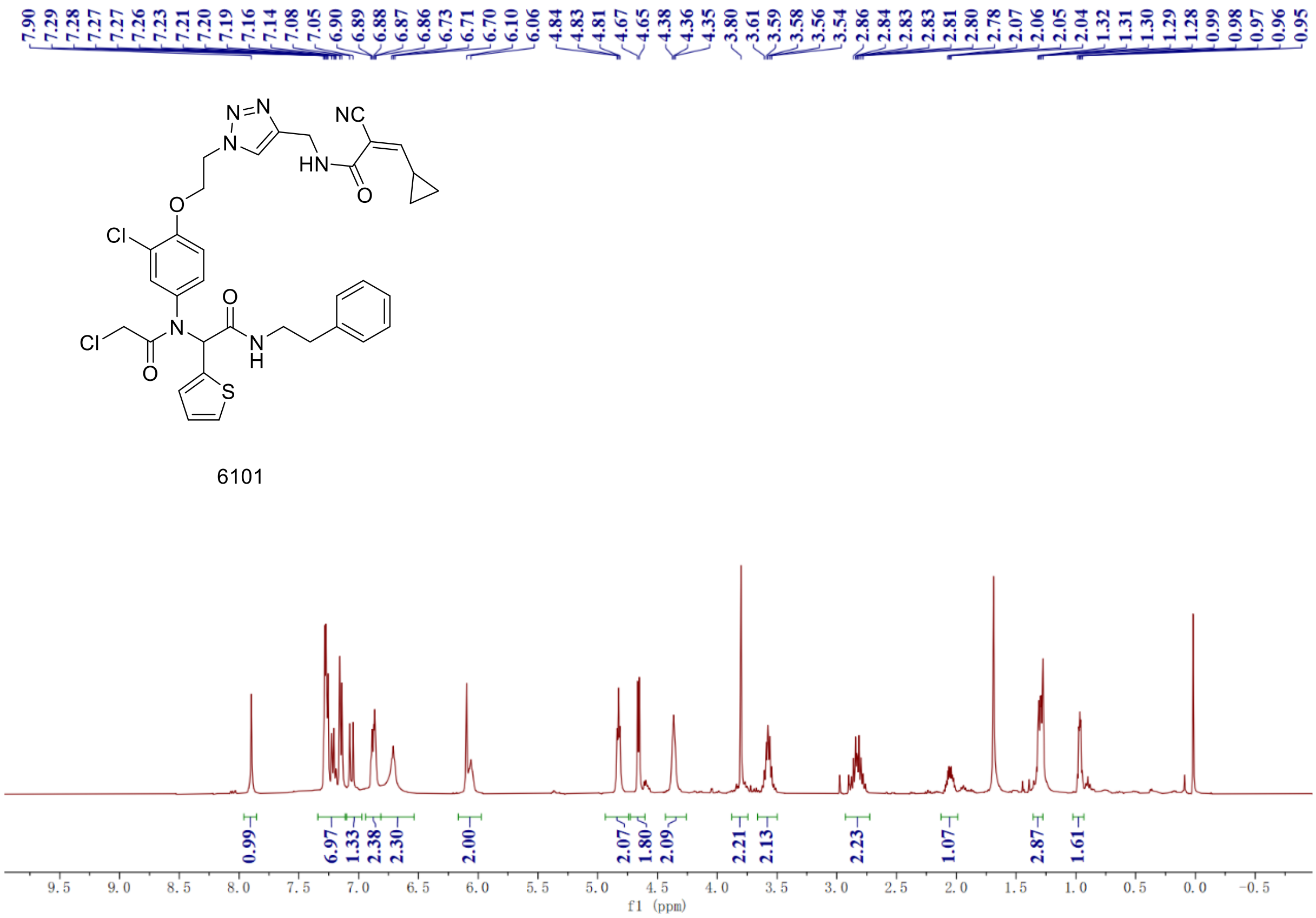

6101

^{1}H of 5075

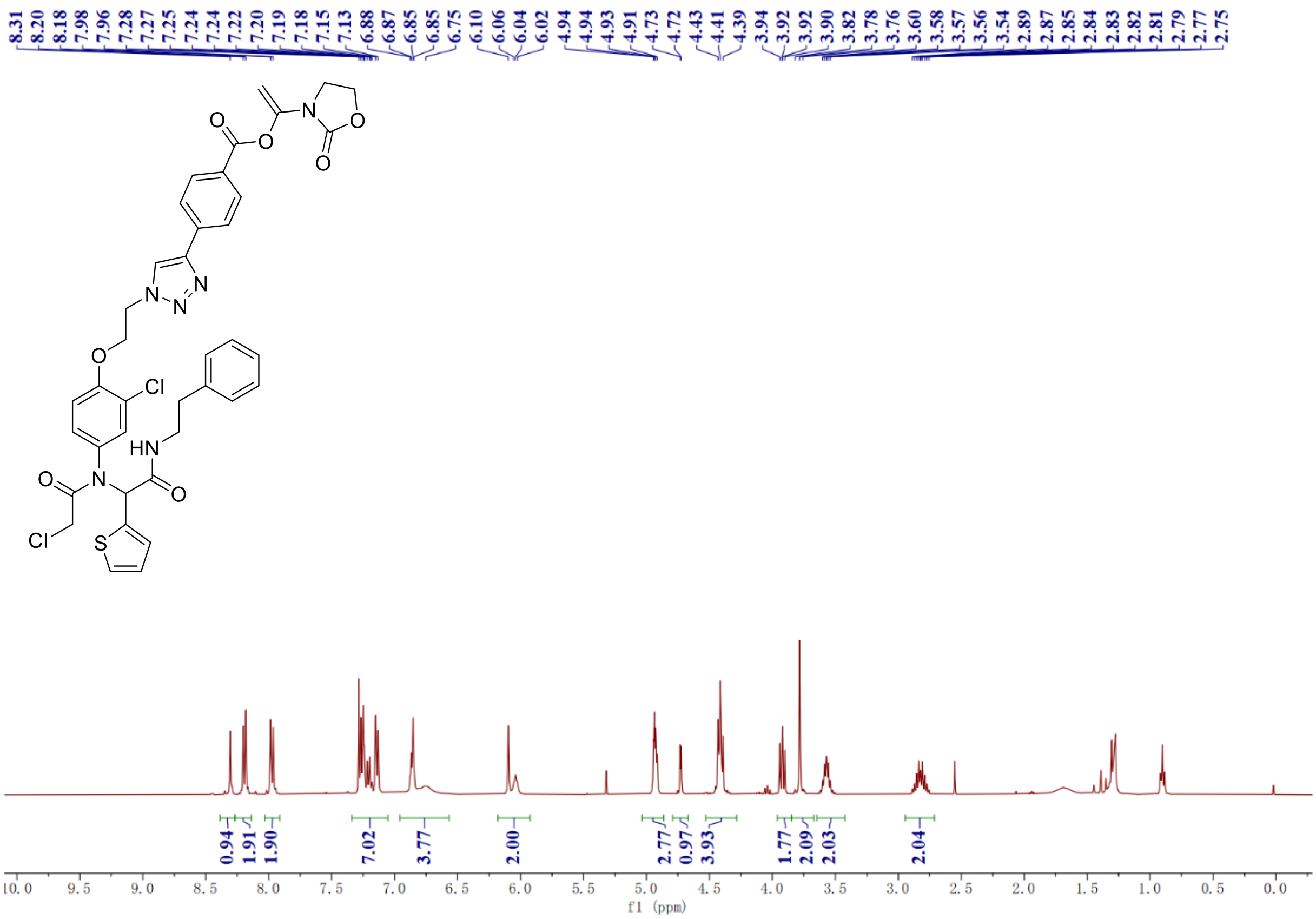

[1]H of 6090

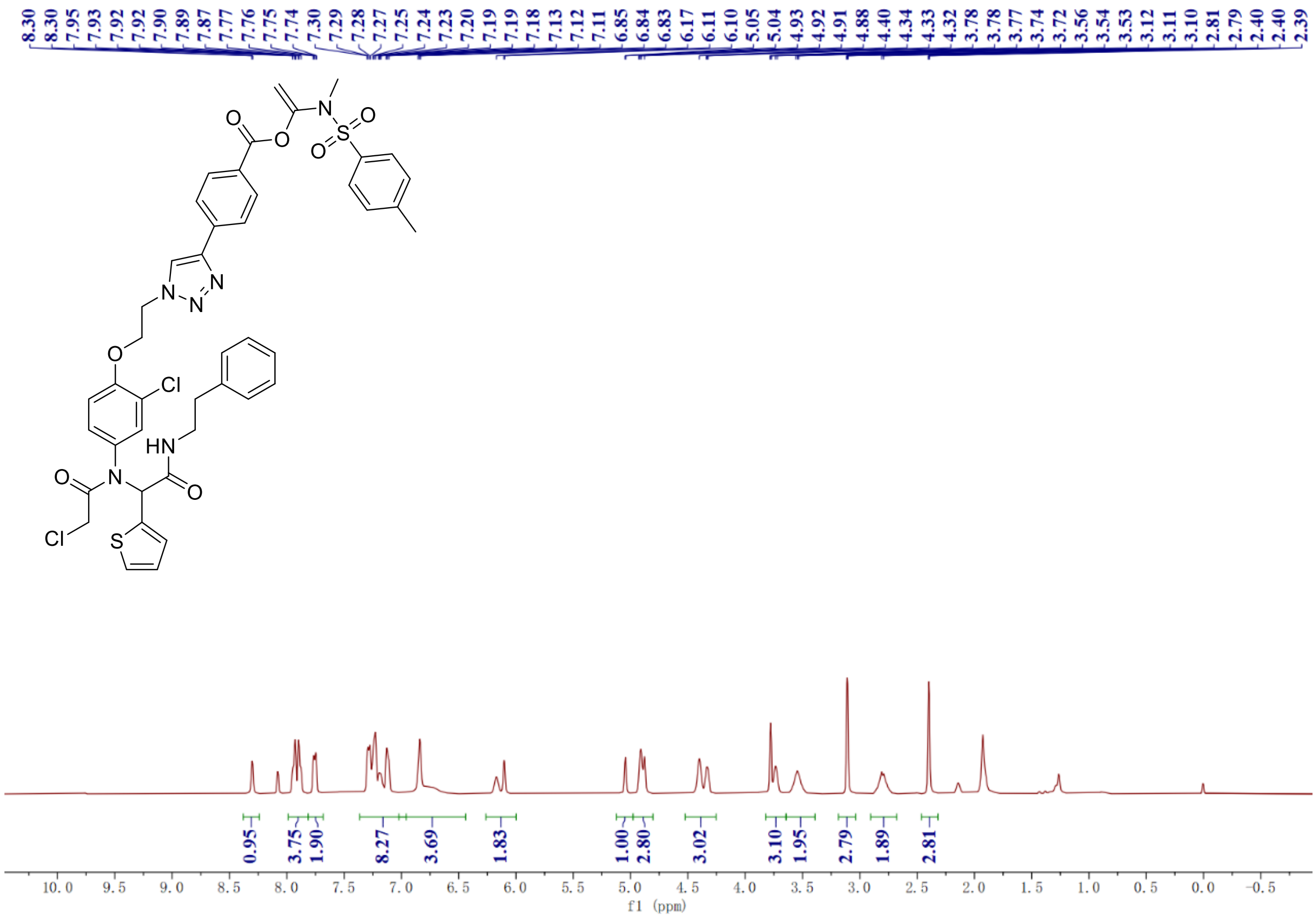

[13]C of 6090

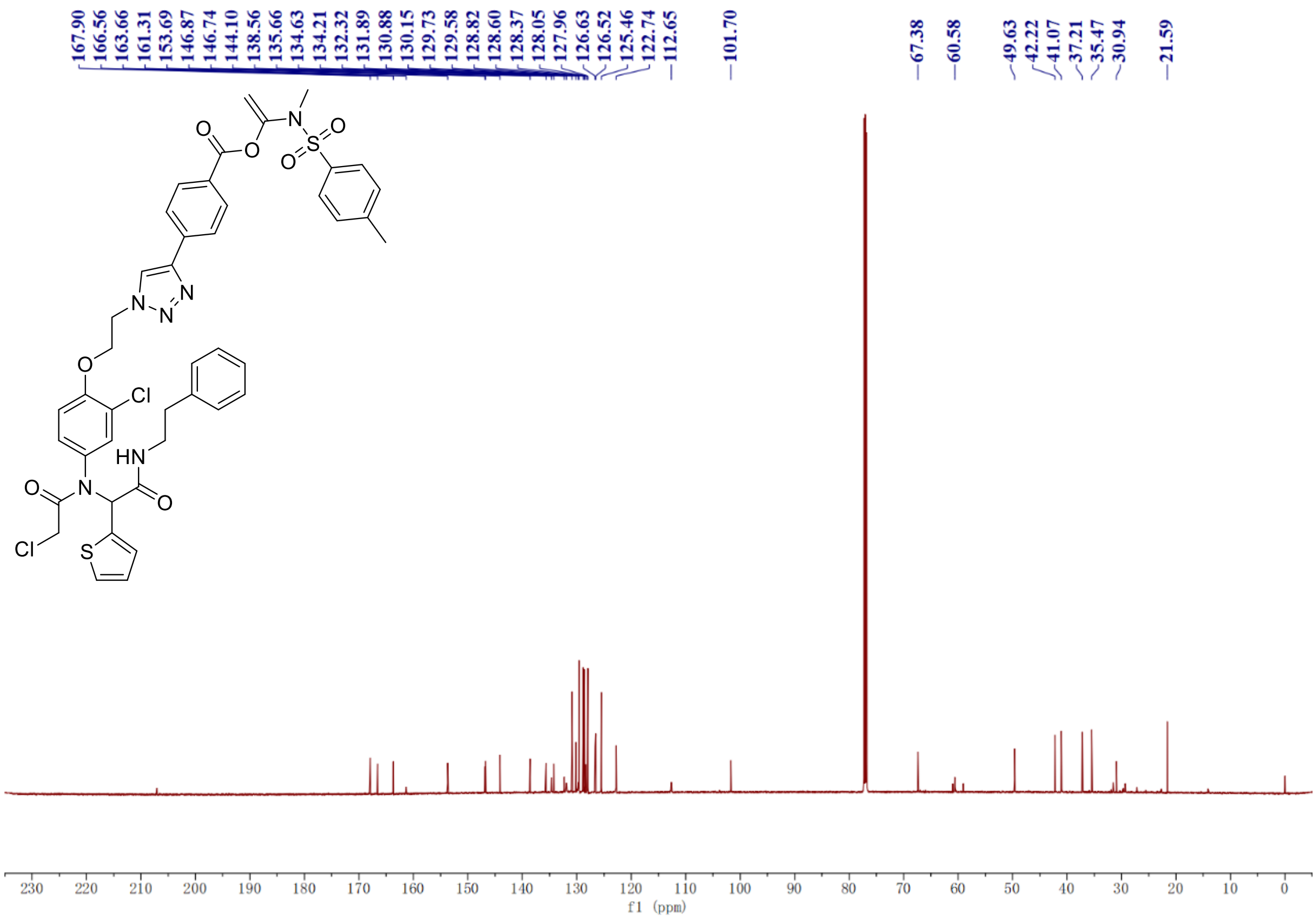

^{1}H of 5073

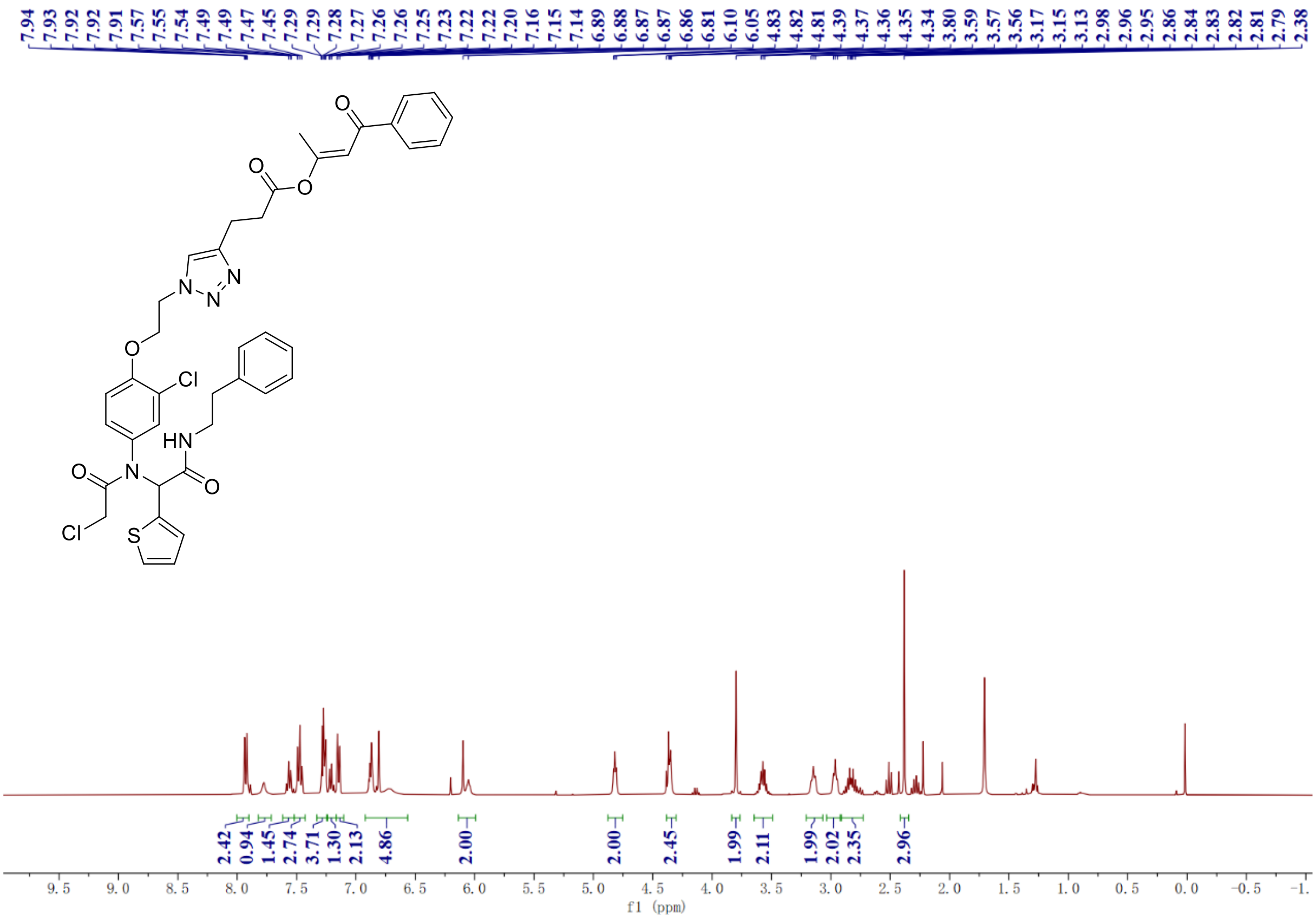

^{1}H of 5085

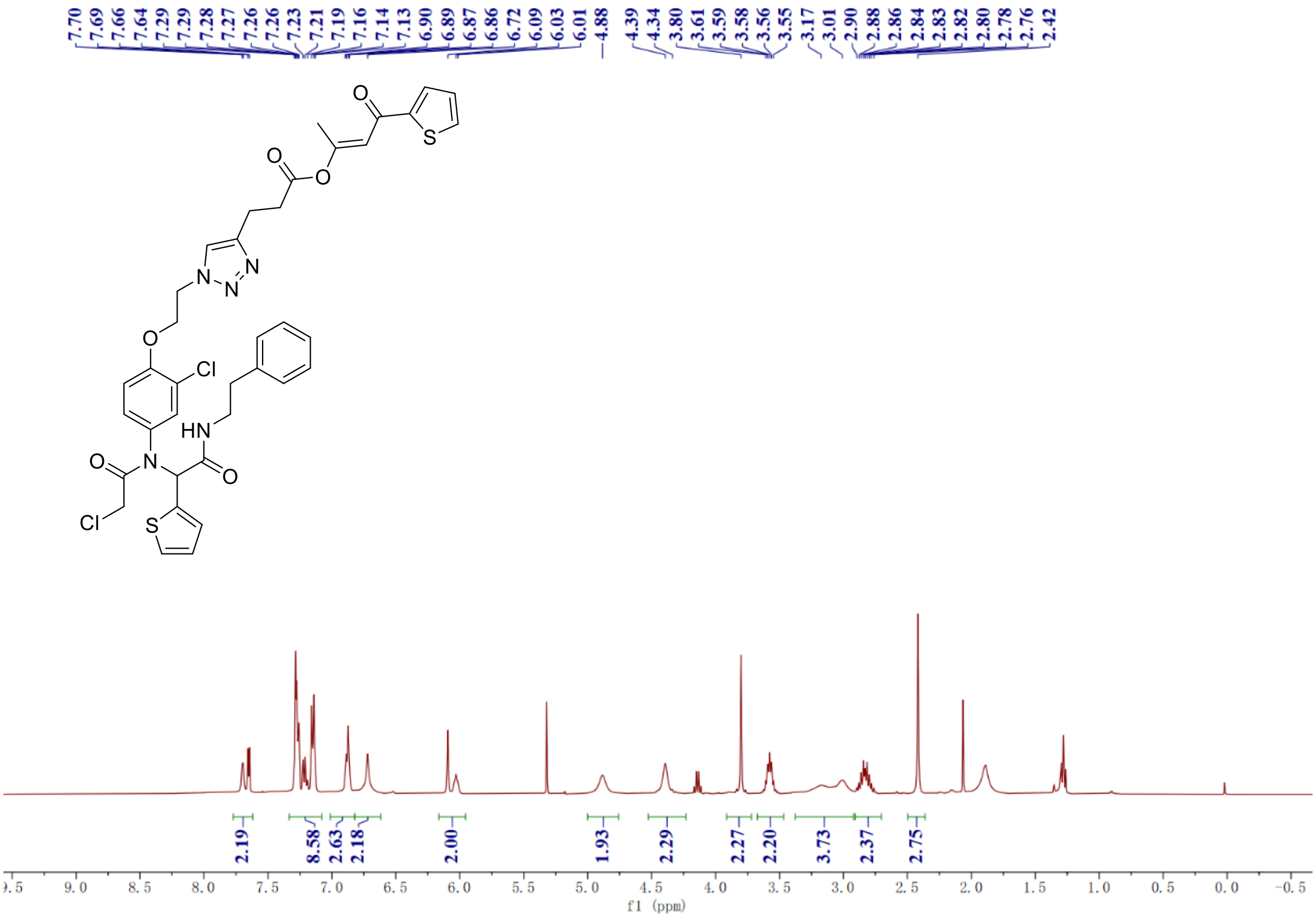

^{1}H of 5074

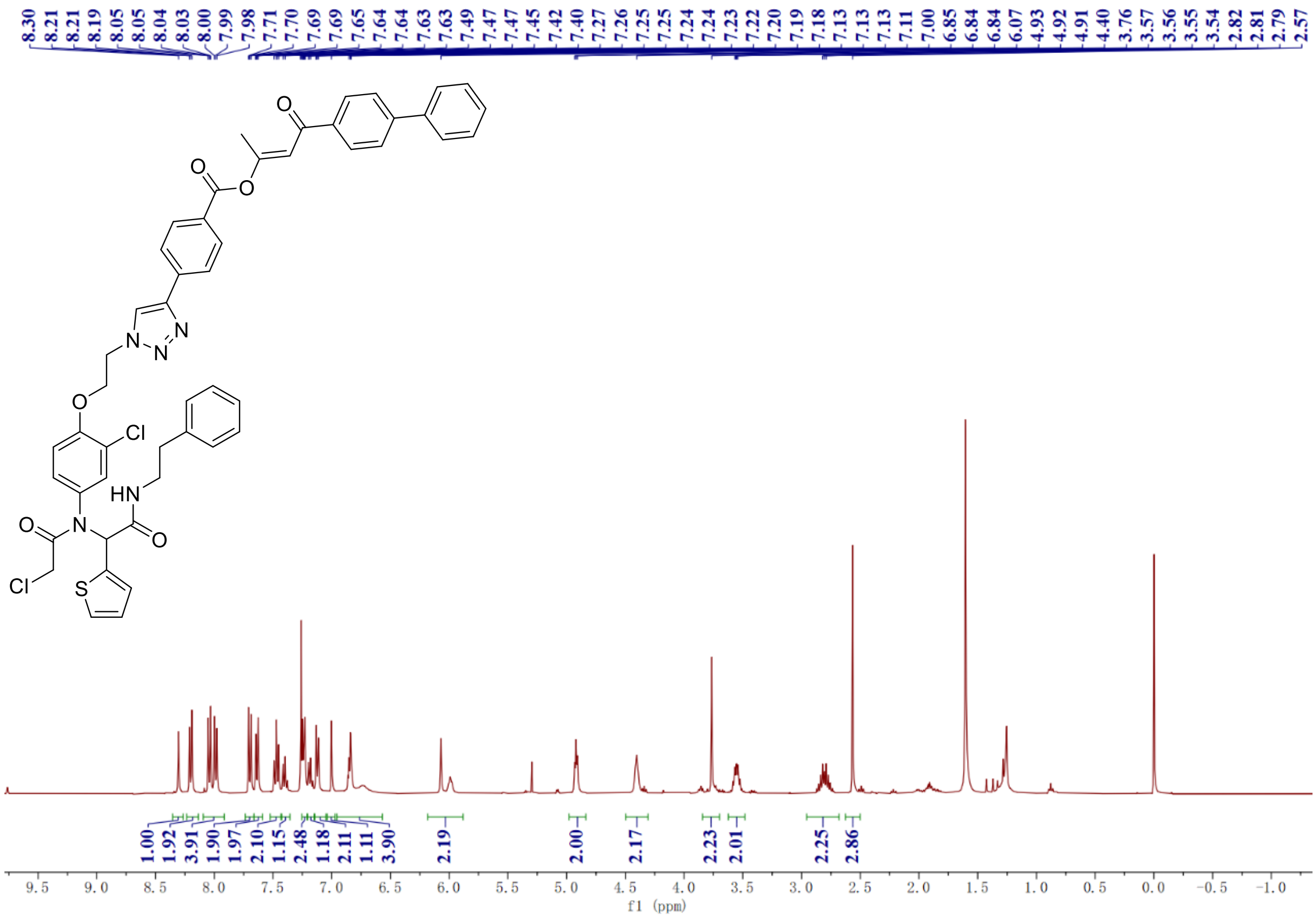

^{1}H of 5088

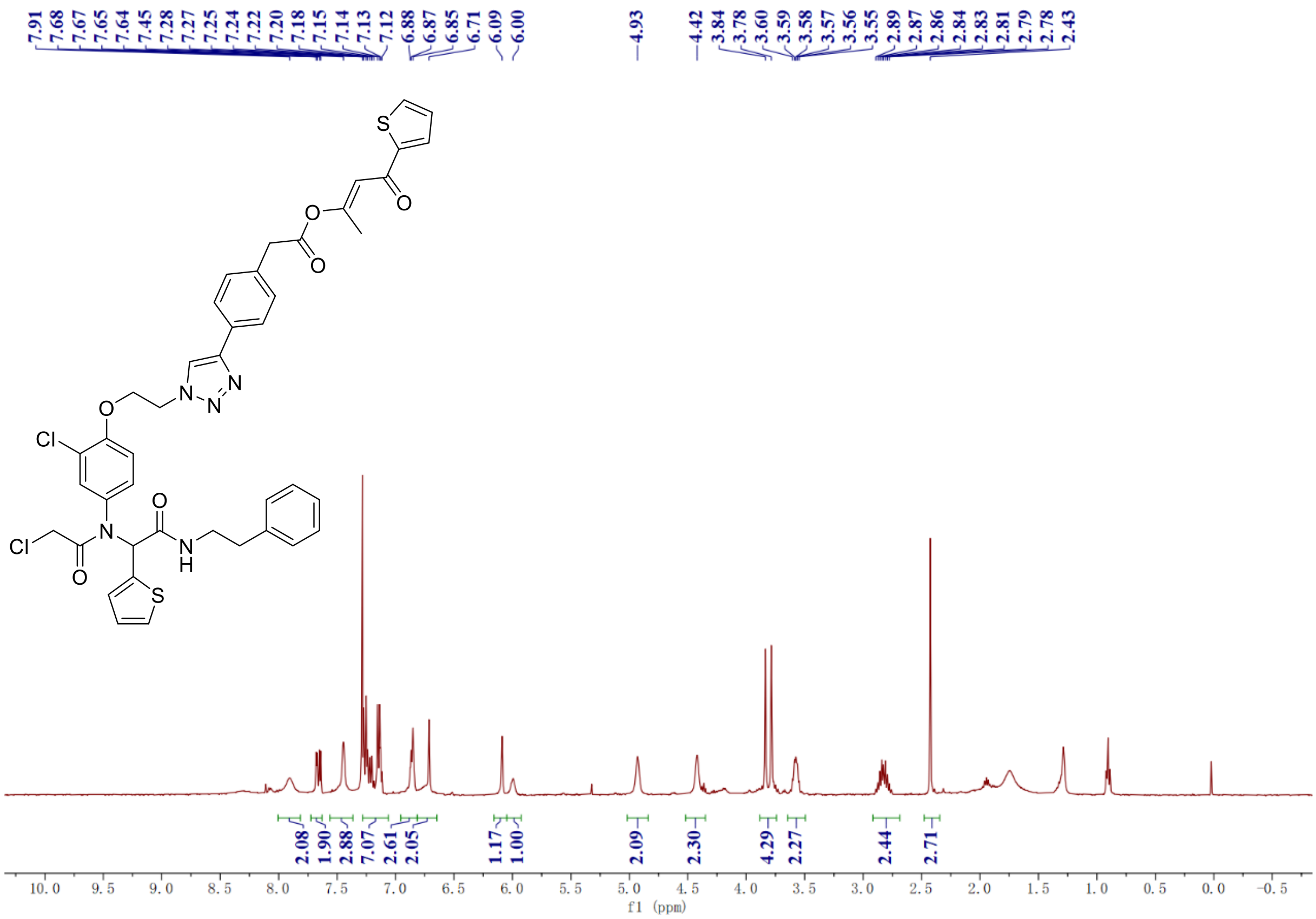

[1]H of 6082

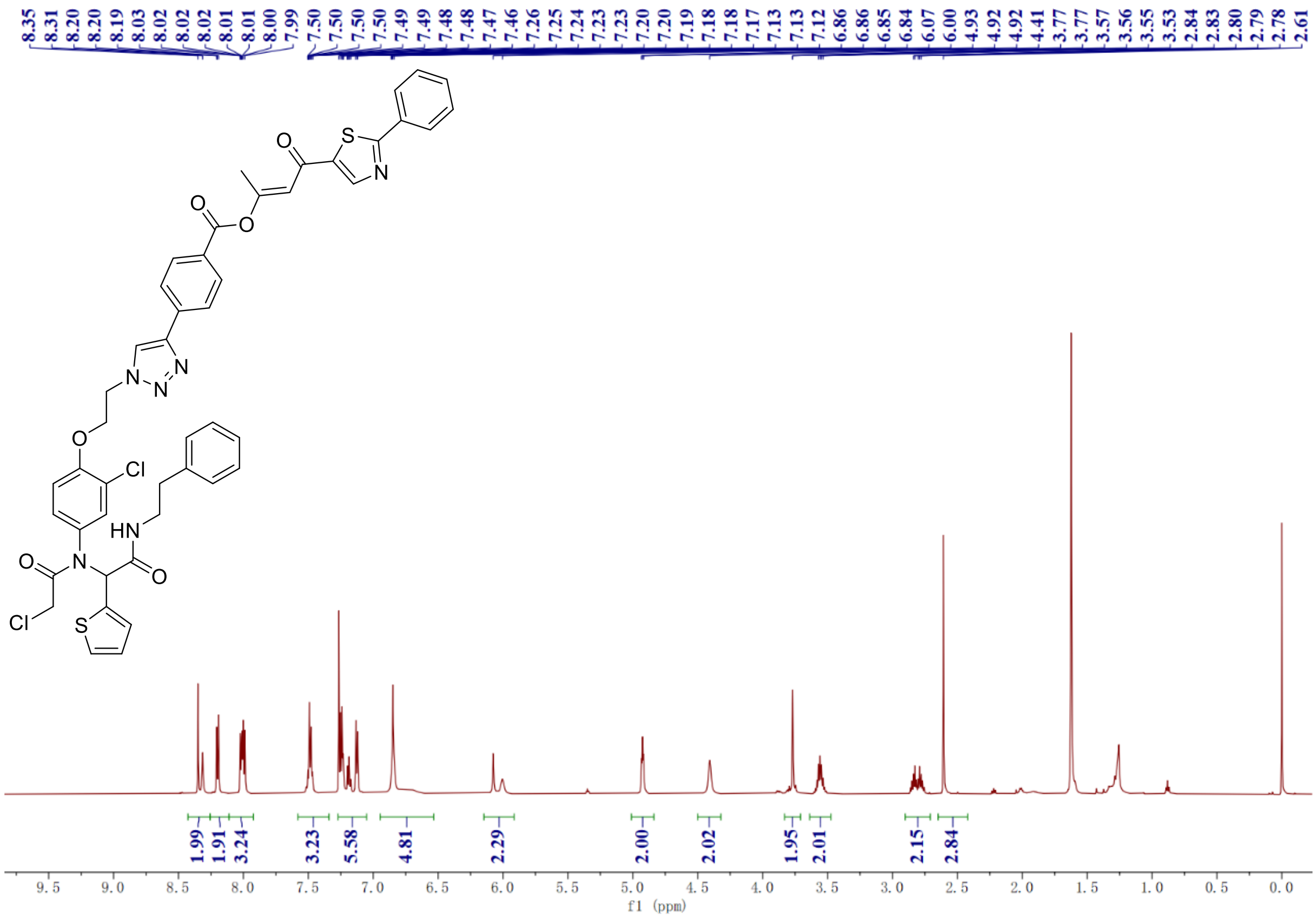

[13]C of 6082

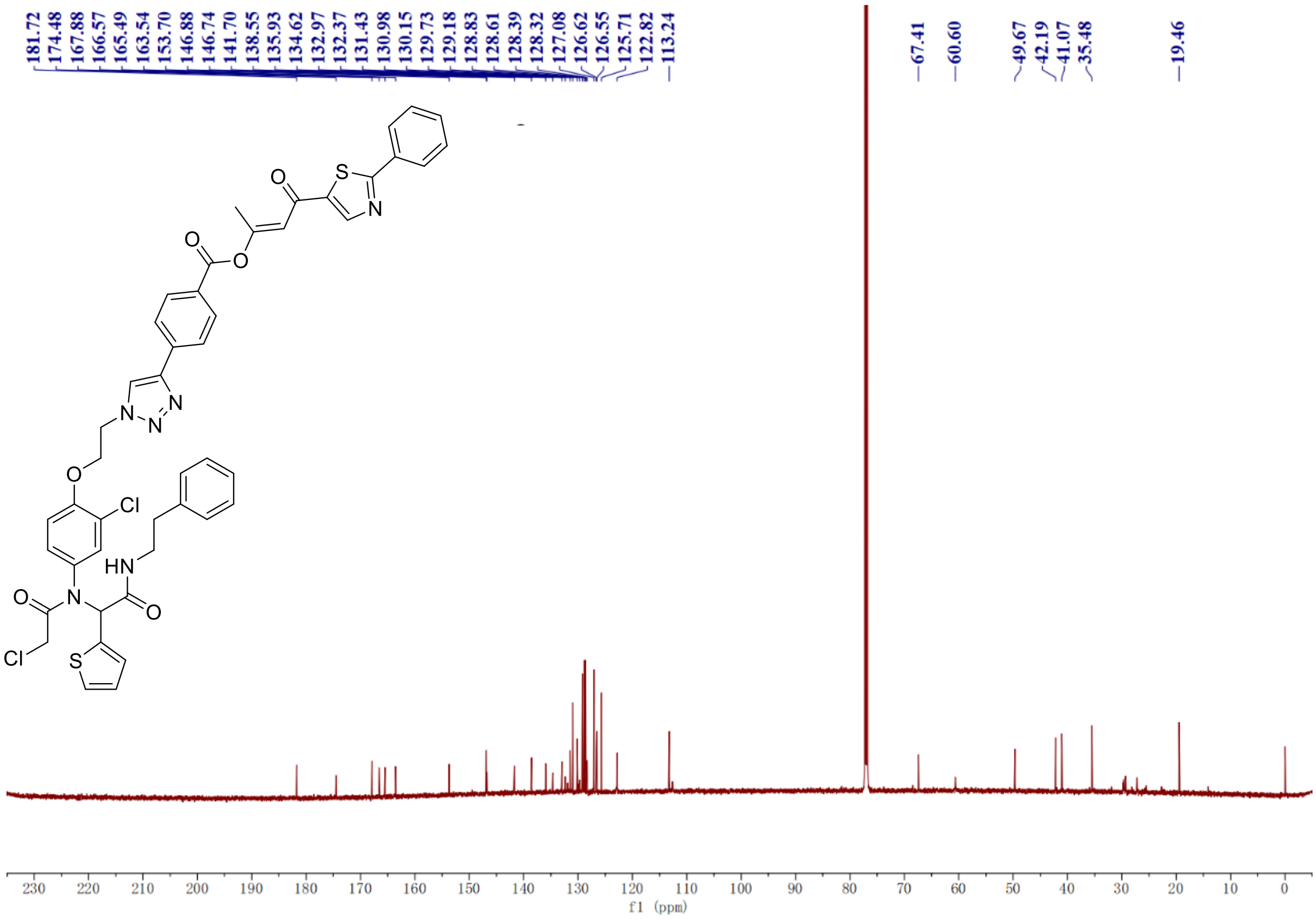

^{1}H of 6083

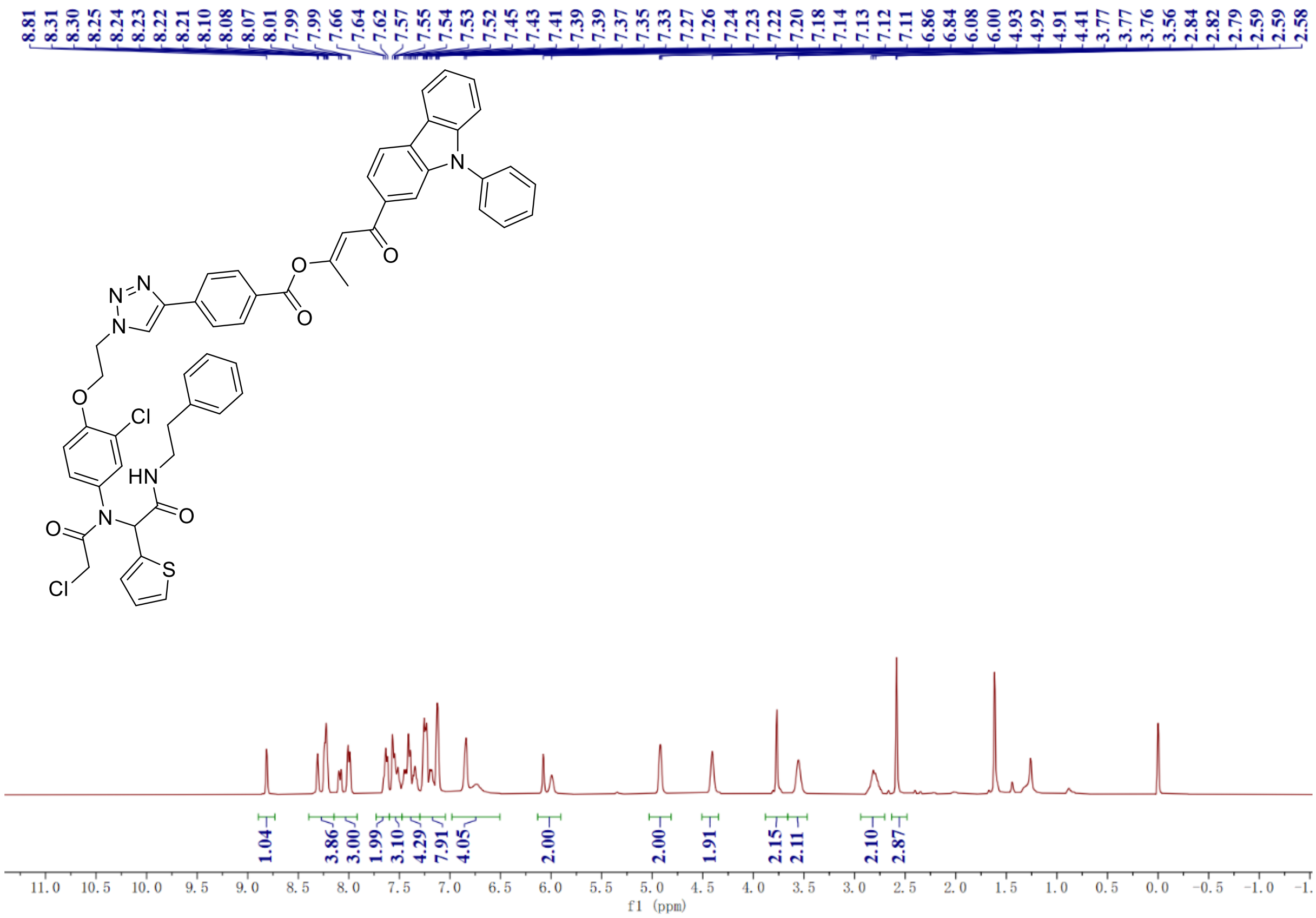

^{13}C of 6083

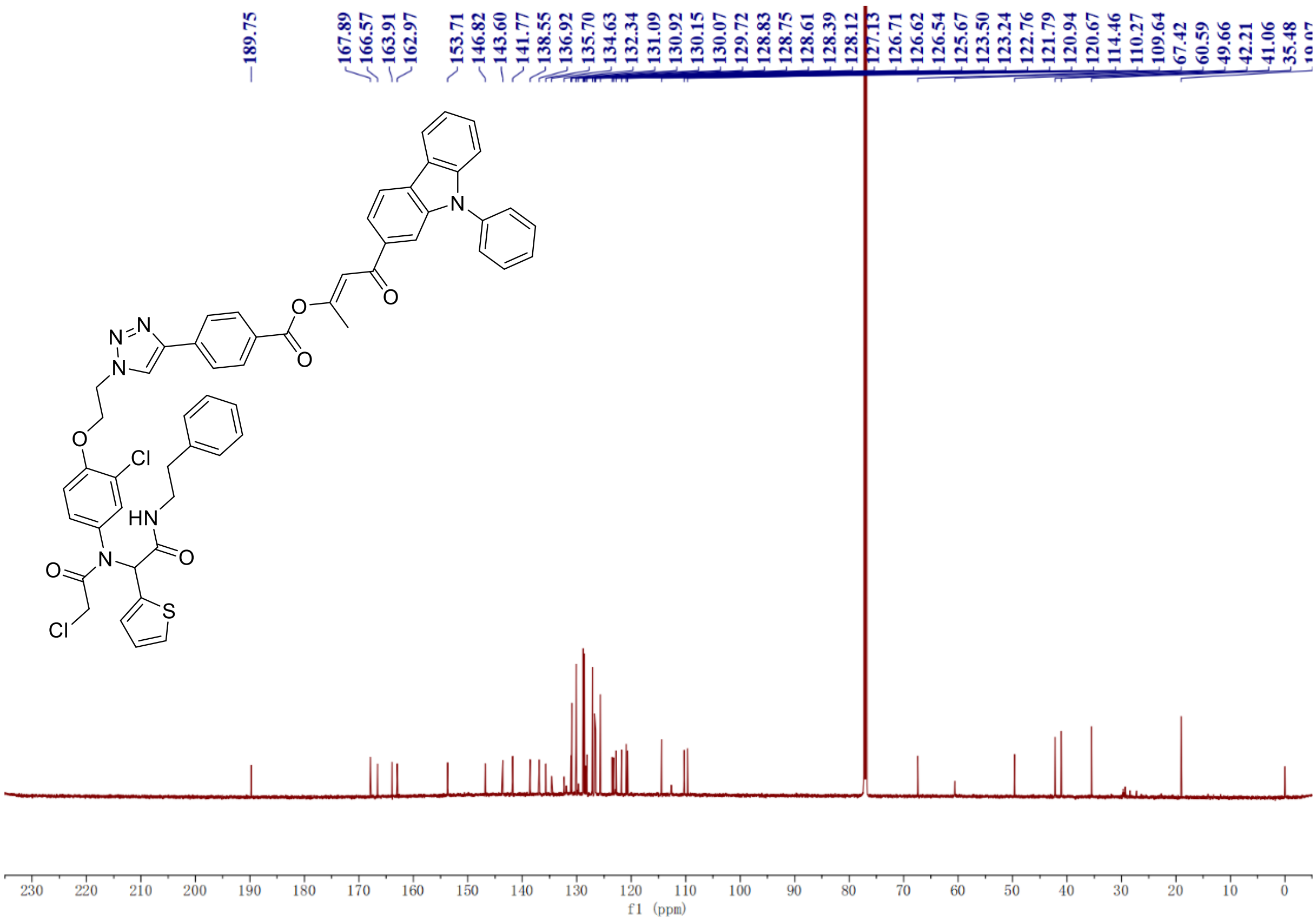

^{1}H of 6084

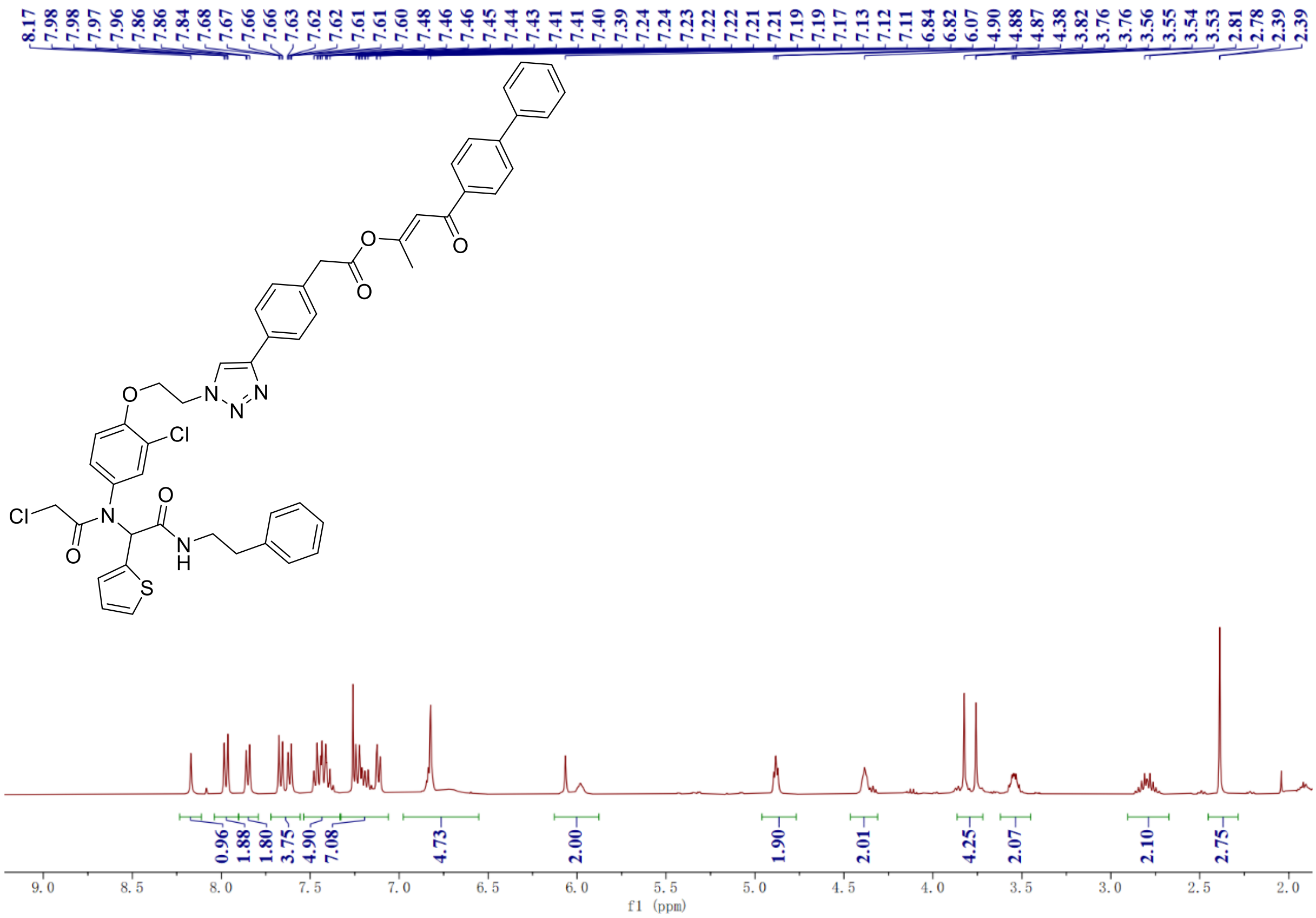

^{1}H of 6092

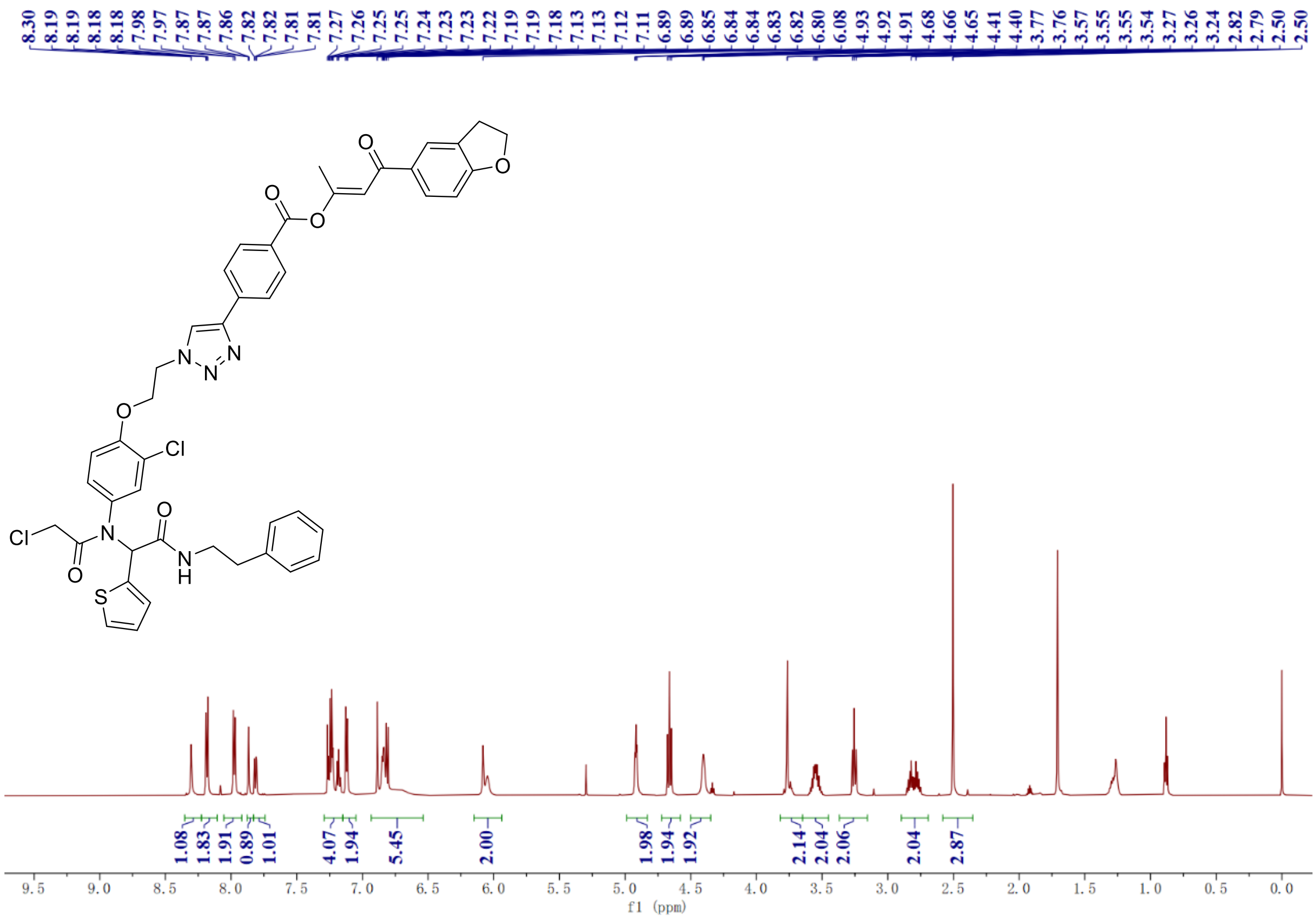

[13]C of 6092

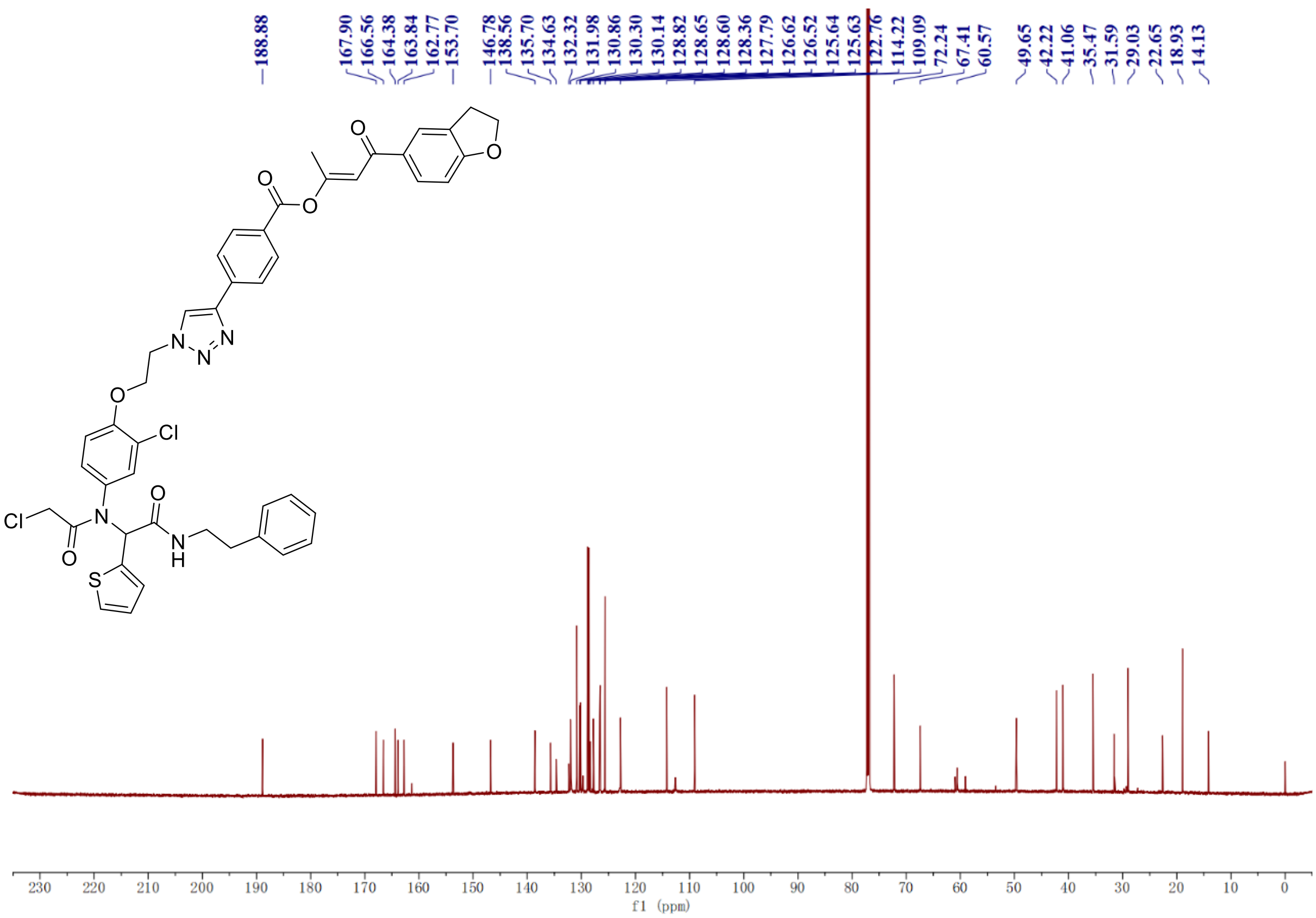

[1]H of 6093

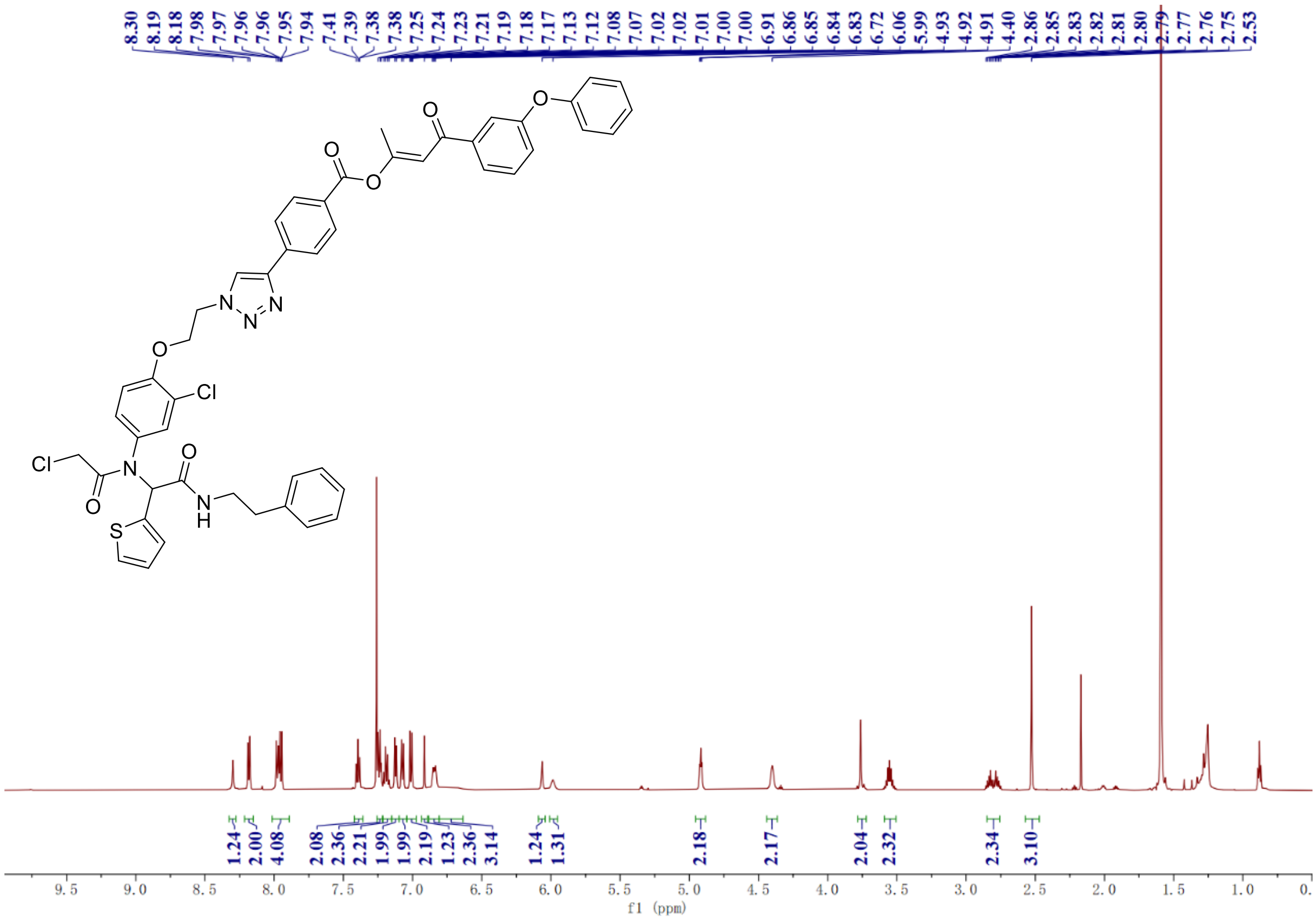

^{13}C of 6093

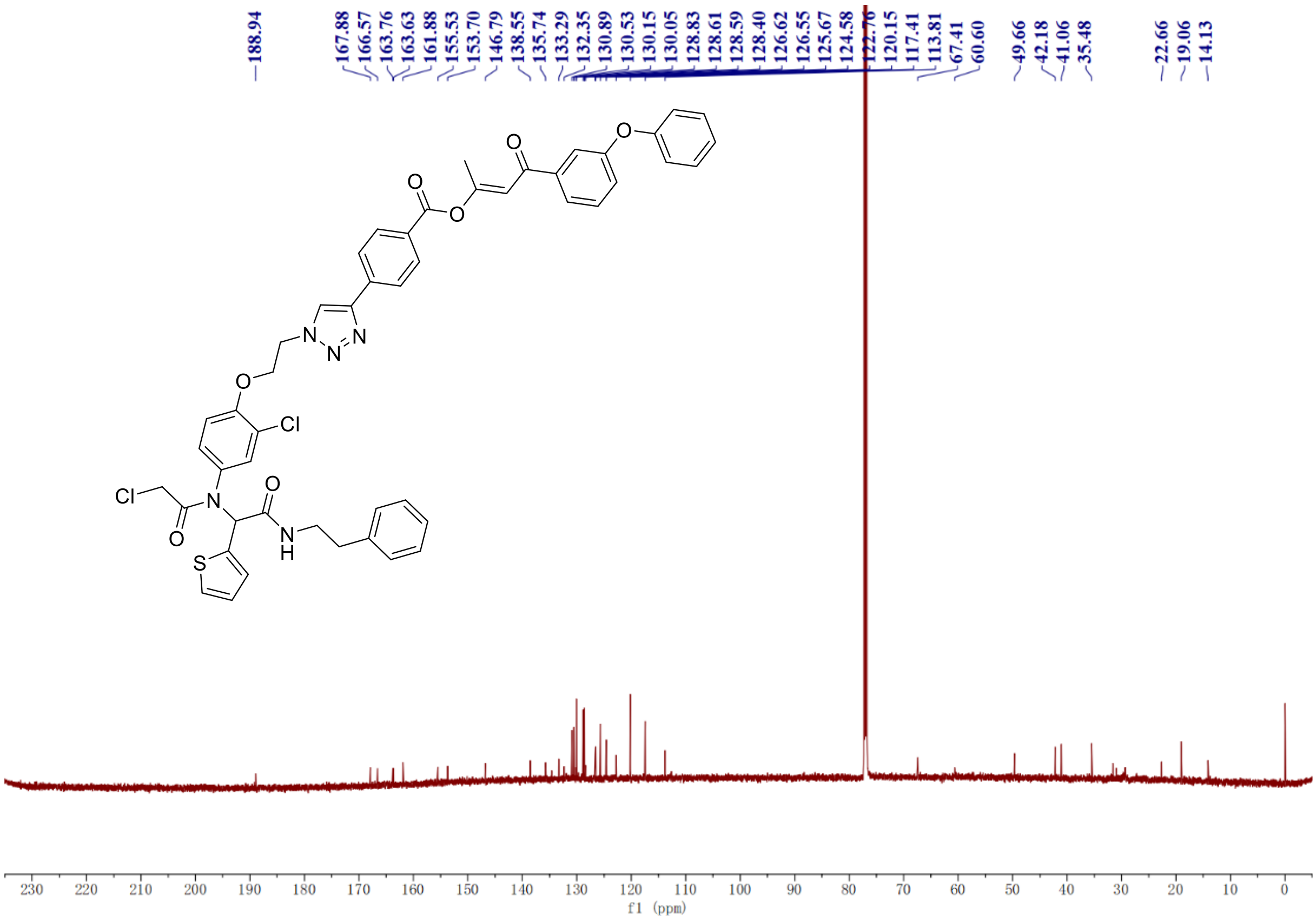

^{1}H of 6094

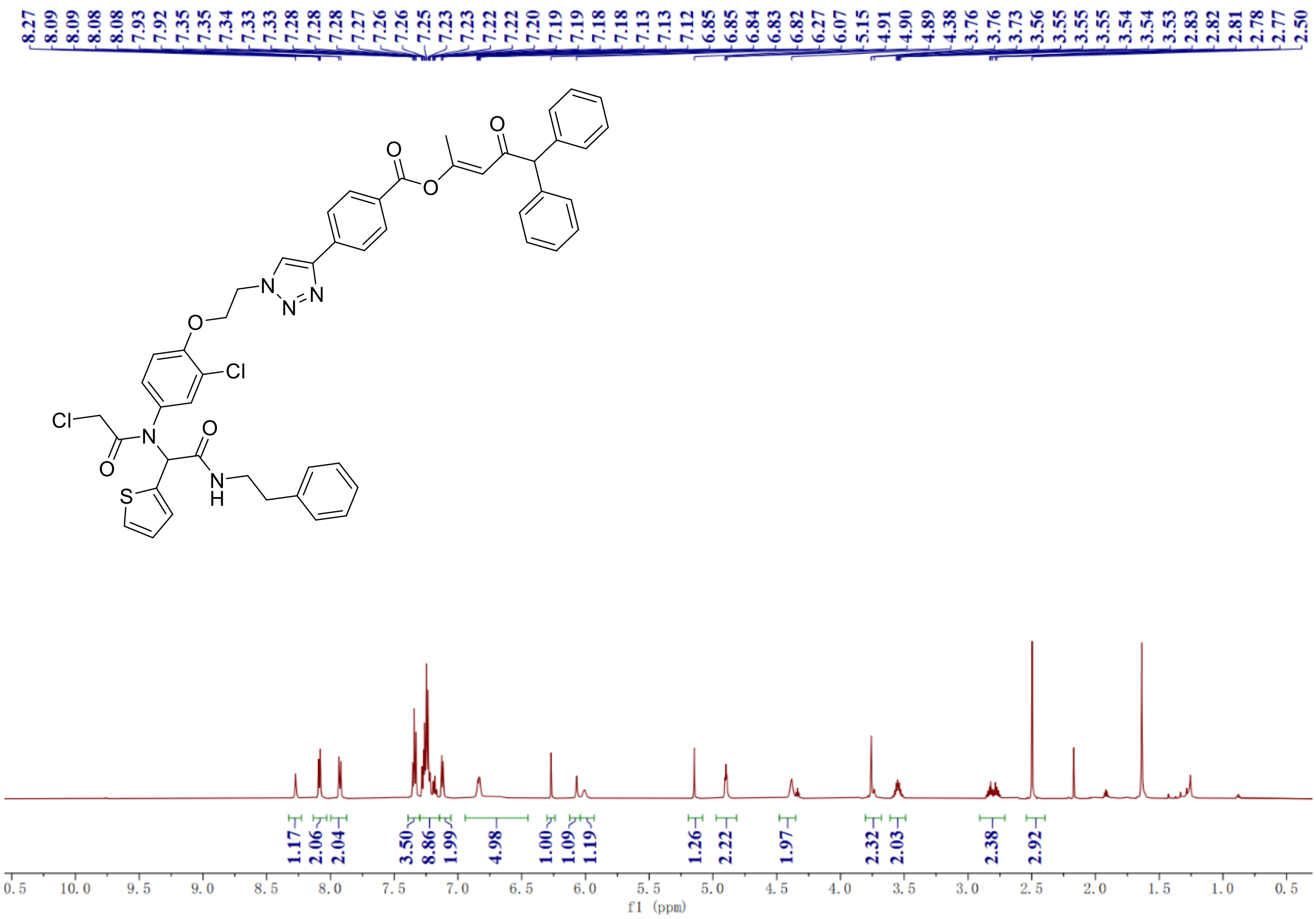

^{13}C of 6094

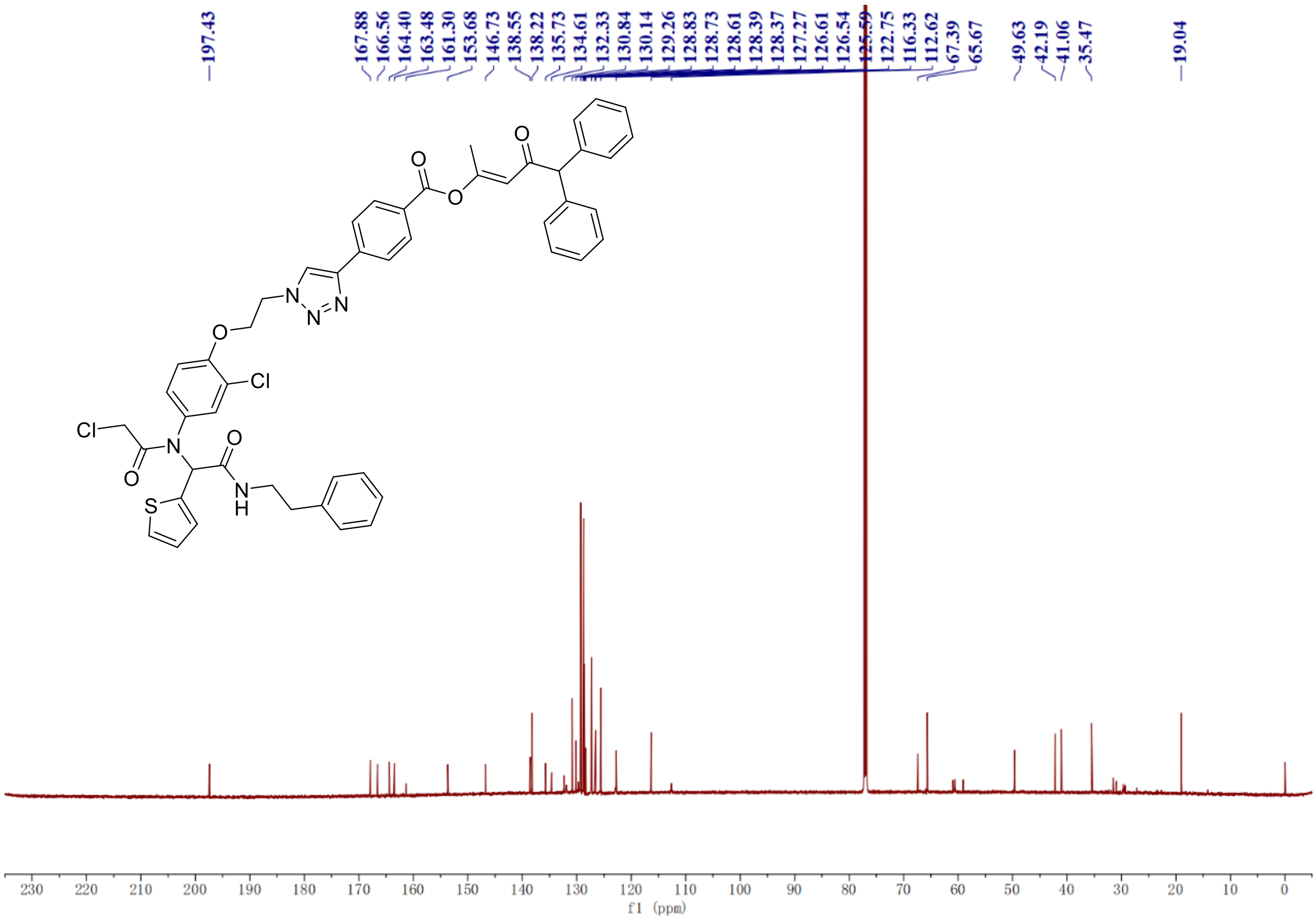

^{1}H of 6100

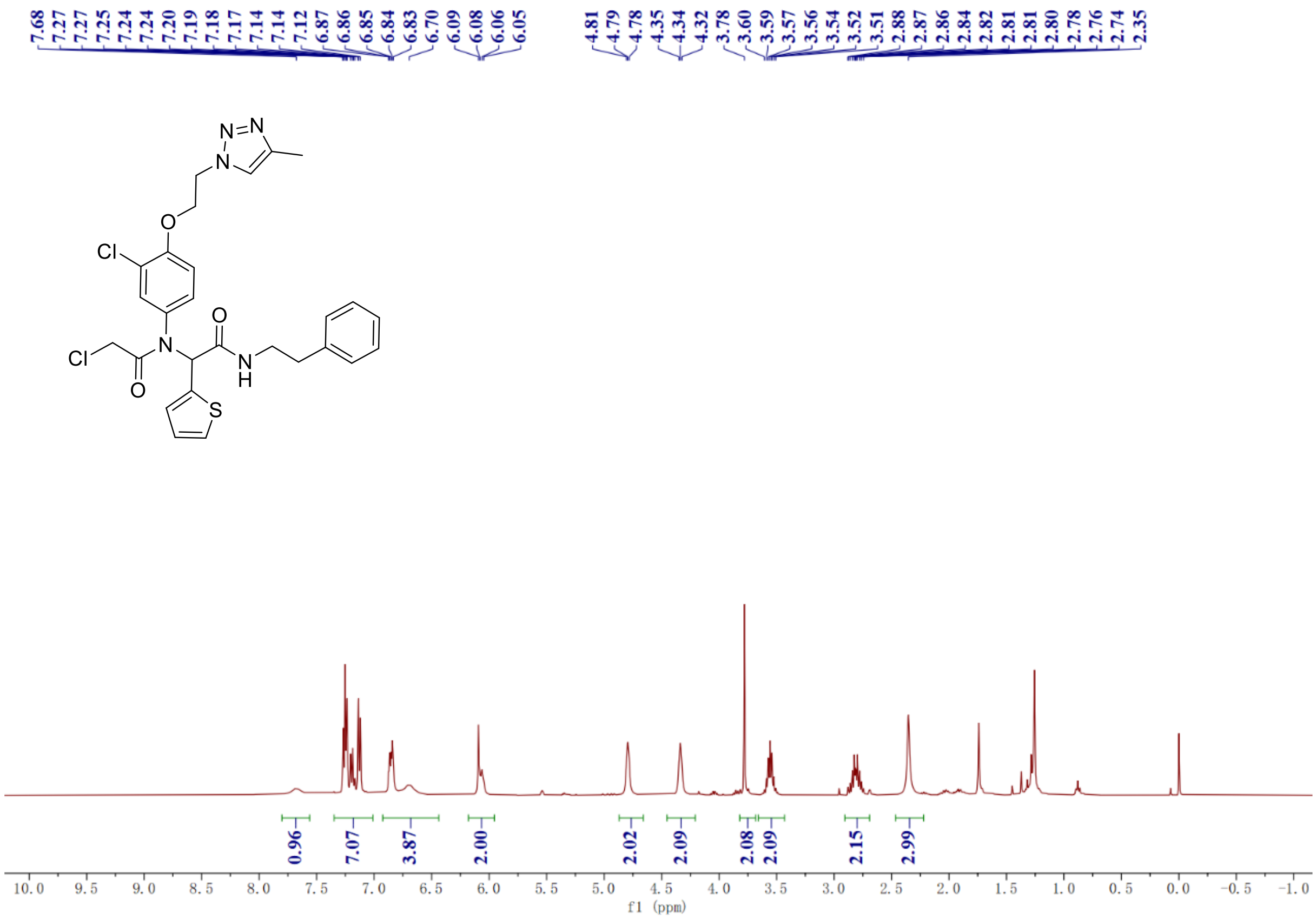

^{13}C of 6100

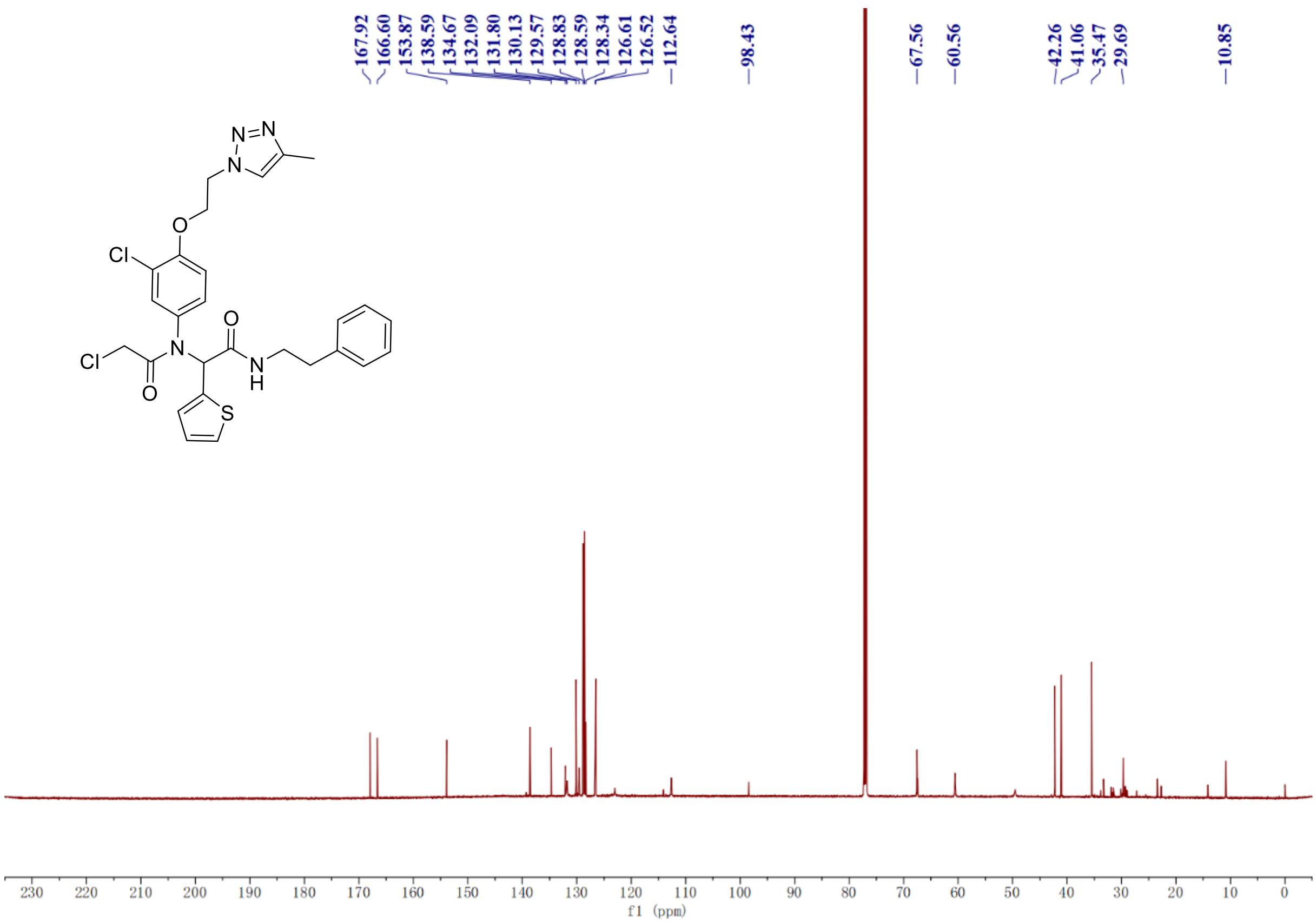

^{1}H of 6104

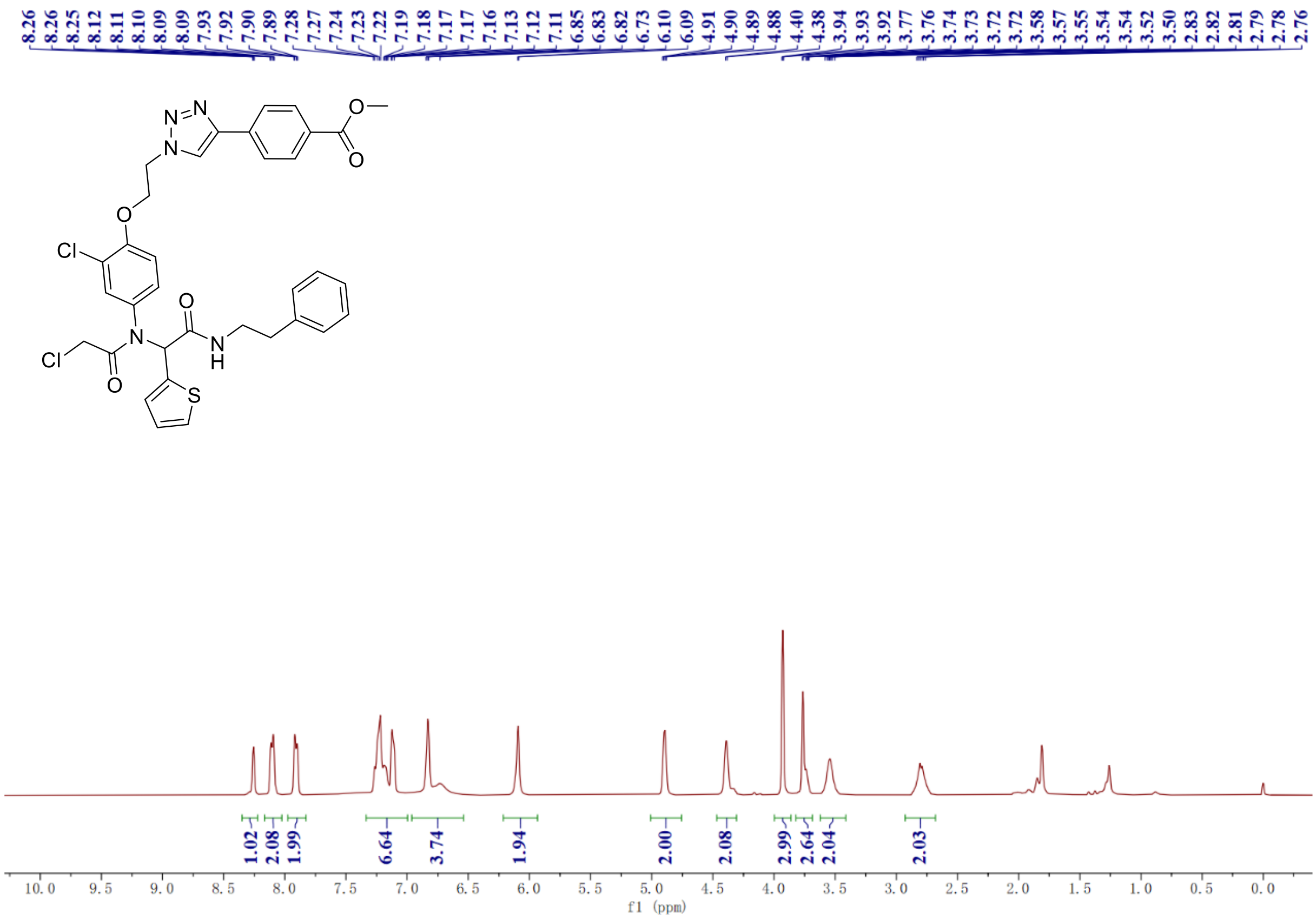

^{13}C of 6104

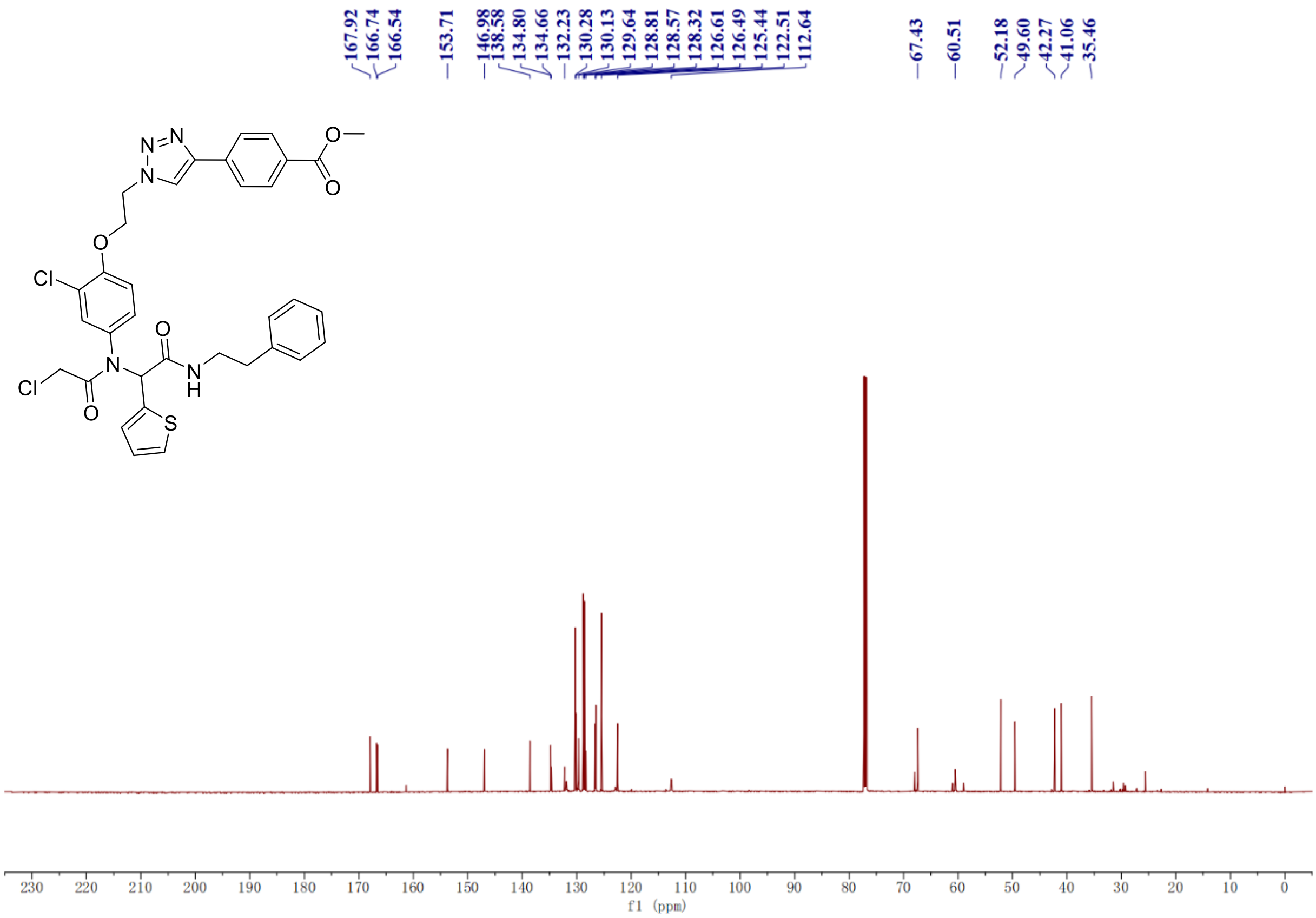

^{1}H of 6105

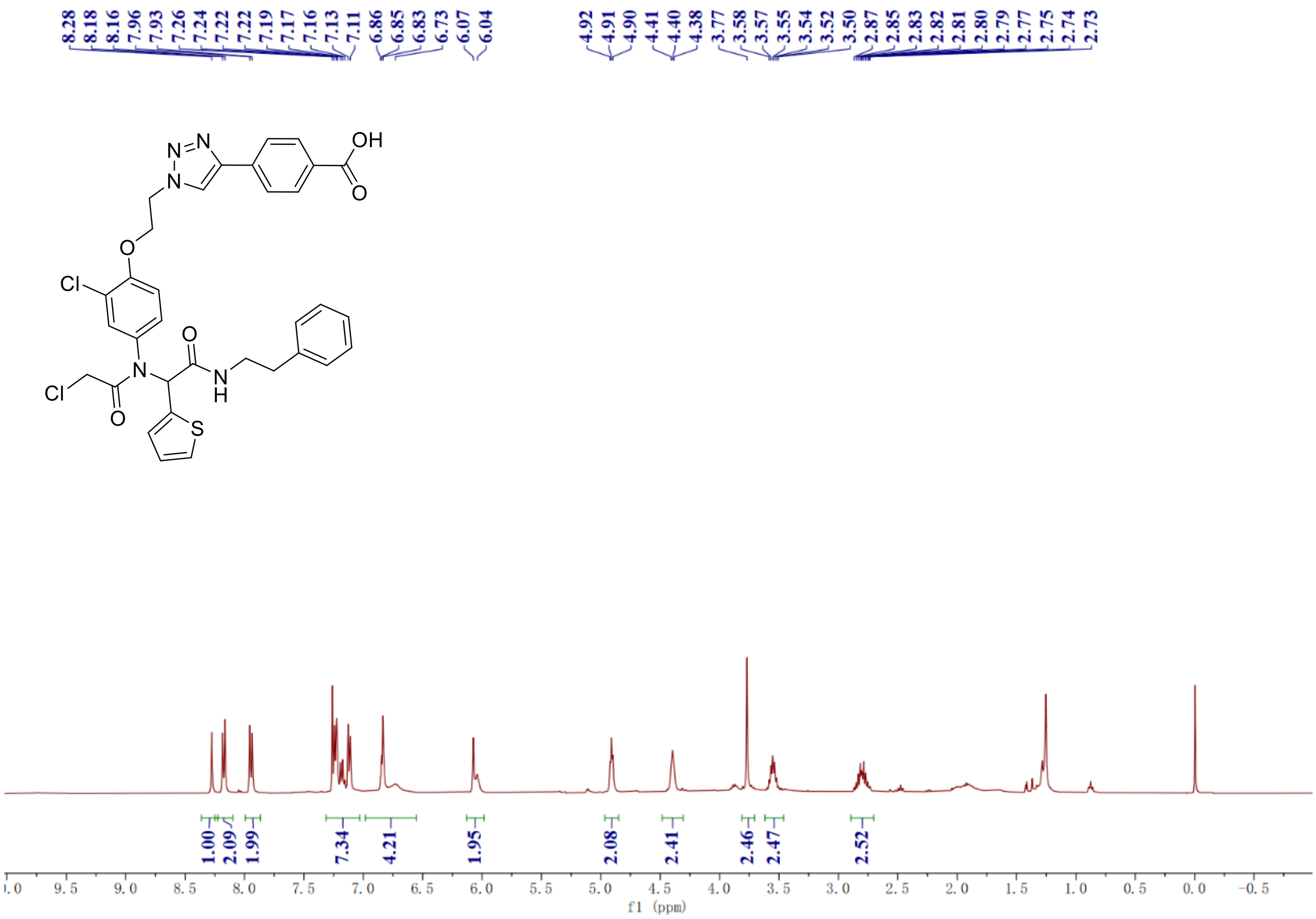

^{13}C of 6105

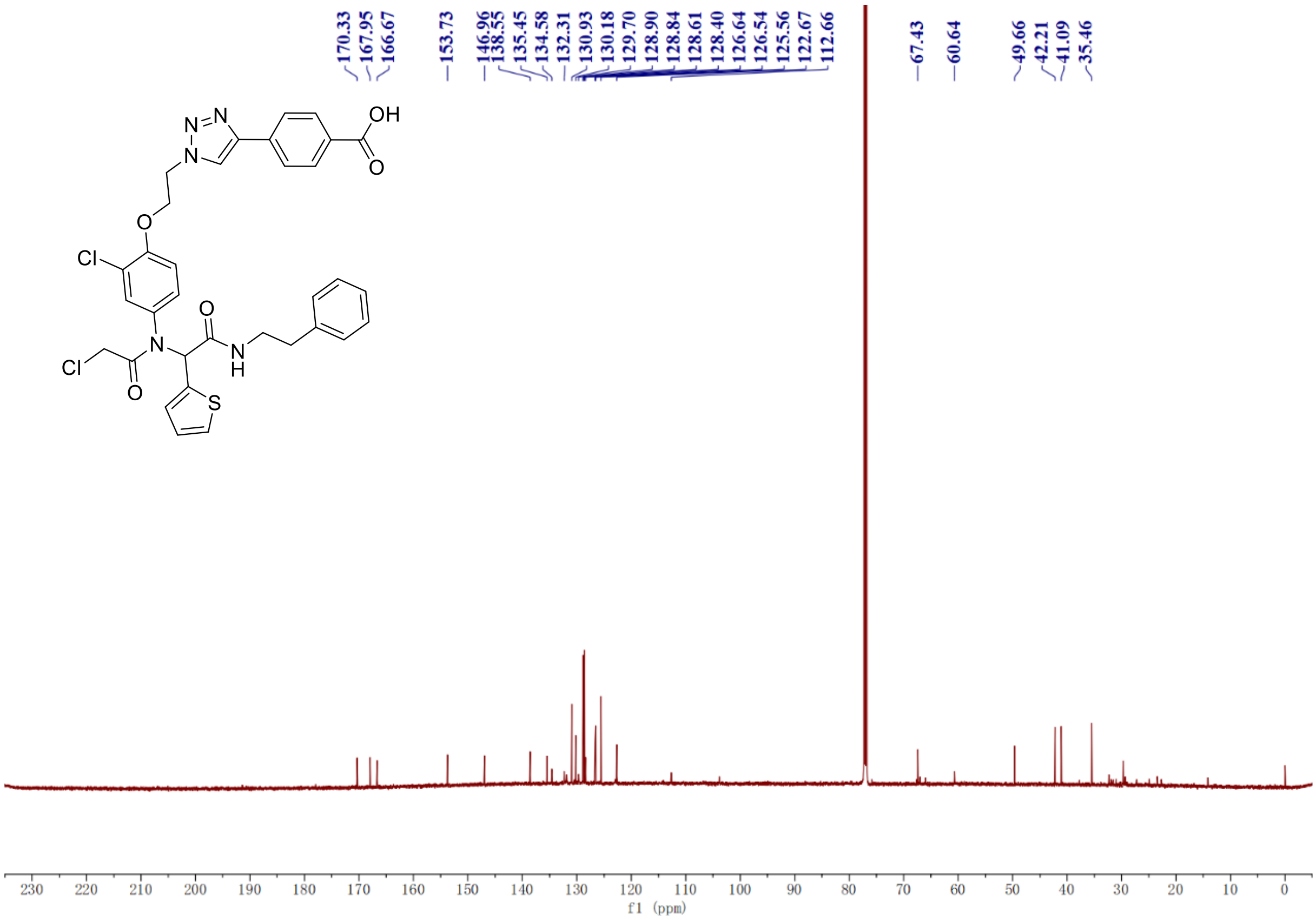

^{1}H of 6136

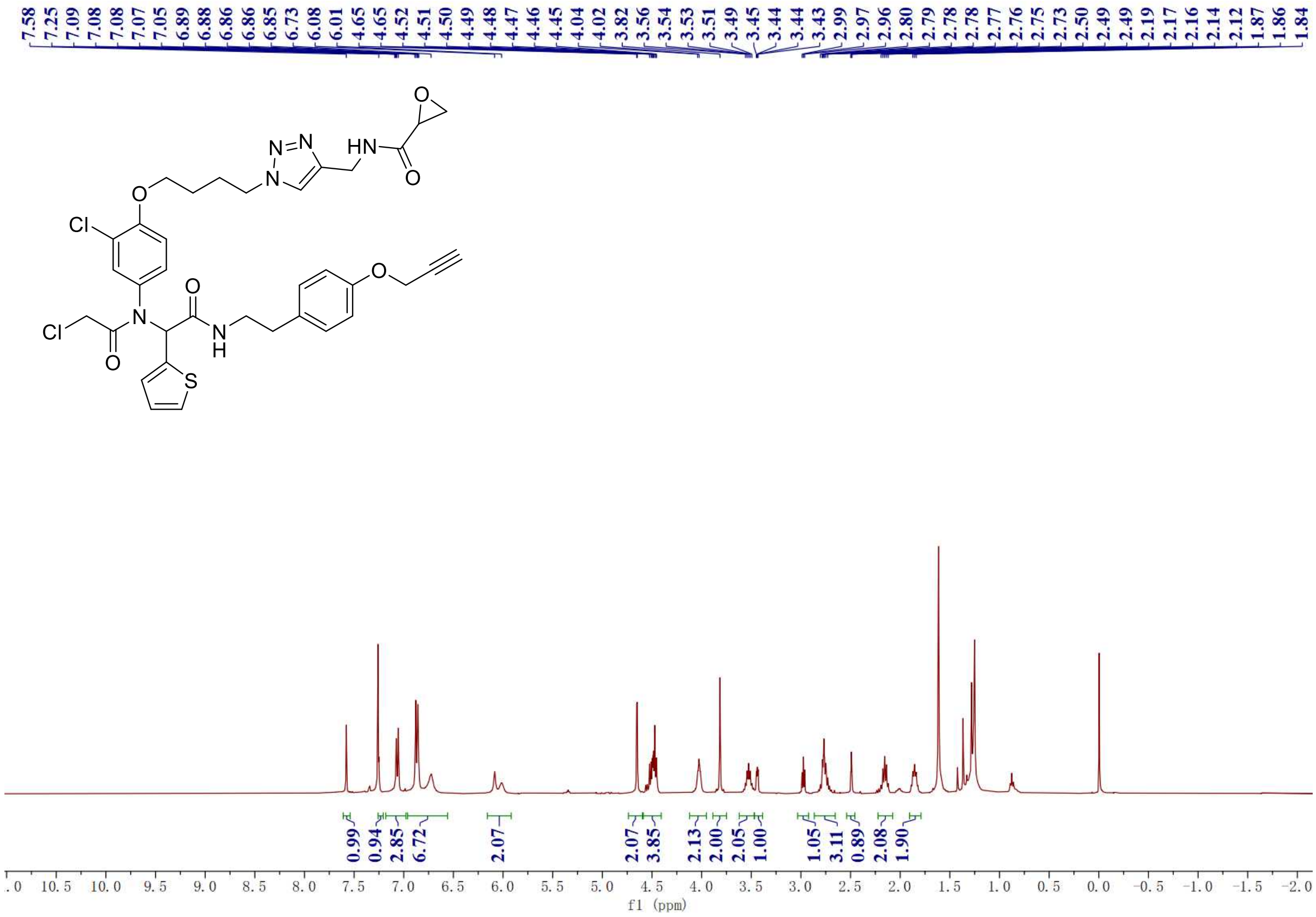

1H of 7057

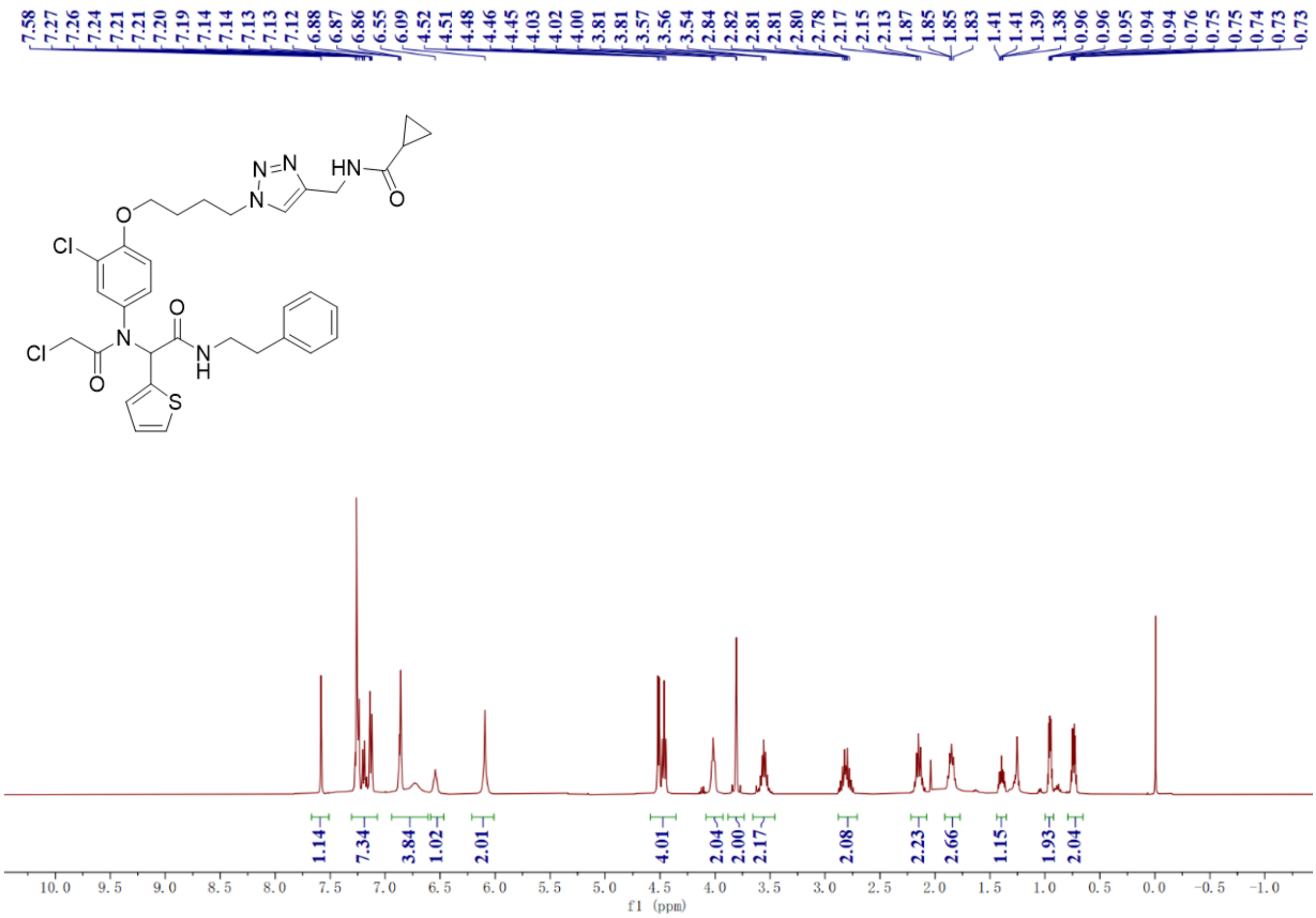

20. Supplementary References

[1] Kłossowski S, Redzej A, Szymkuć S, Ostaszewski R. Studies towards enzymatic kinetic resolutions of 1,3-diol peptidomimetics obtained via the Ugi reaction. Arkivoc. 2013;2013(8):134-143.

[2] Liu Y, Yu Z, Li P, et al. Proteome-wide Ligand and Target Discovery by Using Strain-Enabled Cyclopropane Electrophiles. J Am Chem Soc. 2024;146(30):20823-20836.

[3] Liu Y, Liu J, Zhang X, et al. Oxidant-Induced Bioconjugation for Protein Labeling in Live Cells. ACS Chem Biol. 2023;18(1):112-122.

[4] Feng Y, Zeng S, Xu F, et al. Dioxazolones as Masked Isocyanate Electrophiles for Global Profiling of Lysine Reactivity and Ligandability. J Am Chem Soc. 2025;147(50):46550-46562.

[5] Gingras, A. C.; Gstaiger, M.; Raught, B.; Aebersold, R. Analysisof protein complexes using mass spectrometry. Nat. Rev. Mol. Cell Biol. 2007, 8 (8), 645−654.

[6] Tyanova, S.; Temu, T.; Sinitcyn, P.; Carlson, A.; Hein, M. Y.; Geiger, T.; Mann, M.; Cox, J. The Perseus computational platform for comprehensive analysis of (prote)omics data. Nat. Methods 2016, 13 (9), 731-740.

[7] Liu Y, Yang T, Huang L, et al. Chemical Proteomics Identifies RBBP7 as a New E3 Ligase Supporting Targeted Protein Degradation. Angew Chem Int Ed Engl. 2025;64(36):e202508538.

[8] Dong J, Li Y, Liang H, et al. HSP70 Interactome-Mediated Proteolysis Targeting Chimera (HSP70-PROTAC) for Ferroptosis-Driven Cancer Treatment. Adv Sci (Weinh). 2026;13(12):e13655.

[9] Chang, L., et al., Discovery of small molecules targeting the tandem tudor domain of the epigenetic factor UHRF1 using fragment-based ligand discovery. Sci Rep, 2021. 11(1): p. 1121.

[10] Sastry, G.M., et al., Protein and ligand preparation: parameters, protocols, and influence on virtual screening enrichments. J Comput Aided Mol Des, 2013. 27(3): p. 221-34.

[11] Repasky, M.P., M. Shelley, and R.A. Friesner, Flexible ligand docking with Glide. Curr Protoc Bioinformatics, 2007. Chapter 8: p. Unit 8 12

[12] Zhu, K., et al., Docking covalent inhibitors: a parameter free approach to pose prediction and scoring. J Chem Inf Model, 2014. **54**(7): p. 1932-40

[13] Suenaga, A., et al., An efficient computational method for calculating ligand binding affinities. PLoS One, 2012. **7**(8): p. e42846

21. Source data

Figure 2B in main text

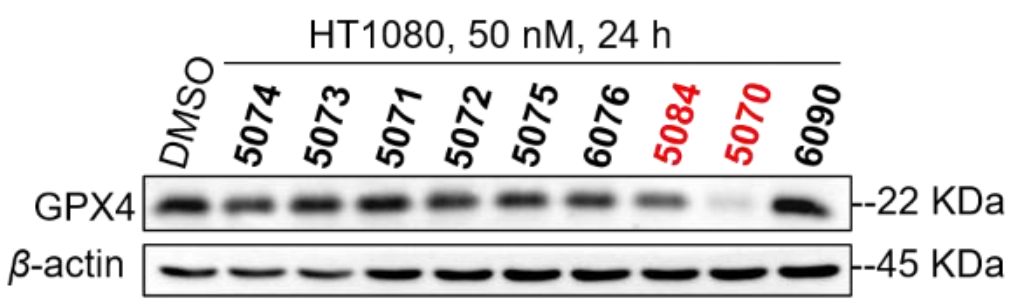

Uncropped blot for Figure 2B

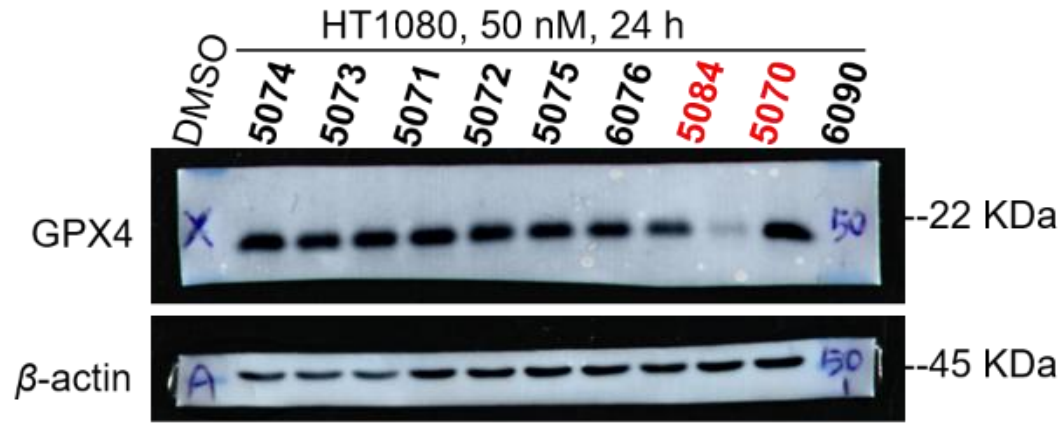

Figure 2B in main text

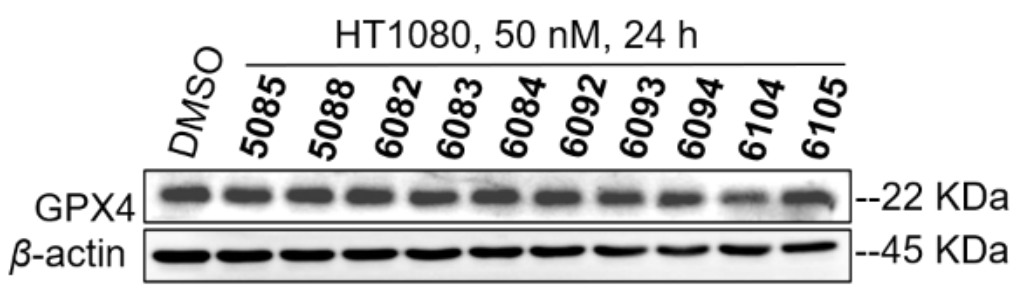

Uncropped blot for Figure 2B

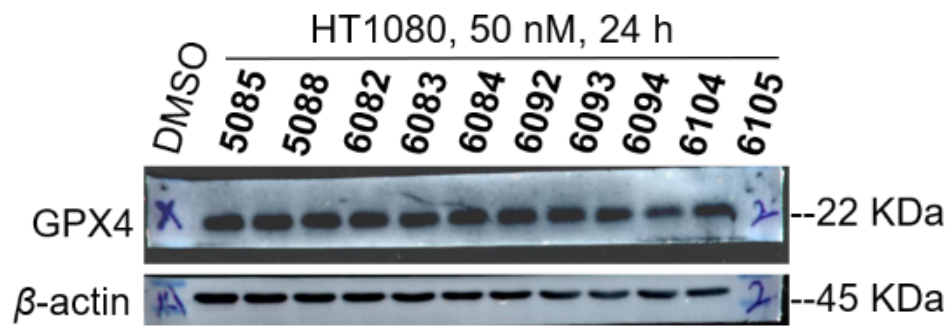

Figure 2C in main text

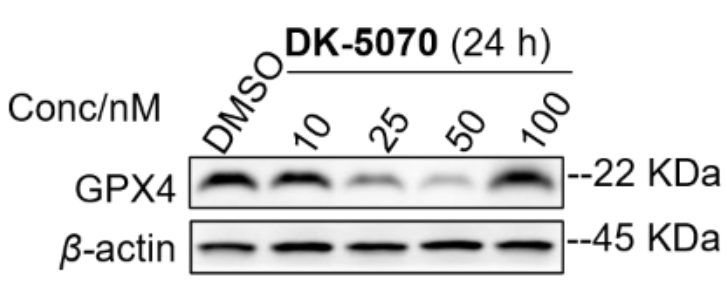

Uncropped blot for Figure 2C

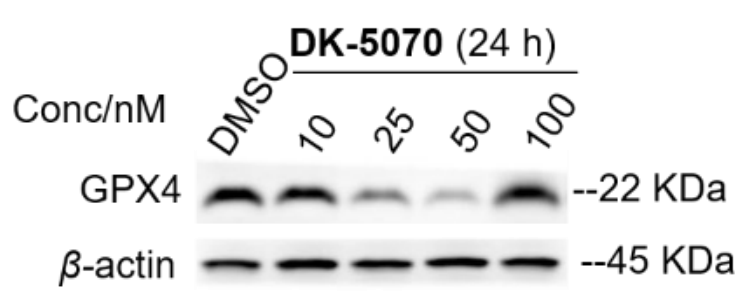

Figure 2D in main text

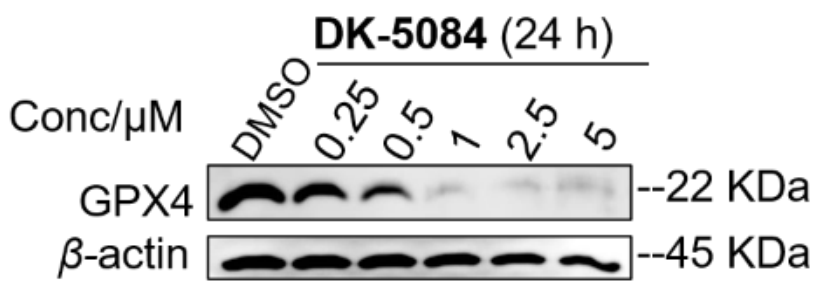

Uncropped blot for Figure 2D

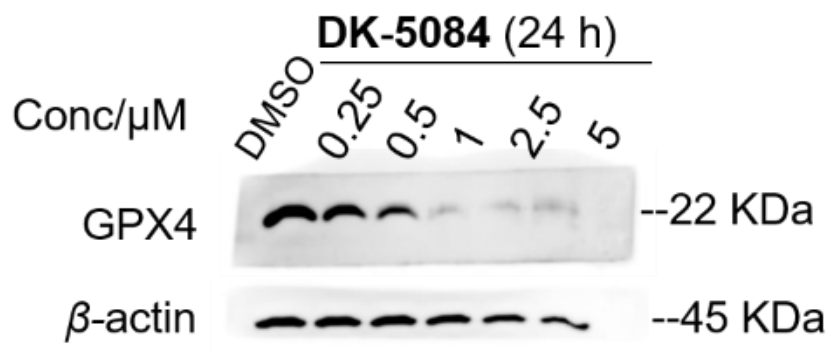

Figure 3B in main text

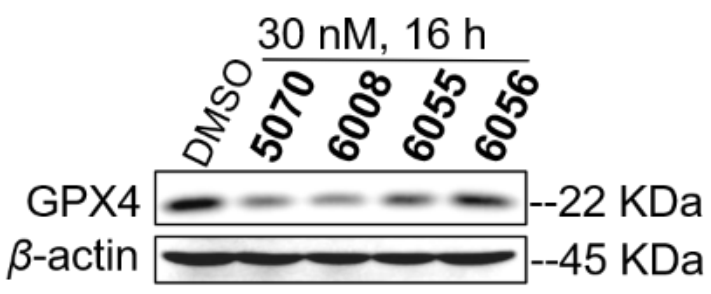

Uncropped blot for Figure 3B

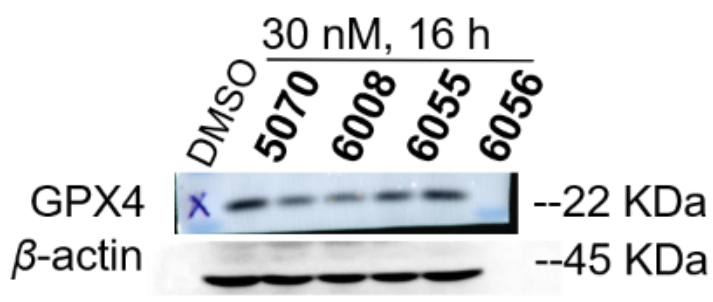

Figure 3C in main text

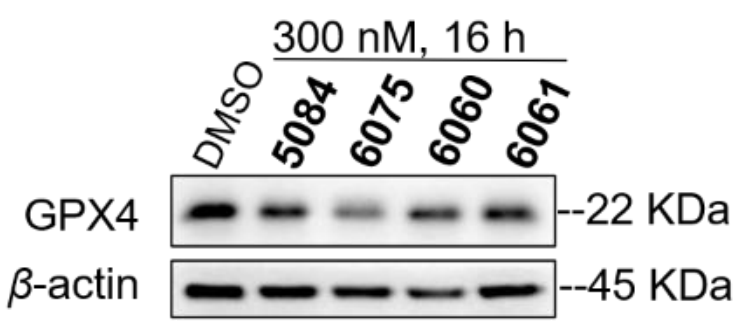

Uncropped blot for Figure 3C

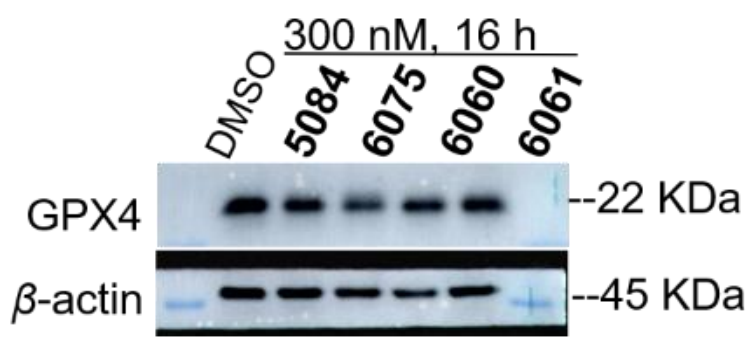

Figure 3D in main text

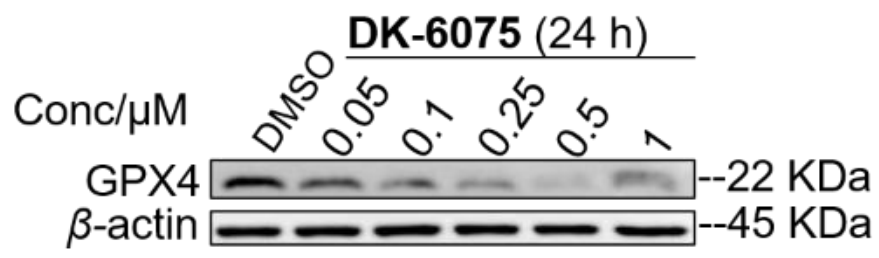

Uncropped blot for Figure 3D

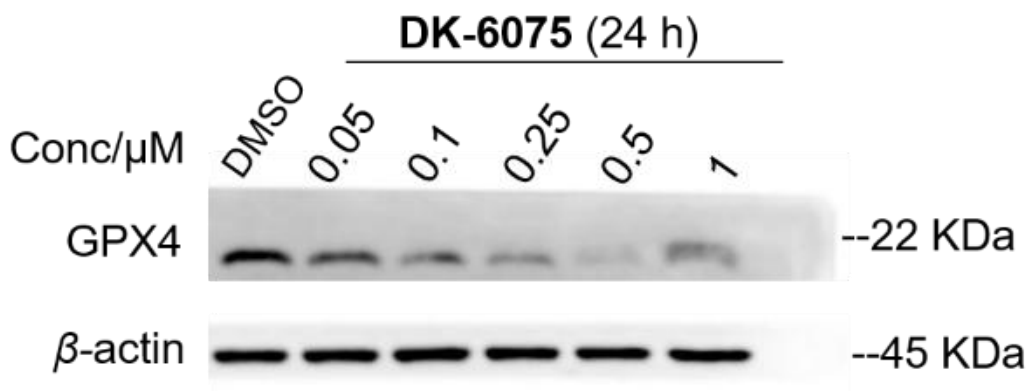

Figure 3E in main text

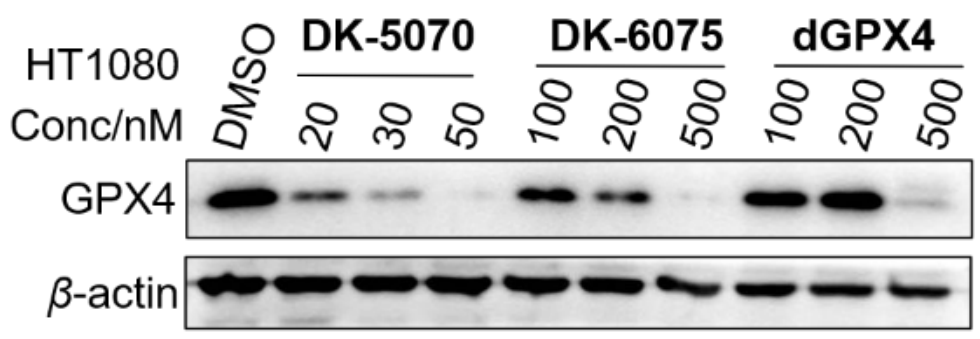

Uncropped blot for Figure 3E

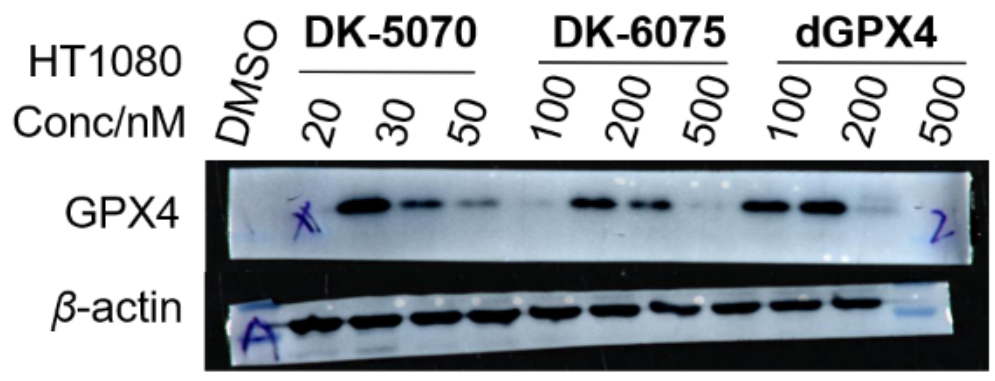

Figure 4B in main text

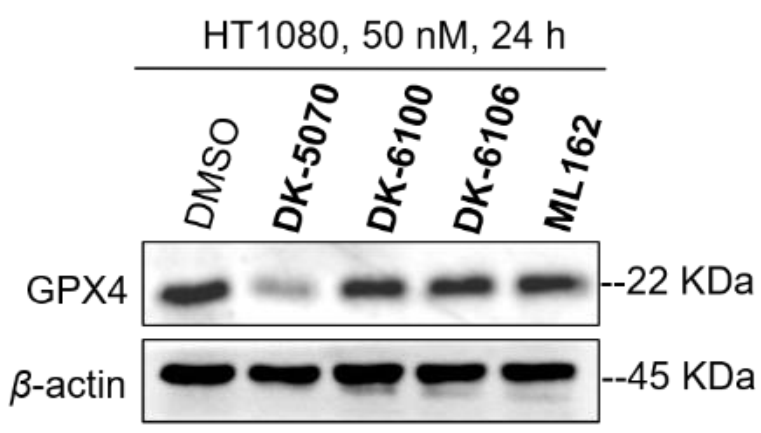

Uncropped blot for Figure 4B

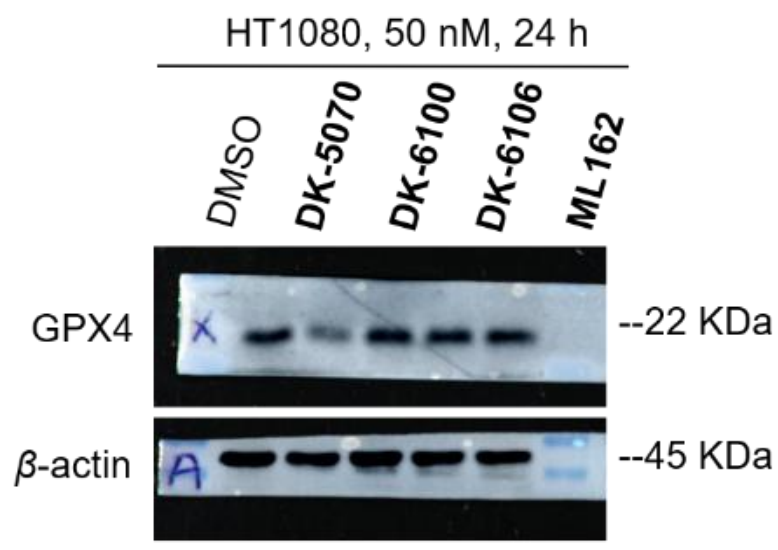

Figure 4C in main text

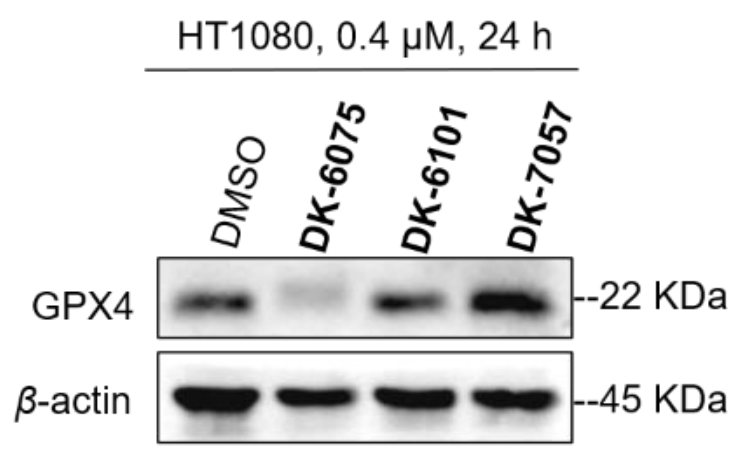

Uncropped blot for Figure 4C

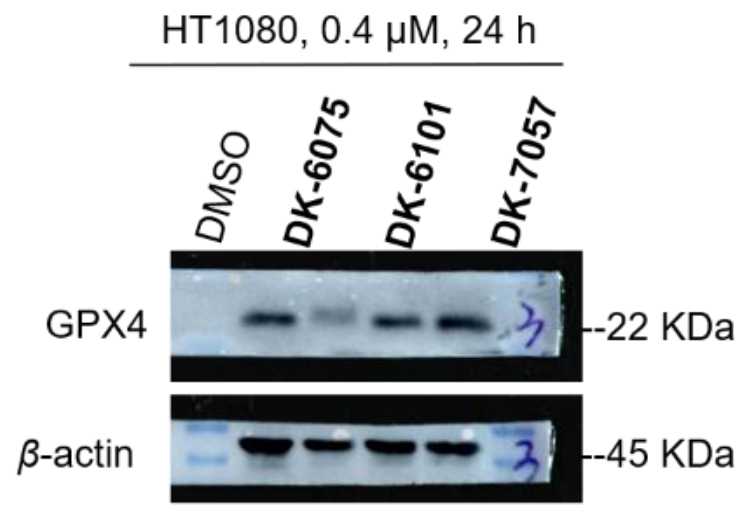

Figure 4D in main text

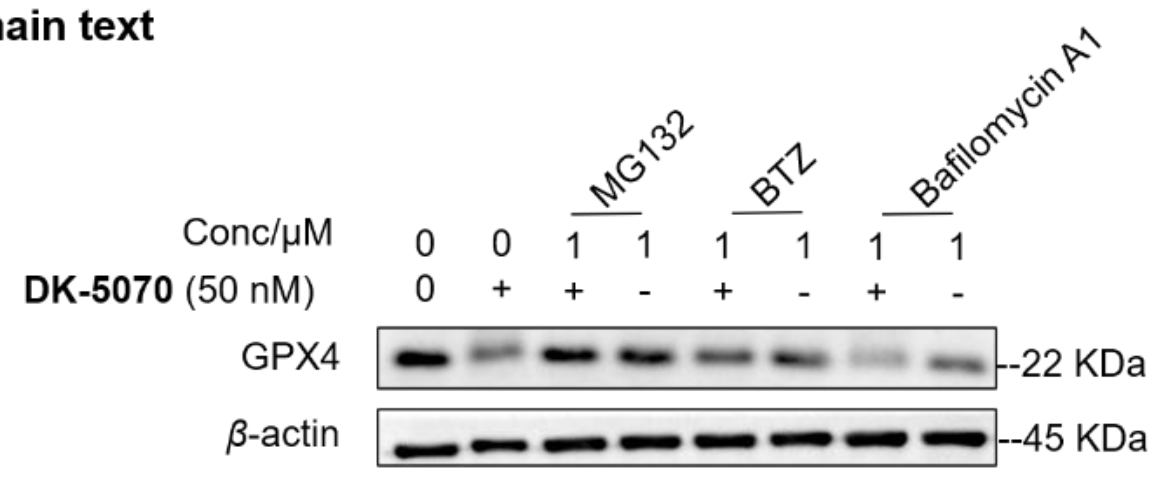

Uncropped blot for Figure 4D

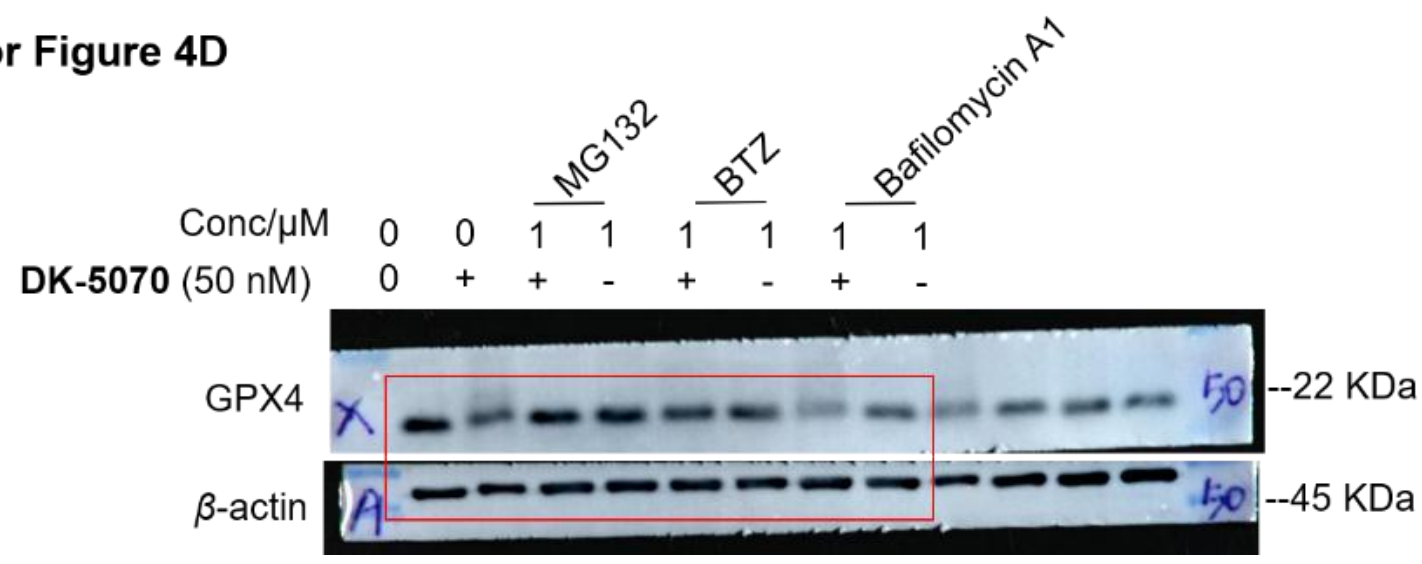

Figure 4E in main text

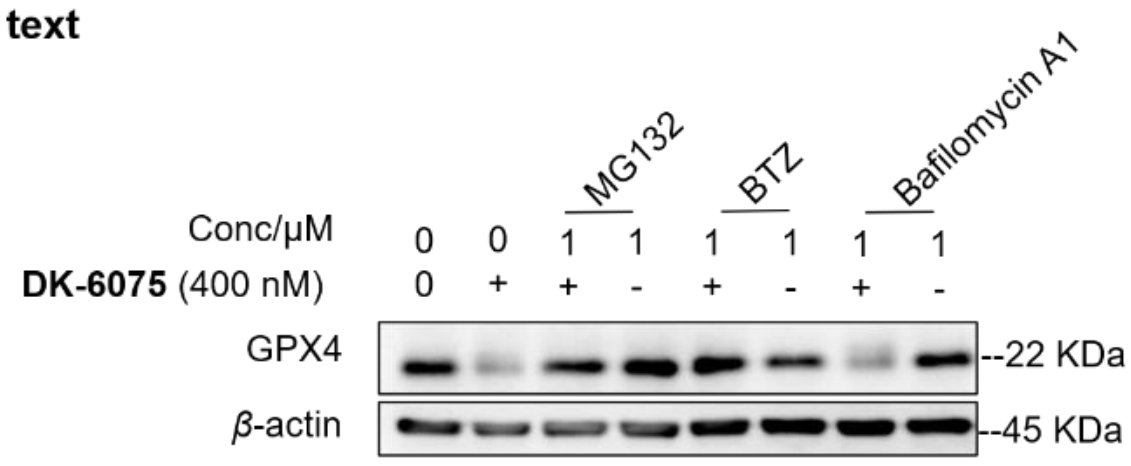

Uncropped blot for Figure 4E

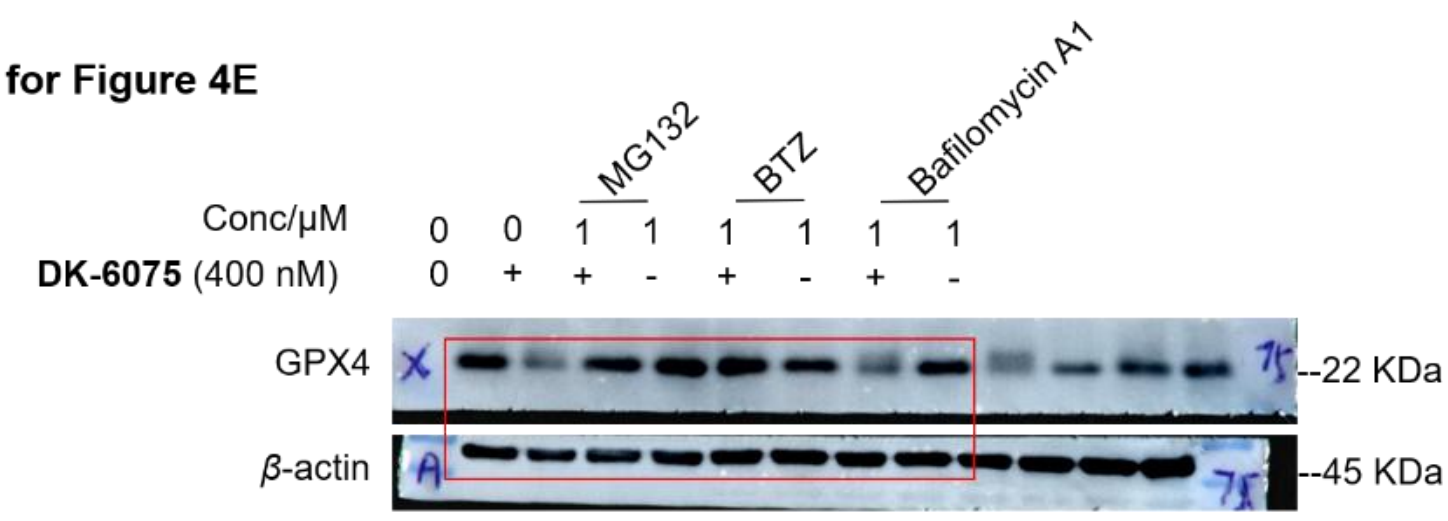

Figure 4F in main text

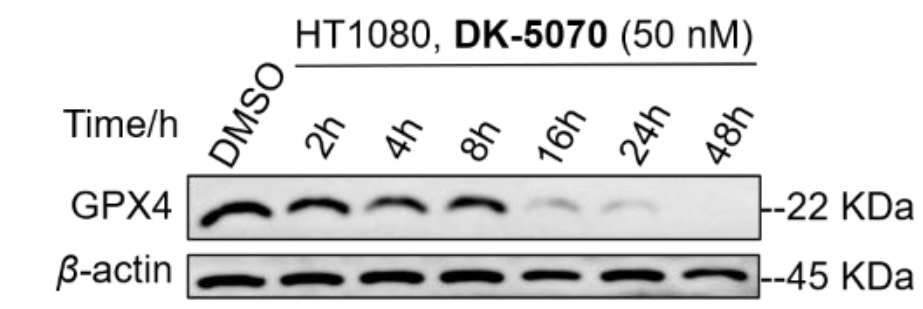

Uncropped blot for Figure 4F

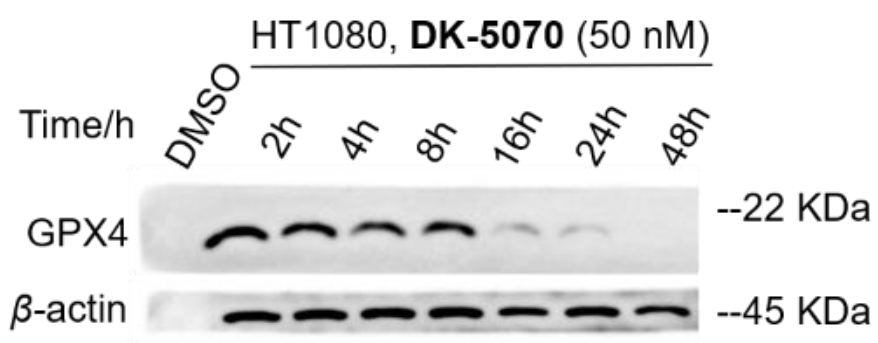

Figure 4F in main text

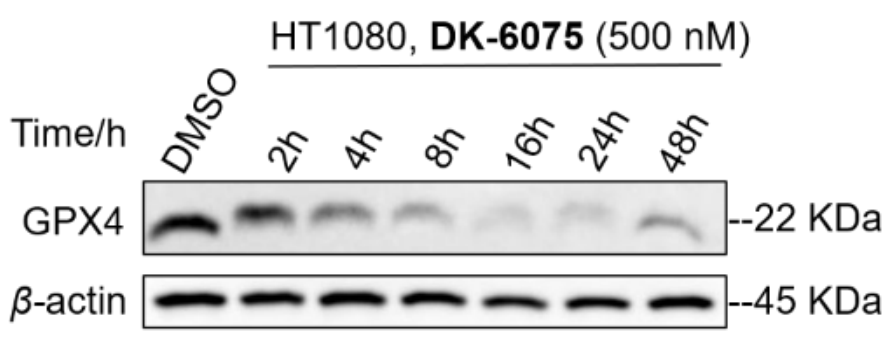

Uncropped blot for Figure 4F

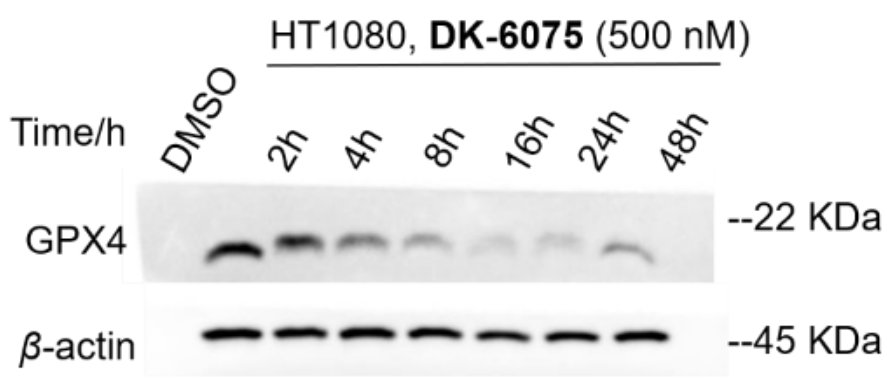

Figure 4G in main text

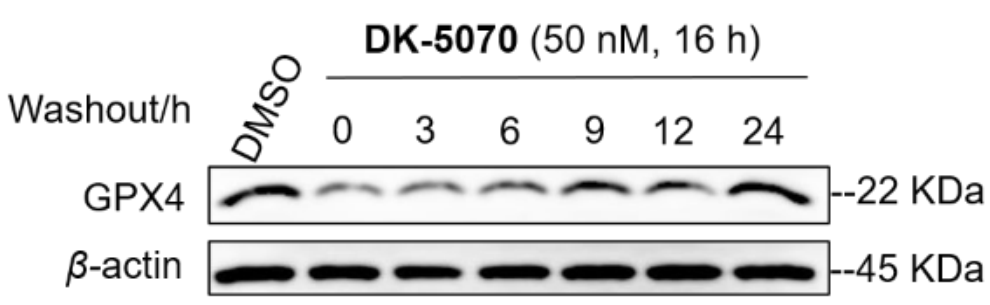

Uncropped blot for Figure 4G

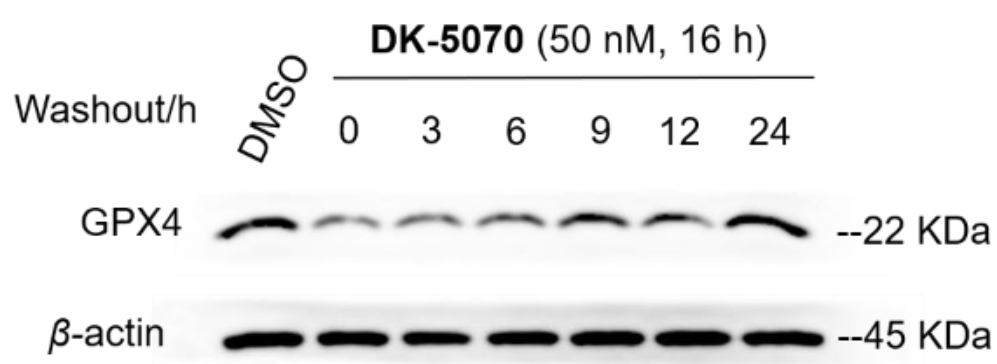

Figure 4H in main text

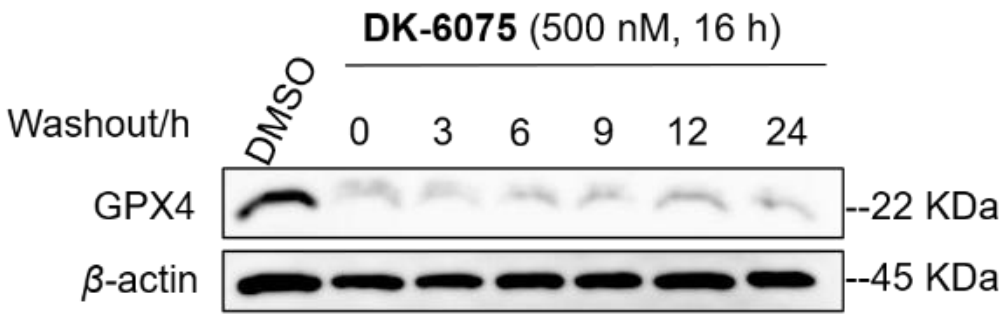

Uncropped blot for Figure 4H

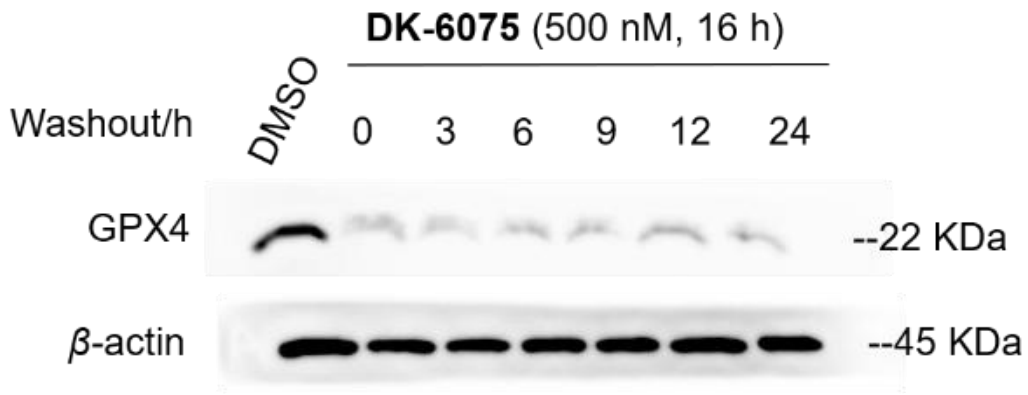

Figure 5G in main text

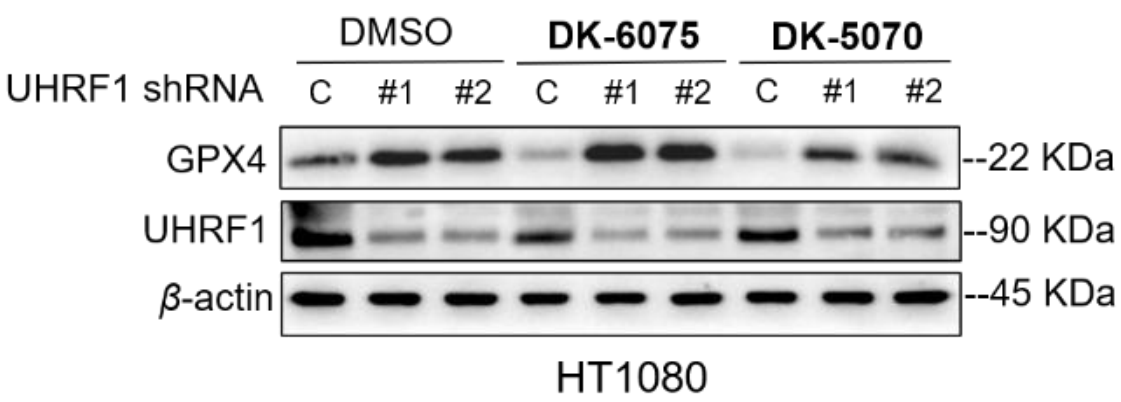

Uncropped blot for Figure 5G

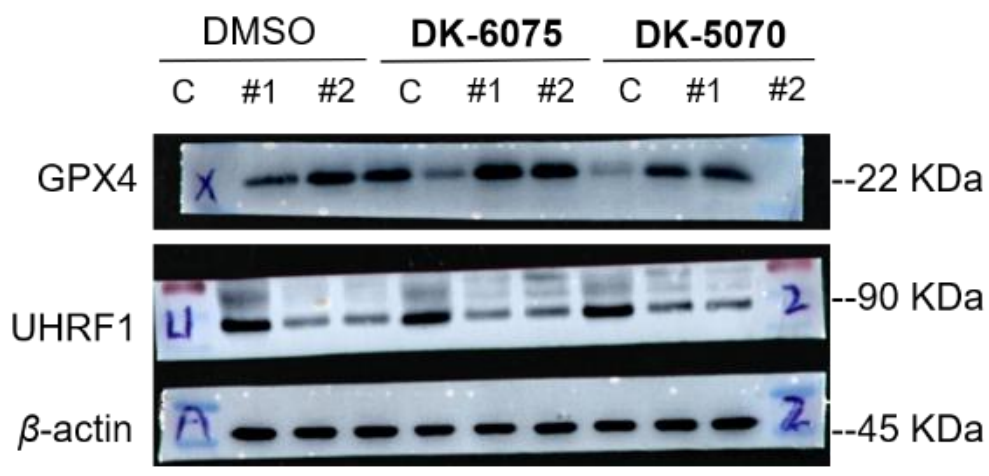

Figure 5H in main text

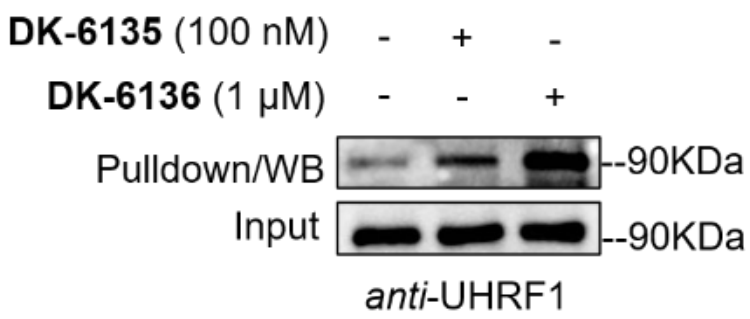

Uncropped blot for Figure 5H

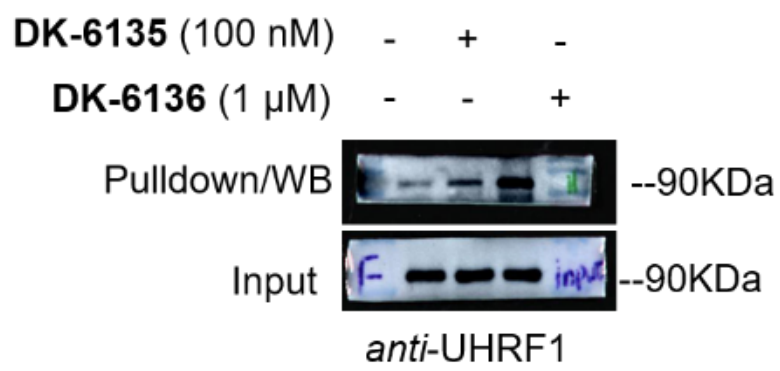

Figure 5I in main text

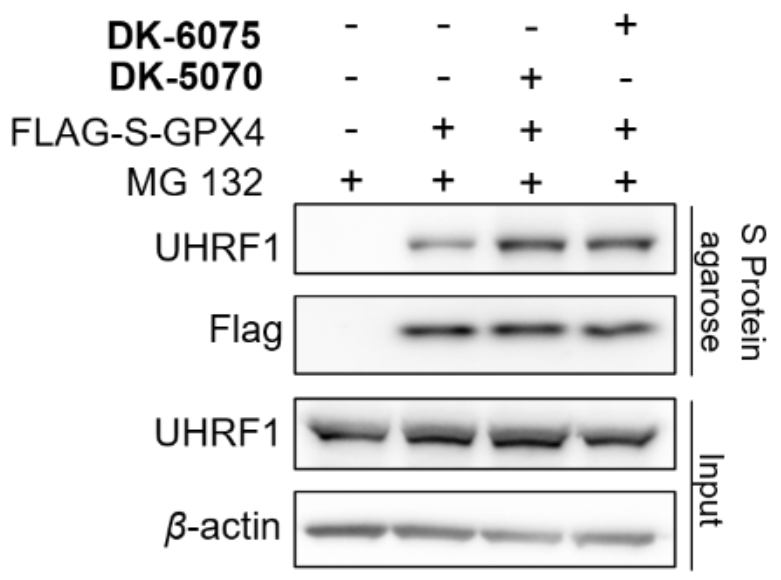

Uncropped blot for Figure 5I

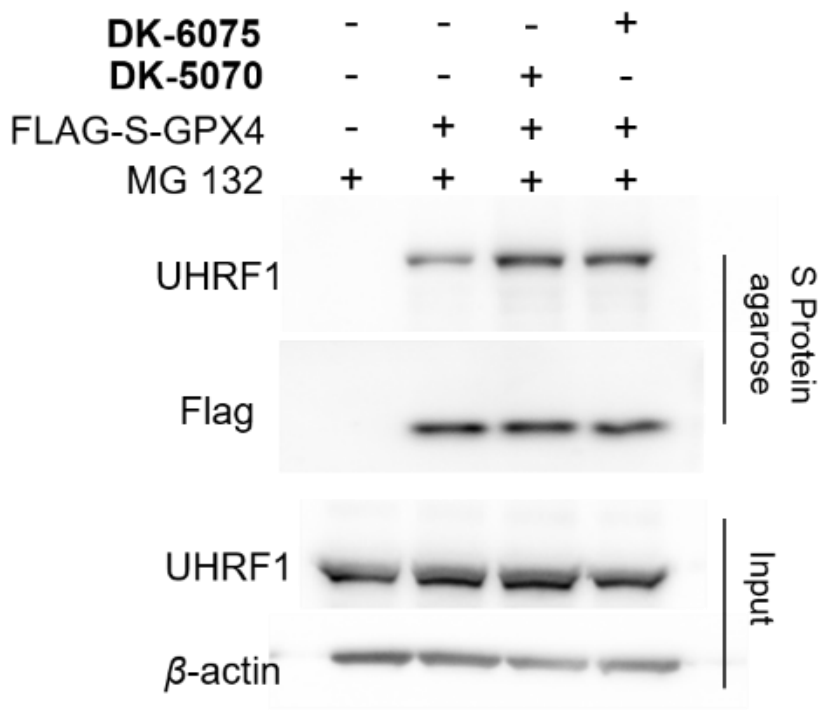

Figure 5K in main text

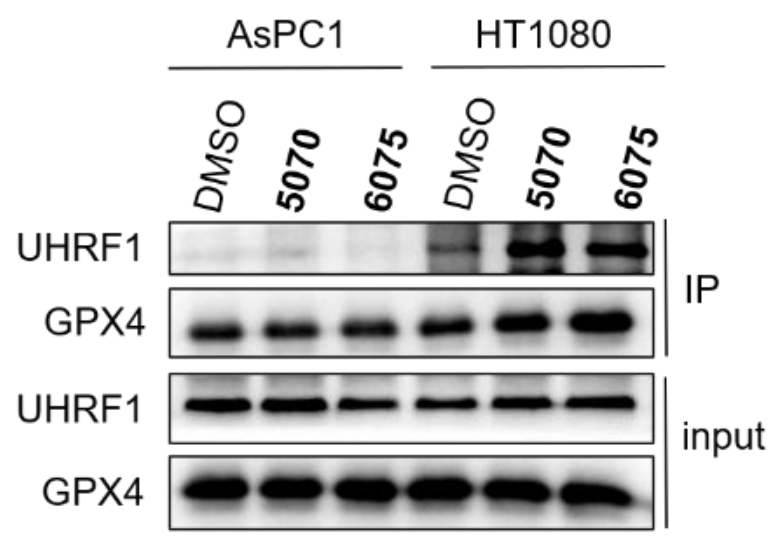

Uncropped blot for Figure 5K

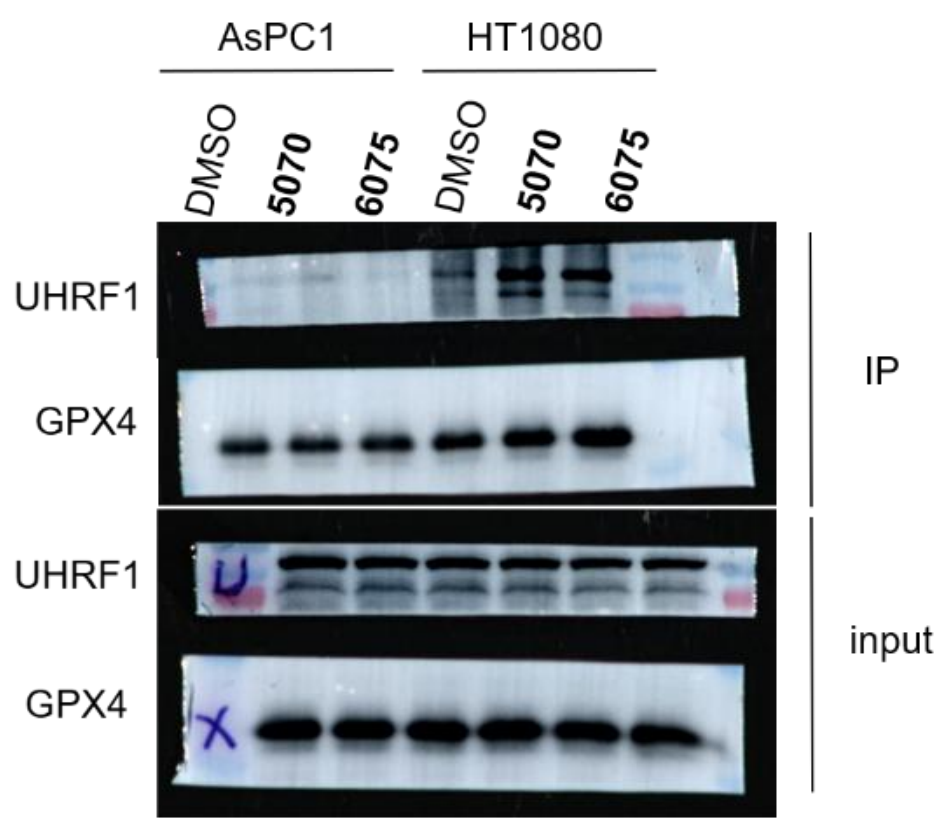

Figure 5J in main text

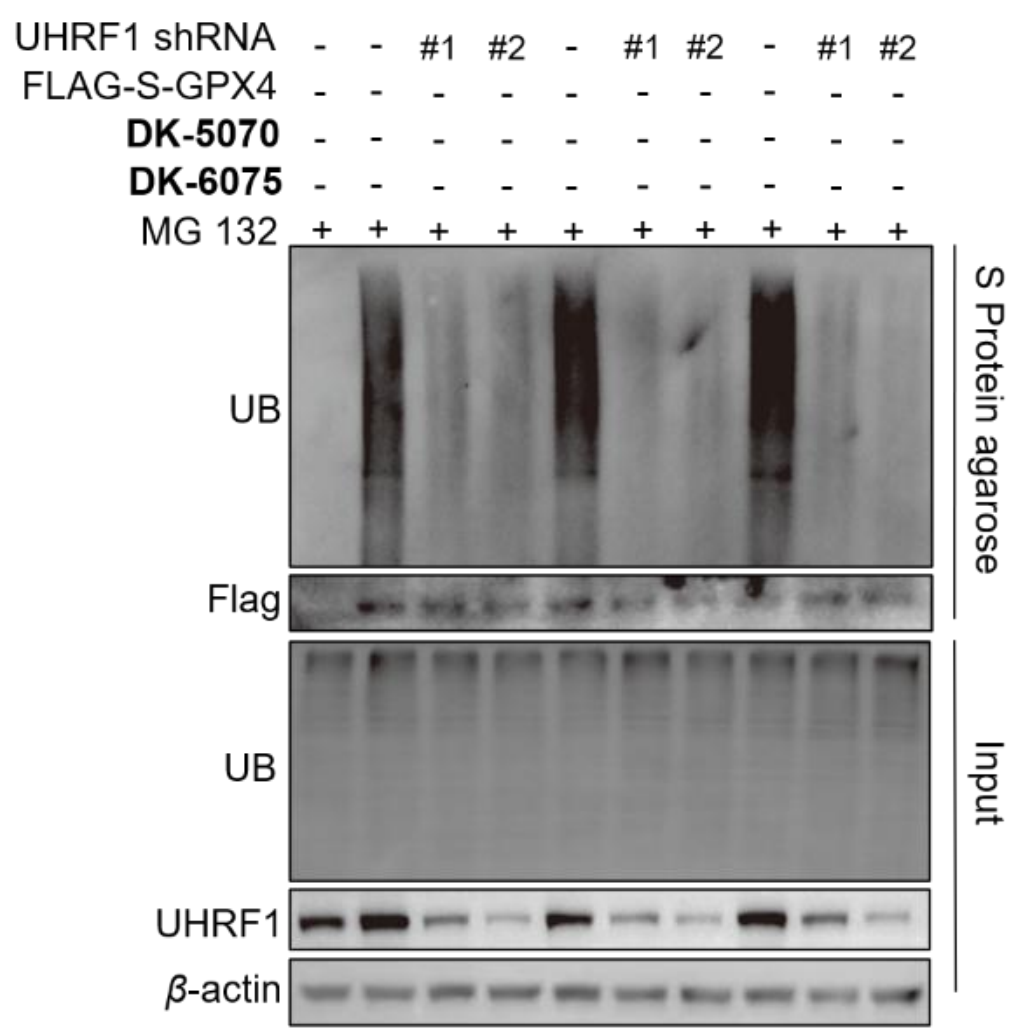

Uncropped blot for Figure 5J

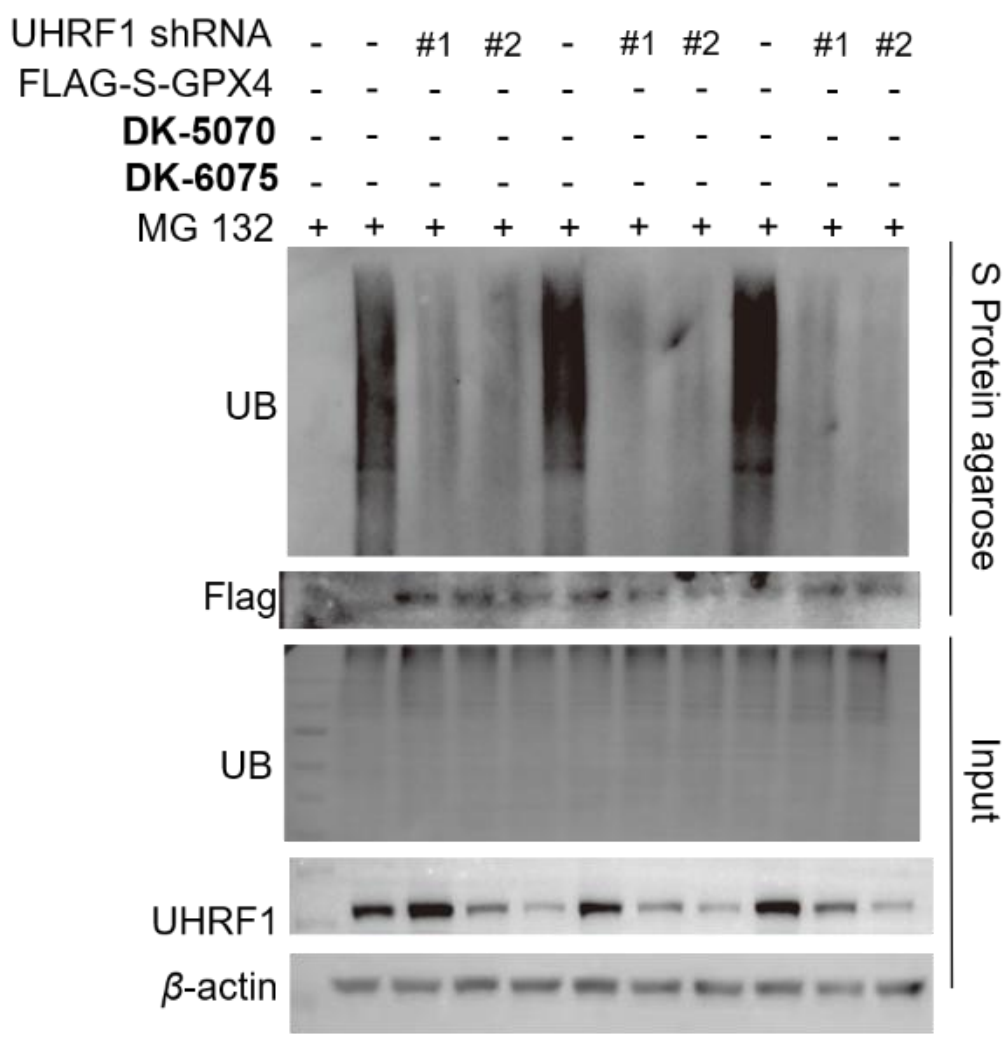

Figure 6D in main text

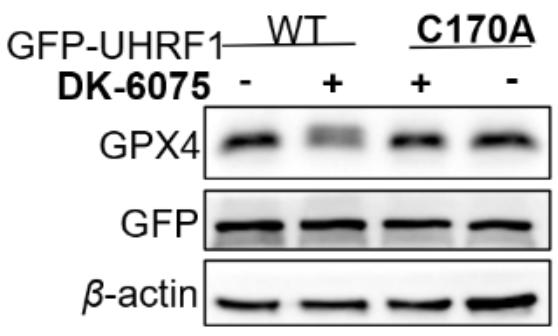

Uncropped blot for Figure 6D

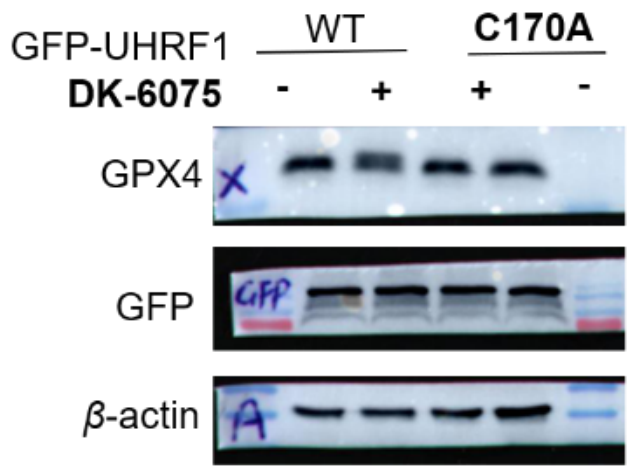

Figure 6E in main text

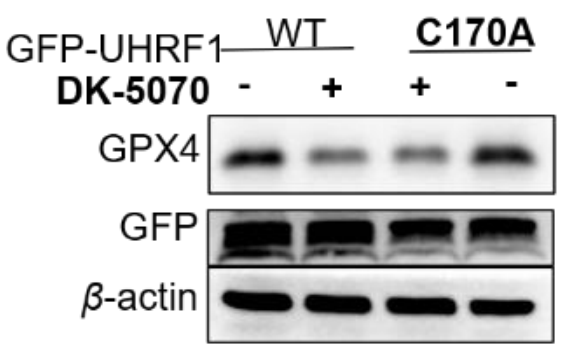

Uncropped blot for Figure 6E

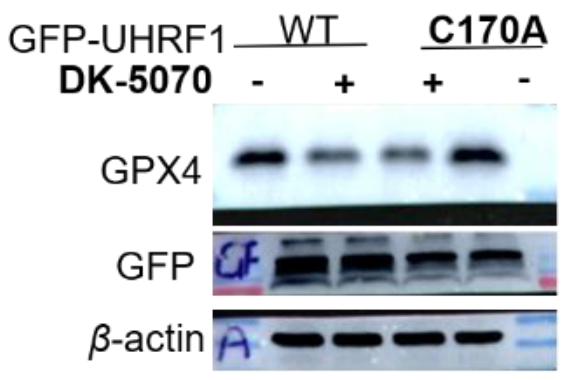

Figure 6F in main text

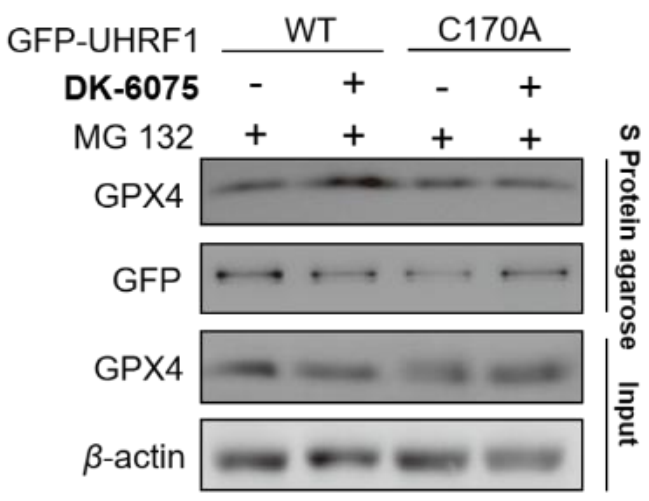

Uncropped blot for Figure 6F

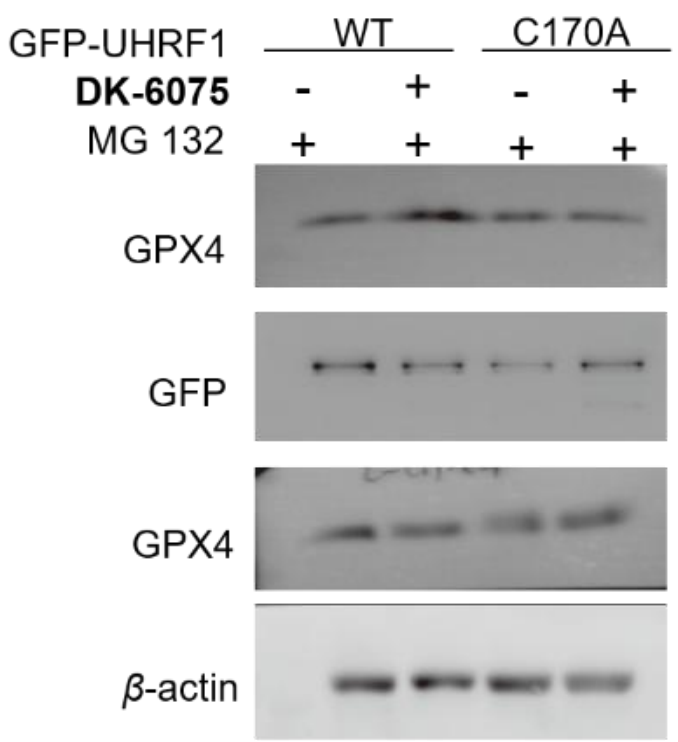

Figure 8H in main text

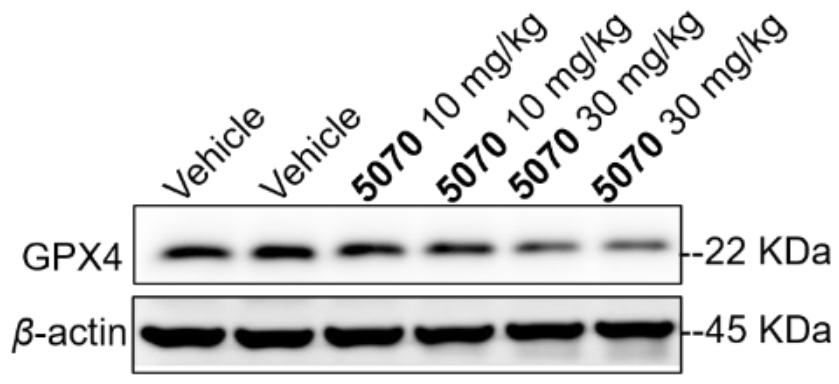

Uncropped blot for Figure 8H

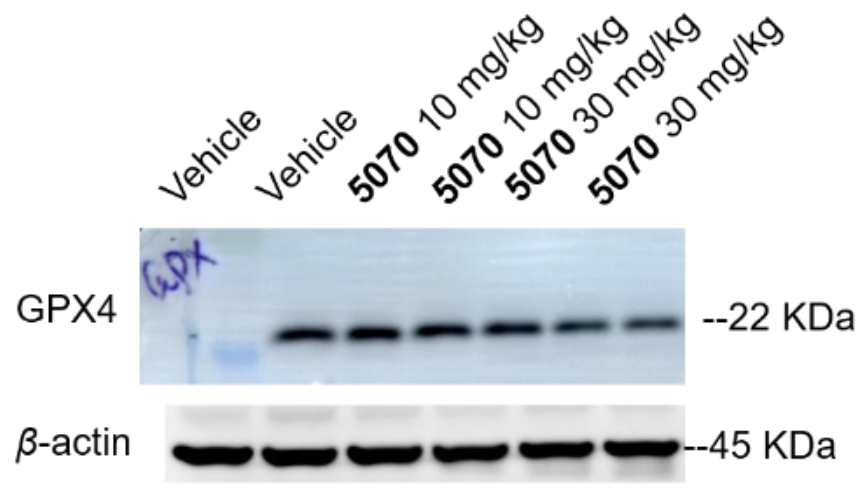

Figure S1A in SI_1

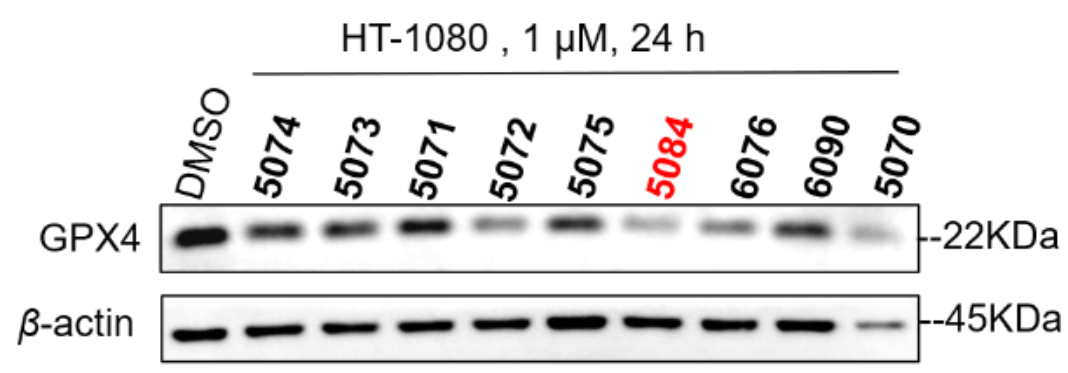

Uncropped blot for Figure S1A

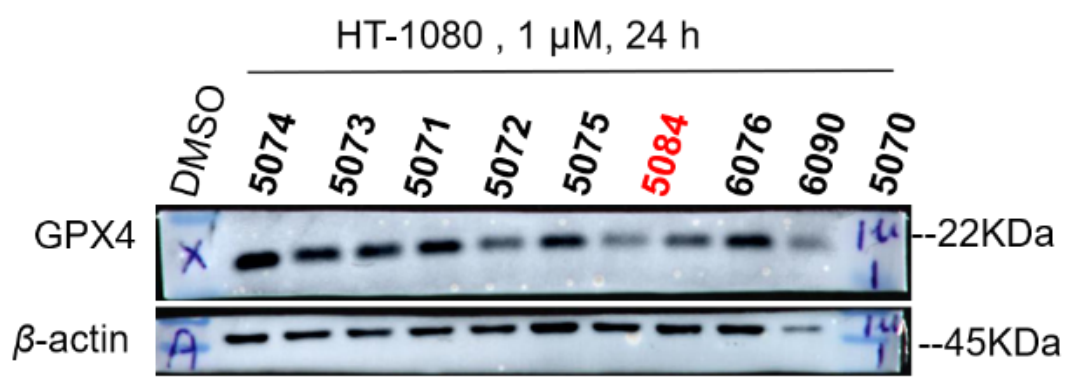

Figure S1A in SI_1

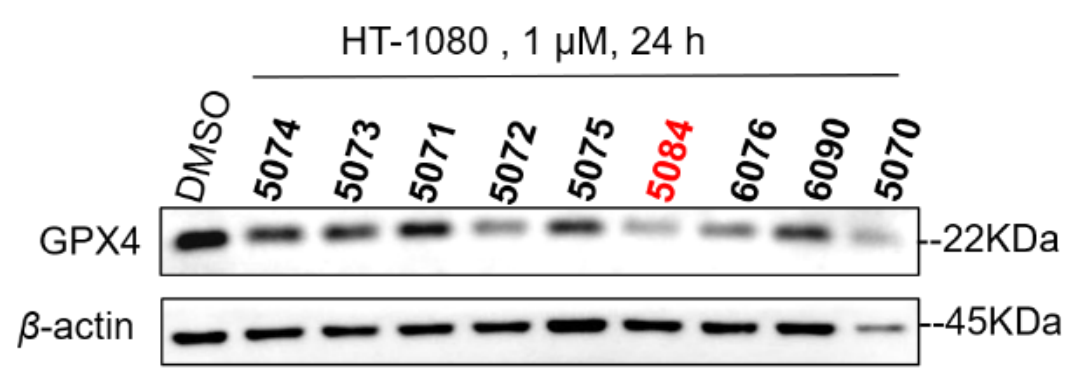

Uncropped blot for Figure S1A

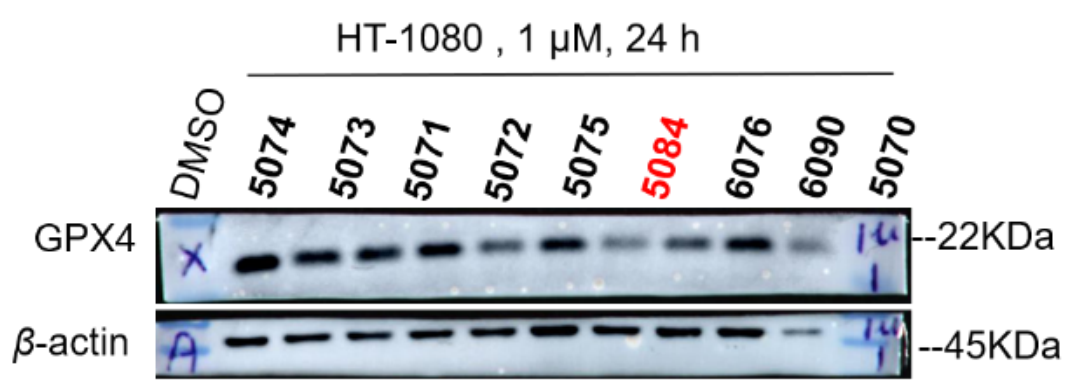

Figure S2A in SI_1

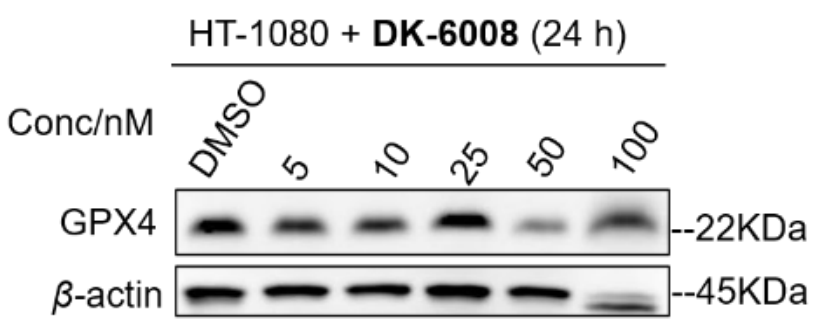

Uncropped blot for Figure S2A

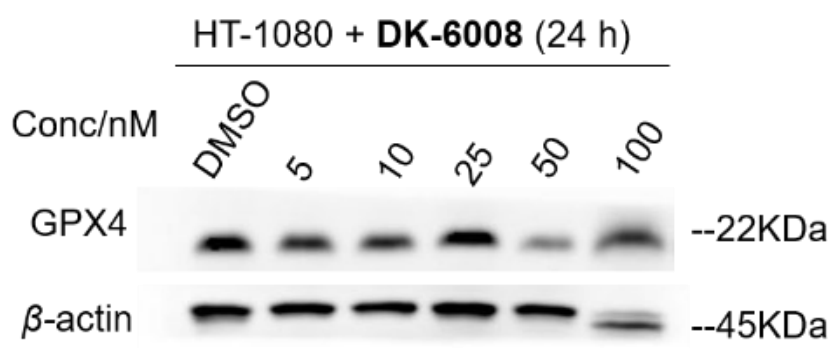

Figure S2D in SI_1

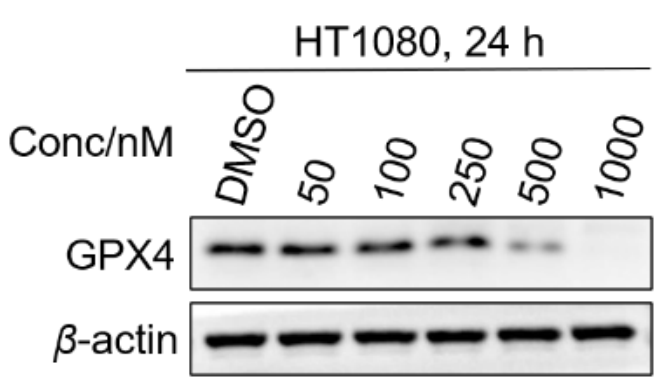

Uncropped blot for Figure S2D

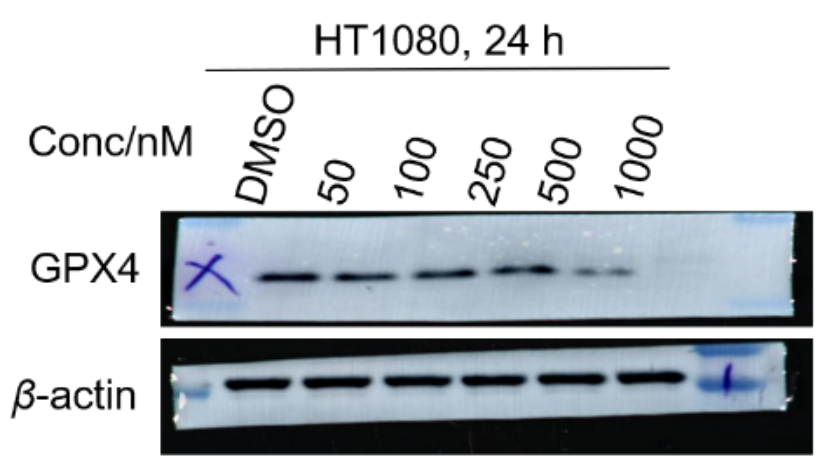

Figure S4A in SI_1

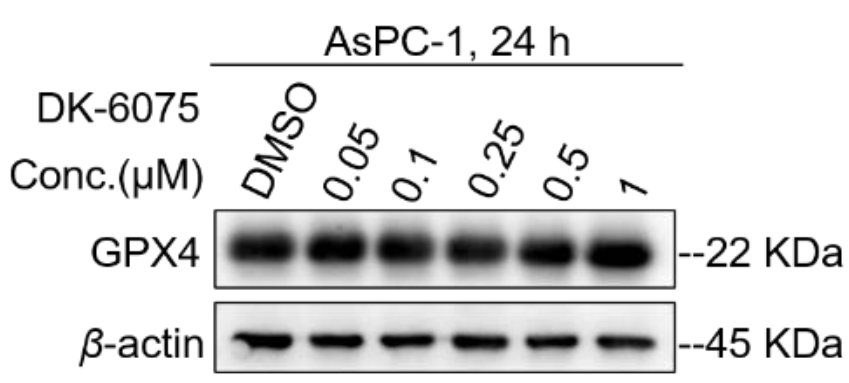

Uncropped blot for Figure S4A

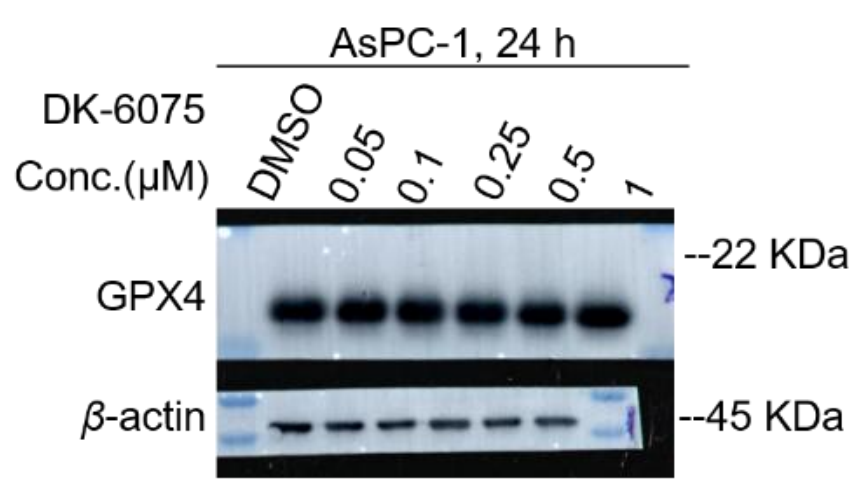

Figure S4A in SI_1

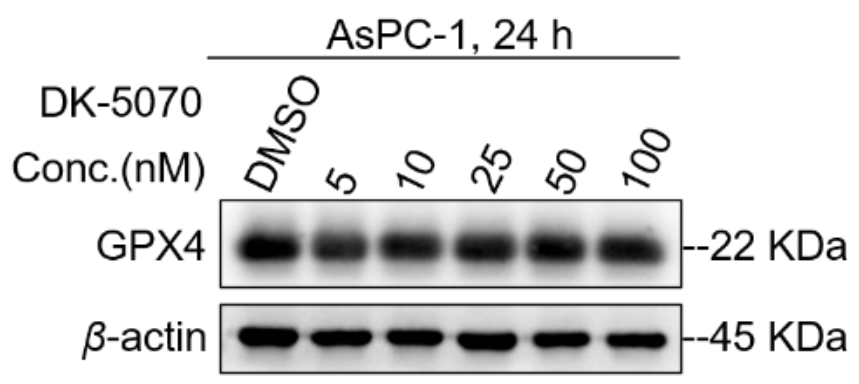

Uncropped blot for Figure S4A

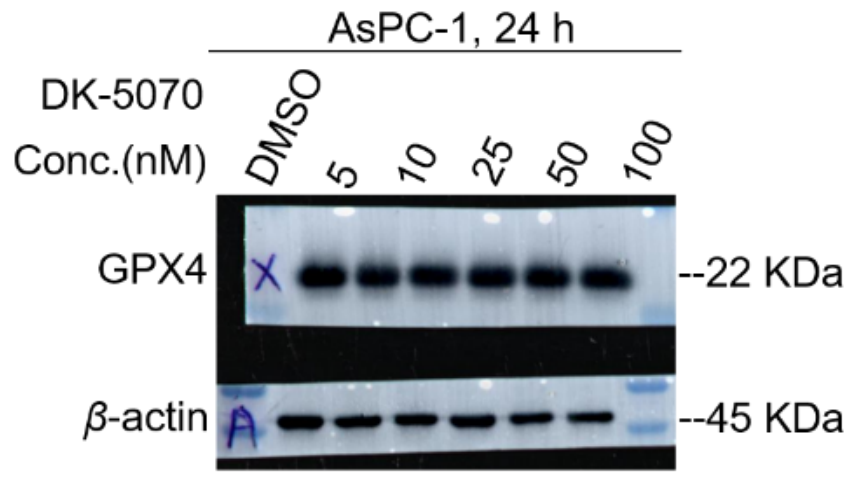

Figure S4B in SI_1

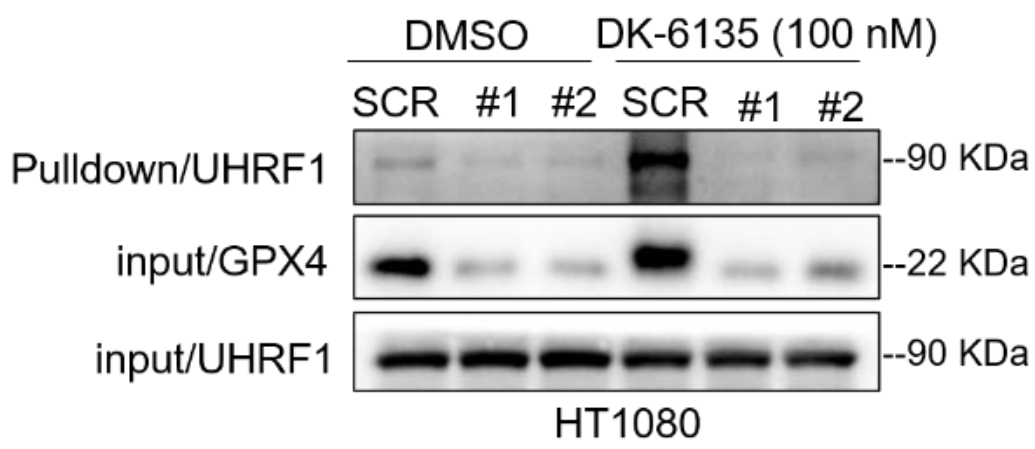

Uncropped blot for Figure S4B

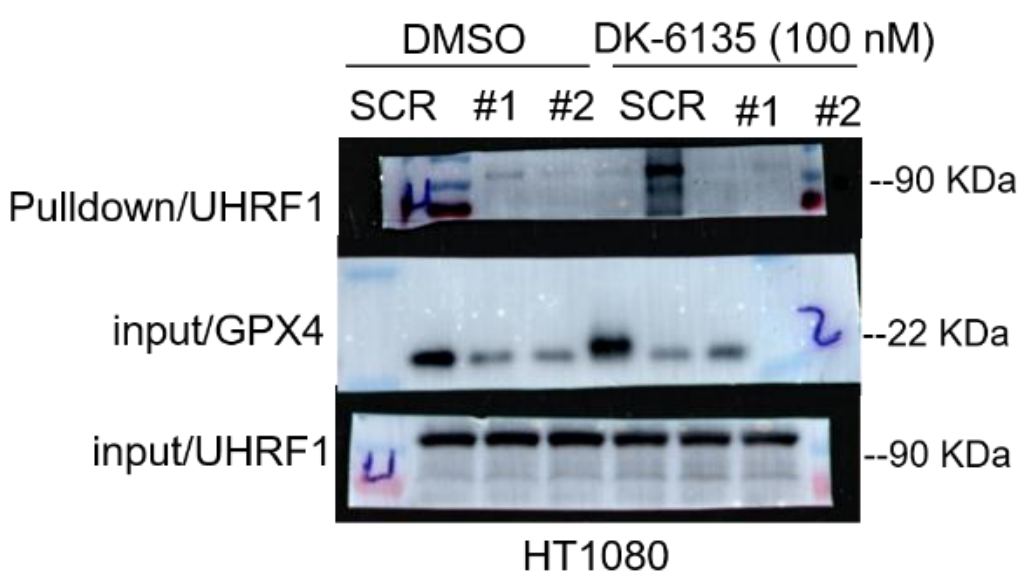

Supplement: Supplementary file 1 [file ADVS-13-e77201-s001.pdf]
